# Dual SYK-HDAC Inhibitor Elicits Striking Efficacy against Acute Myeloid Leukemia: Rational Design, Synthesis, and Biological Evaluation

**DOI:** 10.1021/acs.jmedchem.6c00039

**Published:** 2026-06-09

**Authors:** Anshul Mishra, Wen-Bin Yang, Tsu-Shao Liu, Amandeep Thakur, Ajmer Singh Grewal, Jacek Marczyk, Giovanni Stelitano, Ram Sharma, Mandeep Rana, Gurpreet Singh, Santosh Kumar Guru, Goutam Rath, Saurabh Chawla, Jing Ping Liou, Chun Hsu Pan, Kunal Nepali

**Affiliations:** † School of Pharmacy, College of Pharmacy, 38032 Taipei Medical University, Taipei 110031, Taiwan; ‡ Research Center for Neuroscience, Taipei Medical University, Taipei 110031, Taiwan; § Ph.D. Program in Medical Neuroscience, College of Medical Science and Technology, Taipei Medical University, Taipei 110031, Taiwan; ∥ Department of Pharmaceutical Sciences, Guru Gobind Singh College of Pharmacy, Near Guru Nanak Khalsa College, Yamuna Nagar 135001, Haryana, India; ⊥ Ontonix S.r.l, 23100 Sondrio, Italy; # BioDynLab Ltd., Toronto, Ontario M5B1Y4, Canada; ∇ Department of Biotechnology, 19001 University of Pavia, Via Ferrata 9, 27100 Pavia, Italy; ○ Department of Pharmaceutical Chemistry, 75126 ISF College of Pharmacy, Moga 142001, Punjab, India; ◆ Department of Biological Sciences, National Institute of Pharmaceutical Education and Research, Hyderabad 500037, India; ¶ Department of Pharmaceutics, School of Pharmaceutical Science, Siksha ‘O’ Anusandhan (Deemed to Be University), Bhubaneswar 751003, Odisha, India; †† School of Biological Sciences, National Institute of Science Education and Research (NISER), P.O. Bhimpur-Padanpur, Jatni, Khurda, Bhubaneswar 752050, Odisha, India; ‡‡ Homi Bhabha National Institute (HBNI), Training School Complex, Anushaktinagar, Mumbai 400094, India; §§ Ph.D. Program in Drug Discovery and Development Industry, College of Pharmacy, Taipei Medical University, Taipei 110031, Taiwan

## Abstract

An integrated transcriptomic and survival analysis led to the identification of SYK and HDAC isoforms as age- and FLT3-dependent prognostic markers in acute myeloid leukemia (AML). Driven by the aforementioned, our research group employed a classical ligand-based hybrid pharmacophore strategy to furnish dual-target hybrid frameworks. Antitumor profiling culminated in a tractable bifunctional agent (Compound **14**, dual SYK-HDAC inhibitor) endowed with substantial cell growth inhibitory effects against MV4–11 cell lines (AML cell lines harboring FLT3-ITD mutations). Compound **14** downregulated the expression levels of p-SYK and modulated the expression levels of the biomarkers associated with intracellular HDAC inhibition. Transcriptomic profiling revealed significantly suppressed lipid-associated metabolic pathways with Compound **14** treatment. Moreover, Compound **14** demonstrated an impressive pharmacokinetic profile and exerted significant antitumor efficacy in the FLT3-ITD-positive AML xenograft mouse model (**approximately 80% decrease in tumor mass**). Also, biochemical blood analysis and histopathological studies revealed that Compound **14** demonstrated a good safety profile.

## Graphical abstract

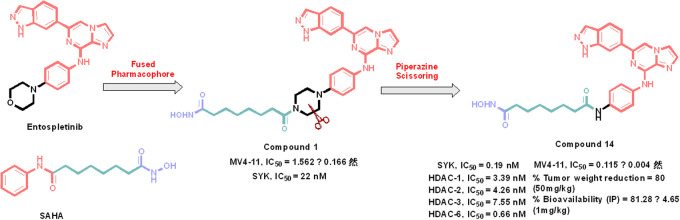

## 1 Introduction

Acute myeloid leukemia (AML), a highly aggressive hematological malignancy, is characterized as a molecularly complex and heterogeneous disease that arises from the uncontrolled proliferation and differentiation of myeloid cells. This uncontrolled proliferation results in the accumulation of immature myeloid precursors in bone marrow and peripheral blood (abnormal hematopoiesis).^1,2^ Current therapeutic approaches for patients with AML include hypomethylating agents (HMAs) and hematopoietic stem cell transplantation (HSCT) as well as the use of intensive combination chemotherapy.^3,4^ The aforementioned treatment methods are associated with a high recurrence rate,^5–7^ which underscores the urgent need for novel and effective treatment modalities. It is worth noting that significant research has been conducted to establish the therapeutic profile of small-molecule inhibitors in AML, *viz*., FLT3 inhibitors, IDH inhibitors, and Bcl-2 inhibitors. Though the aforementioned classes of inhibitors have elicited substantial efficacy in various AML subsets, their candidacy as futuristic therapeutic options is currently marred by modest pharmacodynamic resilience, toxicity, and drug resistance issues.^8^ Thus, the chemical toolbox of anti-AML agents is required to be enriched with magic bullets that can outwit the notoriety of AML cells and overcome the constraints associated with the existing therapies.

Histone deacetylase has emerged as the most sought-after target for the fabrication of small-molecule antitumor templates. As such, HDACs regulate gene expression by deacetylation of lysine residues on histone and nonhistone proteins. Literature precedents reveal that abnormal expression of HDAC isoforms and oncogenic HDAC-containing transcriptional complexes are involved in diverse malignancies.^9,10^ HDAC inhibitors induce relief of transcriptional repression in cancer and are considered to be a key to epigenetic cancer therapy. Substantial efforts invested in the field of HDAC inhibitor-based antitumor drug discovery have culminated in the US FDA approval of four HDAC inhibitors, *viz*., **SAHA** for cutaneous T-cell lymphoma, **Romidepsin** for peripheral T-cell lymphoma, **Belinostat** (**PXD-101**) for relapsed or refractory peripheral T-cell lymphoma and **Panobinostat** for multiple myeloma (Figure 1S, Supporting Information).^11^ Notably, aberrant expression of class I (HDAC1, HDAC2, and HDAC3) and class II (HDAC6) HDAC isoforms has been reported in AML, thereby labeling them as key regulators of AML pathogenesis. Key disclosures in this context are (i) significant elevation of HDAC1 expression in refractory and drug-resistant AML cells,^12^ (ii) overexpression of HDAC2 in AML cells *via* interaction with the oncogenic transcription factor c-Myc,^13^ (iii) upregulation of HDAC3 in AML cells *via* AKT activation,^14^ (iv) elevated HDAC6 expression associated with the hedgehog signaling pathway and expression of resistance genes like ABCC1 and ASX in AML patients.^15,16^ Despite the aforementioned precedential claims, the therapeutic bandwidth of HDAC inhibitors in AML has been restricted by modest clinical responses, which in turn raises the intrigue in terms of “not so satisfactory” anti-AML effects of this class of epigenetic inhibitors. Nevertheless, the application constraints of HDAC inhibitors in AML have been tactically handled lately *via* combination therapy. Notably, DNA damage in AML cell lines was exerted *via* a combination of Panobinostat with cytarabine,^17^ reversal of resistance in FLT3-ITD mutant cells attained *via* a cocktail of novel class 1 HDAC inhibitor (IHCH9033) in combination with quizartinib,^18^ and synergistic efficacy in FLT3-ITD acute myeloid leukemia, leading to enhanced apoptosis achieved through chidamide and cytarabine combination,^19^ representing some optimistic outcomes of combination therapy with HDAC inhibitors in AML. Thus, balanced modulation of another therapeutic target along with HDAC isoforms is anticipated to steer the wheels of the HDAC inhibitor drug discovery campaign for AML.

Spleen tyrosine kinase (SYK) is expressed mainly in hematopoietic cells such as B-lymphocytes and myeloid cells.^20,21^ SYK mediates the BCR signaling, activating downstream pathways through BTK and PI3K. It is also characterized by atypical expression and activation in specific leukemia subtypes such as chronic lymphocytic leukemia (CLL), acute myeloid leukemia (AML), and diffuse large B cell lymphoma (DLBCL).^22^ The synthetic bank of SYK inhibitors has quite a few entries as investigational small-molecule SYK inhibitors (Figure 1S), *viz*., **Fostamatinib** for the treatment of diffuse large B cell lymphoma and lymphoma (Phase II clinical trials),^23,24^ **entospletinib** for the treatment of CLL, MCL, and DLBCL (Phase I/II),^25^ **lanraplenib** (GS-9876) for acute myeloid leukemia [Phase I/II (NCT02959138/NCT02885181)],^26^ **cerdulatinib** for DLBCL [phase II (NCT01994382)],^27^ and **sovleplenib**, for mature B cell neoplasms (Phase I NCT02857998).^28^ It is noteworthy to mention that SYK plays a prominent role in regulating AML-driven genes such as Hoxa9/Meis1, and the integrin Fc receptor signaling in AML.^29–33^ Key disclosures from the studies conducted in pursuit of assessing the efficacy of SYK inhibitors in AML underscore their ability to inhibit AML cell proliferation, induce apoptosis in FLT3/ITD mutant human AML cells, and diminish AML stem cell survival.^30–35^ Despite these revelations, the monotonic application of SYK inhibitors as a therapeutic strategy in AML has garnered divided opinions from the drug discovery teams owing to the complex and multifactorial nature of AML, coupled with compensatory activation of alternative survival pathways in AML. The aforementioned is backed by promising outcomes demonstrated by the combination therapy involving SYK inhibitors in AML in terms of leukemic burden reduction, overcoming resistance mechanisms, and disrupting the mitochondrial biogenesis (leukemia stem cells).^36–38^ Notably, the combination of entospletinib with consolidation chemotherapy has progressed to a Phase 3 clinical trial in NPM1-mutated AML patients, and the lanraplenib–gilteritinb (FLT inhibitor) combination for FLT3 mutated AML patients is being evaluated in clinical trials. In a nutshell, the prospects of SYK inhibitors in AML heavily rely on the application of drug cocktails of mechanistically specific chemotypes or bifunctional adducts (dual inhibitors) capable of simultaneously addressing biochemically related targets in pursuit of attaining amplified therapeutic benefits in AML.

Literature precedents reveal that SYK kinase plays a critical role in promoting leukemic survival and lineage blockade by activating key transcriptional networks in cancer.^39–41^ Specifically, SYK activation triggers the downstream activation of NF-κB and STAT signaling, pathways that are known to be modulated by HDAC activity.^42,43^ HDACs play an important role in maintaining the chromatin environment that supports these oncogenic programs.^44–47^ Inhibition of specific HDAC isoforms weakens these transcriptional circuits, disrupting critical signaling pathways, such as ERK and DNMT1 (DNA methyltransferase 1), which have been implicated in SYK-associated signaling.^48,49^ Collectively, these findings indicate a functional convergence of SYK-mediated signaling and HDAC-regulated epigenetic mechanisms at multiple levels, including epigenetic regulation, nonhistone protein modification, SYK-mediated transcriptional regulation, and apoptosis.^50^ Based on this functional convergence, concurrent modulation of HDAC and SYK for extracting synergistic efficacy in AML appears to be a rational approach, which can either be accomplished *via* combination therapy or through rationally fabricated chimeric chemical architectures (dual inhibitors). Notably, dual inhibitors have gained significant traction over drug combinations owing to a multitude of advantages, *viz*., predictable pharmacokinetic profile, better safety, improved patient compliance, simpler clinical trial design, and a lower risk of drug resistance.^51^ In alignment with the recent trends in medicinal chemistry tilted toward the construction of dual inhibitory chemotypes as superior alternatives to drug cocktails, the hypothesis of modulating SYK and HDAC for enhanced efficacy in AML was tested in the present study *via* the creation of dual SYK-HDAC inhibitors as anti-AML scaffolds.

## 2 Results and Discussion

### 2.1 Drug Design Strategy

To support the proposed cotargeting approach, we analyzed the publicly available AML transcriptomic data sets using the GEPIA3 platform. The analyses were intended to support target selection and did not establish a driver role of SYK or HDAC isoforms in AML pathogenesis. The analysis outcome indicated that SYK and HDAC pathways are clinically associated with aggressive AML phenotypes, thereby providing a rationale for simultaneous targeting of these targets (Supporting Information, Section 2—**Clinical Transcriptomic Analysis**, Figure 2S). Further, the translation of envisionment to practical grounds necessitated the identification of well-established pharmacophores of SYK and HDAC for the construction of chimeric chemical architectures. Gladly, HDACs have been established as the most validated target for the generation of bifunctional small-molecule inhibitors, largely due to the well-defined and modular pharmacophore model. This generalized HDAC inhibitory template comprises three components, *viz*., CAP (surface recognition part), linker, and zinc-binding motif. Specifically, the CAP demonstrates interactions with the amino acid residues around the entrance (active site), a hydrophobic linker occupies the tunnel, and chelation of the zinc ion is achieved by the zinc ion.^51^ Notably, the remarkable success of the aforementioned template is attributed to its structural plasticity, which enables the accommodation of diverse pharmacophoric fragments as the surface recognition part. This flexibility has been widely exploited to direct the antitumor efficacy of the resulting scaffolds toward a particular malignancy. Lately, our research group has been actively working on the design of bifunctional HDAC inhibitors.^52–56^ Notably, the outcomes of our drug discovery campaign on chimeric HDAC inhibitors validate the strategy of installing a meticulously curated surface recognition motif in pursuit of addressing specific malignancies. Building upon these insights, insertion of a SYK inhibitory fragment within the HDAC inhibitory template was viewed as a potential strategy to furnish potent anti-AML effects.

### 2.2 Recruitment of SYK Inhibitory Fragment as the Surface Recognition Part and Its Structural Interrogation

Structural analysis of the investigational SYK inhibitor was done to pinpoint a structurally flexible SYK inhibitor that could be installed as the surface recognition part of the HDAC inhibitory model. Notably, the chemical architecture of entospletinib was considered apt for the design of hybrid scaffold construction (Figure 3S, Supporting Information). Notably, the selection of entospletinib as a SYK binding pharmacophore was made on the basis of its structural attributes. Entospletinib comprises a conserved hinge-binding heteroaromatic core that is involved in key hydrogen bond interactions with the hinge region residues. Specifically, the N-1 atom of the imidazopyrazine core accepts a hydrogen bond from the backbone NH of Ala451, and the aniline NH interacts with the carbonyl oxygen of the same residue. In addition, the indazole NH donates a hydrogen bond to the carboxylate of Asp512. Notably, the aforementioned triad of key interactions plays an instrumental role in anchoring the SYK inhibitor in the SYK active site and is critical for maintaining a high kinase affinity. In addition to the aforementioned structural units, the chemical architecture of entospletinib comprises a terminal piperazine nitrogen that projects toward the solvent-exposed region of the kinase binding site. It is worth noting that this outward-projecting vector (piperazine nitrogen) does not participate in hinge interactions and was pinpointed as the chemically accessible exit vector (suitable attachment point) that can tolerate structural elaboration (linker installation) without significantly disrupting kinase recognition. On the basis of these pharmacophoric considerations, the entospletinib framework was selected as a suitable template for hybrid inhibitor design.

Though the aforementioned validation was convincing enough to propel us toward the construction of Entospletinib-based HDAC inhibitors, a lack of structural investigation on Entospletinib at position 6 conferred a scope to interrogate the impact of placement of electronically and sterically diverse aryl/heteroaryl groups on the SYK inhibitory activity. Unfortunately, the endeavor did not pay dividends as we could not identify a structural derivative endowed with more pronounced SYK inhibitory effects than Entospletinib (the results of this structural investigation are presented in Supporting Information—section 3, Figure 3S and Table 1S).

### 2.3 Design of a Hybrid Scaffold (Entospletinib–SAHA Hybrid) and Its Structural Refinement

With the clear-cut understanding of the impact of the C-6 occupier on the SYK inhibitory activity, a hybrid scaffold combining entospletinib and SAHA was designed at the outset (Figure 1A). This construct featured the appendage of the entospletinib fragment (surface recognition part) to the six-methylene unit linker bearing hydroxamic acid functionality. The **hybrid 1** was docked in the active site of SYK and HDAC isoforms (HDAC1, 2, 3, and 6) (Figures 1B and 5S, Supporting Information). Although the designed hybrid scaffold demonstrated a favorable binding interaction profile with the amino acid residues of HDAC isoforms (HDAC1, HDAC2, HDAC3, and HDAC6) as well as SYK, the potential for further structural optimization was distinctly evident. In the context of zinc-ion coordination, metal chelation was consistently observed across all HDAC isoforms. However, specific interaction features, such as the partial protrusion of the indazole ring beyond the catalytic cavity (HDAC1), lack of engagement of the imidazo­[1,2-*a*]­pyrazine ring with key amino acid residues (**HDAC1, HDAC2, HDAC3**, and **SYK**), and limited hydrogen-bonding interactions in multiple binding poses, underscore the need for structural refinement of the lead hybrid scaffold.

**Figure 1:**
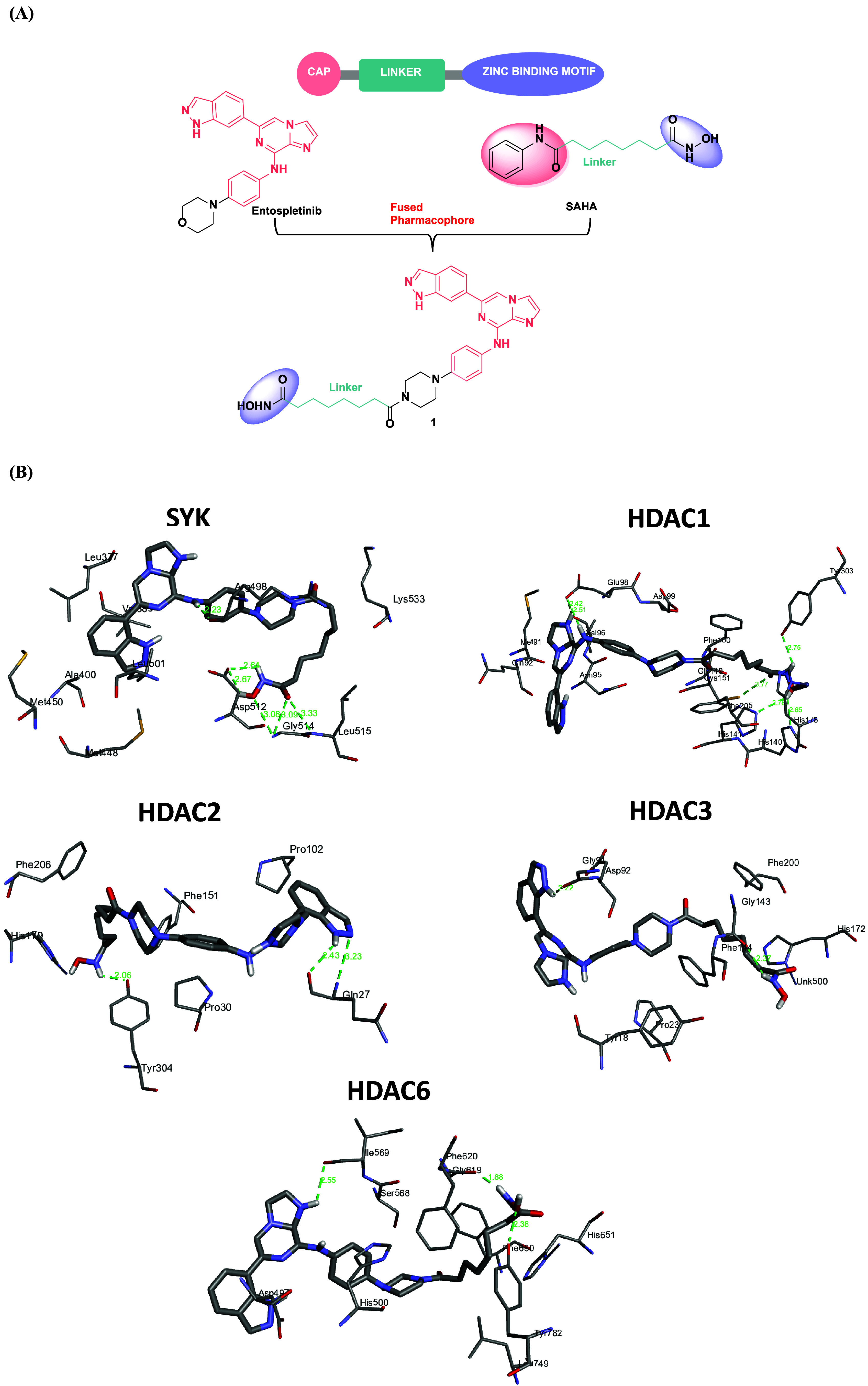
(A) Design strategy for the generation of the Entospletinib–SAHA hybrid (**1**). (B) Docking Analysis of **1** within the active site of **SYK, HDAC1, HDAC2, HDAC3, and HDAC6** (PDB IDs: 4PUZ, 5ICN, 6WBZ, 4A69, 5EDU, respectively, 3D-docked poses).

Accordingly, systematic structural modifications were conceptualized to fine-tune the conformational flexibility and improve spatial accommodation within the active site, including linker variation through methylene unit homologation or truncation and incorporation of chemically diverse linker motifs (e.g., benzyl and benzyl acrylamide groups) and scissoring of the piperazine ring, resulting in the design of compounds **2–18** (Figure 2).

**Figure 2:**
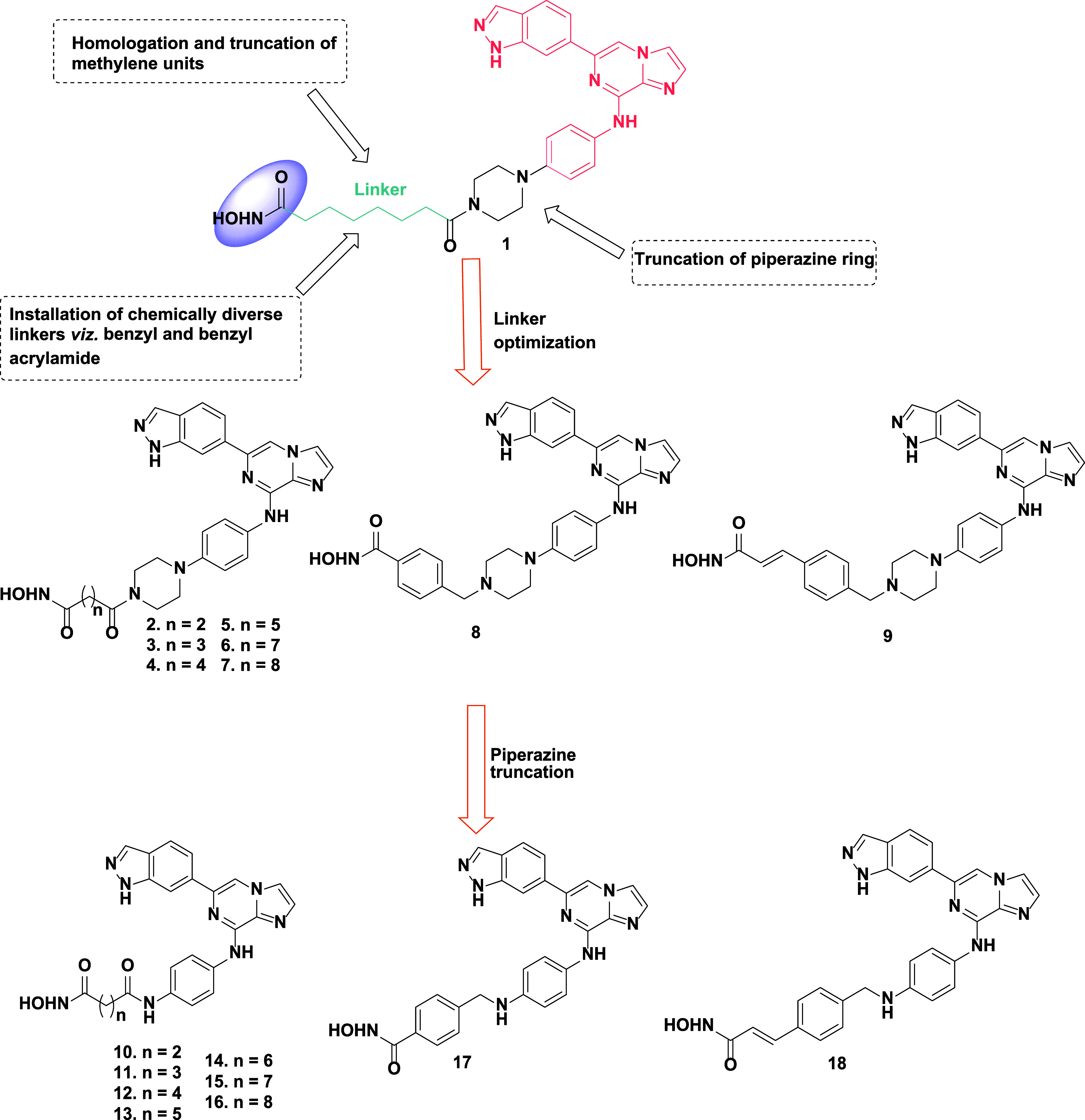
Fine-tuning of **1** and target structures.

### 2.4 Synthesis

The route to the synthesis of entospletinib derivatives **ED1-ED27** is depicted in Scheme 1. 6,8-Dibromoimidazo­[1,2-*a*]­pyrazine was appended with 4-morpholino-4-yl-phenylamine *via* nucleophilic substitution reaction using diisopropylamine as the base. The resulting intermediate **20** was then subjected to tetrakis­(triphenylphosphine)­palladium(0)-catalyzed Suzuki arylation with boronic acid (substituted phenyls and heteroaryl substrates). Gladly, the synthetic route established demonstrated significant flexibility in the context of applicability to chemically diverse boronic acids and enabled the formation of products (**ED1 to ED27**) in moderate to good yields.

**Scheme 1:**
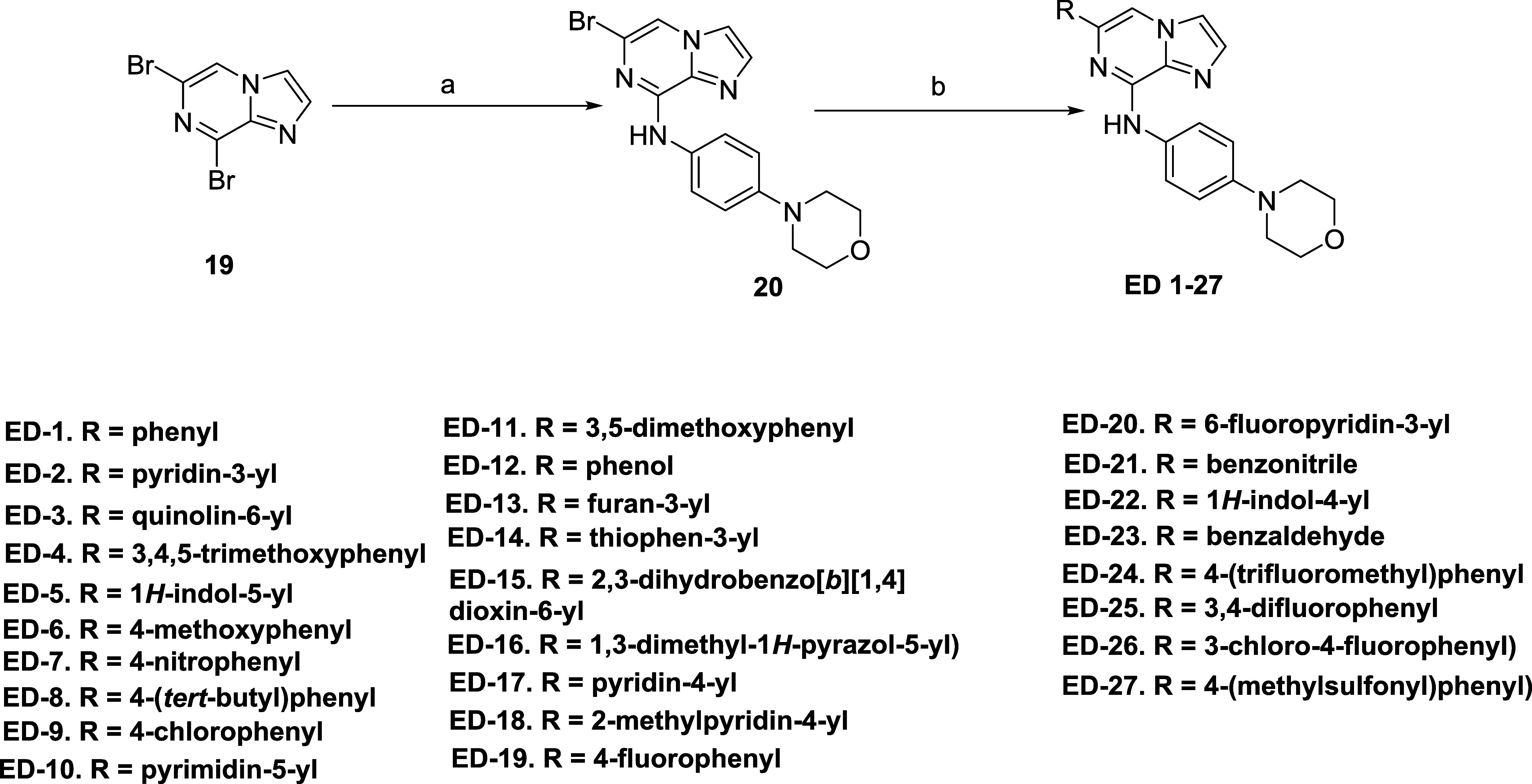
Reagents and Conditions^a^

a (a) 4-Morpholino-4-yl-phenylamine, DIPEA, isopropyl alcohol, 80 °C, 4 h; (b) various boronic acids, Na_2_CO_3_, Pd­(PPh_3_)_4_, dioxane:water (9:1), 100 °C, 3 h.

For the generation of adducts **ED28-ED-30**, the synthetic route illustrated in Scheme 2 was employed. The route commenced with the nucleophilic substitution reaction of dibromoimidazo­[1,2-*a*]­pyrazine with *tert*-butyl 4-(4-aminophenyl)­piperazine-1-carboxylate, aniline, and N-Boc piperazine to obtain intermediates **21–23**. Tetrakis­(triphenylphosphine)­palladium(0)-assisted Suzuki arylation of intermediate **21** and **23** with indazole-6-boronic acid, followed by subsequent TFA-assisted Boc deprotection, generated Entospletinib derivatives **ED28 and 30**. For the generation of the adduct **ED-29**, intermediate **22** was subjected to C–C bond formation with indazole-6-boronic acid, leveraging the same methodology as employed for **ED28** and **ED-30**.

**Scheme 2:**
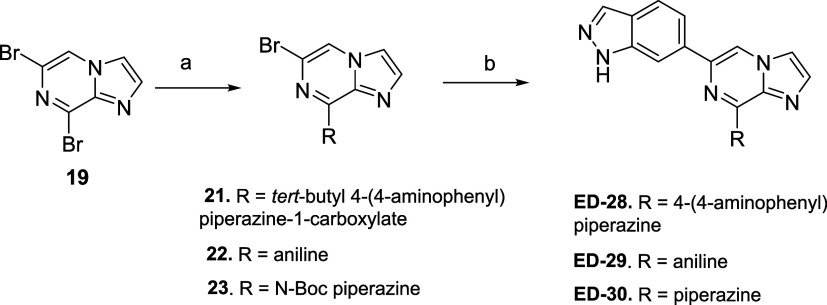
Reagents and Conditions^a^

a (a) Fo**r 21:** *tert*-Butyl 4-(4-Aminophenyl)­piperazine-1-carboxylate, isopropyl alcohol, DIPEA, 80 °C, 4 h; **For 22:** aniline; **For 23:** N-Boc piperazine, DIPEA, isopropyl alcohol, 80 °C, 4 h; (b) **For ED28** & **ED-30:** (i) indazole-6-boronic acid, Na_2_CO_3_, Pd­(PPh_3_)_4_, dioxane/water (9:1), 100 °C, 3 h; (ii) TFA, DCM, 0°C-rt, 4 h; **For ED-29:** indazole-6-boronic acid, Na_2_CO_3_, Pd­(PPh_3_)_4_, dioxane/water (9:1), 100 °C, 3 h.

The construction of target scaffolds **1–18** was accomplished through multistep synthetic routes illustrated in Schemes 3–6. Initially, methylene chain linker piperazine-containing chemical architectures **1–7** were furnished *via* a synthetic route depicted in Scheme 3. The route commenced with the appendage of *tert*-butyl 4-(4-aminophenyl)­piperazine-1-carboxylate with the 6,8-dibromoimidazo­[1,2-*a*]­pyrazine core (**19**) *via* base-catalyzed nucleophilic substitution reaction to obtain intermediate **21**. Organopalladium [(Pd­(PPh_3_)_4)_] catalyzed Suzuki arylation of the intermediate **21** with indazole-6-boronic acid generated an adduct, which was subsequently treated with trifluoroacetic acid to enable the deprotection of the Boc group. The resulting intermediate **ED28** was then amidated with alkoxy alkanoic acids of varied length (**n = 2–8**). Notably, alteration in the context of the number of methylene units in the linker part was attempted in pursuit of deciphering the impact of linker length variation on the cellular and enzymatic activity. Further, we attempted to employ the conventional methodology for the generation of hydroxamic acids that comprised a reaction sequence, *viz*., LiOH-assisted conversion of esters to carboxylic acids, amidation of the acids with NH_2_OTHP/NH_2_OBn, and debenzylation/deprotection (tetrahydropyranyl functionality). Unfortunately, our attempts were marred by poor yields and long reaction times that led us to explore a divergent pathway to directly convert the esters (**24–30**) to hydroxamic acids *via* treatment with NH_2_OH employing 1,8-diazabicyclo [5.4.0]­undecene-7-ene (DBU) as the non-nucleophilic base. Delightfully, the yields of all of the target hydroxamic acids (**1–7**) attained through the aforementioned methodology were acceptable.

**Scheme 3:**
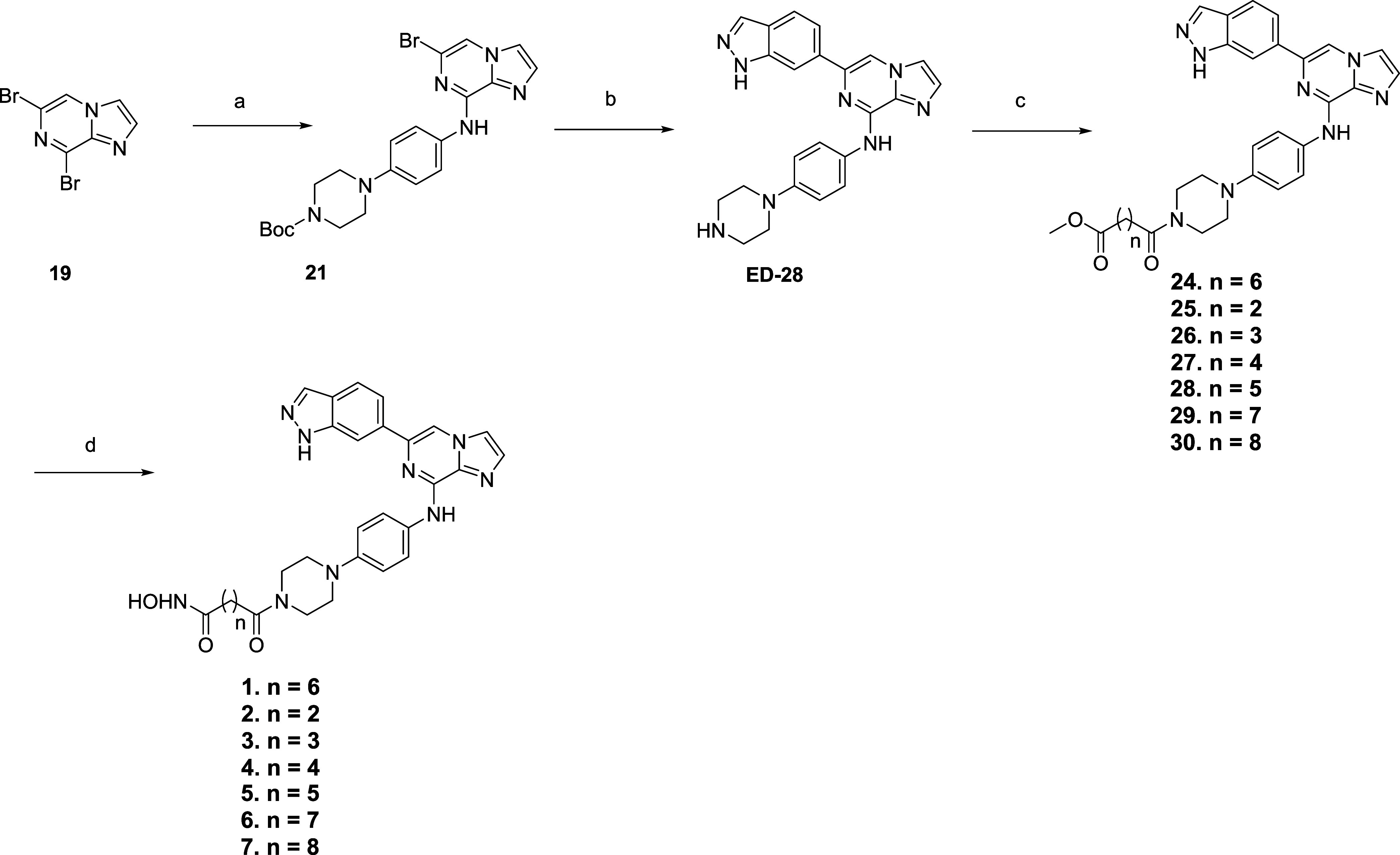
Reagents and Conditions^a^

a (a) *tert*-Butyl 4-(4-aminophenyl)­piperazine-1-carboxylate, DIPEA, isopropyl alcohol, 80 °C, 4 h; (b) (i) indazole-6-boronic acid, Na_2_CO_3_, Pd­(PPh_3_)_4_, dioxane/water (9:1), 100 °C, 3 h; (ii) TFA, DCM, 0 °C-rt, 4 h; (c) various alkoxy oxoalkanoic acids (*n* = 2–8), HATU, DIPEA, DMF, rt, 4 h; (d) NH_2_OH _(50%aq),_ DBU, MeOH, 0°C 2 h.

To gain a deeper insight into the linker-cytotoxicity relationship, installation of benzyl and benzyl acrylamide functionality as the linker component of the designed adducts was attempted *via* a reaction scheme depicted in Scheme 4. It is worth noting that the aforementioned strategy was spurred by the success of tubastatin, a selective HDAC6 inhibitor bearing a benzyl linker, and LBH589, a pan HDAC inhibitor comprising a benzyl acrylamide linker. The route began with the benzylation of the starting material **ED28** with ethyl 4-(bromomethyl)­benzoate using K_2_CO_3_ as the base to afford intermediate **31**. A parallel attempt was made to reductively aminate **ED28** with 4-formylcinnamic ester using NaCNBH_3_ as the reducing agent to obtain intermediate **32**. Lithium hydroxide-mediated de-esterification of intermediates **31** and **32**, followed by subsequent amidation of the generated carboxylic acids with NH_2_OTHP using EDC/HOBt-based coupling procedures and protic acid-assisted cleavage of tertrahydropyranyl functionality, culminated in the formation of designed structural templates **8** and **9** in acceptable yields.

**Scheme 4:**
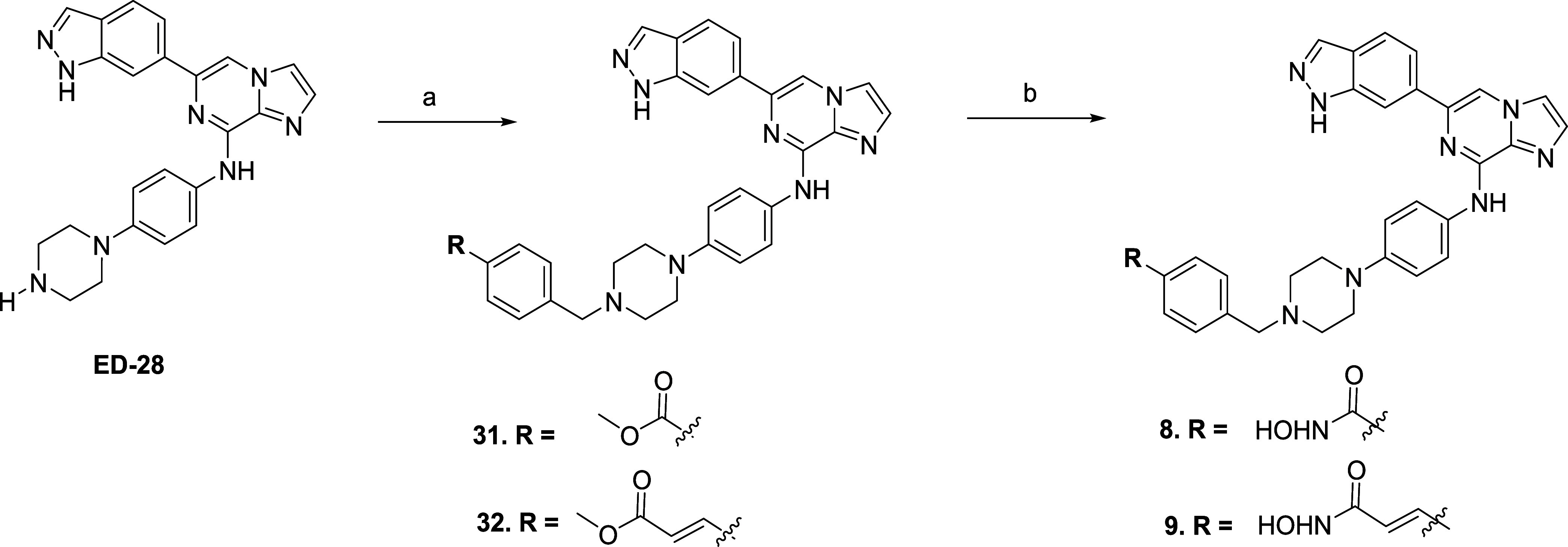
Reagents and Conditions^a^

a (a) (i) **For 31:** methyl 4-(bromomethyl)­benzoate, K_2_CO_3_, DMF, rt, 4 h; (ii) **For 32:** 4-formylcinnamic ester, NaCNBH_3,_ CH_3_COOH, C_2_H_5_OH, rt, 2 h; (b) (i) LiOH_(aq)_, dioxane, rt, 6 h; (ii) NH_2_OTHP, EDC·HCl, HOBt, DIPEA, DMF, rt, 4 h; (iii) TFA _(10%aq),_ MeOH, rt, 2 h.

Schemes 5 and 6 illustrate the multistep synthetic routes to piperazine scissored counterparts (compounds **10–18**) of compounds **1–9**. Notably, the aforementioned structural alteration was planned in an attempt to assess whether the piperazine truncation can confer conformational aptness to the resulting scaffolds to exert balanced modulation of SYK and HDAC isoforms. For the synthesis of scaffolds **10–16**, a similar strategy as outlined in Scheme 3 was employed, with the use of *tert*-butyl (4-aminophenyl)­carbamate in place of *tert*-butyl 4-(4-aminophenyl)­piperazine-1-carboxylate for the first step of the multistep synthetic route as the only point of difference (Scheme 5). For the synthesis of benzyl and benzyl acrylamide-based chemical architectures with the obliterated piperazine ring, *N*1-(6-(1H-indazol-6-yl)­imidazo­[1,2-*a*]­pyrazin-8-yl)­benzene-1,4-diamine was used as the starting material, and the same reaction sequence, as depicted in Scheme 4, was performed to obtain the target templates (**17** and **18**) in acceptable yields.

**Scheme 5:**
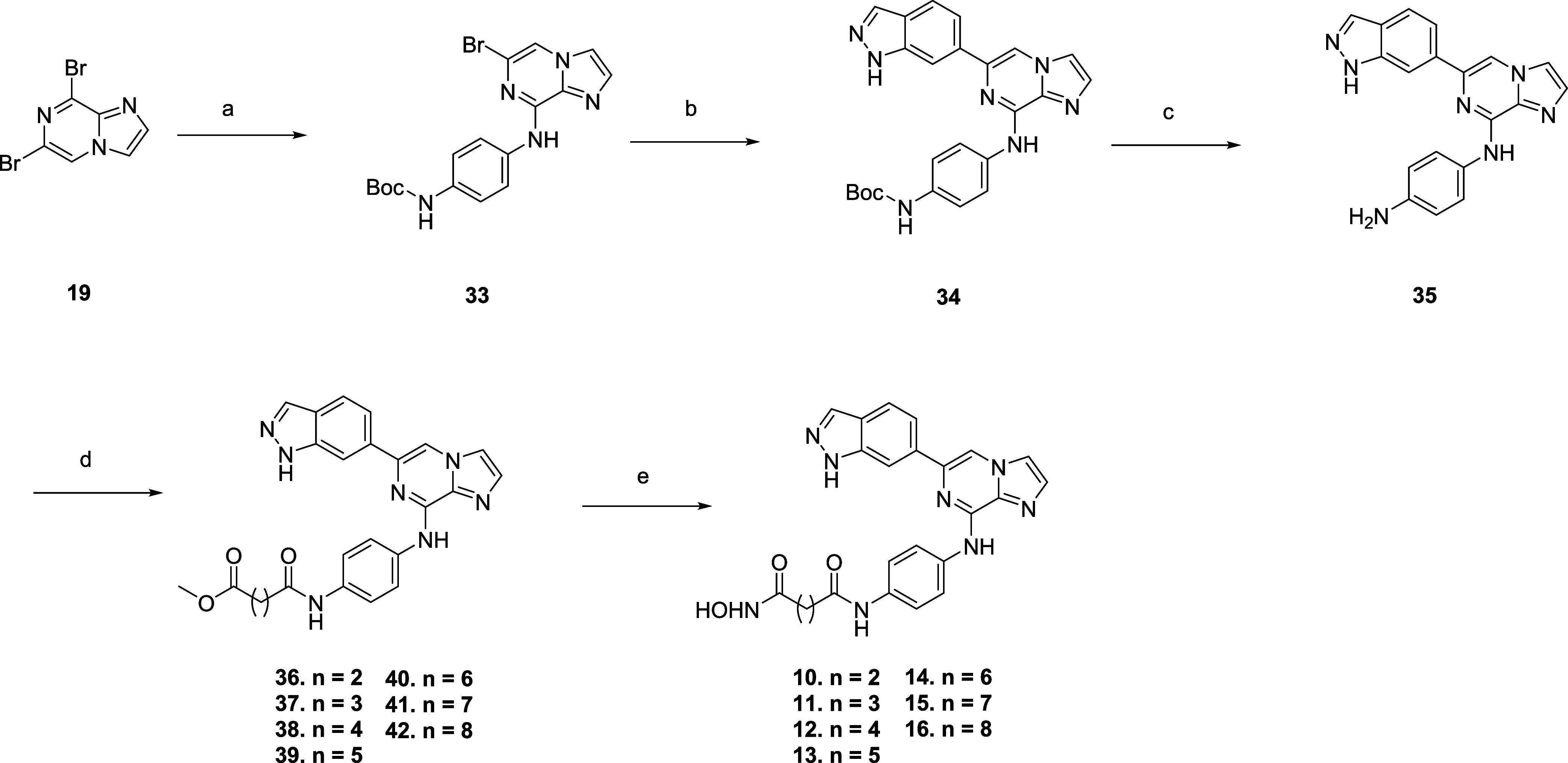
Reagents and Conditions^a^

a (a) *tert*-Butyl (4-aminophenyl)­carbamate, DIPEA, isopropyl alcohol, 80 °C, 4 h; (b) indazole-6-boronic acid, Na_2_CO_3_, Pd­(PPh_3_)_4_, dioxane, water (9:1), reflux, 3 h; (c) TFA, DCM, 0°C-rt, 4 h; (d) various alkoxy oxoalkanoic acids (*n* = 2–8), HATU, DIPEA, DMF, rt, 4 h; (e) NH_2_OH _(50%aq),_ DBU, MeOH, 0°C 2 h.

**Scheme 6:**
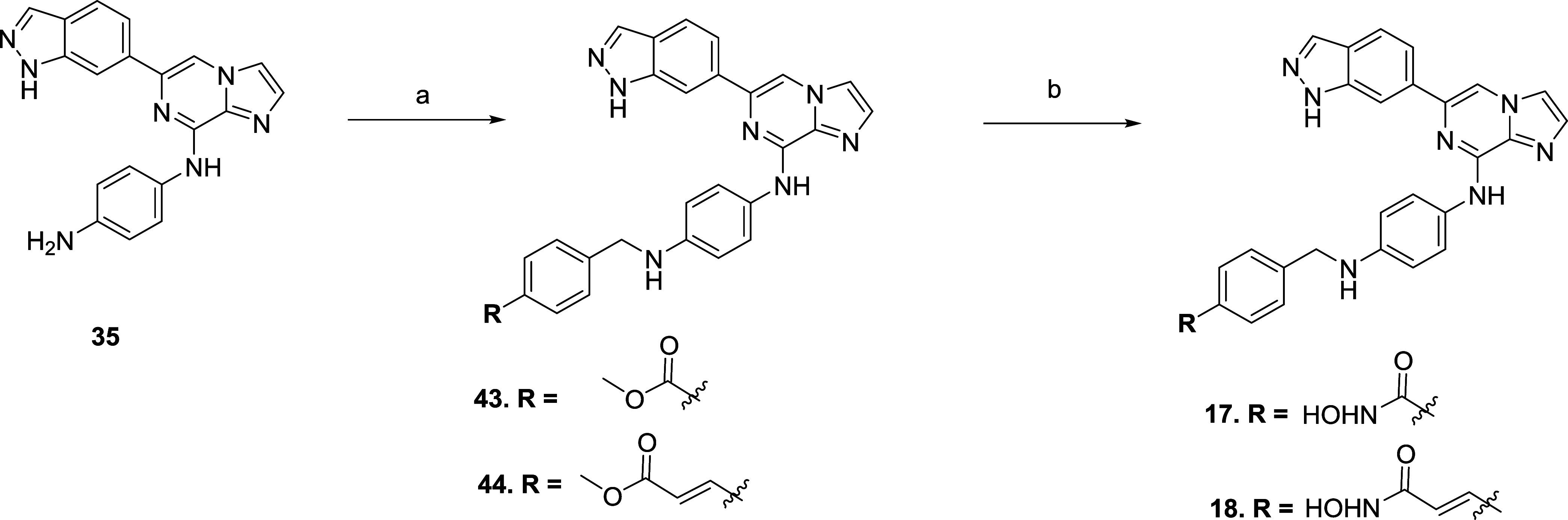
Reagents and Conditions^a^

a (a) (i) **For 43:** methyl 4-(bromomethyl)­benzoate, K_2_CO_3_, DMF, rt, 4 h; (ii) **For 44:** 4-formylcinnamic ester, NaCNBH_3,_ CH_3_COOH, C_2_H_5_OH, rt, 2h; (b) (i) LiOH_(aq)_, dioxane, rt, 6 h; (ii) NH_2_OTHP, EDC·HCl, HOBt, DIPEA, DMF, rt, 4 h; (iii) TFA _(10%aq),_ MeOH, rt, 2 h.

### 2.5 SYK Inhibitory Evaluation

The biological evaluation campaign for the synthesized compounds began with the assessment of the SYK inhibitory potential. This study was prioritized over the *in vitro* cytotoxicity and HDAC inhibitory studies to assess whether the generated scaffolds featuring the appendage of entospletinib core to the HDAC inhibitory components retain the SYK inhibitory activity. The assay was conducted by Reaction Biology Corporation (Malvern, Pennsylvania). Entospletinib was employed as the standard for the evaluation. Compounds were preincubated with kinase and substrate (and cofactors if needed) mixtures at room temperature, and then the reaction was initiated by the addition of radioisotopically labeled ATP (33P-γ-ATP). The outcome of the assay (Table 1) revealed that out of the **18** target adducts, **14** compounds, *viz*., **1–5** and **10–18** demonstrated substantial SYK inhibitory activity. Interestingly, the scaffolds featuring the piperazine connector **1–7** elicited a consistent trend in their SYK inhibitory profile as homologation of the methylene units (linker) led to downward trends [compare **2 (n = 2)** and **3 (n = 3); 3 (n = 3)** and **4 (n = 4); 4 (n = 4)** and **5 (n = 5); 5 (n = 5)** and **1 (n = 6); 1 (n = 6)** and **6 (n = 7);** and **6 (n = 7)** and **7 (n = 8)**]. Noteworthy to mention that, the extension of the methylene chain from *n* = 2 to *n* = 6 only led to a slight decline in activity, with compound**s 1** to **5** still eliciting SYK inhibition at subnanomolar concentrations. Intriguingly, further chain elongation from *n* = 6 to *n* = 7 and 8 [compare **1** with compound **6** (*n* = 7) and compound **7** (*n* = 8)] led to a dramatic loss in SYK inhibitory potential. Replacing the methylene chain linker with benzyl linker (compound **8**) and benzyl acrylamide linker (compound **9**) also displayed reduced inhibitory potential compared to the most potent piperazine-bearing scaffold (compound **2**, IC_50_ = 10.6 nM); however, the more pronounced loss in potency was observed with the latter in comparison to the former. Further, the profiling of piperazine-truncated adducts **10–18** was done, which led us to figure out the impact of piperazine scissoring on the SYK inhibitory activity. Gladly, the truncation of the piperazine ring proved to be favorable as compound **10–18** displayed an impressive SYK inhibitory profile with IC_50_ values ranging from subnanomolar concentrations to low nanomolar concentrations. Among them, compound **14** and compound **17** were found to be endowed with the most striking SYK abrogatory potential (IC_50_ value = 0.19 nM for both compounds) and were around **14**-fold more potent than Entospletinib (IC_50_ = 2.77 nM). Contrary to SYK inhibitory profiles of piperazine-bearing adducts **1–9**, the piperazine-truncated counterparts **10–18** manifested a more variable activity trend on homologation of the methylene chain. Specifically, extension of linker length from *n* = 2 to 4 led to decrease in activity, while further supplementation of the methylene units in the linker part was found to be beneficial as significant amplification in SYK inhibitory activity was witnessed for compound **13** (*n* = 5, IC_50_ = 1.41 nM), compound **14** (*n* = 6, IC_50_ = 0.19 nM), and compound **15** (*n* = 7, IC_50_ = 0.25 nM). Although a decline in SYK inhibitory concentration was observed on increasing the chain length from *n* = 6 to *n* = 8, the magnitude of the decrease in activity was marginal in piperazine obliterated counterparts (compound**s 15** and **16)** of compound**s 6** and **7**. Notably, compound **17**, the benzyl linker-based compound, displayed tantamount SYK inhibitory potency to compound **14**. Also, the insertion of benzyl acrylamide linker was well tolerated as compound **18** elicited significant SYK inhibitory activity at a single-digit concentration (IC_50_ = 1.48 nM); however, it was inferior to compound **17** as a SYK inhibitor. Cumulatively, the results of this study led to the identification of six compounds, *viz*., **13–18** endowed with more pronounced SYK inhibitory activity than Entospletinib.

**Table 1:** SYK Inhibitory Activity of Compounds **1–18**

compound	SYK inhibition (IC_50,_ nM)^a^	compound SYK inhibition (IC_50_, nM)^a^	
1	22	11	7.64
2	10.6	12	20.8
3	18	13	1.41
4	19	**14**	**0.19**
5	19.7	15	0.25
6	135	16	0.53
7	719	**17**	**0.19**
8	97.7	18	1.48
9	366	**Entospletinib**	2.77
**10**	**5.54**	**Staurosporine**	0.25

a The assay was conducted by the Reaction Biology Corporation, Malvern, Pennsylvania. Compounds were dissolved in DMSO and tested in 10-dose IC_50_ mode with 3-fold serial dilution starting at 10 μM.

### 2.6 *In Vitro* Cytotoxicity Studies

The synthesized adducts **1–18** were evaluated for the *in vitro* cytotoxicity against human acute myeloid leukemia cell lines (**MV4–11 cells harboring FLT3-ITD mutation**) using the MTT assay. SAHA and Entospletinib were used for the comparison of cell growth inhibitory effects of compounds **1–18** (Table 2). The assessment endeavor commenced with the evaluation of the hybrid scaffold (**1**) that demonstrated moderate cytotoxicity against MV4–11 cell lines. Notably, structural engineering attempts on 1, *viz*., dehomologation, homologation, and linker switch from methylene chain to benzyl/benzyl acrylamide linker, did not pay dividends in the context of potentiation of cell growth inhibitory effects. Indeed, all of the piperazine appended scaffolds, **1** and **2–9**, displayed weak cytotoxic potential in comparison to SAHA (FDA-approved HDAC inhibitor); however, **1–6** demonstrated superior antitumor effects against MV4–11 cells in comparison to Entospletinib. Attempts to correlate the *in vitro* cytotoxic activity profile of compounds **1–9** with the SYK inhibitory assessment results indicated that compounds endowed with weak SYK inhibitory activity (compound**s 6–9**) also manifested weak cell growth inhibitory effects against the MV4–11 cells with IC_50_ values >3 μM. The aforementioned observation indicated that SYK inhibition is an underlying mechanism for the *in vitro* antiproliferative effects of the furnished chemical architectures. Further structural alteration attempts, *viz*., piperazine scissoring, proved to be beneficial as adducts **10–18** exhibited an improved cytotoxic profile compared to their piperazine appended counterparts (**1–9**). It is noteworthy to mention that, barring compound**s 10** and **11**, all of the other piperazine-truncated scaffolds (**12–18**) exerted more pronounced cell growth inhibition than the standards employed (**SAHA and Entospletinib**). Correlation of SYK inhibitory activity of adducts **10–18** (Table 1) with the *in vitro* cytotoxicity results led us to deduce that the enhancement in the SYK inhibitory effects was accompanied by the increase in the cell growth inhibitory effects of piperazine obliterated compounds. Impressively, compound **14–17**, exerting SYK inhibition at subnanomolar concentrations, elicited the most substantial anticancer activity, with IC_50_ values of **0.11, 0.22, 0.31**, and **0.18 μM**. On the basis of the aforementioned notions, the impact of linker variation and piperazine truncation was clearly evident on the cytotoxicity. Also, it was observed that the Entospletinib core within the structural template of target scaffolds exhibited higher flexibility toward the inclusion of chemically diverse linkers. For instance, compound **14** bearing a six-methylene chain, as well as compound **17** featuring the tetheration of Entospletinib core to hydroxamic acids *via* a benzyl linker, demonstrated remarkable *in vitro* anti-AML effects and were nearly equipotent in the cell growth inhibitory effects. Overall, compound **14** was found to be the most potent cell growth inhibitor of MV4–11 cells.

**Table 2:** *In Vitro* Cytotoxicity Studies of the Synthesized Compounds against MV4-11 Cells

compound	MV4–11 cells (IC_50,_ μM)^a^	compound	MV4–11 cells (IC_50,_ μM)^a^
1	1.562 ± 0.166	11	0.8934 ± 0.031
2	1.064 ± 0.166	12	0.4595 ± 0.023
3	1.983 ± 0.106	13	0.4024 ± 0.034
4	1.117 ± 0.064	14	0.115 ± 0.004
5	1.813 ± 0.263	15	0.2238 ± 0.015
6	3.233 ± 0.285	16	0.3122 ± 0.014
7	>5	17	0.1825 ± 0.013
8	3.580 ± 0.315	18	0.516 ± 0.039
9	4.629 ± 0.056	SAHA	0.928 ± 0.134
10	0.9263 ± 0.066	Entospletinib	2.48 ± 0.938

a SD = standard deviation. All experiments were independently performed at least three times (*n* = 3) to determine the mean and SD.

### 2.7 Quantitative Complexity Management (QCM)-Artificial Intuition Technology (4th Generation of AI)-Based Validation

The *in vitro* cytotoxicity study indicated that removal of the piperazine ring from the structural template of **hybrid** compound **1** resulted in improved antiproliferative activity, as the corresponding piperazine-truncated derivatives (**10–18**) were consistently more potent than their piperazine-containing counterparts (**1–9**) in MV4–11 cells. Because such activity differences can arise from multiple factors (including target affinity, physicochemical properties, permeability, and conformational behavior), we sought a complementary structural-dynamics-based interpretation of this SAR trend using the OPTIMUS platform (Ontonix, Italy), previously evaluated at the University of Pavia, Italy. OPTIMUS employs a Quantitative Complexity Management (QCM) framework that analyzes intrinsic molecular organization through atomic participation factor (APF) descriptors derived from molecular dynamics simulations. Importantly, this approach does not predict binding affinity or potency; rather, it provides a dynamics-based descriptor that may assist in identifying regions of the molecule that are structurally influential. It is important to mention that such descriptors should only be interpreted as hypothesis-supporting rather than mechanistically deterministic indicators of activity.^57,58^

Representative piperazine-containing hybrids (**1, 8, 9**) and their truncated analogues (**14, 17, 18**) were subjected to molecular dynamics simulations followed by QCM processing to generate complexity profiles (Figure 3). Atoms exhibiting APF values ≥3% were classified as high-participation contributors, reflecting regions that strongly influence organized molecular motion. Notably, compound**s 14, 17**, and **18** displayed a substantially greater number of such contributors (14, 11, and 8 atoms, respectively), accounting for more than 40% of the molecular participation contribution compared with their counterparts 1, 8, and 9 (each containing four atoms ≥ 3%), which showed lower cumulative contributions (approximately 16–30%). In particular, the APF spectrum of compound **14** revealed a cluster of atoms (C2–C8 together with N1, O2, and O3) exhibiting similar and relatively elevated participation factors and a broader distribution of dynamically influential regions across the scaffold, suggesting coordinated rather than dispersed internal motion.

**Figure 3:**
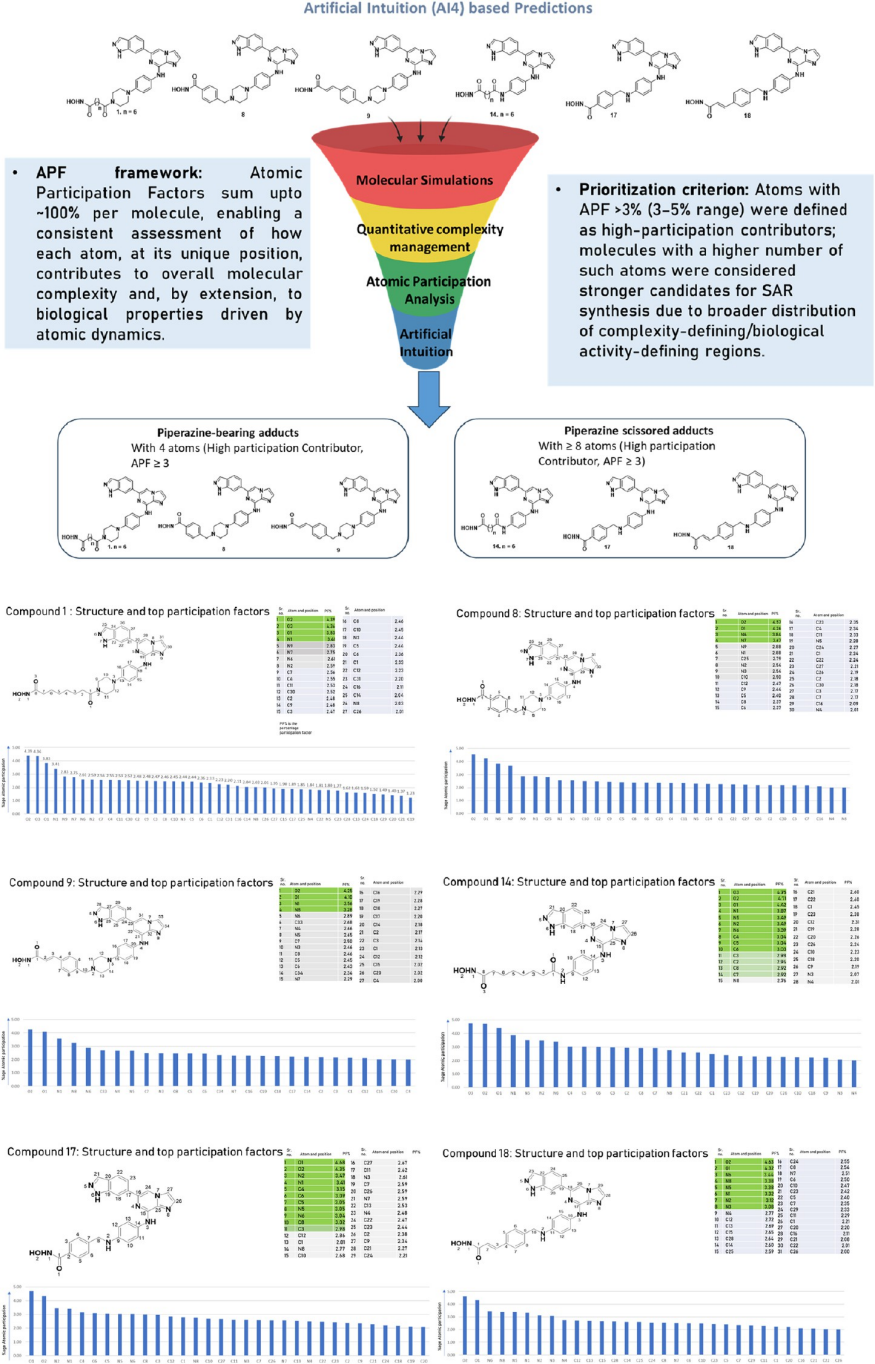
Quantitative Complexity Management (QCM) (artificial intuition–4th generation AI technology)-based prediction-APF spectra of designed compound**s 1, 8, 9, 14, 17, and 18**.

Overall, these observations are in line with the trends of the SAR study and indicate that the removal of the piperazine functionality may lead to a more organized distribution of internal molecular motion that might be correlated to the improvement in cellular activity. It is worth noting that biological potency is influenced by multiple factors beyond conformational dynamics, and QCM analysis here has just been presented as a complementary structural interpretation rather than a definitive mechanistic explanation. Thus, the outcome supports the piperazine truncation strategy as a plausible structural optimization approach while acknowledging that permeability, target affinity, and off-target interactions may also contribute to the observed activity differences.

### 2.8 HDAC Isoform Inhibition Assay

Compound **14** was evaluated for HDAC isoform inhibitory effects against the HDAC isoforms (HDAC1–10) (Table 3). The assay was performed by Reaction Biology Corporation, USA. The HDACs 1, 2, 3, and 6 activities were measured using a fluorogenic peptide substrate derived from p53 residues 379–382 (RHKK­(Ac)­AMC). HDACs 4, 5, 7, and 9 activities were measured using a fluorogenic substrate (Trifluoroacetyl Lysine). HDAC8 activity was evaluated using a fluorogenic peptide substrate derived from p53 residues 379–382 (RHK­(Ac)­K­(Ac)­AMC). HDAC10 activity was assessed using the fluorogenic substrate Ac-spermidine-AMC. Delightfully, compound **14** manifested remarkable potential to inhibit HDAC1, 2, 3, and 6 isoforms at low nanomolar concentrations with IC_50_ values of 3.39, 4.26, 7.55, and 0.66 nM, respectively. Also, the selectivity indices for compound **14** were determined, which indicated striking selectivity toward the HDAC6 isoform. Notably, compound **14** demonstrated a selectivity index of >20000 toward HDAC6 over HDAC4, 5, and 7. Also, compound **14** was found to be 6969 and 1319-fold more selective toward HDAC6 in comparison to HDAC8 and HDAC10. Important to mention that HDAC inhibitor **14** exhibited preferentiality toward HDAC6 isoform in comparison to the other HDAC isoforms (HDAC1, 2, and 3) that also demonstrated sensitivity to its exposure. Overall, compound **14** was deduced to be a class I HDAC (HDAC1, HDAC2, and HDAC3) inhibitor as well as a class II B HDAC (HDAC6) inhibitor.

**Table 3:** HDAC Inhibition Activity of the Compound **14**

	(IC_50_, nM)^a^				
target	14	trichostatin	TMP269	Quisinostat	SF^b^ HDAC6/HDAC isoforms (1–5, 7–10)^c^
**HDAC1**	**3.39**	1.56	ND	ND	5.13
**HDAC2**	**4.26**	5.89	ND	ND	6.45
**HDAC3**	**7.55**	2.42	ND	ND	11.43
**HDAC4**	>10,000	ND	108	ND	>20,000
**HDAC5**	>10,000	ND	168	ND	>20,000
**HDAC6**	**0.66**	1.27	ND	ND	---
**HDAC7**	>10,000	ND	39.4	ND	>20,000
**HDAC8**	4600	430	ND	ND	6969
**HDAC9**	---	ND	13.6	ND	---
**HDAC10**	871	ND	ND	7.11	1319

a Empty cells indicate no inhibition observed or compound activity that could not be fitted to an IC_50_ curve.

b This assay was conducted by the Reaction Biology Corporation, Malvern, Pennsylvania. Compounds were dissolved in DMSO and tested in a 10-dose IC_50_ mode with 3-fold serial dilution starting at 10 mM.

c Selectivity factor = selectivity factor for HDAC6 over HDAC isoforms (1–5, 7–9) (SF6/1 = IC_50_ (HDAC1)/IC_50_ (HDAC6)).

### 2.9 Western Blot Analysis (p-SYK Levels), Molecular Modeling Study (SYK), and Kinase Selectivity Assay

The outcomes shown in Tables 1 and 3 ascertain the potential of compound **14** as a dual inhibitor of SYK and HDAC isoforms. Continued attempts to elucidate the mechanistic insights of compound **14** as an antileukemic agent prompted us to perform Western blot analysis to assess the modulatory effect of compound **14** treatment on the expression levels of p-SYK. Notably, p-SYK is the enzymatically active form of SYK reported to initiate the downstream signaling cascades, *viz*., PLCγ2, BLNK, and ERK. As such, the activation of SYK occurs through site-specific tyrosine phosphorylation.^59^ The outcome of the Western blot analysis indicates that compound **14** markedly downregulated the expression levels of p-SYK, indicating the potential of compound **14** as a SYK inhibitor. The p-SYK/total SYK ratio, which is considered to be the quantitative measure of SYK activation, is shown in Figure 4A, indicating a significant reduction in this ratio, which suggests that the compound effectively suppresses SYK activation. Notably, the downregulatory effects of compound **14** on p-SYK levels were more pronounced than those of Entospletinib at equivalent concentrations.

**Figure 4:**
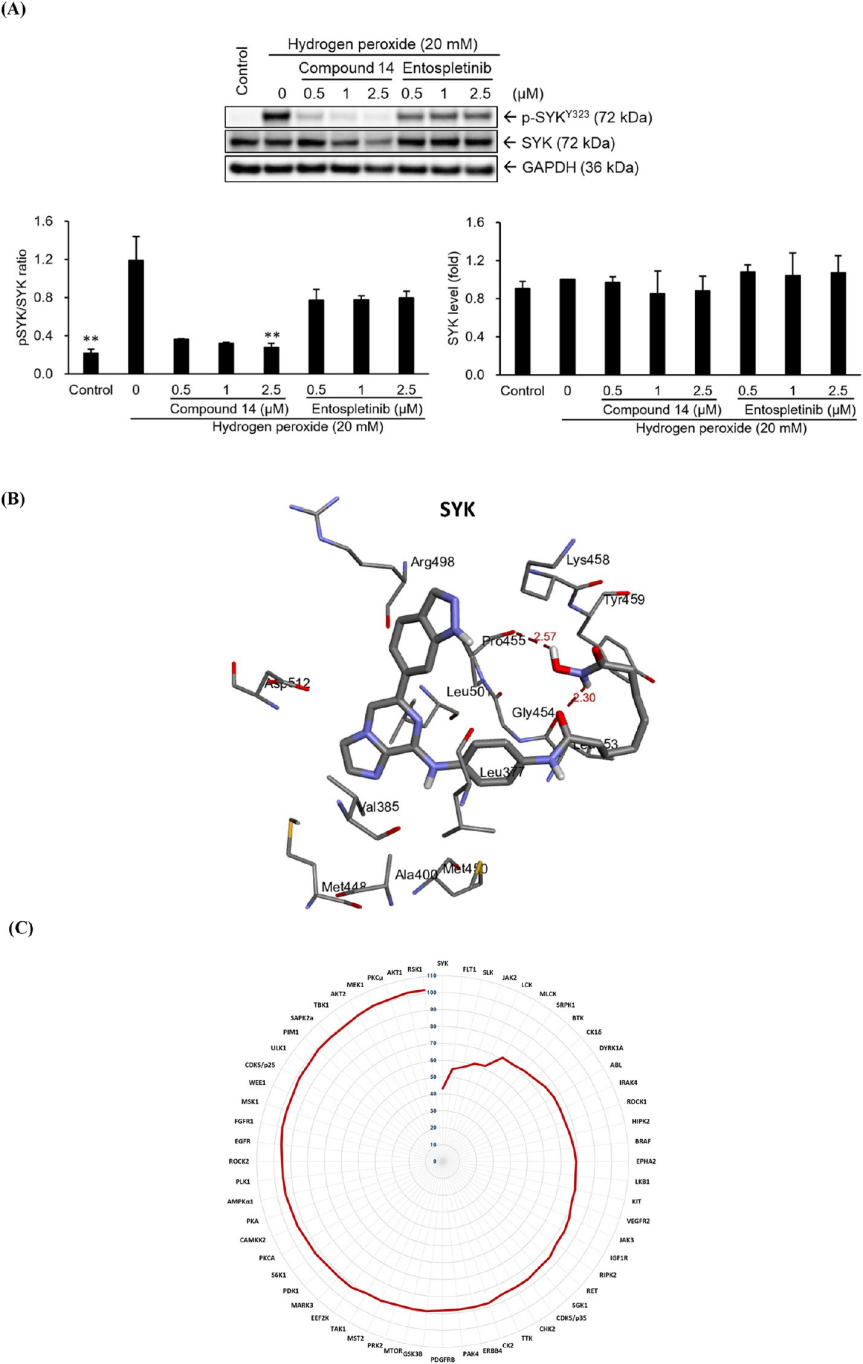
(A) MV4–11 cells seeded on a 6-well plate (1.5 × 10^6^ cells/well) were treated with various concentrations (0.5, 1, and 2.5 μM) of either compound **14** or Entospletinib for 24 h and then coincubated with 20 mM hydrogen peroxide for a further 30 min. Afterward, the cells were harvested to examine the expression levels of specific proteins using Western blot analysis. ** *p* < 0.01 compared to the group treated with 20 mM hydrogen peroxide alone. (B) 3D-docked pose of compound **14** showing hydrogen bond interactions, PDB ID (4PUZ). (C) Kinase selectivity analysis of the compound **14** against a panel of 60 kinases.

Further molecular modeling studies were performed, and compound **14** was docked in the active site of SYK (**PDB ID:** 4PUZ) to predict the binding interactions. The docking score (binding free energy, kcal/mol) as well as residues involved in hydrogen bonding and hydrophobic interactions are presented in Table 2S, and the docking interactions have been depicted in Figures 4B and 6S. Notably, the spatial accommodation of compound **14** within the catalytic site of SYK revealed that the compound is well enclosed within the hydrophobic cavity. The interaction analysis showed hydrogen bond interactions between the “carbonyl group” of Leu453 and “NH group” (**hydroxamic acid**) of compound **14** (bond distance: 2.30 Å), and “carbonyl group” of Pro455 and ‘OH group’ (**hydroxamic acid**) of compound **14** (bond distance: 2.57 Å). It is noteworthy to mention that these polar interactions are imperative to anchor the compound near the hinge region of the SYK binding domain. Also, a dense network of pi-interactions and hydrophobic interactions was observed that contributed to binding stabilization. Specifically, compound **14** was found to be involved in interactions with Leu453 (Amide-Pi-Stacked), Leu377, Pro455, & Leu501 (Pi-Sigma), Val385, Ala400, Pro455, Lys458, Arg498, & Leu501 (Pi-Alkyl), Met448, and Met450 (Pi-Sulfur) in the active site of the SYK protein. It is important to mention that Pi-sulfur interactions enhance the lipophilic compatibility of the ligand within the nonpolar region of the binding site. Also, compound **14** (aromatic moiety and aliphatic chain) interacted with multiple hydrophobic residues (Figure 6S, Supporting Information). Overall, compound **14** manifested favorable affinity and stability within the SYK binding cavity and formed an intricate network of stabilizing interactions *via* engagement with polar as well as nonpolar residues.

To assess the selectivity of compound **14** toward SYK, the compound was assayed against 60 kinases by the Kinase Enzymatic Radiometric [Km ATP] KinaseProfiler LeadHunter Assay of Eurofins-Cerep SA (Celle-Lévescault, France). Quantification of the degree of inhibition by compound **14** was done by the determination of residual kinase activity. Resultantly, SYK was found to be the most sensitive target in the kinase panel, indicating a SYK-biased kinome inhibition profile of compound **14**. Notably, compound **14** treatments also impacted the residual activity of FLT1, SLK, and JAK2 and LCK; however, limited off-target engagement was observed for the remainder of the panel (Figure 4C).

### 2.10 Western Blot Analysis (Expression Levels of Biomarkers Associated with Intracellular HDAC Inhibition) and Molecular Modeling Study (HDAC Isoforms)

Compound **14** was tested for its modulatory potential toward the expression levels of biomarkers associated with intracellular HDAC inhibition. Specifically, the regulatory effect of test compounds on acetylation levels of α-tubulin and histone H3 (biomarkers associated with HDAC inhibition) in MV4–11 cells was assessed. Encouragingly, compound **14** demonstrated significant upregulation of the acetylation levels on the ninth lysine of histone H3 (Ac–H3K9) in MV4–11 cells. Also, an upregulatory effect of compound **14** was evidenced on Acetyl α-tubulin. It is well established that histone H3 hyperacetylation is a consequence of class 1 HDAC inhibition, while acetyl tubulin upregulation results from HDAC6 inhibition.^60^ These findings, coupled with the results depicted in Table 3, confirm that compound **14** harbors an inhibitory potential toward class 1 HDACs (HDAC1, HDAC2, and HDAC3) as well as class II B HDAC (HDAC6), as indicated by the upregulated H3 acK9 and α-Tubulin acK40 levels (signatory feature of class I HDAC and HDAC6 inhibition) (Figure 5). Correlation of results presented in Tables 1–3 **and** Figures 4–5 indicates that the striking anti-AML effects of compound **14** stem from dual inhibition of SYK and HDAC.

**Figure 5:**
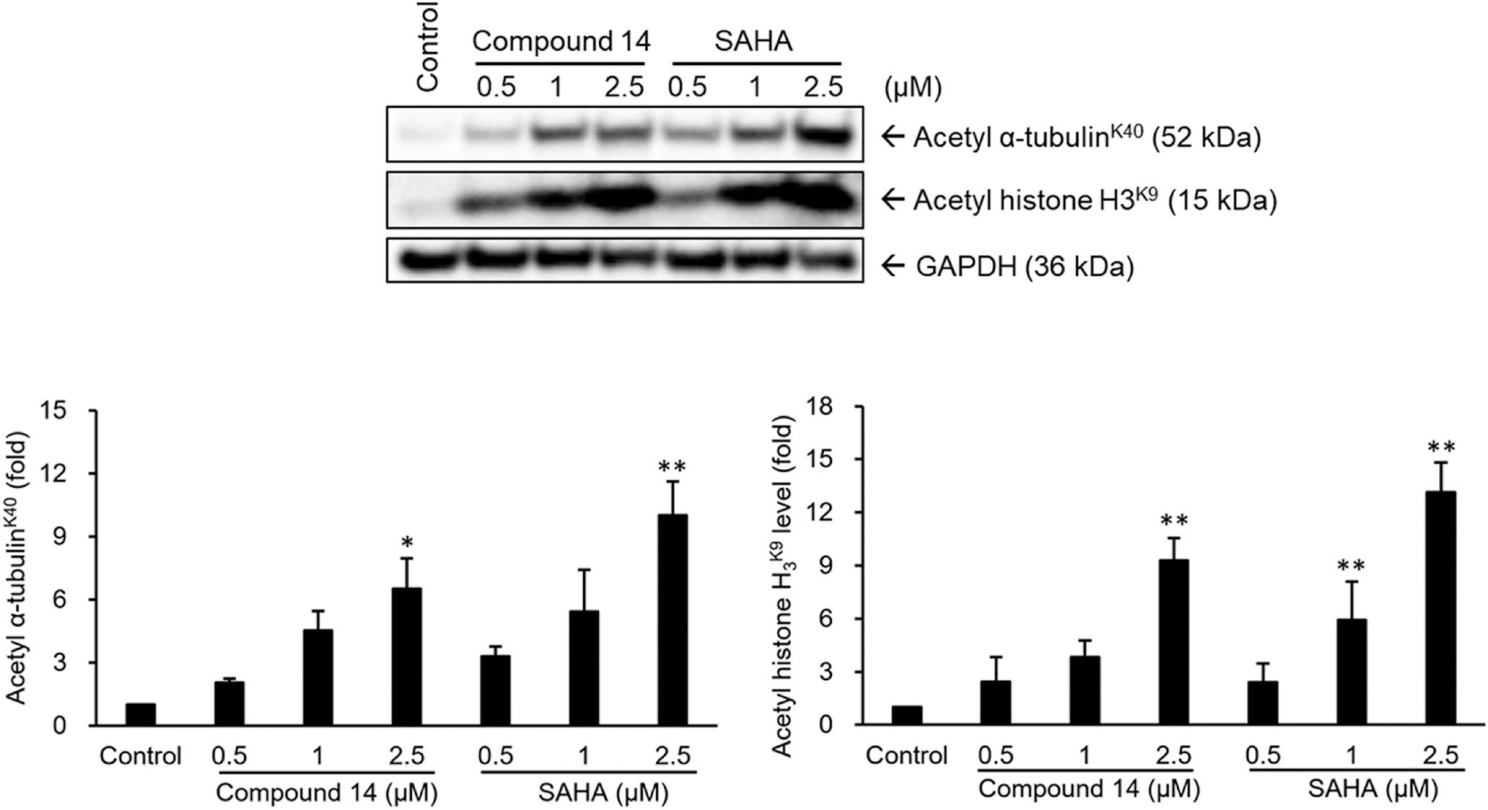
MV4–11 cells seeded on a 6-well plate (1.5 × 10^6^ cells/well) were treated with various concentrations (0.5, 1, and 2.5 μM) of either compound **14** or SAHA for 24 h. After that, the cells were harvested to examine the expression levels of specific proteins using Western blotting.

Compound **14** was further docked with HDAC1, HDAC2, HDAC3, and HDAC6 (PDB IDs: 5ICN, 6WBZ, 4A69, and 5EDU, respectively) to predict the binding interactions of this compound with these proteins. The docking score (binding free energy, kcal/mol) as well as residues involved in hydrogen bonding and hydrophobic interactions are presented in Table 3S.

The docking interactions of the compound **14** with active site residues of HDAC1 (PDB ID: 5ICN) are shown in Figure 6 (3D-docked poses) and Figure 7S (Orientation and 2D docked pose of compound **14**, Supporting Information). A well buried binding pose of compound **14** with a network of hydrophobic interactions, hydrogen-bonding interactions, coupled with chelation with the zinc ion, was observed. Docking analysis showed hydrogen bond interactions between the backbone “carbonyl group” Arg93 and “NH group” of benzimidazole ring of compound **14** (bond distance: of 3.16 Å), “NH group” of Asn95 and “N group” of purine ring of compound **14** (bond distance: 3.15 Å), “NH group” of Gly145 and “carbonyl group” of compound **14** (bond distance: 2.84 Å), and “OH group” of Tyr303 residue and “OH” & “carbonyl” groups of compound **14** (bond distance: 2.74, and 3.10 Å, respectively). The aforementioned indicates a stabilized bifurcated hydrogen bond network at the mouth of the tunnel. Stacking of the aromatic ring of compound **14** into a hydrophobic subpocket was observed, which led to Pi-Alkyl interactions with Phe150, His178, and Phe205. Also, a Pi-Anion interaction with Glu186 anchored the aromatic system. It is important to mention that the coordination of compound **14** with Zn401 (chelation) was observed. Collectively, the docking analysis indicated an impressive accommodative pattern of the compound in the active site of the HDAC1 protein.

**Figure 6:**
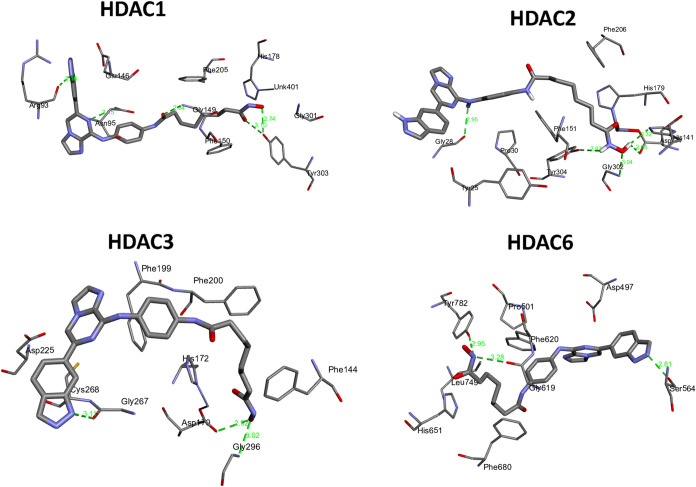
Interaction analysis of compound **14** with the amino acid residues of HDAC isoforms (docking study) – 3D-docked pose of compound **14**.

Docking analysis of the compound **14** within the active site of HDAC2 (PDB ID: 6WBZ) [Figure 6 (3D-docked poses) and Figure 7S (Orientation and 2D docked pose of compound **14**, Supporting Information)] revealed that the compound was well accommodated within the enzyme’s active site tunnel and was engaged in a network of hydrophobic interactions, hydrogen bond interactions, and metal coordination. Specifically, hydrogen bond interactions between “carbonyl group” Gly28 and “NH group” of compound **14** (bond distance: 3.40 Å), “ring N” of His141 and “OH group” of compound 14 (bond distance: 3.17 Å), “carbonyl group” of Asp177 and “OH group” of compound **14** (bond distance: 3.04, Å), and “NH group” of Gly302 and “OH group” of compound **14** (bond distance: 3.04 Å) were observed. Also, hydrophobic interactions with Pro30, Phe151, and His179 (Pi-Alkyl), Phe206 (Pi-Sigma), and metal interaction with Zn401 in the active site of the HDAC2 protein were manifested by compound **14.** Overall, the docking study indicated that compound **14** spans the hydrophobic tunnel as well as the zinc-binding region of HDAC2 and mimics the binding mode of classical HDAC inhibitors.

Docking analysis of compound **14** within the active site of HDAC3 protein (PDB ID: 4A69) [Figure 6 (3D-docked poses) and Figure 7S (Orientation and 2D docked pose of compound **14**, Supporting Information)] revealed that compound **14** is positioned deep inside the hydrophobic tunnel and occupies the adjacent lipophilic subpockets as well as the zinc-binding domain of HDAC3. Multiple stabilizing hydrogen bond interactions between “carbonyl group” Asp170 and “OH group” of compound **14** (bond distance: 2.92 Å), “carbonyl group” of Gly267 and “NH group” of benzimidazole ring of compound **14** (bond distance: 3.02 Å), and “NH group” of Gly296 and “OH group” of compound **14** (bond distance: 3.02 Å) were observed, contributing to the stability of the ligand within the catalytic groove. Also, hydrophobic interactions with Phe144, Phe200, and Cys268 (Pi-Alkyl), His172 (Pi-Sigma), Phe199 (Pi–Pi T-shaped), and metal interaction with Zn500 (hallmark of HDAC inhibitory binding) in the active site of the HDAC3 protein were observed. On the whole, compound **14** manifests a combination of hydrogen bond stabilization, hydrophobic interactions, and metal coordination. The aforementioned interactions effectively anchor compound **14** within the active site of HDAC3. Moreover, Pi-interactions through multiple aromatic residues enhance the structural complementarity.

Docking analysis of compound **14** (HDAC6-PDB ID: 5EDU) [Figure 6 (3D-docked poses) and Figure 7S (Orientation and 2D docked pose of compound **14**, Supporting Information)] revealed that compound **14** was engaged in multiple hydrogen bonds and hydrophobic and metal coordination interactions within the active site of HDAC6. Specifically, hydrogen-bonding interaction was observed between the benzimidazole nitrogen of compound **14** and the side chain hydroxyl group of Ser564 (bond distance: 2.81 Å) Additionaly, hydrogen-bonding interactions were also observed between “carbonyl group” of Gly619 and “NH group” of compound **14** (bond distance: 3.28 Å), and “OH group” of Tyr782 and “NH group” of compound **14** (bond distance: 2.95 Å). The aforementioned hydrogen-bonding interactions played a key role in anchoring the compound **14** within the active site of HDAC6 *via* a triad of polar interactions. Also, hydrophobic interactions with Pro501, Phe620, His651, & Phe680 (Pi-Alkyl), Asp497 (Pi-Anion), and Leu749 (Alkyl) were observed, which contributed to the hydrophobic stabilization of **14** along the tunnel. Notably, Pi-electron complementarity was enhanced *via* Pi–Pi T-shaped stacking interactions between the aromatic ring of **14** and Phe620 (Pi–Pi T-shaped). Moreover, Pi–anion interaction between the aromatic ring of **14** and Asp497 provided an electrostatic component to the binding affinity. Metal interaction with Zn901 in the active site of the HDAC6 protein was also observed, which ascertained the zinc chelating ability of compound **14**. Overall, the docking study revealed that compound **14** adopted an optimal orientation and occupied the extended catalytic tunnel of HDAC6, thereby engaging in hydrogen bonding near the rim residues, hydrophobic stabilization within the lipophilic channel, and direct interaction with the catalytic zinc ion.

### 2.11 Flow Cytometric Evaluation, Annexin V–PI Assay, Western Blot Analysis (Apoptosis and Autophagy Markers), and Staining Assays

To evaluate the effect of compound **14** on cell cycle progression, MV4–11 cells were treated with increasing concentrations (0.25, 0.5, and 1 μM) for 24 h and analyzed by flow cytometry following PI staining. Compared to control cells, which predominantly resided in the G0/G1 phase, a dose-dependent increase in the Sub-G1 population, indicative of apoptotic cell death, was observed with compound **14** treatment. Notably, the increase in Sub-G1 population was accompanied by a progressive decline in the G0/G1 fraction, while only minor changes were observed in the S and G2/M phases. This outcome suggests that compound **14** does not induce a strong phase-specific arrest, as no significant accumulation of cells at S or G2/M checkpoints was detected. Instead, these findings indicate that its primary mechanism of action is the induction of apoptosis. Consistently, treatment with SAHA (1 μM) produced a comparable increase in Sub-G1 cells, supporting the validity of the observed effect (Figure 7A).

**Figure 7:**
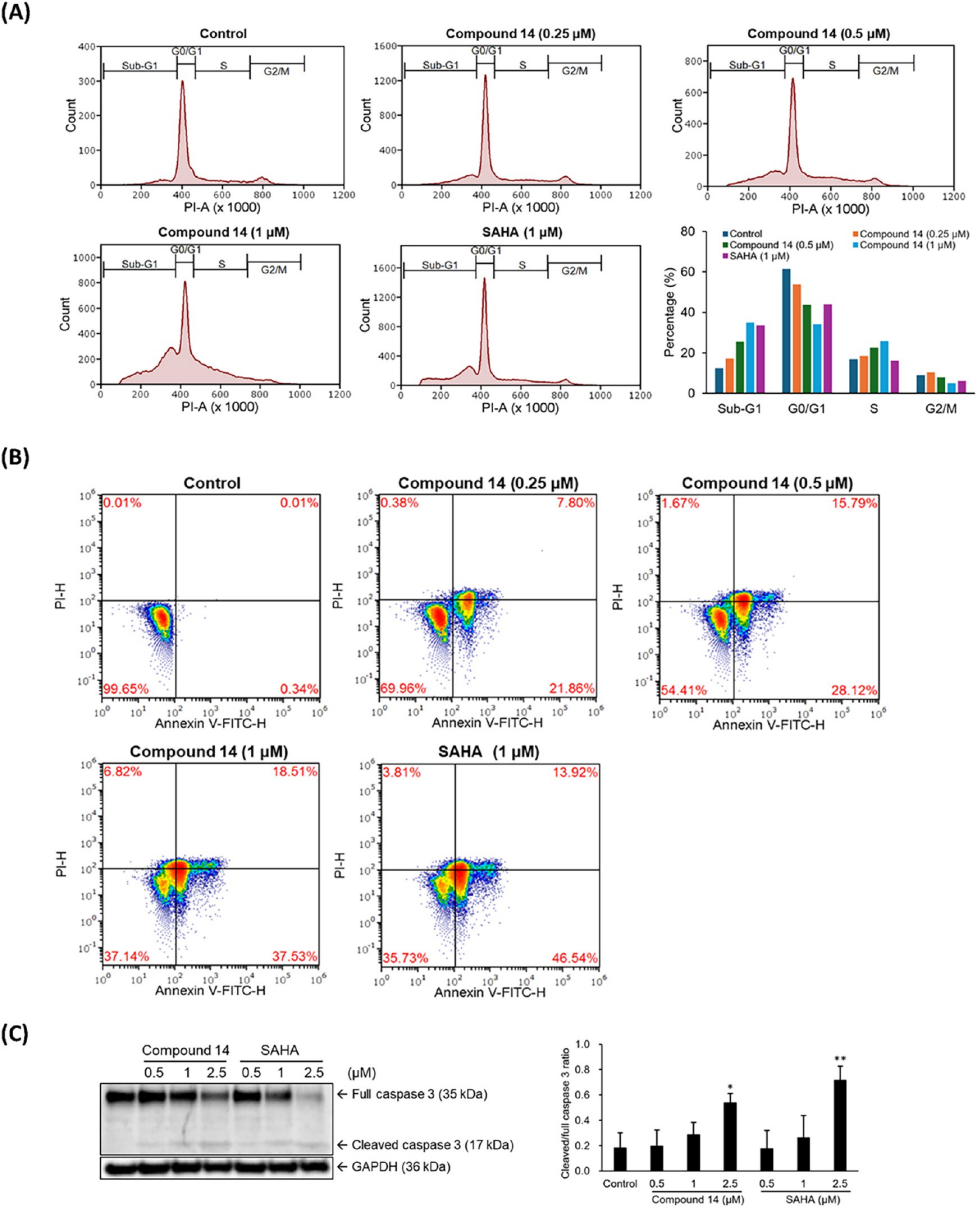

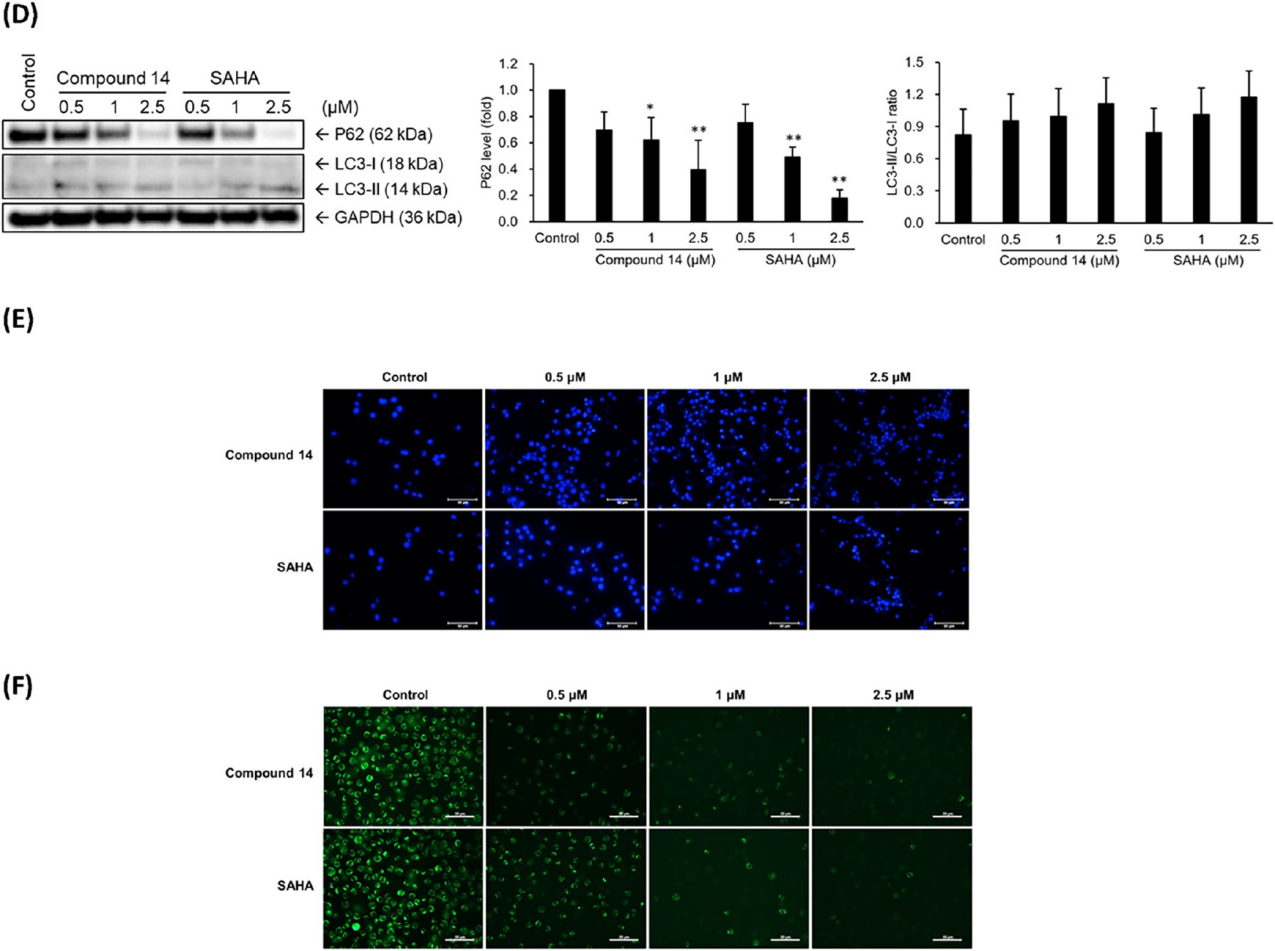
(A) Impact of compound **14** on the distribution of cell cycle phases in MV4–11 cells. The cells were incubated with compound **14** or SAHA for 24 h. After treatment, cells were harvested, and the distribution of cell cycle phases was analyzed by flow cytometry. (B) **Apoptosis-inducing effect of** compound **14 in MV4–11 cells**. Cells were incubated with compound **14** or SAHA for 24 h. After the treatment, cells were harvested and stained using propidium iodide (PI) and annexin V-FITC for 30 min. The apoptotic cells were subsequently identified using flow cytometry. (C) Western blot analysis to determine the expression level of cleaved caspase-3 with compound **14** treatment. (D) Western blot analysis to determine the expression level of p62 and LC3-II with compound **14** treatment. (E) Analysis of nuclear morphology by DAPI staining in MV4–11 cells. (F) Evaluation of mitochondrial membrane potential by rhodamine 123 staining in MV4–11 cells.

The pro-apoptotic effect of compound **14** was evaluated in MV4–11 cells using Annexin V-FITC/PI dual staining followed by flow cytometry after 24 h of treatment. The vast majority of the population remained viable (Annexin V^–^/PI^–^, 99.65%), with negligible levels of apoptosis (0.35%) in the control cells. A significant and dose-dependent increase in apoptotic cell populations was observed with compound **14**. At 0.25 μM, early and late apoptotic cells increased to 21.86% and 7.80%, respectively (total 29.7%). Increasing the concentration to 0.5 μM further elevated apoptosis to 43.9% (early 28.12%, late 15.79%). At 1 μM, compound **14** induced a substantial apoptotic response, with 37.53% early and 18.51% late apoptotic cells (total 56.0%). Notably, treatment with SAHA (1 μM) demonstrated a comparable apoptotic profile (Figure 7B).

Western blot analysis was performed to evaluate the ability of compound **14** to induce caspase-3 cleavage (Figure 7C). Resultantly, it was observed that the compound could cause the cleavage of caspase-3, indicating its potential to induce apoptosis. Notably, a significant increase in the cleaved/total caspase-3 ratio at 2.5 μM (*p* < 0.01) was revealed by the quantitative analysis. Also, compound **14** treatment led to enhanced autophagic flux as evidenced by a progressive reduction of p62 levels. In addition, compound **14** also demonstrated moderate ability to induce autophagy, as evidenced by increased LC3-II accumulation and a higher LC3-II/LC3-I ratio (Figure 7D).

DAPI staining was further performed to validate the induction of the apoptosis. The outcome of the study revealed that compound **14** dose-dependently led to significant changes in nuclear architecture (chromatin condensation and nuclear fragmentation) in a dose-dependent manner. Also, the formation of apoptotic bodies was observed in the morphology of the MV4–11 cells. Notably, the aforementioned alterations were only observed in the nuclear morphology of Compound **14**-treated cells, whereas the untreated cells demonstrated normal intact morphology (Figure 7E). Overall, the outcome of this study aligns with the results of annexin V/PI flow cytometry and caspase-3 cleavage data, ascertaining the apoptosis-triggering ability of compound **14**. Rhodamine 123 staining was also performed to confirm the apoptosis-inducing ability of Compound **14**. Intact mitochondrial function was observed with control cells, as evidenced by strong green fluorescence, whereas Compound **14**-treated cells exhibited a decline in rhodamine fluorescence in a concentration-dependent manner, underscoring the mitochondrial-disrupting ability of Compound **14** (Figure 7F). Thus, it was deduced that Compound **14** exerts apoptosis through mitochondrial membrane potential loss.

### 2.12 Transcriptomic Profiling of Compound **14**

MV4–11 cells were treated with compound **14** for 48 h, followed by total RNA extraction using the NucleoSpin RNA kit according to the manufacturer’s protocol. RNA sequencing was performed by BIOTOOLS Co. (New Taipei City, Taiwan). Transcriptomic profiling and gene set enrichment analysis (GSEA) revealed significantly suppressed lipid-associated metabolic pathways with compound **14** treatment. A downregulatory pattern of key molecules involved in sterol and fatty acid biosynthesis, such as DHCR24, FASN, LDLR, SCD, and SREBF1, in the compound-treated group relative to control was observed (heatmap analyses). Also, a strong negative enrichment score, as demonstrated by GSEA enrichment plots (Figure 8), indicates the ability of compound **14** to attenuate cholesterol and fatty acid metabolic programs at the transcriptomic level.

**Figure 8:**
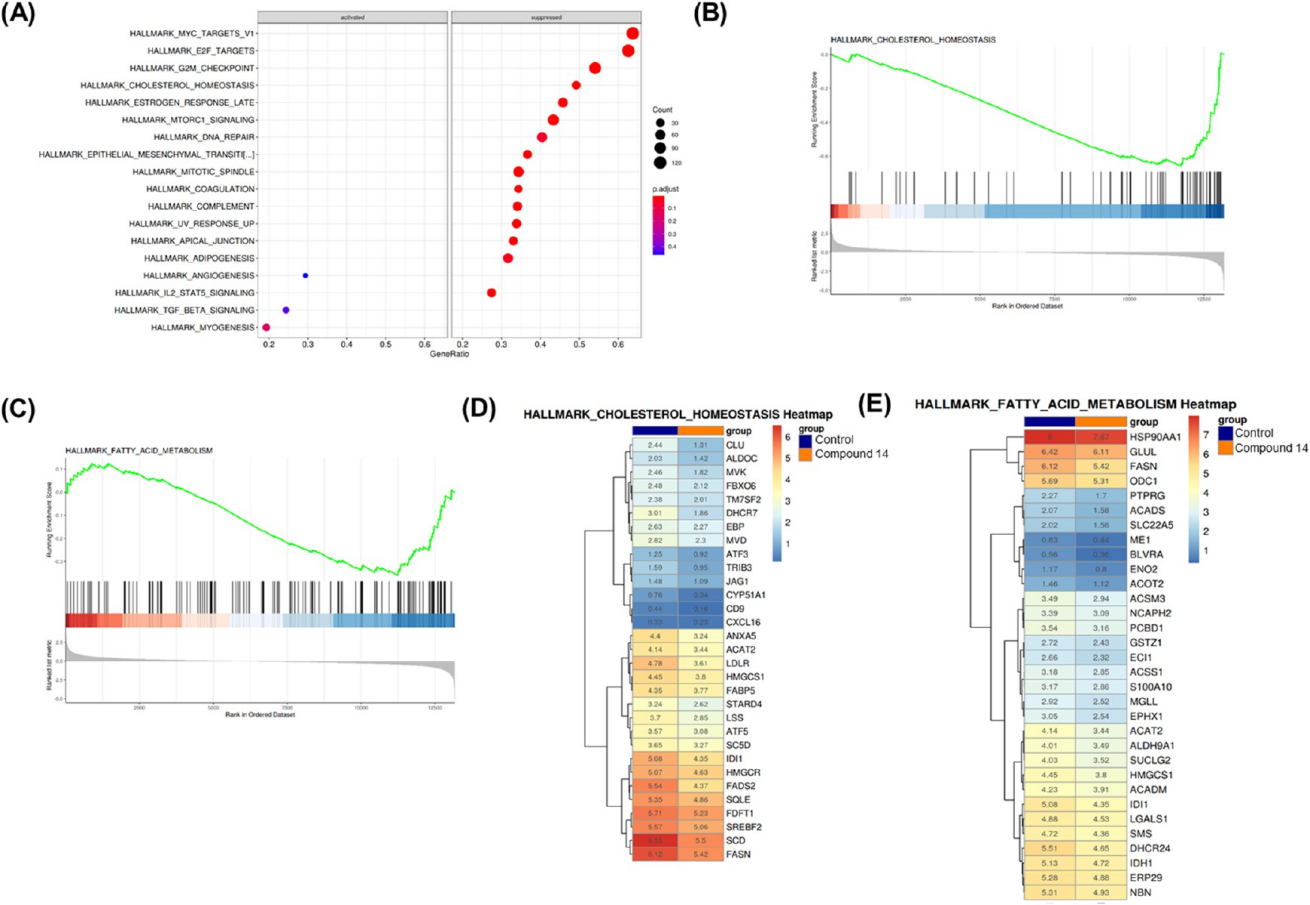
Gene set enrichment analysis (GSEA) of compound **14** in MV4–11 cells. (A) Summary plot of GSEA. (B) GSEA plot for cholesterol homeostasis (C) GSEA plot for fatty acid metabolism. (D) Heatmap of compound **14** for cholesterol homeostasis. (E) Heatmap of compound **14** for fatty acid metabolism.

Differential expression profiling was observed with a volcano plot (Figure 9A), indicating upregulation and downregulation of several genes, while GO enrichment analysis (Figure 9B) indicated notable modulation of biological processes related to lipid metabolism, fatty acid biosynthetic process, and cholesterol homeostasis. KEGG pathway enrichment analysis further revealed that several downregulated KEGG pathways were related to lipid metabolism, including fatty acid metabolism and biosynthesis, the PPAR signaling pathway, and the biosynthesis of unsaturated fatty acids (Figure 9C). Among the differentially expressed genes associated with lipid metabolism, SREBF1 serves as a key upstream transcription factor that promotes the expression of numerous genes involved in the metabolism and biosynthesis of fatty acids and cholesterol.^61–64^

**Figure 9:**
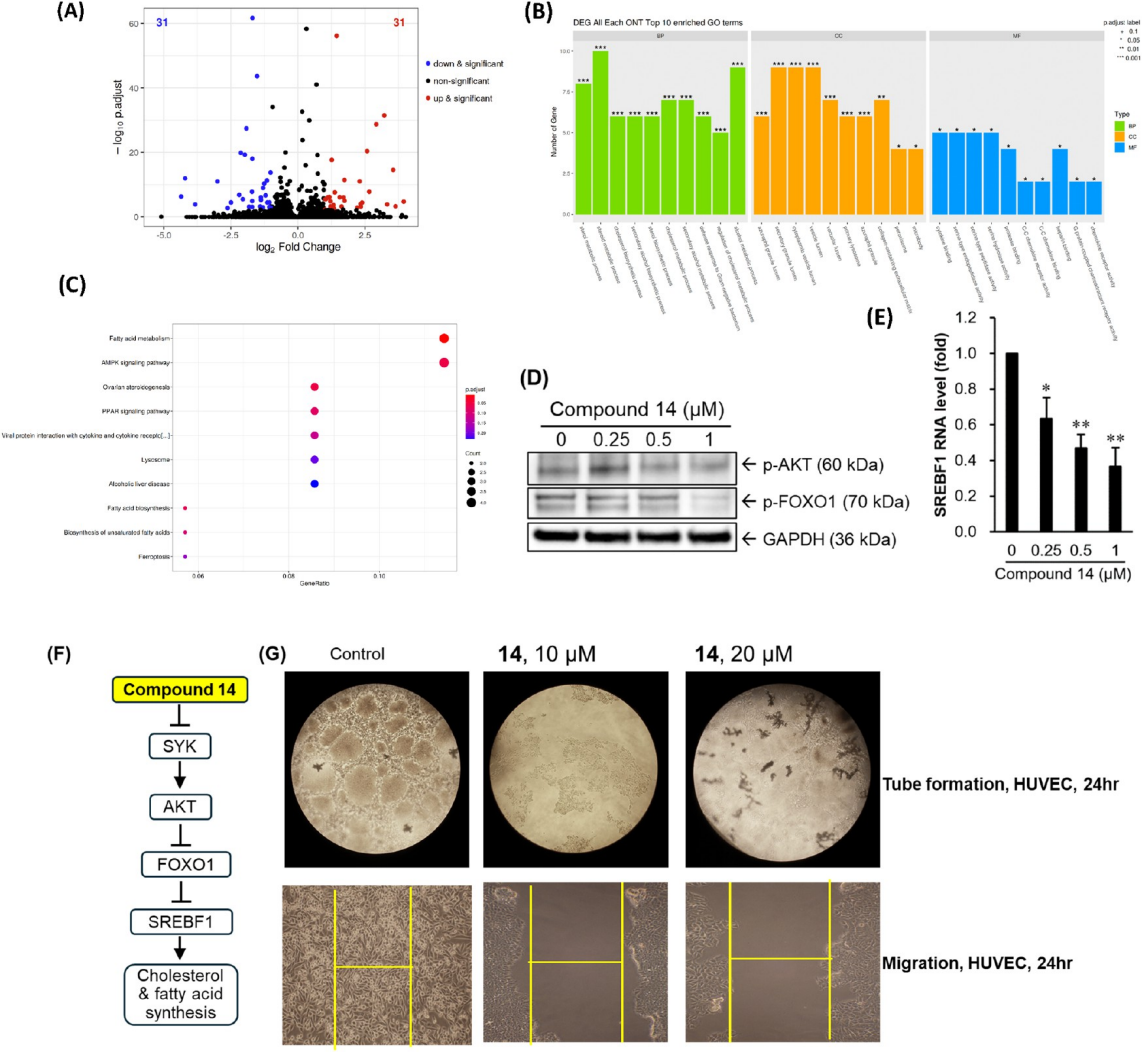
Transcriptomic and functional characterization of compound 14 in MV4–11 cells. (A) Differentially expressed genes (DEGs) were analyzed by RNA sequencing and shown as a volcano plot. (B) Gene Ontology (GO) enrichment analysis of DEGs. (C) Dotplot of KEGG enrichment analysis results. (D) Activation level of downstream proteins of SYK in MV4–11 cells treated with compound **14** for 24 h. (E) Gene expression level of SREBF1 in MV4–11 cells treated with compound **14** for 24 h. (F) Representative mechanism of compound **14** in inhibiting MV4–11 cells. (G) Effect of compound **14** on the endothelial tube formation assay and migration assay.

Literature precedents reveal the dependence of AML cell proliferation on metabolic reprogramming. In particular, enhanced de novo lipogenesis is required for membrane biosynthesis and energy production in rapidly dividing leukemic blasts.^61,62,65–67^ Specifically, the enhanced activity of SREBF1 has been associated with the prolonged survival and proliferation of AML cells. Several reports indicate that SREBF1 inhibition leads to metabolic stress and reduced leukemic cell viability.^64,68^ In our study, Compound **14** induced a dose-dependent downregulation of SREBF1 mRNA levels (Figure 9E), indicating suppression of lipogenic signaling pathways. This reduction is expected to impair lipid biosynthesis, thereby limiting the availability of essential components required for the AML cell growth. Additionally, the functional link of SREBF1 to oncogenic PI3K/AKT signaling has also been well established, and its downregulation has been reported to cause a reduction in tumor cell proliferation and promote apoptosis.^64,68^

In B cell receptor (BCR)-dependent diffuse large B cell lymphoma (DLBCL), SYK/PI3K signaling controls cholesterol biosynthesis and blocks SYK/PI3K triggers HRK-dependent apoptosis.^69^ A previous study reported that SYK inhibition can activate FOXO1 by reducing AKT-mediated phosphorylation on FOXO1 in tonic BCR-dependent DLBCL.^70^ Active (or dephosphorylated) FOXO1 has been shown to suppress the transcriptional level of the SREBF1 gene. Therefore, the phosphorylation levels of AKT and FOXO1 were measured in MV4–11 cells treated with compound **14**. Our results indicated that the intervention with compound **14**, a dual SYK-HDAC inhibitor, can suppress AKT activation, leading to a decrease in the phosphorylation level of FOXO1 and subsequently activating FOXO1 (Figure 9D). Furthermore, a reduction in the transcriptional level of SREBF1 was also confirmed in MV4–11 cells after treatment with compound **14** (Figure 9E). Accordingly, the representative mechanisms by which compound **14** downregulates lipid metabolism and biosynthesis are briefly summarized in Figure 9F.

GSEA analysis results revealed downregulation of an angiogenesis-associated gene set following compound **14** treatment. To explore whether this transcriptional trend was accompanied by altered endothelial cell behavior, an endothelial tube formation assay and migration assay were performed (Figure 9G). Notably, in the tube formation assay, compound **14** treatment resulted in reduced tube formation in a dose-dependent manner. A moderate decrease in branching and network integrity was observed with compound **14** treatment (10 μM), while a higher dose of compound **14** (20 μM) led to a more pronounced loss of tubular structures. Likewise, dose-dependent impairment of cell migration, as evidenced by a wider scratch gap, was observed with compound **14** treatment in the migration assay.

### 2.13 Pharmacokinetic Characterization of Compound **14**

A pharmacokinetic study of compound **14** was conducted in mice. The compound was administered *via* three different routes, *viz*., Intravenous (IV), intraperitoneal (IP), and oral. Notably, a terminal half-life (*t*_1/2_) of 370.32 ± 17.82 min, a mean residence time (MRT) of 516.41 ± 24.29 min, and an AUC_0_–∞ of 7559.45 ± 359.35 ng·mL^–1^·min were observed in the IV arm (1 mg/kg), indicative of prolonged systemic exposure. A comparable pharmacokinetic profile was demonstrated by compound **14** on IP administration (1 mg/kg) with a *t*_1/2_ of 275.02 ± 10.52 min, MRT of 418.44 ± 18.86 min, and an AUC_0_–∞ of 6144.60 ± 293.30 ng·mL^–1^·min, corresponding to a high systemic bioavailability of 81.28 ± 4.65%. On the contrary, significantly lower systemic exposure was observed with oral administration of compound **14**. Limited gastrointestinal absorption is anticipated on oral administration of compound **14**, as the low bioavailability (19.74 ± 1.04%) was observed. Also, slow absorption kinetics were demonstrated by compound **14** on oral administration as evidenced by extended *t*_1/2_ (416.81 ± 20.90 min) and prolonged MRT (695.33 ± 28.22 min). On the basis of the aforementioned data, IP administration of compound **14** was selected as the preferred route for *in vivo* antileukemic evaluation, owing to a high systemic bioavailability (∼81%) observed with this route of administration, coupled with ease of administration (Table 4 and Figure 15S).

**Table 4:** Results of *In Vivo* Pharmacokinetics of Compound **14** *via* Three Different Routes, *viz*., IV, IP, and Oral

	administration route		
parameters	IP administration (1 mg/kg)	IV administration (1 mg/kg)	oral administration (50 mg/kg)
*t*_1/2_ (min)	275.02 ± 10.52	370.32 ± 17.82	416.81 ± 20.90
*T*_max_ (min)	120.00 ± 5.08	60.00 ± 2.19	240.00 ± 10.87
*C*_max_ (ng/mL)	14.60 ± 0.74	17.55 ± 0.95	123.95 ± 5.89
AUC_0–*t*_ (ng/mL·min)	6144.60 ± 293.30	7559.45 ± 359.35	74638.50 ± 3956.15
MRT_0‑inf_ (min)	418.44 ± 18.86	516.41 ± 24.29	695.33 ± 28.22
bioavailability (%)	81.28 ± 4.65	100.00	19.74 ± 1.04

### 2.14 *In Vivo* Pharmacodynamics and *In Vivo* Target Engagement Study of Compound **14**

Compound **14** was subjected to *in vivo* anticancer evaluation in the FLT3-ITD-positive AML xenograft mouse model. MV4–11 cells were subcutaneously implanted in Balb/c nude mice, and compound **14** was administered IP at doses of 25 and 50 mg/kg (Figure 10A). SAHA was used as the positive control for the comparative analysis. Throughout the treatment period, no significant changes in body weight were observed with compound **14** at both doses (25 mg/kg and 50 mg/kg) (Figure 10B). A dose-dependent antitumor efficacy was evidenced with compound **14** in tumor volume measurements, with compound **14** eliciting remarkable tumor growth suppression effects relative to those of the vehicle control. Notably, Compound **14** demonstrated a more pronounced tumor growth inhibition potential than SAHA (Figure 10C). Replicating the trends of the tumor volume measurement study, compound **14** at the dose of 50 mg/kg exerted a marked reduction in the tumor weight relative to the control group. It is important to mention that compound **14**, at the dose of 50 mg/kg, exerted approximately an 80% decrease in the tumor mass, while **SAHA** at the same dose led to a 42% decrease in the tumor weight (Figure 10D). The quantitative assessment of the antileukemic efficacy of compound **14** *via* tumor weight analysis corroborates with smaller tumor burdens in the compound **14**-treated group (50 mg/kg) as observed in representative tumor-bearing mice images (Figure 10E). Overall, the outcome of this study ascertains that compound **14** is endowed with substantial *in vivo* anti-AML efficacy and supports further preclinical evaluation as an anti-AML agent.

**Figure 10:**
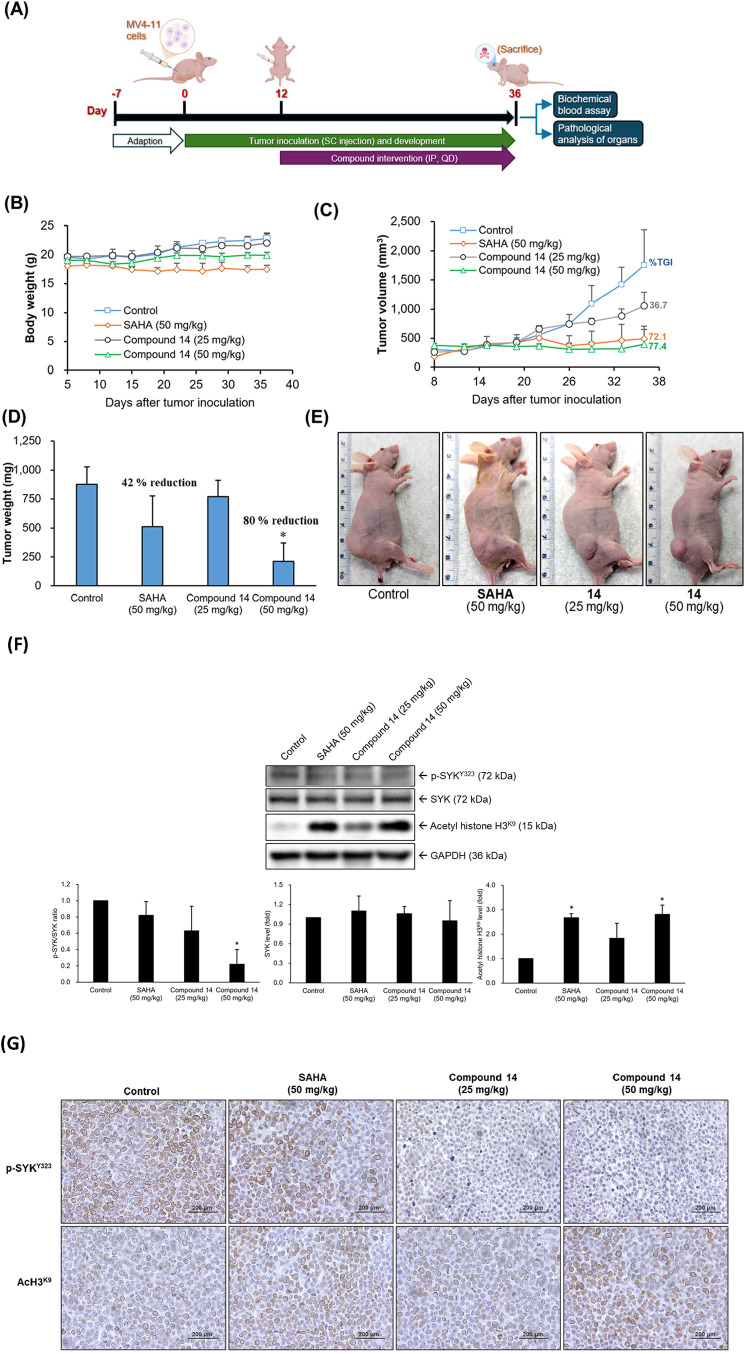
Antitumor activity of compound **14** was assessed in an MV4–11 xenograft mouse model. (A) Timeline and experimental design of the *in vivo* study. (B) Body weight and (C) Tumor volume were measured twice a week following tumor inoculation. (D) Tumor weight measurement. (E) Representative photographs of MV4–11 xenograft-bearing mice at the end of the treatment period. (F) Effect of compound **14** on SYK and histone H3 activity in a murine xenograft leukemia model. The tumors from each group were harvested to analyze SYKY323 and histone H3K9 expression levels. * *p* < 0.05 compared to the control group. (G) Immunohistochemistry analysis of tumor specimens in murine xenograft leukemia models. The expression and distribution of phosphorylated SYK^Y323^ and acetylated histone H3^K9^ in tumor specimens were detected by IHC, visualized using a DAB chromogen that produced a brown color.

Further, the *in vivo* target engagement study of compound **14** in a murine xenograft leukemia model was carried out. Western blot analysis was performed on tumor samples harvested from treated animals to determine the levels of phosphorylated SYK (p-SYK^Y323^) and acetylated histone H3K9. The results indicated a dose-dependent decrease in p-SYK^Y323^ levels following treatment with Compound **14**. Notably, the total SYK expression remained unchanged, suggesting inhibition of kinase activity without affecting protein abundance. Quantitative analysis further revealed a significant reduction in the p-SYK/SYK ratio with compound **14** at the dose of 50 mg/kg compared to the vehicle (*p* < 0.05). In parallel, Compound **14** induced a concentration-dependent increase in the acetylation levels of histone H3K9, consistent with HDAC inhibition. Notably, a statistically significant elevation in acetylation was detected at the higher dose of Compound **14** (50 mg/kg) (*p* < 0.05). While compound **14** modulated both p-SYK and acetyl histone levels, SAHA treatment only induced histone acetylation and had minimal impact on SYK phosphorylation. These outcomes indicate that compound **14** effectively engages both targets *in vivo* (SYK and HDAC) and supports its candidacy as a dual SYK/HDAC inhibitor.

Also, immunohistochemical (IHC) analysis was performed on tumor sections collected from the murine leukemia xenograft model following treatment with compound **14** and SAHA. Strong p-SYK^Y323^ staining, indicative of active SYK signaling, along with low levels of AcH3^K9^, was observed in tumors from vehicle-treated animals. A significant increase in AcH3K9 staining was observed with SAHA treatment (50 mg/kg), confirming effective HDAC inhibition *in vivo*. As expected, SAHA treatment resulted in only a modest decrease in p-SYK levels. In contrast, a dose-dependent decrease in the expression of p-SYK^Y323^, accompanied by a corresponding increase in AcH3K9 levels, was exerted by Compound **14**. At a dose of 25 mg/kg, compound **14** produced moderate suppression of SYK phosphorylation and increased histone acetylation, whereas the higher dose of compound **14** (50 mg/kg) resulted in pronounced reduction in p-SYK staining along with robust induction of AcH3K9. The aforementioned findings indicate effective *in vivo* target engagement by compound **14**, leading to simultaneous inhibition of SYK phosphorylation and enhancement of histone acetylation, consistent with its proposed dual mechanism of action.

### 2.15 Biochemical Blood Analysis

A comprehensive biochemical blood analysis was performed to assess the safety and tolerability profile of compound **14** (Table 5). The serum levels of aspartate aminotransferase (AST), alanine transaminase (ALT), blood urea nitrogen (BUN), creatine phosphokinase (CPK), and creatinine (CRE) were measured by the National Center for Biomodels (Taipei, Taiwan). Notably, mice treated with SAHA exhibited marked elevation in CPK (718.5 ± 145.3 U/L) in comparison to the vehicle-treated control group (CPK = 325.1 ± 145.3 U/L), consistent with increased muscular stress. Also, modest elevation in ALT and BUN levels was observed with SAHA in comparison to the vehicle-treated control group (ALT = 33.0 ± 4.6 U/L, BUN = 42.0 ± 8.1 mg/dL), while CRE levels remained unchanged, indicating no apparent impairment of renal function. On the contrary, all of the biochemical indices were maintained with compound **14** at both the doses (25 mg/kg and 50 mg/kg) within the physiological range (ALT = 36.0 ± 7.9 and 34.4 ± 4.8 U/L; BUN = 31.5 ± 5.0 and 35.5 ± 2.7 mg/dL; CRE = 0.093 ± 0.009 and 0.083 ± 0.007 mg/dL; CPK = 98.8 ± 34.1 and 255.5 ± 101.0 U/L, respectively). Thus, the results indicate preserved hepatic and renal function with compound **14** treatment. Also, any significant biochemical indication of muscle toxicity was not observed with compound **14**. Overall, the results of biochemical blood analysis support the favorable systemic tolerability of compound **14**.

**Table 5:** Biochemical Blood Analysis of the Xenograft Mouse Tumor Model^a^

	parameters			
groups	ALT (U/L)	BUN (mg/dL)	CRE (mg/dL)	CPK (U/L)
Control	33.0 ± 4.6	42.0 ± 8.1	0.097 ± 0.003	325.1 ± 145.3
50 mg/kg SAHA	38.5 ± 5.6	47.0 ± 7.6	0.097 ± 0.015	718.5 ± 145.3
25 mg/kg Compound **14**	36.0 ± 7.9	31.5 ± 5.0	0.093 ± 0.009	98.8 ± 34.1
50 mg/kg Compound **14**	34.4 ± 4.8	35.5 ± 2.7	0.083 ± 0.007	255.5 ± 101.0

a ALT, alanine aminotransferase; BUN, blood urea nitrogen; CRE, creatinine; and CPK, creatinine phosphokinase.

### 2.16 Histopathological Evaluation

Various organs, including the heart, liver, spleen, lungs, and kidneys, were collected from sacrificed animals to examine histopathological changes. The organs underwent paraffin embedding, tissue sectioning, and hematoxylin-eosin (HE) staining, which were carried out by the Laboratory Animal Center of TMU. Control and SAHA were used for comparison of the impact of compound **14** on the morphology of the major organs. Resultantly, any noticeable morphological alterations were not observed in cardiac, hepatic, splenic, pulmonary, or renal tissues with compound **14** at doses of 25 mg/kg and 50 mg/kg. In contrast, mild hepatic cellular swelling, degenerative changes in myocardium, focal inflammatory infiltration, and slight tubular congestion in kidney sections, consistent with mild tissue toxicity, were observed in the SAHA (Figure 11). In a nutshell, the compound **14**-treated group could preserve the normal histology across all of the organs, indicating a more favorable histopathological tolerability in comparison to SAHA.

**Figure 11:**
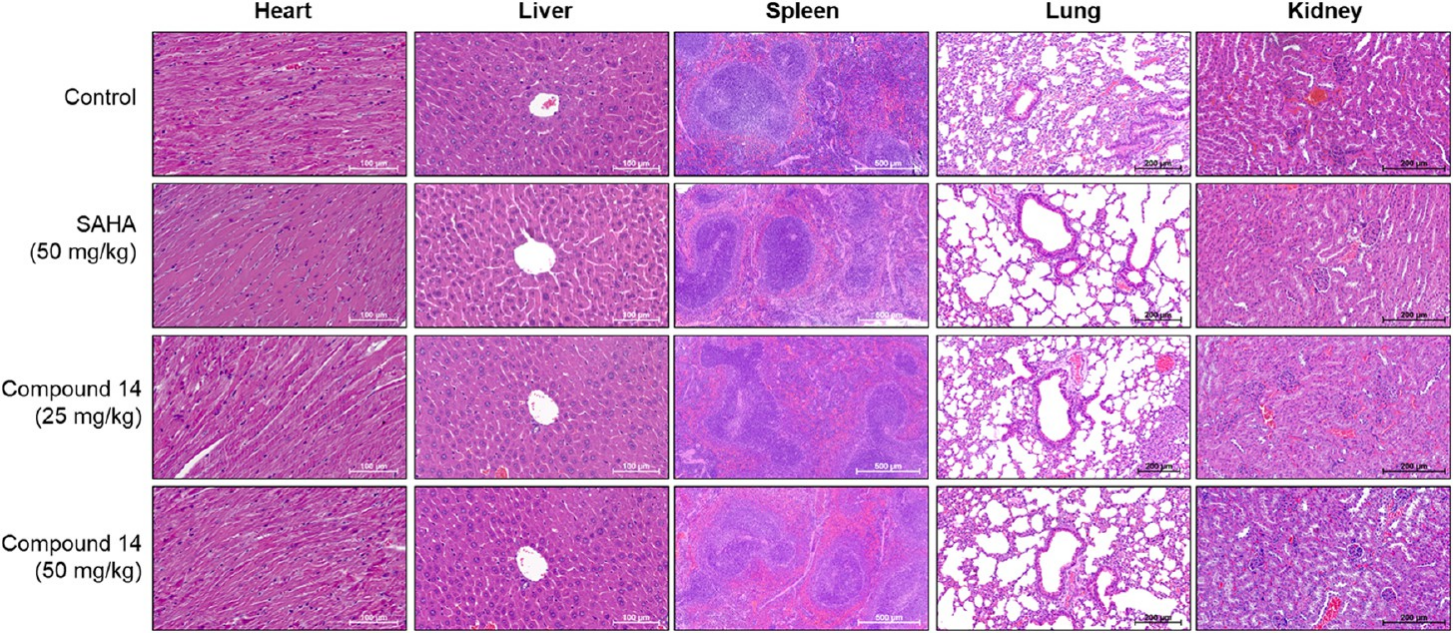
Histopathological analysis of the organs harvested from the *in vivo* study.

## 3 Conclusion

The growth trajectory of spleen tyrosine kinase (SYK) inhibitors has been marred by a lack of entries in the FDA-approved drug repository. Several meticulously optimized SYK antagonists have progressed to advanced stage evaluations; however, insufficient antitumor efficacy coupled with modest pharmacodynamic resilience in AML has hindered their transition from investigational drug armory to clinical drug pipeline. Similarly, the application horizons of inhibitors targeting the most established epigenetic target, histone deacetylases, have also demonstrated restricted therapeutic bandwidth due to several dose-limiting toxicities and modest clinical responses in AML. Noteworthy is the fact that both SYK and multiple HDAC isoforms are overexpressed in acute myeloid leukemia (AML), and SYK-mediated kinase signaling intersects with HDAC-regulated epigenetic circuits in leukemia. Despite these revelations, therapeutic success targeting these axes individually has not yielded the expected outcomes in terms of anti-AML effects. Intrigued by the aforementioned constraints associated with the monotonic application of SYK and HDAC inhibitors in AML, the approach of simultaneously modulating SYK and HDAC *via* dual SYK-HDAC inhibitors to attain enhanced efficacy in AML was tested in the present study. The strategy leveraged for the design of the hybrid structure involved the installation of the chemical architecture of entospletinib (investigational SYK inhibitor) as the surface recognition part of the HDAC inhibitor three-component model. Subsequently, the designed scaffolds were synthesized and evaluated for anti-AML effects against MV4–11 cell lines (AML cell lines harboring FLT3-ITD mutations). Resultantly, compound **14** was pinpointed through the *in vitro* cytotoxicity studies as a strikingly potent cell growth inhibitor of MV4–11 cell lines. The outcome of *in vitro* SYK inhibition and HDAC isoform inhibition assay revealed that the remarkable cytotoxicity of compound **14** stems from dual balanced modulation of SYK and HDAC. Docking studies conducted to rationalize the enzymatic inhibition assay results indicated that compound **14** interacted with various amino acid residues of SYK and HDAC isoforms. Further explorations conducted to elucidate the underlying mechanism of cell growth repression exerted by the compound **14** confirmed its ability to cause cell cycle arrest at the Sub-G1 phase . Also, a dose-dependent increase in apoptotic cells in MV4–11 cells with compound **14** was observed (Annexin V-PI assay), thereby positioning it as an apoptosis inducer. Findings from DAPI staining assay, Western blot analysis (caspase-3 cleavage), and rhodamine staining assay corroborated with the results of Annexin-V assay and flow cytometry, underscoring the apoptosis induction ability of compound **14**. Exhaustive transcriptomic profiling revealed that compound **14** could suppress lipid-associated metabolic pathways. The pharmacokinetic profiling of compound **14** was conducted in mice *via* three different routes (IV, IP, and oral), and high systemic bioavailability was observed with IP administration. Thus, IP administration of compound **14** was selected as the preferred route for the *in vivo* antitumor study in the FLT3-ITD-positive AML xenograft mouse model. Resultantly, compound **14** exerted significant tumor growth inhibitory potential, as evidenced through tumor volume measurements and tumor weight analysis. Also, compound **14** demonstrated an excellent systemic profile, as evident from the results of biochemical blood assay, and could preserve the normal histology across all of the organs (heart, liver, spleen, lungs, and kidneys). Overall, compound **14** demonstrates the potential as a preclinical tractable dual HDAC–SYK inhibitor for AML therapy.

## 4 Experimental Section

### 4.1 Chemistry

A Bruker DRX-500 spectrometer (600, 500, and 300 MHz) was used to record ^1^H and ^13^C NMR spectra. High-resolution mass spectra (HRMS) were obtained by using a JEOL JMS-700 electron impact (EI) mass spectrometer. The purity of the final compounds was assessed by using a Hitachi 2000 series HPLC system equipped with an Agilent ZORBAX Eclipse XDB-C18 column (5 mm particle size). The column measured 4.6 mm in diameter by 150 mm in length, and all compounds showed over 95% purity by HPLC analysis. Column chromatography was performed using silica gel (Merck Kieselgel 60, No. 9385, 230e400 mesh ASTM)

#### 4.1.1 6-Bromo-*N*-(4-morpholinophenyl)­imidazo­[1,2-*a*]­pyrazin-8-amine (**20**)

To the stirred solution of intermediate 6,8-dibromoimidazo­[1,2-*a*]­pyrazine (19) (6.98 g, 25.23 mmol), 4-morpholino-4-yl-phenylamine (3.14 g, 17.66 mmol) isopropyl alcohol (60 mL) and DIPEA (3.91 g, 30.28 mmol) was added dropwise to the reaction mixture and the reaction mixture was allowed to stir at 80 °C for 4 h. After the completion of the reaction, cold water was added to the reaction mixture, and resultant precipitates were filtered out and dried under high vacuum and further purified by using column chromatograhy using hexane:EA (6:4) as solvent to obtain intermediate 20. Light brown powder, yield 96.20%. ^1^H NMR (300 MHz, DMSO-*d*_6_) δ (ppm) 9.78 (s, 1H), 8.18 (s, 1H), 7.92 (s, 1H), 7.81 (d, *J* = 9.0 Hz, 2H), 7.60 (s, 1H), 6.96 (d, *J* = 9.0 Hz, 2H), 3.75 (t, *J* = 6.0 Hz, 4H), 3.09 (t, *J* = 6.0 Hz, 4H).

#### 4.1.2 *tert*-Butyl 4-(4-((6-bromoimidazo­[1,2-*a*]­pyrazin-8-yl)­amino)­phenyl)­piperazine-1-carboxylate (**21**)

To the stirred solution of intermediate 6,8-dibromoimidazo­[1,2-*a*]­pyrazine (19) (0.713 g, 2.57 mmol), *tert*-butyl 4-(4-aminophenyl) piperazine-1-carboxylate (0.359 g, 2.89 mmol) and isopropyl alcohol (20 mL), in which DIPEA (1.35 g, 10.80 mmol), were added dropwise to the reaction mixture and allowed to stir at 80 °C for 4 h. After the completion of the reaction, the cold water was added to the reaction mixture, and the resultant precipitates were filtered out and dried under high vacuum and further purified by using column chromatograhy using hexane:EA (6:4) as solvent to obtain intermediates (21–23). Light brown powder, yield 82.31%. ^1^H NMR (300 MHz, DMSO-*d*_6_) δ (ppm) 9.80 (s, 1H), 8.20 (s, 1H), 7.93 (s, 1H), 7.82 (d, *J* = 9.0 Hz, 2H), 7.62 (s, 1H), 6.99 (d, *J* = 9.0 Hz, 2H), 3.48 (s, 4H), 3.09 (s, 4H), 1.44 (s, 9H).

#### 4.1.3 6-Bromo-*N*-phenylimidazo­[1,2-*a*]­pyrazin-8-amine (**22**)

Intermediate 22 was synthesized by using intermediate (19), aniline (0.200 g, 2.14 mmol), and the same synthetic methodology was utilized as for the synthesis of intermediate 21. Brown powder, yield 29.03%. ^1^H NMR (300 MHz, DMSO-*d*_6_) δ (ppm) 9.98 (s, 1H), 8.28 (s, 1H), 7.98 (d, *J* = 6.0 Hz, 3H), 7.65 (s, 1H), 7.38 (t, *J* = 9.0 Hz, 2H), 7.08 (t, *J* = 6.0 Hz, 1H).

#### 4.1.4 *tert*-Butyl 4-(6-bromoimidazo­[1,2-*a*]­pyrazin-8-yl)­piperazine-1-carboxylate (**23**)

Intermediate 23 was synthesized by using intermediate (19), N-Boc piperazine (0.396 g, 2.14 mmol), and the same synthetic methodology was utilized as for the synthesis of intermediate 21. Brown powder, yield 70.26%. ^1^H NMR (300 MHz, DMSO-*d*_6_) δ (ppm) 8.17 (s, 1H), 7.90 (s, 1H), 7.57 (s, 1H), 4.21 (s, 4H), 3.49 (d, *J* = 21 Hz, 4H), 1.43 (m, 9H).

### 4.2 General Procedure for the Synthesis of Entospletinib Derivatives **ED 1–27**

To the stirred solution of intermediate 20 (0.200 g, 0.53 mmol), various boronic acids (1–1.5 equiv) and sodium carbonate (0.169 g, 1.60 mmol) in *p*-dioxane:water (9:1, 10 mL) were added, and the reaction mixture was allowed to stir at room temperature with nitrogen purging for about 15 min, and then Pd­(PPh_3_)_4_ (0.123 g, 0.10 mmol) was added to the reaction mixture and further allowed to stir for 3 h at 100 °C. After the completion of the reaction, cold water was added to the reaction mixture, and the resultant precipitates were filtered out and dried under vacuum. The crude product was further purified by using column chromatography using DCM:MeOH (9.5:0.5) as solvent to obtain **ED 1–27**.

#### 4.2.1 *N*-(4-Morpholinophenyl)-6-phenylimidazo­[1,2-*a*]­pyrazin-8-amine (**ED1**)

Yellow powder, yield 21.64%. ^1^H NMR (300 MHz, DMSO-*d*_6_) δ (ppm) 9.48 (s, 1H), 8.57 (s, 1H), 8.02 (m, 5H), 7.63 (s, 1H), 7.51 (t, *J* = 6 Hz, 2H), 7.40 (d, *J* = 6 Hz, 1H), 7.00 (d, *J* = 9 Hz, 2H), 3.77 (d, *J* = 6 Hz, 4H), 3.10 (d, *J* = 3 Hz, 4H).

#### 4.2.2 *N*-(4-Morpholinophenyl)-6-(pyridin-3-yl)­imidazo­[1,2-*a*]­pyrazin-8-amine (**ED-2**)

Off white, yield 26.86%. ^1^H NMR (300 MHz, DMSO-*d*_6_) δ (ppm) 9.56 (s, 1H), 9.19 (d, *J* = 3 Hz, 1H), 8.67 (s, 1H), 8.58 (m, 1H), 8.32 (m, 3H), 7.65 (s, 1H), 7.52 (m, 1H), 7.00 (d, *J* = 9 Hz, 2H), 3.76 (t, *J* = 3 Hz, 4H), 3.10 (t, *J* = 3 Hz, 4H).

#### 4.2.3 *N*-(4-Morpholinophenyl)-6-(quinolin-6-yl)­imidazo­[1,2-*a*]­pyrazin-8-amine (**ED-3**)

Yellow solid, yield 15.18%. ^1^H NMR (300 MHz, DMSO-*d*_6_) δ (ppm) 9.57 (s, 1H), 8.92 (m, 1H), 8.78 (s, 1H), 8.59 (d, *J* = 3 Hz, 1H), 8.48 (d, *J* = 9 Hz, 1H), 8.39 (m, 1H), 8.13 (m, 4H), 7.67 (s, 1H), 7.60 (m, 1H), 7.06 (d, *J* = 9 Hz, 2H), 3.79 (t, *J* = 6 Hz, 4H), 3.13 (t, *J* = 6 Hz, 4H).

#### 4.2.4 *N*-(4-Morpholinophenyl)-6-(3,4,5-trimethoxyphenyl)­imidazo­[1,2-*a*]­pyrazin-8-amine (**ED-4**)

Off white, yield 11.41%. ^1^H NMR (300 MHz, DMSO-*d*_6_) δ (ppm) 9.51 (s, 1H), 8.63 (s, 1H), 8.02 (d, *J* = 9 Hz, 2H), 7.95 (s, 1H), 7.63 (s, 1H), 7.35 (s, 2H), 6.97 (d, *J* = 9 Hz, 1H), 3.89 (s, 6H), 3.76 (t, *J* = 3 Hz, 4H), 3.70 (s, 3H), 3.08 (d, *J* = 6 Hz, 4H).

#### 4.2.5 6-(1H-Indol-5-yl)-*N*-(4-morpholinophenyl)­imidazo­[1,2-*a*]­pyrazin-8-amine (**ED-5**)

Off white, yield 26.68%. ^1^H NMR (300 MHz, DMSO-*d*_6_) δ (ppm) 11.1 (s, 1H), 9.39 (s, 1H), 8.48 (s, 1H), 8.18 (s, 1H), 8.07 (d, *J* = 9 Hz, 2H), 7.95 (s, 1H), 7.75 (m, 1H), 7.64 (m, 2H), 7.49 (d, *J* = 9 Hz, 1H), 7.38 (s, 1H), 7.01 (d, *J* = 9 Hz, 2H), 6.52 (s, 1H), 3.77 (t, *J* = 6 Hz, 4H), 3.11 (t, *J* = 6 Hz, 4H).

#### 4.2.6 6-(4-Methoxyphenyl)-*N*-(4-morpholinophenyl)­imidazo­[1,2-*a*]­pyrazin-8-amine (**ED-6**)

Brown powder, yield 15.22%. ^1^H NMR (300 MHz, DMSO-*d*_6_) δ (ppm) 9.43 (s, 1H), 8.47 (s, 1H), 8.01 (m, 5H), 7.70 (s, 1H), 7.06 (m, 4H), 3.81 (s, 3H), 3.77 (t, *J* = 6 Hz, 4H), 3.08 (s, 4H).

#### 4.2.7 *N*-(4-Morpholinophenyl)-6-(4-nitrophenyl)­imidazo­[1,2-*a*]­pyrazin-8-amine (**ED-7**)

Brown powder, yield 5.0%. ^1^H NMR (300 MHz, DMSO-*d*_6_) δ (ppm) 9.63 (s, 1H), 8.83 (s, 1H), 8.37 (d, *J* = 9 Hz, 2H), 8.27 (d, *J* = 9 Hz, 2H), 8.02 (m, 3H), 7.68 (d, *J* = 3 Hz, 1H), 7.02 (d, *J* = 12 Hz, 2H), 3.78 (t, *J* = 6 Hz, 4H), 3.11 (d, *J* = 3 Hz, 4H).

#### 4.2.8 6-(4-(*tert*-Butyl)­phenyl)-*N*-(4-morpholinophenyl)­imidazo­[1,2-*a*]­pyrazin-8-amine (**ED-8**)

Yellowish solid, yield 10.96%. ^1^H NMR (300 MHz, DMSO-*d*_6_) δ (ppm) 9.45 (s, 1H), 8.51 (s, 1H), 8.50 (m, 3H), 7.92 (m, 2H), 7.62 (d, *J* = 3 Hz, 1H), 7.52 (m, 2H), 6.99 (d, *J* = 9 Hz, 2H), 3.75 (d, *J* = 3 Hz, 4H), 3.10 (m, 4H), 1.32 (s, 9H).

#### 4.2.9 6-(4-Chlorophenyl)-*N*-(4-morpholinophenyl)­imidazo­[1,2-*a*]­pyrazin-8-amine (**ED-9**)

Brown powder, yield 14.81%. ^1^H NMR (300 MHz, DMSO-*d*_6_) δ (ppm) 9.51 (s, 1H), 8.61 (s, 1H), 8.01 (m, 5H), 7.63 (s, 1H), 7.56 (d, *J* = 9 Hz, 2H), 6.99 (d, *J* = 9 Hz, 2H), 3.77 (t, *J* = 6 Hz, 4H), 3.10 (t, *J* = 6 Hz, 4H).

#### 4.2.10 *N*-(4-Morpholinophenyl)-6-(pyrimidin-5-yl)­imidazo­[1,2-*a*]­pyrazin-8-amine (**ED-10**)

Yellowish solid, yield 17.58%. ^1^H NMR (300 MHz, DMSO-*d*_6_) δ (ppm) 9.65 (s, 1H), 9.33 (s, 1H), 9.18 (s, 1H), 8.77 (s, 1H), 8.00 (s, 1H), 7.95 (d, *J* = 9 Hz, 2H), 7.67 (s, 1H), 7.01 (d, *J* = 9 Hz, 2H), 3.74 (d, *J* = 3 Hz, 4H), 3.09 (d, *J* = 3 Hz, 4H).

#### 4.2.11 6-(3,5-Dimethoxyphenyl)-*N*-(4-morpholinophenyl)­imidazo­[1,2-*a*]­pyrazin-8-amine (**ED-11**)

Brown powder, yield 13.47%. ^1^H NMR (300 MHz, DMSO-*d*_6_) δ (ppm) 9.49 (s, 1H), 8.63 (s, 1H), 8.00 (t, *J* = 9 Hz, 3H), 7.63 (s, 1H), 7.20 (d, *J* = 3 Hz, 2H), 6.97 (d, *J* = 9 Hz, 2H), 6.52 (d, *J* = 3 Hz, 1H), 3.83 (s, 6H), 3.76 (t, *J* = 3 Hz, 4H), 3.09 (t, *J* = 6 Hz, 4H).

#### 4.2.12 4-(8-((4-Morpholinophenyl)­amino)­imidazo­[1,2-*a*]­pyrazin-6-yl)­phenol (**ED-12**)

Off white, yield 31.88%. ^1^H NMR (300 MHz, DMSO-*d*_6_) δ (ppm) 9.63 (s, 1H), 9.39 (s, 1H), 8.39 (s, 1H), 8.01 (d, *J* = 9 Hz, 2H), 7.92 (s, 1H), 7.82 (d, *J* = 9 Hz, 2H), 7.59 (s, 1H), 6.99 (d, *J* = 9 Hz, 2H), 6.88 (d, *J* = 9 Hz, 2H), 3.76 (t, *J* = 3 Hz, 4H), 3.09 (t, *J* = 3 Hz, 4H).

#### 4.2.13 6-(Furan-3-yl)-*N*-(4-morpholinophenyl)­imidazo­[1,2-*a*]­pyrazin-8-amine (**ED-13**)

Brown powder, yield 12.43%. ^1^H NMR (300 MHz, DMSO-*d*_6_) δ (ppm) 9.45 (s, 1H), 8.30 (s, 1H), 8.10 (s, 1H), 8.01 (d, *J* = 9 Hz, 2H), 7.92 (d, *J* = 3 Hz, 1H), 7.77 (d, *J* = 3 Hz, 1H), 7.60 (s, 1H), 6.99 (m, 3H), 3.76 (t, *J* = 3 Hz, 4H), 3.09 (t, *J* = 6 Hz, 4H).

#### 4.2.14 *N*-(4-Morpholinophenyl)-6-(thiophen-3-yl)­imidazo­[1,2-*a*]­pyrazin-8-amine (**ED-14**)

Brown powder, yield 14.42%. ^1^H NMR (300 MHz, DMSO-*d*_6_) δ (ppm) 9.48 (s, 1H), 8.48 (s, 1H), 8.03 (d, *J* = 9 Hz, 2H), 7.94 (d, *J* = 3 Hz, 2H), 7.67 (m, 3H), 7.00 (d, *J* = 9 Hz, 4H), 3.77 (t, *J* = 6 Hz, 4H), 3.10 (t, *J* = 6 Hz, 4H).

#### 4.2.15 6-(2,3-Dihydrobenzo­[*b*]­[1,4]­dioxin-6-yl)-*N*-(4-morpholinophenyl)­imidazo­[1,2-*a*]­pyrazin-8-amine (**ED-15**)

White powder, yield 44.97%. ^1^H NMR (300 MHz, DMSO-*d*_6_) δ (ppm) 9.45 (s, 1H), 8.47 (s,1H), 7.98 (d, *J* = 9 Hz, 2H), 7.92 (s, 1H), 7.60 (s, 1H), 7.48 (m, 3H), 4.29 (s, 4H), 3.76 (t, *J* = 3 Hz, 4H), 3.10 (d, *J* = 6 Hz, 4H).

#### 4.2.16 6-(1,3-Dimethyl-1*H*-pyrazol-5-yl)-*N*-(4-morpholinophenyl)­imidazo­[1,2-*a*]­pyrazin-8-amine (**ED-16**)

Off white, yield 14.90%. ^1^H NMR (300 MHz, DMSO-*d*_6_) δ (ppm) 9.53 (s, 1H), 8.25 (d, *J* = 3 Hz, 1H), 7.98 (d, *J* = 3 Hz, 1H), 7.84 (m, 2H), 7.65 (s, 1H), 6.95 (t, *J* = 6 Hz, 2H), 6.30 (d, *J* = 3 Hz, 1H), 3.92 (s, 3H), 3.75 (t, *J* = 3 Hz, 4H), 3.08 (t, *J* = 6 Hz, 4H), 2.17 (s, 3H).

#### 4.2.17 *N*-(4-Morpholinophenyl)-6-(pyridin-4-yl)­imidazo­[1,2-*a*]­pyrazin-8-amine (**ED-17**)

Yellowish solid, yield 39.19%. ^1^H NMR (300 MHz, DMSO-*d*_6_) δ (ppm) 9.59 (s, 1H), 8.82 (s, 1H), 8.67 (d, *J* = 6 Hz, 2H), 8.00 (m, 5H), 7.67 (s, 1H), 7.01 (d, *J* = 9 Hz, 2H), 3.77 (t, *J* = 3 Hz, 4H), 3.11 (t, *J* = 3 Hz, 4H).

#### 4.2.18 6-(2-Methylpyridin-4-yl)-*N*-(4-morpholinophenyl)­imidazo­[1,2-*a*]­pyrazin-8-amine (**ED-18**)

Yellowish solid, yield 37.86%. ^1^H NMR (300 MHz, DMSO-*d*_6_) δ (ppm) 9.58 (s, 1H), 8.79 (d, *J* = 3 Hz, 1H), 8.53 (d, *J* = 3 Hz, 1H), 7.99 (d, *J* = 6 Hz, 3H), 7.81 (s, 1H), 7.75 (d, *J* = 6 Hz, 1H), 7.67 (d, *J* = 3 Hz, 1H), 7.01 (d, *J* = 9 Hz, 2H), 3.76 (s, 4H), 3.11 (t, *J* = 6 Hz, 4H).

#### 4.2.19 6-(4-Fluorophenyl)-*N*-(4-morpholinophenyl)­imidazo­[1,2-*a*]­pyrazin-8-amine (**ED-19**)

Brown solid, yield 38.46%. ^1^H NMR (300 MHz, DMSO-*d*_6_) δ (ppm) 9.49 (s, 1H), 8.55 (s, 1H), 8.04 (m, 5H), 7.63 (d, *J* = 6 Hz, 1H), 7.35 (t, *J* = 9 Hz, 2H), 6.99 (d, *J* = 9 Hz, 2H), 3.75 (d, *J* = 3 Hz, 4H), 3.10 (d, *J* = 6 Hz, 4H).

#### 4.2.20 6-(6-Fluoropyridin-3-yl)-*N*-(4-morpholinophenyl)­imidazo­[1,2-*a*]­pyrazin-8-amine (**ED-20**)

Brown solid, yield 72.11%. ^1^H NMR (300 MHz, DMSO-*d*_6_) δ (ppm) 9.58 (s, 1H), 8.81 (s, 1H), 8.66 (s, 1H), 8.52 (m, 1H), 7.97 (t, *J* = 6 Hz, 3H), 7.65 (s, 1H), 7.33 (d, *J* = 6 Hz, 1H), 7.00 (d, *J* = 9 Hz, 2H), 3.76 (t, *J* = 3 Hz, 4H), 3.10 (t, *J* = 3 Hz, 4H).

#### 4.2.21 4-(8-((4-Morpholinophenyl)­amino)­imidazo­[1,2-*a*]­pyrazin-6-yl)­benzonitrile (**ED-21**)

Brown solid, yield 52.13%. ^1^H NMR (300 MHz, DMSO-*d*_6_) δ (ppm) 9.59 (s, 1H), 8.77 (s, 1H), 8.19 (d, *J* = 9 Hz, 1H), 7.99 (m, 5H), 7.66 (s, 1H), 7.00 (d, *J* = 9 Hz, 2H), 3.77 (t, *J* = 3 Hz, 4H), 3.11 (t, *J* = 6 Hz, 4H).

#### 4.2.22 6-(1*H*-Indol-4-yl)-*N*-(4-morpholinophenyl)­imidazo­[1,2-*a*]­pyrazin-8-amine (**ED-22**)

White solid, yield 16.43%. ^1^H NMR (300 MHz, DMSO-*d*_6_) δ (ppm) 11.27 (s, 1H), 9.40 (s, 1H), 8.46 (d, *J* = 3 Hz, 1H), 8.07 (d, *J* = 12 Hz, 2H), 7.62 (m, 5H), 7.56 (m, 2H), 7.46 (m, 1H), 6.93 (m, 2H), 3.73 (d, *J* = 3 Hz, 4H), 3.07 (d, *J* = 3 Hz, 4H).

#### 4.2.23 4-(8-((4-Morpholinophenyl)­amino)­imidazo­[1,2-*a*]­pyrazin-6-yl)­benzaldehyde (**ED-23**)

White solid, yield 61.97%. ^1^H NMR (300 MHz, DMSO-*d*_6_) δ (ppm) 10.05 (d, *J* = 3 Hz, 1H), 9.57 (s, 1H), 8.76 (s, 1H), 8.23 (m, 2H), 8.03 (m, 5H), 7.66 (d, *J* = 3 Hz, 1H), 7.01 (t, *J* = 3 Hz, 2H), 3.75 (d, *J* = 3 Hz, 4H), 3.10 (d, *J* = 3 Hz, 4H).

#### 4.2.24 *N*-(4-Morpholinophenyl)-6-(4-(trifluoromethyl)­phenyl)­imidazo­[1,2-*a*]­pyrazin-8-amine (**ED-24**)

Off white, yield 11.96%. ^1^H NMR (300 MHz, DMSO-*d*_6_) δ (ppm) 9.46 (s, 1H), 8.64 (s, 1H), 8.18 (d, *J* = 9 Hz, 2H), 7.98 (s, 1H), 7.93 (d, *J* = 9 Hz, 2H), 7.82 (d, *J* = 9 Hz, 2H), 7.63 (s, 1H), 6.98 (d, *J* = 9 Hz, 2H), 3.07 (d, *J* = 6 Hz, 4H).

#### 4.2.25 6-(3,4-Difluorophenyl)-*N*-(4-morpholinophenyl)­imidazo­[1,2-*a*]­pyrazin-8-amine (**ED-25**)

Brown solid, yield 33.17%. ^1^H NMR (300 MHz, DMSO-*d*_6_) δ (ppm) 9.54 (s, 1H), 8.62 (s, 1H), 8.00 (m, 4H), 7.94 (d, *J* = 9 Hz, 1H), 7.82 (s, 1H), 7.64 (m, 1H), 6.99 (d, *J* = 9 Hz, 2H), 3.76 (t, *J* = 3 Hz, 4H), 3.10 (t, *J* = 3 Hz, 4H).

#### 4.2.26 6-(3-Chloro-4-fluorophenyl)-*N*-(4-morpholinophenyl)­imidazo­[1,2-*a*]­pyrazin-8-amine (**ED-26**)

Brown solid, yield 63.71%. ^1^H NMR (300 MHz, DMSO-*d*_6_) δ (ppm) 9.55 (s, 1H), 8.65 (s, 1H), 8.15 (d, *J* = 6 Hz, 1H), 7.99 (m, 4H), 7.64 (s, 1H), 7.56 (t, *J* = 9 Hz, 1H), 6.98 (d, *J* = 6 Hz, 2H), 3.77 (t, *J* = 6 Hz, 4H), 3.10 (t, *J* = 6 Hz, 4H).

#### 4.2.27 6-(4-(Methylsulfonyl)­phenyl)-*N*-(4-morpholinophenyl)­imidazo­[1,2-*a*]­pyrazin-8-amine (**ED27**)

Brown solid, yield 43.75%. ^1^H NMR (300 MHz, DMSO-*d*_6_) δ (ppm) 9.59 (s, 1H), 8.75 (s, 1H), 8.24 (d, *J* = 6 Hz, 2H), 8.03 (m, 5H), 7.66 (s, 1H), 7.00 (d, *J* = 6 Hz, 2H), 3.76 (d, *J* = 3 Hz, 4H), 3.35 (s, 3H), 3.10 (s, 4H).

### 4.3 General Procedure for the Synthesis of Entospletinib Derivatives **ED28–30**

To the stirred solution of intermediate 21–23 (1 equiv), indazole-6-boronic acid (1–1.5 equiv), sodium carbonate (0.169 g, 1.60 mmol) in p-dioxane:water (9:1, 10 mL) was added, and the reaction mixture was allowed to stir at room temperature with nitrogen purging for about 15 min, and then Pd­(PPh_3_)_4_ (0.123 g, 0.10 mmol) was added to the reaction mixture and further allowed to stir for 3 h at 100 °C. After the completion of the reaction, cold water was added to the reaction mixture, and the resultant precipitates were filtered out and dried under vacuum. The crude product was further purified by using column chromatography using DCM:MeOH (9.5:0.5) as solvent to obtain ED-29. Furthermore, for the synthesis of ED28 and ED-30, initially, the same synthetic methodology was utilized as in ED-29, further followed by the addition of dry DCM (5 mL) and TFA (2.23 g, 19.50 mmol), dropwise at 0 °C in the reaction mixture, and it was allowed to stir at room temperature for 4 h. After the completion of the reaction, the reaction mixture was concentrated using a rotary evaporator, followed by the addition of diethyl ether to precipitates. The precipitates were further triturated by washing with diethyl ether to get the resultant compounds.

#### 4.3.1 6-(1H-Indazol-6-yl)-*N*-(4-(piperazin-1-yl)­phenyl)­imidazo­[1,2-*a*]­pyrazin-8-amine (**ED28**)

Yellowish solid, yield 67.90%. ^1^H NMR (300 MHz, DMSO-*d*_6_) δ (ppm) 9.66 (s, 1H) 8.89 (s, 1H) 8.73 (s, 1H), 8.20 (s,1H), 8.13 (s, 1H), 8.07 (m, 3H), 7.89 (d, *J* = 8.4 Hz 1H), 7.76 (d, *J* = 6.3 Hz, 1H), 7.57 (d, *J* = 9.0 Hz, 1H), 7.11 (d, *J* = 9 Hz, 2H), 3.35 (m, 8H), 1.24 (s, 1H).

#### 4.3.2 6-(1H-Indazol-6-yl)-*N*-phenylimidazo­[1,2-*a*]­pyrazin-8-amine (**ED-29**)

White solid, yield 19.70%. ^1^H NMR (300 MHz, DMSO-*d*_6_) δ (ppm) 13.20 (s, 1H), 9.69 (s, 1H), 8.73 (s, 1H), 8.19 (d, *J* = 6 Hz, 3H), 8.09 (s, 1H), 8.02 (s, 1H), 7.87 (d, *J* = 9 Hz, 1H), 7.74 (d, *J* = 6 Hz, 1H), 7.68 (d, *J* = 3 Hz, 1H), 7.43 (t, *J* = 9 Hz, 2H), 7.10 (t, *J* = 9 Hz, 1H).

#### 4.3.3 6-(1H-Indazol-6-yl)-8-(piperazin-1-yl)­imidazo­[1,2-*a*]­pyrazine (**ED-30**)

Off white, yield 44.82%. ^1^H NMR (300 MHz, DMSO-*d*_6_) δ (ppm) 13.18 (s, 1H), 9.03 (s, 1H), 8.76 (s, 1H), 8.16 (s, 1H), 8.09 (d, *J* = 3 Hz, 1H), 8.02 (d, *J* = 3 Hz, 1H), 7.84 (d, *J* = 9 Hz, 1H), 7.74 (m, 1H), 7.63 (s, 1H), 4.55 (d, *J* = 6 Hz, 4H).

#### 4.3.4 Methyl 8-(4-(4-((6-(1H-Indazol-7-yl) imidazo­[1,2-*a*]­pyrazin-8-yl) amino)­phenyl) piperazin-1-yl)-8-oxooctanoate (**24**)

To the stirred solution of the intermediate ED28 (0.200 g, 0.48 mmol) and suberic acid monomethyl ester (0.096 g, 0.73 mmol) in DMF (3 mL), HATU (0.370 g, 0.97 mmol), followed by DIPEA (0.126 g, 0.97 mmol) was added to the reaction mixture and allowed to stir at room temperature for 2 h. After the completion of the reaction, cold water was added to the reaction mixture, and the resultant precipitates were filtered out and dried under high vacuum. The crude product was further purified by column chromatography using MeOH:DCM (0.5:9.5) as solvent to obtain the intermediate 24. Light yellowish powder, yield 70.81%. ^1^H NMR (300 MHz, DMSO-*d*_6_) δ (ppm) 9.64 (s, 1H), 9.21 (s, 1H), 8.80 (s, 1H), 8.05 (s, 1H), 8.04 (m, 5H), 7.67 (s, 1H), 7.11 (d, *J* = 9.3 Hz, 2H), 3.64 (s, 4H), 3.58 (s, 3H), 3.12 (m, 4H), 2.33 (m, 4H), 2.02 (t, *J* = 6.3 Hz, 2H), 1.77 (t, *J* = 6.3 Hz, 2H), 1.53 (m, 4H).

#### 4.3.5 Methyl 4-(4-(4-((6-(1H-Indazol-7-yl) imidazo­[1,2-*a*]­pyrazin-8-yl)­amino) phenyl) piperazin-1-yl)-4-oxobutanoate (**25**)

Intermediate 25 was synthesized by using intermediate ED28 and succinic acid monomethyl ester, and the same synthetic methodology was utilized as for the synthesis of intermediate 24. Light green powder, yield 62.23%. ^1^H NMR (300 MHz, DMSO-*d*_6_) δ (ppm) 9.63 (s, 1H), 9.18 (s, 1H), 8.80 (s, 1H), 8.51 (s, 1H), 8.03 (m, 4H), 7.65 (d, *J* = 1.2 Hz, 1H), 7.08 (d, *J* = 9.3 Hz, 2H), 3.64 (s, 4H), 3.58 (s, 3H), 3.17 (m, 4H), 2.86 (d, *J* = 6 Hz, 2H), 2.68 (t, *J* = 4.8 Hz, 2H).

#### 4.3.6 Methyl 5-(4-(4-((6-(1H-Indazol-7-yl)­imidazo­[1,2-*a*]­pyrazin-8-yl)­amino)­phenyl)­piperazin-1-yl)-5-oxopentanoate (**26**)

Intermediate 26 was synthesized by using intermediate ED28 and methyl hydrogen glutarate, and the same synthetic methodology was utilized as for the synthesis of intermediate 24. Yellowish-green powder, yield 65.15%. ^1^H NMR (300 MHz, DMSO-*d*_6_) δ (ppm) 9.65 (s, 1H), 9.23 (s, 1H), 8.81 (s, 1H), 8.50 (s, 1H), 8.04 (m, 4H), 7.68 (d, *J* = 1.2 Hz, 1H), 7.11 (d, *J* = 9.0 Hz, 2H), 3.63 (s, 4H), 3.61 (s, 3H), 3.31 (m, 4H), 2.39 (t, *J* = 7.2 Hz, 2H), 2.06 (t, *J* = 7.5 Hz, 2H), 1.79 (m, 2H).

#### 4.3.7 Ethyl 6-(4-(4-((6-(1H-Indazol-7-yl) imidazo­[1,2-*a*]­pyrazin-8-yl)­amino) phenyl) piperazin-1-yl)-6-oxohexanoate (**27**)

Intermediate 27 was synthesized by using intermediate ED28 and monoethyl adipate, and the same synthetic methodology was utilized as for the synthesis of intermediate 24. Light yellowish powder, yield 66.11%. ^1^H NMR (300 MHz, DMSO-*d*_6_) δ (ppm) 9.62 (s, 1H), 9.18 (s, 1H), 8.78 (s, 1H), 8.48 (s, 1H), 8.08 (m, 4H), 7.65 (s, 1H), 7.08 (d, *J* = 9.3 Hz, 2H), 4.06 (m, 2H), 3.62 (s, 4H), 3.14 (m, 4H), 2.36 (m, 4H), 1.70 (m, 7H).

#### 4.3.8 Ethyl 7-(4-(4-((6-(1H-Indazol-7-yl) imidazo­[1,2-*a*]­pyrazin-8-yl) amino)­phenyl) piperazin-1-yl)-7-oxoheptanoate (**28**)

Intermediate 28 was synthesized by using intermediate ED28 and monoethyl pimelate, and the same synthetic methodology was utilized as for the synthesis of intermediate 24. Light yellowish powder, yield 68.56%. ^1^H NMR (300 MHz, CD_3_OD) δ (ppm) 9.10 (s, 1H), 8.34 (s, 1H), 8.12 (s, 1H), 7.08 (m, 5H), 7.53 (s, 1H), 7.11 (d, *J* = 9.0 Hz, 2H), 4.09 (m, 2H), 3.73 (m, 4H), 3.15 (m, 4H), 2.44 (t, *J* = 7.8 Hz, 2H), 2.34 (m, 4H), 1.80 (t, *J* = 7.5 Hz, 2H), 1.65 (m, 6H).

#### 4.3.9 Methyl 9-(4-(4-((6-(1H-Indazol-7-yl) imidazo­[1,2-*a*]­pyrazin-8-yl)­amino) phenyl) piperazin-1-yl)-9-oxononanoate (**29**)

Intermediate 29 was synthesized by using intermediate ED28 and monomethyl azelate, and the same synthetic methodology was utilized as for the synthesis of intermediate 24. Dark yellowish powder, yield 72.25%. ^1^H NMR (300 MHz, CD_3_OD) δ (ppm) 9.13 (s, 1H), 8.38 (s, 1H), 8.14 (s, 1H), 7.84 (m, 5H), 7.55 (s, 1H), 7.12 (d, *J* = 9.0 Hz, 2H), 3.72 (m, 4H), 3.62 (s, 3H), 3.14 (m, 4H), 2.41 (t, *J* = 7.2 Hz, 2H), 2.31 (m, 4H), 1.60 (m, 8H).

#### 4.3.10 Methyl 10-(4-(4-((6-(1H-Indazol-7-yl) imidazo­[1,2-*a*]­pyrazin-8-yl)­amino) phenyl)­piperazin-1-yl)-10-oxodecanoate (**30**)

Intermediate 30 was synthesized by using intermediate ED28 and methyl hydrogen sebacate, and the same synthetic methodology was utilized as for the synthesis of intermediate 24. Dark yellowish powder, yield 67.09%. ^1^H NMR (300 MHz, DMSO-*d*_6_) δ (ppm) 9.64 (s, 1H), 9.22 (s, 1H), 8.80 (s, 1H), 8.49 (s, 1H), 8.04 (m, 5H), 7.67 (s, 1H), 7.10 (d, *J* = 8.7 Hz, 2H), 3.58 (m, 4H), 3.57 (s, 3H), 3.19 (m, 4H), 2.31 (m, 6H), 1.80 (t, *J* = 6.9 Hz, 2H), 1.53 (m, 8H).

#### 4.3.11 Methyl 4-((4-(4-((6-(1H-Indazol-6-yl) imidazo­[1,2-*a*]­pyrazin-8-yl)­amino) phenyl)­piperazin-1-yl)­methyl)­benzoate (**31**)

To the stirred solution of the intermediate ED28 (0.600 g, 1.46 mmol) and methyl 4-bromomethylbenzoate (0.355 g, 1.46 mmol) in DMF (4 mL), K_2_CO_3_ (0.404 g, 2.92 mmol) was added to the reaction mixture and allowed to stir at room temperature for 2 h. After the completion of the reaction, cold water was added to the reaction mixture, and the product was extracted in ethyl acetate (40 mL × 3). The combined layer was washed with water and brine, dried over MgSO_4_, and concentrated under reduced pressure. The crude product was further purified by column chromatography using MeOH/DCM (1:9) as solvent to obtain the intermediate 31. White, yellowish powder, yield 64.31%. ^1^H NMR (300 MHz, DMSO-*d*_6_) δ (ppm) 13.17 (s, 1H), 9.49 (s, 1H), 8.65 (s, 1H), 8.18 (s, 1H), 8.08 (s, 1H), 7.96 (m, 3H), 7.84 (d, *J* = 8.4 Hz, 1H), 7.73 (s, 1H), 7.63 (d, *J* = 1.2 Hz, 1H), 7.52 (d, *J* = 8.4 Hz, 2H), 7.00 (d, *J* = 9.0 Hz, 2H), 4.32 (m, 2H), 3.63 (s, 3H), 3.15 (m, 4H), 2.56 (m, 4H).

#### 4.3.12 Methyl (*E*)-3-(4-((4-(4-((6-(1H-Indazol-6-yl)­imidazo­[1,2-*a*]­pyrazin-8-yl) amino) phenyl)­piperazin-1-yl)­methyl)­phenyl)­acrylate (**32**)

To the stirred solution of the intermediate ED28 (0.400 g, 0.97 mmol) and methyl 4-formylcinnamate (0.185 g, 0.97 mmol) in ethanol (3 mL), acetic acid (1 drop) was added and allowed to stir at room temperature for 10 min, followed by the addition of sodium cyanoborohydride (0.091 g, 1.46 mmol) at 0 °C for 4 h. After the completion of the reaction, cold water was added to the reaction mixture, and the product was extracted in ethyl acetate (20 mL x 3). The combined layer was washed with water and brine, dried over MgSO_4_, and concentrated under reduced pressure. The crude product was further purified by column chromatography using MeOH:DCM (1:9) as the solvent to obtain the intermediate 32. Light brown powder, yield 60.36%. ^1^H NMR (300 MHz, DMSO-*d*_6_) δ (ppm) 13.19 (s, 1H), 9.51 (s, 1H), 8.67 (s, 1H), 8.10 (m, 5H), 7.86 (d, *J* = 8.4 Hz, 1H), 7.71 (m, 5H), 7.43 (d, *J* = 7.5 Hz, 2H) 7.02 (d, *J* = 9.0 Hz, 2H), 6.67 (d, *J* = 16.2 Hz, 1H), 3.75 (s, 3H), 3.65 (s, 2H), 3.17 (s, 4H), 2.58 (s, 4H).

#### 4.3.13 *tert*-Butyl (4-((6-Bromoimidazo­[1,2-*a*]­pyrazin-8-yl)­amino)­phenyl)­carbamate (**33**)

To the stirred solution of *tert*-butyl­(4-aminophenyl)­carbamate (3.0 g, 14.40 mmol) and 6,8-dibromoimidazo­[1,2-*a*]­pyrazine (19) (5.18 g, 18.72 mmol) in isopropyl alcohol (25 mL), the DIPEA (2.70 g, 21.60 mmol) was added dropwise, and the reaction mixture was allowed to stir at 80 °C overnight. After the completion of the reaction, cold water was added to the reaction mixture, and the resultant precipitates were filtered out and dried under vacuum. The crude product was further purified by column chromatography using Hexane/EA (4:6) as solvent to obtain intermediate 33. Off-white powder, yield 95.6%. ^1^H NMR (300 MHz, DMSO-*d*_6_) δ (ppm) 9.86 (s,1H), 9.29 (s, 1H), 8.23 (d, *J* = 2.7 Hz, 1H), 7.95 (d, *J* = 3.9 Hz, 1H), 7.81 (m, 2H), 7.63 (d, *J* = 1.2 Hz, 1H), 7.43 (s, 2H), 1.49 (s, 9H).

#### 4.3.14 *tert*-Butyl (4-((6-(1H-Indazol-7-yl)­imidazo­[1,2-*a*]­pyrazin-8-yl)­amino)­phenyl)­carbamate (**34**)

To the stirred solution of intermediate 33 (2.0 g, 4.94 mmol), (1H-indazol-6-yl)­boronic acid (1.60 g, 9.89 mmol), and sodium carbonate (1.57 g, 14.84 mmol) in *p*-dioxane/water (9:1, 25 mL) were added, and the reaction mixture was allowed to stirred at room temperature with nitrogen purging for about 15 min, and Pd­(PPh_3_)_4_ (1.14 g, 0.98 mmol) was added to the reaction mixture and further allowed to stir for 3 h at 100 °C. After the completion of the reaction, cold water was added to the reaction mixture, and the resultant precipitates were filtered out and dried under vacuum. The crude product was further purified by using column chromatography using Hexane:EA (1:9) as solvent to obtain intermediate 34. Light yellowish powder, yield 82.58%. ^1^H NMR (300 MHz, CDCl_3_) δ (ppm) 8.49 (s, 1H), 8.18 (s, 1H), 7.71 (m, 4H), 7.62 (s, 1H), 7.50 (d, *J* = 1.2 Hz, 1H), 7.31 (d, *J* = 8.7 Hz, 2H), 6.45 (s, 1H), 1.46 (s, 9H).

#### 4.3.15 *N*1-(6-(1H-Indazol-7-yl)­imidazo­[1,2-*a*]­pyrazin-8-yl)­benzene-1,4-diamine (**35**)

To the stirred solution of intermediate 34 (1.40 g, 3.17 mmol) in dry DCM, TFA (1.8 g, 15.85 mmol) was added dropwise at 0 °C, and the reaction mixture was allowed to stir at room temperature for 4 h. After the completion of the reaction, the reaction mixture was concentrated using a rotary evaporator, followed by the addition of diethyl ether to precipitate. The precipitates were further purified by washing with diethyl ether to get the resultant intermediate 35. Light green powder, yield 76.32%. ^1^H NMR (300 MHz, DMSO-*d*_6_) δ (ppm) 13.20 (s, 1H), 9.22 (s, 1H), 8.60 (s, 1H), 8.15 (s, 1H), 8.07 (s, 1H), 7.95 (d, *J* = 1.2 Hz, 1H), 7.83 (d, *J* = 8.4 Hz, 1H), 7.71 (m, 3H), 7.60 (d, *J* = 1.2 Hz, 2H), 6.63 (d, *J* = 6.9 Hz, 2H).

#### 4.3.16 Methyl 4-((4-((6-(1H-Indazol-7-yl)­imidazo­[1,2-*a*]­pyrazin-8-yl)­amino)­phenyl)­amino)-4-oxobutanoate (**36**)

Intermediate 36 was synthesized by using intermediate 35 and succinic acid monomethyl ester, and the same synthetic methodology was utilized as for the synthesis of intermediate 24. Light yellowish powder, yield 60.21%. ^1^H NMR (300 MHz, DMSO-*d*_6_) δ (ppm) 13.25 (s, 1H), 9.96 (s, 1H), 9.70 (s, 1H), 9.06 (s, 1H), 8.81 (s, 1H), 8.54 (s, 1H), 8.07 (m, 4H) 7.69 (m, 1H), 7.63 (d, *J* = 9.0 Hz, 2H), 3.63 (s, 3H), 3.53 (t, *J* = 6.6 Hz, 2H), 2.87 (t, *J* = 6.0 Hz, 2H).

#### 4.3.17 Methyl 5-((4-((6-(1H-Indazol-7-yl) imidazo­[1,2-*a*]­pyrazin-8-yl)­amino)­phenyl)­amino)-5-oxopentanoate (**37**)

Intermediate 37 was synthesized by using intermediate 35 and methyl hydrogen glutarate, and the same synthetic methodology was utilized as for the synthesis of intermediate 24. Dark yellowish powder, yield 53.12%. ^1^H NMR (300 MHz, DMSO-*d*_6_) δ (ppm) 13.2 (s, 1H), 9.88 (s, 1H), 9.70 (s, 1H), 9.11 (s, 1H), 8.81 (s, 1H), 8.51 (m, 1H), 8.17 (d, *J* = 8.7 Hz, 2H), 8.05 (m, 3H), 7.69 (s, 1H), 7.66 (d, *J* = 8.7 Hz, 2H), 3.62 (s, 3H), 2.41 (t, *J* = 7.8 Hz, 2H), 2.07 (t, *J* = 7.2 Hz, 2H), 1.90 (m, 2H).

#### 4.3.18 Methyl 6-((4-((6-(1H-Indazol-7-yl)­imidazo­[1,2-*a*]­pyrazin-8-yl)­amino)­phenyl)­amino)-6-oxohexanoate (**38**)

Intermediate 38 was synthesized by using intermediate 35 and monomethyl adipate, and the same synthetic methodology was utilized as for the synthesis of intermediate 24. Dark yellowish powder, yield 67.23%. ^1^H NMR (300 MHz, DMSO-*d*_6_) δ (ppm) 13.20 (s, 1H), 9.86 (s, 1H), 9.70 (s, 1H), 9.12 (s, 1H), 8.81 (s, 1H), 8.51 (s, 1H), 8.17 (d, *J* = 8.7 Hz, 2H), 8.06 (m, 3H), 7.69 (s, 1H), 7.66 (m, 2H), 4.07 (m, 3H), 3.25 (t, *J* = 7.2 Hz, 2H), 2.40 (t, *J* = 7.2 Hz, 2H), 1.68 (m, 4H).

#### 4.3.19 Ethyl 7-((4-((6-(1H-Indazol-7-yl)­imidazo­[1,2-*a*]­pyrazin-8-yl)­amino)­phenyl)­amino)-7-oxoheptanoate (**39**)

Intermediate 39 was synthesized by using intermediate 35 and monoethyl pimelate, and the same synthetic methodology was utilized as for the synthesis of intermediate 24. Light yellowish powder, yield 58.10%. ^1^H NMR (300 MHz, DMSO-*d*_6_) δ (ppm) 13.21 (s, 1H), 9.81 (s, 1H), 9.60 (s, 1H), 8.69 (s, 1H), 8.17 (s, 1H), 8.08 (m, 3H), 8.06 (s, 1H), 7.86 (d, *J* = 8.7 Hz, 1H), 7.73 (d, *J* = 8.7 Hz, 1H), 7.65 (m, 3H), 4.02 (m, 2H) 3.56 (s, 3H), 2.29 (s, 2H), 1.56 (m, 4H), 1.30 (m, 4H).

#### 4.3.20 Methyl 8-((4-((6-(1H-Indazol-7-yl)­imidazo­[1,2-*a*]­pyrazin-8-yl)­amino)­phenyl)­amino)-8-oxooctanoate (**40**)

Intermediate 40 was synthesized by using intermediate 35 and suberic acid monomethyl ester, and the same synthetic methodology was utilized as for the synthesis of intermediate 24. Light yellowish powder, yield 68.18%. ^1^H NMR (300 MHz, DMSO-*d*_6_) δ (ppm) 13.22 (s, 1H), 9.82 (s, 1H), 9.61 (s, 1H), 8.70 (s, 1H), 8.17, (s, 1H), 8.08 (m, 3H), 8.06 (s, 1H), 7.86 (d, *J* = 8.4 Hz, 1H), 7.74 (d, *J* = 8.4 Hz, 1H), 7.65 (m, 3H), 3.57 (s, 3H), 2.29 (s, 2H), 1.56 (m, 4H), 1.30 (m, 6H).

#### 4.3.21 Methyl 9-((4-((6-(1H-Indazol-7-yl)­imidazo­[1,2-*a*]­pyrazin-8-yl)­amino)­phenyl)­amino)-9-oxononanoate (**41**)

Intermediate 41 was synthesized by using intermediate 35 and monomethyl azelate, and the same synthetic methodology was utilized as for the synthesis of intermediate 24. Dark yellowish powder, yield 52.23%. ^1^H NMR (300 MHz, DMSO-*d*_6_) δ (ppm) 13.21 (s, 1H), 9.26 (s, 1H), 8.59 (s, 1H), 8.15 (s, 1H), 8.09 (s, 1H), 7.97 (d, *J* = 1.2 Hz, 1H), 7.81 (m, 3H), 7.71 (s, 1H), 7.62 (m, 1H), 7.46 (d, *J* = 8.1 Hz, 2H), 3.57 (s, 3H), 2.30 (s, 2H), 1.56 (m, 6H), 1.30 (m, 6H).

#### 4.3.22 Methyl 10-((4-((6-(1H-Indazol-7-yl) imidazo­[1,2-*a*]­pyrazin-8-yl)­amino) phenyl)­amino)-10-oxodecanoate (**42**)

Intermediate 42 was synthesized by using intermediate 35 and methyl hydrogen sebacate, and the same synthetic methodology was utilized as for the synthesis of intermediate 24. Dark yellowish powder, yield 53.29%. ^1^H NMR (300 MHz, DMSO-*d*_6_) δ (ppm) 13.92 (s, 1H), 9.83 (s, 1H), 9.69 (s, 1H), 9.13 (s, 1H), 8.81 (s, 1H), 8.53 (m, 3H), 8.17 (m, 4H), 7.69 (s, 1H), 7.66 (d, *J* = 9.0 Hz, 2H), 3.58 (s, 3H), 2.28 (m, 7H), 1.81 (m, 2H), 1.59 (m, 7H).

#### 4.3.23 Methyl 4-(((4-((6-(1H-Indazol-6-yl)­imidazo­[1,2-*a*]­pyrazin-8-yl)­amino)­phenyl)­amino)­methyl)­benzoate (**43**)

Intermediate 43 was synthesized by using intermediate 35 and the same synthetic methodology as utilized for the synthesis of intermediate 31. Dark yellowish powder, yield 49.23%. ^1^H NMR (300 MHz, DMSO-*d*_6_) δ (ppm) 13.19 (s, 1H), 9.28 (s, 1H), 8.61 (s, 1H), 8.15 (s, 1H), 8.15 (d, *J* = 16.8 Hz, 1H), 7.96 (m, 3H), 7.83 (m, 3H), 7.72 (d, *J* = 8.7 Hz, 1H), 7.62 (s, 1H), 7.56 (d, *J* = 8.1 Hz, 2H), 6.65 (d, *J* = 8.7 Hz, 2H), 6.24 (t, *J* = 6.3 Hz, 1H), 4.42 (d, *J* = 5.7 Hz, 2H), 3.84 (s, 3H).

#### 4.3.24 Methyl (*E*)-3-(4-(((4-((6-(1H-Indazol-6-yl) imidazo­[1,2-*a*]­pyrazin-8-yl)­amino) phenyl)­amino) methyl) phenyl)­acrylate (**44**)

Intermediate 44 was synthesized by using intermediate 35 and the same synthetic methodology as utilized for the synthesis of intermediate 32. Dark brown powder, yield 46.32%. ^1^H NMR (300 MHz, DMSO-*d*_6_) δ (ppm) 13.18 (s, 1H), 9.26 (s, 1H), 8.59 (s, 1H), 8.13 (d, *J* = 18.6 Hz, 1H) 7.95 (s, 1H), 7.81 (m, 3H) 7.70 (m, 3H), 7.60 (m, 2H), 7.45 (d, *J* = 8.1 Hz, 2H), 6.62 (d, *J* = 3.3 Hz, 1H), 6.17 (s, 1H), 4.32 (s, 2H), 3.71 (s, 3H).

#### 4.3.25 8-(4-(4-((6-(1H-Indazol-6-yl)­imidazo­[1,2-*a*]­pyrazin-8-yl)­amino)­phenyl)­piperazin-1-yl)-*N*-hydroxy-8-oxooctanamide (**1**)

To the stirred solution of intermediate 24 (0.100 g, 0.19 mmol) in MeOH (3 mL), DBU (0.087 g, 0.57 mmol) was added dropwise to the reaction mixture at 0 °C and stirred for 30 min, followed by the addition of Hydroxylamine (0.063 g, 1.90 mmol). The reaction mixture was further stirred for 2 h. After the completion of the reaction, the reaction mixture was diluted with water, and the pH was adjusted to 7 using 3 N HCl. The resultant precipitates were filtered out from the reaction mixture and dried under vacuum, and further purified by column chromatography using MeOH:DCM (1:9) as solvent to obtain compound **1**. Off-white powder, yield 67.90%. HPLC purity: 96.217% (tR: 22.467 min); mp: 240–243 °C, ^1^H NMR (600 MHz, DMSO-*d*_6_) δ (ppm) 13.19 (s, 1H), 10.33 (s, 1H), 9.51 (s, 1H), 8.66 (s, 2H), 8.19 (s, 1H), 8.08 (s, 1H), 8.04 (d, *J* = 9.0 Hz, 2H), 7.99 (s, 1H), 7.84 (d, *J* = 9.0 Hz, 1H), 7.72 (d, *J* = 8.4 Hz, 1H), 7.63 (s, 1H), 7.02 (d, *J* = 8.4 Hz, 2H), 3.61 (s, 4H), 3.12 (d, *J* = 30 Hz, 4H), 2.35 (t, *J* = 7.8 Hz, 2H), 1.95 (t, *J* = 7.8 Hz, 2H), 1.50 (m, 4H), 1.28 (m, 4H). ^13^C (150 MHz, DMSO-*d*_6_) δ (ppm) 171.10, 169.57, 146.77, 145.27, 140.95, 136.91, 135.61, 133.87, 133.19, 132.73, 132.58, 121.59, 121.08, 118.75, 116.88, 116.77, 109.15, 107.77, 50.05, 49.58, 45.32, 41.36, 32.69, 32.65, 28.93, 28.88, 25.47, 25.14. HRMS (ESI) for C_31_H_36_N_9_O_3_ (M + H^+^): calcd 582.2941; found 582.2952.

#### 4.3.26 4-(4-(4-((6-(1H-Indazol-6-yl)­imidazo­[1,2-*a*]­pyrazin-8-yl)­amino)­phenyl)­piperazin-1-yl)-*N*-hydroxy-4-oxobutanamide (**2**)

Compound **2** was synthesized using the intermediate 25 and the same reaction conditions as followed in compound 1. Yellowish-white powder, yield 60.23%, HPLC purity: 95.063% (tR: 20.162 min); mp: 216–220 °C, ^1^H NMR (300 MHz, DMSO-*d*_6_) δ (ppm) 10.42 (s, 1H), 9.63 (s, 1H), 8.73 (s, 2H), 8.21 (s, 1H), 8.12 (s, 1H), 8.06 (d, *J* = 5.1 Hz, 3H), 7.88 (d, *J* = 8.4 Hz, 1H), 7.75 (t, *J* = 6.3 Hz, 2H), 7.12 (d, *J* = 8.7 Hz, 2H), 3.65 (s, 4H), 3.21 (d, *J* = 22.2 Hz, 4H), 2.63 (t, *J* = 7.2 Hz, 2H), 2.26 (t, *J* = 6.9 Hz, 2H). ^13^C (150 MHz, DMSO-*d*_6_) δ (ppm) 173.22, 170.20, 145.89, 144.94, 144.98, 140.98, 137.39, 135.36, 133.65, 132.11, 132.092, 123.084, 121.56, 121.18, 118.77, 117.06, 117.01, 109.21, 107.73, 46.70, 43.33, 28.06, 27.95, 25.64. HRMS (ESI) for C_27_H_28_N_9_O_3_ (M + H^+^): calcd 526.2315; found 526.2300.

#### 4.3.27 5-(4-(4-((6-(1H-Indazol-6-yl)­imidazo­[1,2-*a*]­pyrazin-8-yl)­amino)­phenyl)­piperazin-1-yl)-*N*-hydroxy-5-oxopentanamide (**3**)

Compound **3** was synthesized using the intermediate 26 and the same reaction conditions as followed in compound 1. Off-white powder, yield 66.32%, HPLC purity: 96.672% (tR: 18.989 min); mp: 237–240 °C, ^1^H NMR (600 MHz, DMSO-*d*_6_) δ (ppm) 13.18 (s, 1H), 10.36 (s, 1H), 9.51 (s, 1H), 8.66 (s, 2H), 8.18 (s, 1H), 8.08 (s, 1H), 8.04 (d, *J* = 9.0 Hz, 2H), 7.98 (s, 1H), 7.84 (d, *J* = 9.0 Hz, 1H), 7.73 (d, *J* = 9.0 Hz, 1H), 7.63 (s, 1H), 7.03 (d, *J* = 9.6 Hz, 2H), 3.63 (t, *J* = 4.8 Hz, 4H), 3.09 (t, *J* = 4.8 Hz, 4H), 2.37 (t, *J* = 7.2 Hz, 2H), 2.03 (t, *J* = 7.8 Hz, 2H), 1.77 (m, 2H). ^13^C (150 MHz, DMSO-*d*_6_) δ (ppm) 170.66, 169.35, 146.77, 145.27, 140.95, 136.91, 135.62, 133.89, 133.19, 132.74, 132.58, 123.02, 121.59, 121.08, 118.75, 116.88, 116.77, 109.15, 107.56, 49.981, 49.56, 49.03,45.26, 41.39, 32.05, 21.34. HRMS (ESI) C_28_H_30_N_9_O_3_ for (M + H^+^): calcd 540.2471; found 540.2487.

#### 4.3.28 6-(4-(4-((6-(1H-Indazol-6-yl)­imidazo­[1,2-*a*]­pyrazin-8-yl)­amino)­phenyl)­piperazin-1-yl)-*N*-hydroxy-6-oxohexanamide (**4**)

Compound **4** was synthesized using the intermediate 27 and the same reaction conditions as followed in compound 1. White powder, yield 64.62%, HPLC purity: 95.235% (tR: 20.968 min); mp: 236–239 °C, ^1^H NMR (600 MHz, DMSO-*d*_6_) δ (ppm) 13.18 (s, 1H), 10.35 (s, 1H), 9.51 (s, 1H), 8.66 (s, 2H), 8.18 (s, 1H), 8.08 (s, 1H), 8.04 (d, *J* = 9.0 Hz, 2H), 7.98 (s, 1H), 7.84 (d, *J* = 8.4 Hz, 1H), 7.72 (d, *J* = 8.4 Hz, 1H), 7.63 (s, 1H), 7.03 (d, *J* = 9.0 Hz, 2H), 3.61 (s, 4H), 3.13 (d, *J* = 31.8 Hz, 4H), 2.37 (t, *J* = 7.2 Hz, 2H), 1.98 (t, *J* = 7.2 Hz, 2H), 1.53 (m, 4H). ^13^C (150 MHz, DMSO-*d*_6_) δ (ppm) 170.97, 169.43, 146.78, 145.27, 140.95, 136.91, 135.61, 133.89, 133.19, 132.74, 132.58, 123.01, 121.59, 121.08, 118.75, 116.88, 116.77, 109.15, 107.56, 50.04, 49.57, 45.32, 41.38, 32.57, 32.39, 25.33, 24.81. HRMS (ESI) for C_29_H_32_N_9_O_3_ (M + H^+^): calcd 554.2628; found 554.2622.

#### 4.3.29 7-(4-(4-((6-(1H-Indazol-6-yl)­imidazo­[1,2-*a*]­pyrazin-8-yl)­amino)­phenyl)­piperazin-1-yl)-*N*-hydroxy-7-oxoheptanamide (**5**)

Compound **5** was synthesized using the intermediate 28 and the same reaction conditions as followed in compound 1. Off-white powder, yield 61.68%. HPLC purity: 97.031% (tR: 21.481 min); mp: 239–242 °C, ^1^H NMR (600 MHz, DMSO-*d*_6_) δ (ppm) 13.18 (s, 1H), 10.32 (s, 1H), 9.51 (s, 1H), 8.65 (s, 2H), 8.19 (s, 1H), 8.09 (s, 1H), 8.04 (d, *J* = 9.0 Hz, 2H), 7.98 (s, 1H), 7.84 (d, *J* = 9.0 Hz, 1H), 7.73 (d, *J* = 8.4 Hz, 1H), 7.63 (s, 1H), 7.02 (d, *J* = 9.0 Hz, 2H), 3.61 (m, 4H), 3.12 (t, *J* = 25.8 Hz, 4H), 2.35 (t, *J* = 7.2 Hz, 2H), 1.96 (t, *J* = 7.2 Hz, 2H), 1.53 (m, 4H), 1.29 (m, 2H). ^13^C (150 MHz, DMSO-*d*_6_) δ (ppm) 171.07, 169.52, 146.78, 145.26, 136.91, 135.61, 133.17, 132.74, 132.57, 123.01, 121.59, 121.09, 118.75, 116.88, 116.77, 109.14, 107.56, 50.03, 49.57, 45.31, 41.36, 32.65, 32.58, 28.81, 25.44, 24.96. HRMS (ESI) for C_30_H_34_N_9_O_3_ (M + H^+^): calcd 568.2784; found 568.2793.

#### 4.3.30 9-(4-(4-((6-(1H-Indazol-6-yl)­imidazo­[1,2-*a*]­pyrazin-8-yl)­amino)­phenyl)­piperazin-1-yl)-*N*-hydroxy-9-oxononanamide (**6**)

Compound **6** was synthesized using the intermediate 29 and the same reaction conditions as followed in compound 1. Off-white powder, yield 66.03%. HPLC purity: 97.536% (tR: 23.629 min); mp: 242–245 °C, ^1^H NMR (600 MHz, DMSO-*d*_6_) δ (ppm) 13.18 (s, 1H), 10.32 (s, 1H), 9.51 (s, 1H), 8.66 (s, 2H), 8.19 (s, 1H), 8.08 (s, 1H), 8.04 (d, *J* = 9.0 Hz, 2H), 7.99 (s, 1H), 7.84 (d, *J* = 9.0 Hz, 1H), 7.73 (d, *J* = 8.4 Hz, 1H), 7.64 (s, 1H), 7.02 (d, *J* = 9.0 Hz, 2H), 3.61 (s, 4H), 3.12 (d, *J* = 29.4 Hz, 4H), 2.23 (t, *J* = 7.8 Hz, 2H), 1.95 (t, *J* = 7.8 Hz, 2H), 1.49 (m, 4H), 1.24 (m, 6H). ^13^C (150 MHz, DMSO-*d*_6_) δ (ppm) 171.13, 169.57, 146.77, 145.26, 136.92, 135.61, 133.88, 133.19, 132.71, 132.57, 123.02, 121.59, 121.08, 118.75, 116.77, 109.15, 107.55, 50.05, 49.59, 45.32, 41.35, 32.68, 29.12, 29.01, 28.92, 25.52, 25.21. HRMS (ESI) for C_32_H_38_N_9_O_3_ (M + H^+^): calcd 596.3097; found 596.3093.

#### 4.3.31 10-(4-(4-((6-(1H-Indazol-6-yl)­imidazo­[1,2-*a*]­pyrazin-8-yl)­amino)­phenyl)­piperazin-1-yl)-*N*-hydroxy-10-oxodecanamide (**7**)

Compound **7** was synthesized using the intermediate 30 and the same reaction conditions as followed in compound 1. White powder, yield 70.23%. HPLC purity: 98.153% (tR: 24.871 min); mp: 210–213 °C, ^1^H NMR (600 MHz, DMSO-*d*_6_) δ (ppm) 13.18 (s, 1H), 10.31 (s, 1H), 9.51 (s, 1H), 8.66 (s, 2H), 8.19 (s, 1H), 8.08 (s, 1H), 8.04 (d, *J* = 9.0 Hz, 2H), 7.98 (s, 1H), 7.84 (d, *J* = 9.0 Hz, 1H), 7.73 (d, *J* = 9.3 Hz, 1H), 7.63 (s, 1H), 7.02 (d, *J* = 9.0 Hz, 2H), 3.61 (s, 4H), 3.12 (d, *J* = 29.4 Hz, 4H), 2.35 (t, *J* = 7.2 Hz, 2H), 1.94 (t, *J* = 7.2 Hz, 2H), 1.51 (m, 4H), 1.26 (m, 8H). ^13^C (150 MHz, DMSO-*d*_6_) δ (ppm) 171.13, 169.56, 146.77, 145.27, 140.94, 136.91, 135.62, 133.88, 133.19, 132.74, 132.58, 123.02, 121.59, 121.08, 118.75, 116.88, 116.76, 109.14, 107.55, 50.04, 59.58, 45.32, 41.36, 32.70, 29.23, 29.21, 29.11, 28.99, 25.54, 25.25. HRMS (ESI) for C_33_H_40_N_9_O_3_ (M + H^+^): calcd 610.3254; found 610.3245.

#### 4.3.32 4-((4-(4-((6-(1H-Indazol-6-yl)­imidazo­[1,2-*a*]­pyrazin-8-yl)­amino)­phenyl)­piperazin-1-yl)­methyl)-*N*-hydroxybenzamide (**8**)

To the stirred solution of intermediate 31 (0.200 g, 0.35 mmol) in *p*-dioxane (5 mL), 1 M LiOH_(aq)_ (0.017g, 1.79 mmol) was added dropwise, and the reaction mixture was stirred at room temperature for 3 h. After the completion of the reaction, the pH of the reaction mixture was adjusted to 5 by using a 1 N HCl solution. The resultant precipitates were filtered out and dried under high vacuum. In an RBF, the collected precipitates (0.125 g, 0.22 mmol), *O*-(tetrahydro-2*H*-pyran-2-yl)­hydroxylamine (0.026 g, 0.22 mmol), EDC·HCl (0.071 g, 0.45 mmol), and HOBt (0.046 g, 0.34 mmol) were dissolved in DMF, followed by the addition of DIPEA (0.074 g, 0.57 mmol), and the reaction mixture was allowed to stir at room temperature for 3 h. After the completion of the reaction, cold water was added to the reaction mixture, and the resultant precipitates were filtered out and dried under vacuum. The crude product was further purified by column chromatography using MeOH:DCM (1:9) as solvent. In an RBF, the purified solid (0.075 g, 0.11 mmol) was dissolved in MeOH (3 mL), and TFA (10% aq) (0.066 g, 0.058 mmol) was added dropwise, and the reaction mixture was stirred at room temperature for 3 h. After the completion of the reaction, the pH was adjusted to 7. The resultant precipitates were filtered out and dried under high vacuum. Yellowish color powder, yield 59.62%. HPLC purity: 98.286% (tR: 18.143 min); mp: 230–233 °C, ^1^H NMR (300 MHz, DMSO-*d*_6_) δ (ppm) 13.20 (s, 1H), 11.18 (s, 1H), 9.47 (s, 1H), 9.00 (s, 1H), 8.65 (s, 1H), 8.18 (s, 1H), 8.07 (s, 1H), 8.01 (m, 3H), 7.83 (d, *J* = 8.4 Hz, 1H), 7.74 (m, 3H), 7.63 (s, 1H), 7.42 (d, *J* = 7.8 Hz, 2H), 6.99 (d, *J* = 9.0 Hz, 2H), 3.58 (s, 2H), 3.17 (t, *J* = 17.4 Hz, 4H), 2.55 (s, 4H). ^13^C (150 MHz, DMSO-*d*_6_) δ (ppm) 164.60, 158.36, 158.15, 147.02, 145.28, 140.95, 136.91, 135.62, 133.86, 132.71, 132.67, 132.58, 129.17, 127.29, 123.00, 121.60, 121.06, 118.72, 116.86, 116.73, 116.18, 109.06, 107.56, 62.04, 53.10, 49.35. HRMS (ESI) for C_31_H_30_N_9_O_2_ (M + H^+^): calcd 560.2522; found 560.2518.

#### 4.3.33 (*E*)-3-(4-((4-(4-((6-(1H-Indazol-6-yl)­imidazo­[1,2-*a*]­pyrazin-8-yl)­amino)­phenyl)­piperazin-1-yl)­methyl)­phenyl)-*N*-hydroxyacrylamide (**9**)

Compound **9** was synthesized using the intermediate 32, and the same synthetic methodology was utilized as followed in compound 8. Light brown powder, yield 62.30%, HPLC purity: 96.347% (tR: 18.989 min); mp: 228–230 °C, ^1^H NMR (600 MHz, DMSO-*d*_6_) δ (ppm) 13.19 (s, 1H), 10.77 (s, 1H), 9.47 (s, 1H), 9.03 (s, 1H), 8.65 (s, 1H), 8.18 (s, 1H), 8.08 (s, 1H), 8.01 (s, 1H), 7.99 (m, 3H), 7.83 (d, *J* = 9.0 Hz, 1H), 7.72 (m, 1H), 7.63 (s, 1H), 7.54 (d, *J* = 7.2 Hz, 2H), 7.47 (d, *J* = 15.6 Hz, 1H), 7.39 (d, *J* = 3.6 Hz, 2H), 6.99 (d, *J* = 9.0 Hz, 2H), 6.48 (d, *J* = 15.6 Hz, 1H), 3.56 (s, 1H), 3.14 (s, 4H), 2.55 (s, 4H). ^13^C (150 MHz, DMSO-*d*_6_) δ (ppm) 163.24, 158.37, 158.17, 145.28, 140.94, 138.55, 136.92, 135.62, 133.87, 132.71, 132.58, 129.86, 127.86, 123.00, 121.601, 118.73, 1163.87, 116.74, 116.19, 109.07, 107.56, 53.07, 49.34. HRMS (ESI) for C_33_H_32_N_9_O_2_ (M + H^+^): calcd 586.2679; found 586.2681.

#### 4.3.34 *N*1-(4-((6-(1H-Indazol-6-yl)­imidazo­[1,2-*a*]­pyrazin-8-yl)­amino)­phenyl)-*N*4-hydroxysuccinamide (**10**)

To the stirred solution of intermediate 36 (0.100 g, 0.21 mmol) in MeOH (3 mL), DBU (0.100 g, 0.65 mmol) was added dropwise to the reaction mixture at 0 °C and stirred for 30 min, followed by the addition of Hydroxylamine (0.072 g, 2.19 mmol). The reaction mixture was further stirred for 2 h. After the completion of the reaction, the pH was adjusted to 7 using 3 N HCl. The resultant precipitates were filtered out and washed with methanol 3 times to obtain compound **10**. Off-white powder, yield 59.24%, HPLC purity: 98.584% (tR: 19.536 min); mp: 218–220 °C, ^1^H NMR (300 MHz, DMSO-*d*_6_) δ (ppm) 13.23 (s, 1H), 10.44 (s, 1H), 9.94 (s, 1H), 9.61 (s, 1H), 8.70 (s, 2H), 8.17 (s, 1H), 8.08 (d, *J* = 7.5 Hz, 3H), 8.00 (s, 1H), 7.86 (d, *J* = 9.0 Hz, 1H), 7.73 (d, *J* = 8.7 Hz, 1H), 7.62 (d, *J* = 8.7 Hz, 2H), 2.57 (t, *J* = 6.9 Hz, 2H), 2.30 (t, *J* = 7.8 Hz, 2H). ^13^C (150 MHz, DMSO-*d*_6_) δ (ppm) 170.54, 169.15, 145.49, 141.26, 137.16, 136.14, 135.82, 134.72, 134.13, 133.13, 132.84, 123.33, 121.43, 120.99, 120.03, 119.03, 117.21, 109.83, 107.81, 32.17, 28.26. HRMS (ESI) for C_23_H_21_N_8_O_3_ (M + H^+^): calcd 457.1736; found 457.1742.

#### 4.3.35 *N*1-(4-((6-(1H-Indazol-6-yl)­imidazo­[1,2-*a*]­pyrazin-8-yl)­amino)­phenyl)-*N*5-hydroxyglutaramide (**11**)

Compound **11** was synthesized using the intermediate 37 and the same synthetic methodology as followed in compound 10. Off-white powder, yield 66.62%. HPLC purity: 95.007% (tR: 19.391 min); mp: 238–239 °C, ^1^H NMR (300 MHz, DMSO-*d*_6_) δ (ppm) 13.26 (s, 1H), 9.91 (s, 1H), 9.67 (s, 1H), 8.72 (s, 1H), 8.20 (s, 1H), 8.12 (m, 3H), 8.02 (s,1H), 7.88 (d, *J* = 8.4 Hz, 1H), 7.75 (d, *J* = 8.7 Hz, 1H), 7.68 (m, 3H), 2.51 (m, 4H), 1.88 (t, *J* = 7.5 Hz, 2H). ^13^C (150 MHz, DMSO-*d*_6_) δ (ppm) 173.52, 170.66, 145.21, 140.97, 136.88, 135.93, 135.54, 134.34, 133.87, 132.85, 132.56, 123.06, 121.14, 120.68, 119.91, 118.76, 116.94, 109.55, 107.52, 51.71, 35.63, 33.13, 20.93. HRMS (ESI) C_24_H_23_N_8_O_3_ for (M + H^+^): calcd 471.1893; found 471.1981.

#### 4.3.36 *N*1-(4-((6-(1H-Indazol-6-yl) imidazo­[1,2-*a*] pyrazin-8-yl)­amino) phenyl)-*N*6-hydroxyadipamide (**12)**

Compound **12** was synthesized using the intermediate 38 and the same synthetic methodology as followed in compound 10. Off-white powder, yield 65.62%. HPLC purity: 95.726% (tR: 23.262 min); mp: 230–233 °C, ^1^H NMR (300 MHz, DMSO-*d*_6_) δ (ppm) 13.27 (s, 1H), 10.41 (s, 1H), 9.89 (s, 1H), 9.66 (s, 1H), 8.72 (s, 2H), 8.20 (s, 1H), 8.12 (m, 3H), 8.09 (s, 1H), 7.88 (d, *J* = 8.4 Hz, 1H), 7.75 (d, *J* = 8.4 Hz, 1H), 7.67 (m, 3H), 2.35 (m, 2H), 2.01 (t, *J* = 6.3 Hz, 2H), 1.59 (m, 4H). ^13^C (150 MHz, DMSO-*d*_6_) δ (ppm) 171.22, 169.48, 145.18, 136.89, 135.88, 135.53, 134.41, 132.85, 132.53, 132.11, 123.04, 121.17, 120.65, 119.93, 118.77, 116.97, 109.55, 107.55, 36.58, 33.74, 32.62, 25.34. HRMS (ESI) for C_22_H_25_N_8_O_3_ (M + H^+^): calcd 485.2049; found 485.2065.

#### 4.3.37 *N*1-(4-((6-(1H-Indazol-6-yl)­imidazo­[1,2-*a*]­pyrazin-8-yl)­amino)­phenyl)-*N*7-hydroxyheptanediamide (**13**)

Compound **13** was synthesized using the intermediate 39 and the same synthetic methodology as followed in compound 10. Off-white powder, yield 62.62%, HPLC purity: 95.922% (tR: 22.314 min); mp: 234–237 °C. ^1^H NMR (300 MHz, DMSO-*d*_6_) δ (ppm) 13.23 (s, 1H), 10.33 (s, 1H), 9.83 (s, 1H), 9.62 (s, 1H), 8.70 (s, 2H), 8.18 (s, 1H), 8.09 (m, 4H), 7.86 (d, *J* = 8.4 Hz, 1H), 7.73 (d, *J* = 9.3 Hz, 1H), 7.63 (m, 3H), 2.30 (t, *J* = 6.9 Hz, 2H), 1.96 (t, *J* = 7.2 Hz, 2H), 1.57 (m, 4H), 1.29 (m, 2H). ^13^C (150 MHz, DMSO-*d*_6_) δ (ppm) 171.27, 169.52, 145.14, 141.01, 136.97, 135.83, 135.50, 134.48, 132.69, 132.46, 130.07, 123.06, 121.15, 120.68, 119.88, 118.77, 116.97, 109.54, 107.58, 36.65, 32.62, 28.74, 25.39. HRMS (ESI) for C_23_H_27_N_8_O_3_ (M + H^+^): calcd 499.2206; found 499.2220.

#### 4.3.38 *N*1-(4-((6-(1H-Indazol-6-yl)­imidazo­[1,2-*a*]­pyrazin-8-yl)­amino)­phenyl)-*N*8-hydroxyoctanediamide (**14**)

Compound **14** was synthesized using the intermediate 40 and the same synthetic methodology as followed in compound 10. Off-white powder, yield 60.38%, HPLC purity: 98.551% (tR: 21.912 min); mp: 239–241 °C, ^1^H NMR (300 MHz, DMSO-*d*_6_) δ (ppm) 13.23 (s, 1H), 10.33 (s, 1H), 9.83 (s, 1H), 9.61 (s, 1H), 8.70 (s, 2H), 8.18 (s, 1H), 8.09 (m, 3H), 8.00 (s, 1H), 7.86 (d, *J* = 8.7 Hz, 1H), 7.73 (d, *J* = 8.7 Hz, 1H), 7.65 (m, 2H), 2.30 (t, *J* = 7.5 Hz, 2H), 1.95 (t, *J* = 7.5 Hz, 2H), 1.57 (m, 4H), 1.30 (m, 4H). ^13^C (150 MHz, DMSO-*d*_6_) δ (ppm) 171.33, 169.58, 145.17, 136.91, 135.83, 135.52, 134.48, 132.78, 132.51, 123.04, 121.14, 120.67, 119.88, 118.77, 118.60, 116.96, 116.61, 109.54, 36.74, 32.68, 28.89, 28.86, 25.56, 25.48. HRMS (ESI) for C_24_H_29_N_8_O_3_ (M + H^+^): calcd 513.2362; found 513.2371.

#### 4.3.39 *N*1-(4-((6-(1H-Indazol-6-yl)­imidazo­[1,2-*a*]­pyrazin-8-yl)­amino)­phenyl)-*N*9-hydroxynonanediamide (**15**)

Compound **15** was synthesized using the intermediate 41 and the same synthetic methodology as followed in compound 10. Off-white powder, yield 59.28%, HPLC purity: 95.102% (tR: 24.109 min); mp: 220–223 °C, ^1^H NMR (300 MHz, DMSO-*d*_6_) δ (ppm) 13.22 (s, 1H), 10.32 (s, 1H), 9.82 (s, 1H), 9.61 (s, 1H), 8.70 (s, 1H), 8.17 (s, 1H), 8.08 (m, 3H), 8.00 (s, 1H), 7.86 (d, *J* = 8.4 Hz, 1H), 7.73 (m, 1H), 7.65 (m, 3H), 2.30 (m, 2H), 2.00 (m, 2H), 1.59 (m, 5H), 1.29 (m, 4H). ^13^C (150 MHz, DMSO-*d*_6_) δ (ppm) 173.80, 171.33, 145.21, 140.97, 136.88, 135.86, 134.47, 133.87, 131.94, 130.07, 123.06, 121.14, 120.68, 119.86, 118.76, 116.97, 107.51, 49.03, 36.79, 33.69, 32.75, 29.06, 28.79, 26.98, 25.63. HRMS (ESI) for C_25_H_31_N_8_O_3_ (M + H^+^): calcd 527.2519; found 527.2504..

#### 4.3.40 *N*1-(4-((6-(1H-Indazol-6-yl)­imidazo­[1,2-*a*]­pyrazin-8-yl)­amino)­phenyl)-*N*10-hydroxydecanediamide (**16**)

Compound **16** was synthesized using the intermediate 42 and the same synthetic methodology as followed in compound 10. Off-white powder, yield 62.10%, HPLC purity: 95.408% (tR: 25.820 min); mp: 220–223 °C, ^1^H NMR (300 MHz, DMSO-*d*_6_) δ (ppm) 13.25 (s, 1H), 9.85 (s, 1H), 9.63 (s, 1H), 8.72 (s, 2H), 8.20 (s, 1H), 8.112 (m, 3H), 8.02 (s, 1H), 7.88 (d, *J* = 8.4 Hz, 1H), 7.75 (m, 1H), 7.64 (m, 3H), 2.34 (m, 4H), 1.95 (t, *J* = 7.2 Hz, 1H), 1.64 (m, 4H), 1.30 (m, 8H). ^13^C (150 MHz, DMSO-*d*_6_) δ (ppm) 173.80, 171.35, 145.21, 140.97, 136.89, 135.86, 134.48, 133.86, 132.84, 132.56, 123.06, 121.13, 120.67, 119.86, 118.77, 116.94, 107.51, 51.57, 36.79, 33.70, 32.69, 29.09, 29.01, 28.87, 25.63, 24.85. HRMS (ESI) for C_26_H_33_N_8_O_3_ (M + H^+^): calcd 541.2675; found 541.2672.

#### 4.3.41 4-(((4-((6-(1H-Indazol-6-yl)­imidazo­[1,2-*a*]­pyrazin-8-yl)­amino)­phenyl)­amino)­methyl)-*N*-hydroxybenzamide (**17**)

Compound **17** was synthesized using the intermediate 43 and the same synthetic methodology as followed in compound 8. Yellowish white powder, yield 61.23%, HPLC purity: 97.821% (tR: 21.054 min); mp: 208–211 °C, ^1^H NMR (300 MHz, DMSO-*d*_6_ *)* δ (ppm) 13.23 (s, 1H), 11.17 (s, 1H), 9.38 (s, 1H), 8.65 (s, 1H), 8.157 (d, *J* = 14.1 Hz, 2H), 8.01 (s, 1H), 7.86 (d, *J* = 8.4 Hz, 3H), 7.72 (m, 4H), 7.50 (d, *J* = 8.1 Hz, 1H), 6.74 (d, *J* = 8.7 Hz, 2H), 4.39 (s, 2H). ^13^C (150 MHz, DMSO-*d*_6_) δ (ppm) 164.55, 158.53, 158.13, 144.93, 140.96, 137.54, 136.96, 135.48, 135.39, 133.82, 132.84, 131.96, 131.70, 129.90, 128.76, 127.82, 127.38, 123.08, 122.09, 121.21, 121.11, 118.88, 116.93, 110.25, 108.86, 107.63, 47.67. HRMS (ESI) for C_27_H_23_N_8_O_2_ (M + H^+^): calcd 491.1944; found 491.1931.

#### 4.3.42 (*E*)-3-(4-(((4-((6-(1H-Indazol-6-yl)­imidazo­[1,2-*a*]­pyrazin-8-yl)­amino)­phenyl)­amino)­methyl)­phenyl)-*N*-hydroxyacrylamide (**18**)

Compound **18** was synthesized using the intermediate 44 and the same synthetic methodology as followed in compound 9. Dark brown powder, yield 69.01%, HPLC purity: 95.711% (tR: 23.483 min); mp: 212–215 °C, ^1^H NMR (300 MHz, DMSO-*d*_6_) δ (ppm) 13.18 (s, 1H), 10.72 (s, 1H), 9.27 (s, 1H), 8.59 (s, 1H), 8.13 (m, 2H), 7.95 (s, 1H), 7.81 (m, 3H), 7.70 (d, *J* = 9.0 Hz, 1H), 7.60 (s, 1H), 7.54 (d, *J* = 7.8 Hz, 2H), 7.44 (m, 3H), 6.66 (d, *J* = 8.4 Hz, 2H), 6.45 (d, *J* = 15.9 Hz, 1H), 4.32 (s, 2H). ^13^C (125 MHz, DMSO-*d*_6_) δ (ppm) 163.36, 145.40, 144.76, 142.68, 141.00, 138.70, 135.79, 133.75, 132.58, 132.61, 129.94, 129.40, 128.22, 127.98, 123.37, 122.24, 122.09, 121.06, 118.90, 116.80, 112.78, 108.70, 107.53, 47.13. HRMS (ESI) for C_29_H_25_N_8_O_2_ (M + H^+^): calcd 517.2100; found 517.2084.

### 4.4 Biology

#### 4.4.1 AML Clinical Data Sources and Preprocessing

Gene expression and clinical data for AML patients were obtained from the publicly available GEPIA3 platform (incorporating TCGA-LAML and GTEx data sets)^71^ and the BeatAML2 data set,^72^ using normalized RNA-seq expression values. Gene-level quantification data were processed as log_2_(TPM+1) or normalized RPKM for downstream analyses. Clinical annotations, including age, FLT3 mutational status (FLT3-ITD or FLT3-WT), overall survival, and ELN2027 risk classification, were retrieved from the corresponding data sets and associated metadata. Statistical significance was assessed using Welch’s *t* test.

#### 4.4.2 Quantitative Complexity Measurement (QCM) Analysis

MD simulations were performed using UCSF Chimera in an aqueous environment to simulate the *in vitro* conditions. All molecules were solvated using the TIP3P Amber model for water (TIP3PBOX) in a truncated octahedron periodic box with a size of 10 Å and neutralized with the appropriate ions if necessary.^73^ For the MD, translational and rotational remover constraints have been applied every three steps. Minimization was performed using the default parameters with a gradient of 10 steps of 0.02 Å for a total of 100 steps. Equilibration was carried out over 2000 steps, heating the system to 298°K through a 10 K/ps gradient every two steps in the NPT ensemble, using the Andersen barostat and Nosé thermostat integrated in Chimera.^74,75^ The production phase was extended for 50 ns in 50,000 steps, in the isothermal–isobaric (NPT) ensemble, to maintain the constant motion of the atom trajectories. Each step was saved to avoid the loss of any important information.^76^ The MD of each molecule was exported as a pdb file and subjected to Quantitative Complexity Measurement (QCM) analysis, using the Artificial Intuition tools developed by Ontonix, as previously reported.

#### 4.4.3 Chemicals and Reagents

Matrigel basement membrane matrix high concentration (#354248) was obtained from Corning Inc. (Corning, NY, USA). Dimethyl sulfoxide (DMSO), PEG-400, and Tween 80 were purchased from Sigma-Aldrich (St. Louis, MO, USA). The primary antibodies used in this study included those against SYK (#E-AB-68344) and a horseradish peroxidase (HRP)-conjugated secondary antibody targeting rabbit immunoglobulin G (IgG; #E-AB-1003), both purchased from Elabscience Biotechnology Inc. (Houston, Texas, USA). Additionally, antibodies against caspase-3 (#19677–1-AP), GAPDH (#60004–1-Ig), phosphorylated SYK (#83331–1-AP), and an HRP-conjugated secondary antibody against mouse IgG (#SA00001–1) were obtained from Proteintech (Rosemont, IL, USA). The antibody that recognizes phosphorylated FOXO1 (#9461S) was sourced from Cell Signaling Technology (Beverly, MA, USA), while the primary antibody against phosphorylated AKT (#GTX50128) was acquired from GeneTex (Irvine, CA, USA). Moreover, primary antibodies against acetyl α-tubulin (#AF4351) and acetyl histone H3–K9 (#A7255) were purchased from Affinity Bioscience (Ann Arbor, MI, USA) and Abclonal (Woburn, MA, USA), respectively. DAPI (#HY-D0814) and rhodamine 123 (#16672) were acquired from MedChemExpress (Monmouth Junction, NJ, USA) and Cayman Chemical (Ann Arbor, MI, USA), respectively. Lastly, the Anti-Fade fluorescence mounting medium (#Ab104135) was obtained from Abcam (Cambridge, MA, USA).

#### 4.4.4 Cell Culture

MV4–11 is a human acute myeloid leukemia (AML) cell line obtained from the American Type Culture Collection (VA, USA). It was cultured in Iscove’s modified Dulbecco’s medium (#12200036; Gibco, Thermo Fisher Scientific Inc., Waltham, MA, USA), supplemented with 10% fetal bovine serum (#35–010-CV; Corning, Glendale, AZ, USA), 1.5 g/L sodium bicarbonate, and a 100× diluted penicillin–streptomycin solution (#30–002-CI; Corning). Cells were kept at 37 °C in a humidified incubator with 5% (v/v) CO_2_ and 95% (v/v) air. The culture medium was refreshed every 2–3 days.

#### 4.4.5 Cell Viability Analysis (MTT Assay)

The MTT assay was performed as per our previously reported methodology.^53^

#### 4.4.6 SYK Inhibitory Assay and HDAC Isoform Enzyme Inhibition Assays

The compounds were submitted to Reaction Biology Corporation, Malvern, PA (http://www.reactionbiology.com) for the evaluation of the SYK inhibitory assay and HDAC isoform inhibitory potential.

#### 4.4.7 Cell Cycle Analysis

Cell cycle analysis was performed as per the previously reported methodology.^53^

#### 4.4.8 Immunoblot

The harvested cells were lysed using a lysis solution containing 25 mM Tris–HCl (pH 7.6), 150 mM NaCl, 0.1% sodium dodecyl sulfate, 0.5% sodium deoxycholate, 5 mM EDTA, 1% Triton X-100, 10% glycerol, 1% ProteaseArrest, and one tablet of PhosSTOP per 10 mL. The lysate was then centrifuged at 14,000*g* for 10 min at 4 °C. The protein concentration of the supernatants was quantified using a Bio-Rad protein assay kit (Bio-Rad; Hercules, CA, USA). Aliquots of 40–50 μg of total proteins were separated by 10% SDS-PAGE and transferred to polyvinylidene difluoride (PVDF) membranes (#10600021; Cytiva, Marlborough, MA, USA). The membranes were blocked with 5% (w/v) skim milk and incubated with primary and secondary antibodies. The chemiluminescent signals were developed with an Advansta WesternBright ECL HRP substrate (#K-12045-D20; San Jose, CA, USA) and acquired by an iBright FL1500 imaging system (Thermo Fischer Scientific Inc.). The band intensities of the detected proteins were normalized to that of the internal control protein (GAPDH), and the expression levels of the identified proteins were presented as fold changes relative to the control group.

#### 4.4.9 *In Vitro* Kinase Assays

Inhibition activity of compound **14** against 60 kinases was assayed by the Kinase Enzymatic Radiometric [Km ATP] KinaseProfiler LeadHunter Assay of Eurofins-Cerep SA (Celle-Lévescault, France).

#### 4.4.10 Real-Time Polymerase Chain Reaction

Total RNA was extracted using RNAzol RT (#RN190; Molecular Research Center Inc., Cincinnati, OH, USA) according to the manufacturer’s instructions. Complementary DNA (cDNA) synthesis was performed using the ReverTra Ace kit (#PU-TRT-200; TOYOBO, Osaka, Japan) following the manufacturer’s guidelines. A real-time PCR was conducted using the THUNDERBIRD Next SYBR qPCR Mix (#QPX-201T; TOYOBO Co., Ltd., Osaka, Japan) in a LightCycler 480 PCR System (Roche Diagnostics, IN, USA). The reaction mixture included 1 μg of cDNA, 1 μL of each primer (10 μM), and 10 μL of the qPCR Mix, bringing the total volume to 20 μL. Following a hot-start activation for 2 min at 95 °C, 45 cycles were performed, with each cycle consisting of 10 s at 95 °C, 10 s at 60 °C, and 10 s at 72 °C. The primer pairs synthesized by Genomics (New Taipei City, Taiwan) were as follows: GAPDH (#NM_002046; forward: GTCTCCTCTGACTTCAACAGCG and reverse: ACCACCCTGTTGCTGTAGCCAA) and SREBF1 (#NM_001005291; forward: ACTTCTGGAGGCATCGCAAGCA and reverse: AGGTTCCAGAGGAGGCTACAAG). The relative transcript expression was calculated using the eq 2^–ΔΔCt^, and the results are presented as multiples of change relative to the control group.

#### 4.4.11 Evaluation of Nuclear Morphology and Mitochondrial Membrane Potential

DAPI staining and rhodamine staining were performed as per the previously reported methodology.^53^

#### 4.4.12 Annexin V–PI Staining

Apoptosis in the treated cells was assessed using the Annexin V-FITC/PI Apoptosis Kit (#E-CK-A211; Elabscience Biotechnology Inc.) according to the manufacturer’s instructions. Briefly, the treated cells were collected and washed with PBS. They were then counted and aliquoted at a concentration of 5 × 10^5^ cells per tube. After centrifuging at 300*g* for 5 min, the pellet was resuspended in 500 μL of 1× Annexin V binding buffer. Next, 5 μL of Annexin V-FITC and 5 μL of PI were added. The solution was gently mixed and incubated in the dark at room temperature for 20 min. Finally, the stained cells were analyzed using flow cytometry.

#### 4.4.13 RNA Sequencing

MV4–11 cells were treated with 0.5 μM compound **14** for 48 h, followed by total RNA extraction using the NucleoSpin RNA kit (#740955.50; Takara Bio USA Inc., San Jose, CA, USA) according to the manufacturer’s protocol. RNA sequencing was performed by BIOTOOLS Co. (New Taipei City, Taiwan). The quantity, purity, and integrity of the extracted RNA were evaluated using SimpliNano Spectrophotometers (Biochrom, MA, USA) and the Qsep 100 DNA/RNA Analyzer (BiOptic Inc., New Taipei City, Taiwan). Libraries were constructed from 1 μg of RNA per sample using the KAPA mRNA HyperPrep Kit, which included poly­(A) enrichment, controlled fragmentation, and strand-specific cDNA synthesis. Size-selected cDNA fragments (300–400 bp) were amplified and quality-checked before sequencing. High-throughput sequencing generated 150 bp paired-end reads on the Illumina NovaSeq 6000 platform. Raw reads underwent quality filtering, adapter removal, and genome alignment, followed by gene quantification, normalization, and differential expression analysis. Enrichment and network analyses of differentially expressed genes (DEGs) were conducted to gain functional insights. P-values from DEG detection were adjusted for the false discovery rate (FDR) using the Benjamini–Hochberg method. Gene Ontology (GO) and KEGG pathway enrichment analyses were performed using ClusterProfiler, while Disease Ontology (DO) enrichment was conducted *via* the DOSE package, integrating data from the DO, DisGeNET, and NCG databases.

#### 4.4.14 Tube Formation Assay and Migration Assay

The endothelial tube formation assay and migration assay using HUVECs were performed as per the previously reported methodology.^77^

#### 4.4.15 *In Vivo* Pharmacokinetics Study

All of the animals and experiments were approved by the ethical committee of Siksha “O” Anusandhan, India **(IAEC/SPS/SOA/271/2025).** Compound **14** was given to rats at a 1 mg/kg dose for both intraperitoneal (IP) and intravenous (IV) routes and a 50 mg/kg dose for the oral route. Rat blood (0.5 mL) was collected in an EDTA anticoagulant tube to prevent clotting at the following time points: 15, 30, 60, 120, 240, and 480 min. The EDTA tube was placed in a cooling centrifuge, and samples were centrifuged at 15,000 rpm for 20 min at 4 °C. The plasma was carefully transferred into a clean microcentrifuge tube using a pipette. Further, ice-cold acetonitrile (ACN) was added to the plasma in a 1:4 ratio (Plasma:ACN), and the contents were thoroughly mixed using a vortex mixer at 2,500 rpm for 30 s. The incubation of the mixture was carried out at −20 °C for 10 min to enhance protein precipitation, followed by centrifugation of the incubated mixture at 15,000 rpm for 10 min at 4 °C. The clear supernatant (protein-free plasma) was carefully transferred to a new tube, avoiding any pellet disruption. The supernatant was filtered through a 0.2 μm syringe filter into a clean tube, and the sample was subsequently diluted and injected into the LC-MS for analysis.

#### 4.4.16 *In Vivo* Antitumor Study (Xenograft Mouse Tumor Model)

The animal study with approval number #LAC-2023–0436 was conducted following the guidelines of the Institutional Animal Care and Use Committee of Taipei Medical University (TMU). The male BALB/c nude mice (CAnN.Cg-Foxn1nu/Crl; six-week-old) were procured from BioLASCO Taiwan Co. (Taipei City, Taiwan) and were housed in a 12-h light/dark cycle at a temperature of 25 °C. The protocol as described in our previous study was employed for the *in vivo* antitumor evaluation.^53^

#### 4.4.17 Immunohistochemistry (IHC) Staining

Subcutaneous tumors were harvested and fixed in 10% neutral buffered formalin. Tissues were embedded in paraffin and sectioned with assistance from the National Center for Biomodels (Taipei, Taiwan). For immunohistochemistry, tissue sections were deparaffinized in xylene, rehydrated through graded ethanol, and rinsed with water. Heat-induced antigen retrieval was performed in 10 mM sodium citrate buffer at 60 °C for 1.5 h. After washing with 1× TBST (Tris-buffered saline with 0.1% Tween 20), endogenous peroxidase activity was quenched with 3% hydrogen peroxide in methanol for 10 min at room temperature. Nonspecific binding was blocked with 1% bovine serum albumin (BSA) in 1× TBST for 1 h, followed by overnight incubation with the primary antibody diluted in 1× TBST at 4 °C. The following day, sections were washed and incubated with a biotinylated secondary antibody (#ab6720; Abcam) for 1 h at room temperature and then processed with the VECTASTAIN Elite ABC kit (#PK-6100; Vector Laboratories, CA, USA) according to the manufacturer’s instructions. Signal detection was achieved using the DAB substrate (#K3467; Agilent Dako, CA, USA). Slides were rinsed, counterstained with hematoxylin solution (#1.05174.0500; Sigma-Aldrich), dehydrated through graded ethanol and xylene, and mounted with Micromount medium (#3801731; Leica Biosystems, IL, USA). Images were acquired using an OLYMPUS IX81 microscope (Olympus Co., Tokyo, Japan) equipped with a SPOT RT3 CCD camera.

#### 4.4.18 Histopathological Analysis

Various organs, including the heart, liver, spleen, lungs, and kidneys, were collected from sacrificed animals to examine histopathological changes. The organs underwent paraffin embedding, tissue sectioning, and hematoxylin-eosin (HE) staining, which were carried out by the Laboratory Animal Center of TMU.

#### 4.4.19 Biochemical Blood Analysis

Whole blood was collected from sacrificed animals to evaluate potential tissue toxicity. The blood samples were allowed to clot at room temperature for 30 min and then centrifuged at 2000*g* for 10 min at 4 °C to obtain serum. The serum levels of aspartate aminotransferase (AST), alanine transaminase (ALT), blood urea nitrogen (BUN), creatine phosphokinase (CPK), and creatinine (CRE) were measured by the National Center for Biomodels (Taipei, Taiwan).

#### 4.4.20 Statistical Analysis

SPSS Statistics software (IBM; Armonk, NY, USA) was used to perform the statistical analysis. All values were presented as mean ± standard deviations (SD). A one-way analysis of variance (ANOVA) combined with a post hoc test (Turkey test) was used to determine significant differences. A p-value less than 0.05 was considered statistically significant. The significance level is denoted by * *p* < 0.05 and ** *p* < 0.01.

#### 4.4.21 Molecular Modeling Study

The crystal structures of HDAC1, HDAC2, HDAC3, HDAC6, and SYK (PDB IDs: 5ICN, 6WBZ, 4A69, 5EDU and 4PUZ, respectively) were retrieved from the protein data bank (https://www.rcsb.org/).^78,79^ All of the crystal structures were prepared individually by removing existing ligands, water molecules, unbound ions, and extra chains using PyMOL (Schrödinger, LLC). Thereafter, nonpolar hydrogens were merged while polar hydrogens were added to each protein using AutoDock tools.^80^ This process was repeated for each protein and then saved in docking-ready PDBQT format. 2-D chemical structure of the compound **14** was drawn using MarvinSketch 18.5.0 (ChemAxon Ltd.) and converted into 3D conformation (mol2 format). The ligand molecule was further saved into the docking-ready PDBQT format using AutoDock tools. Docking of the ligands (compound **14)** as well as cocrystallized PDB ligands to various protein targets and determination of binding affinities was carried out using AutoDock Vina.^81^

## Supplementary Material





## Notes

Structures of investigational SYK inhibitors and FDA-approved HDAC inhibitors, clinical transcriptomic analysis; structural investigation of entospletinib; validation of docking protocol; docking study—compound **1** (2D docked poses); docking study—compound **14** (SYK—orientation and 2D docked poses); docking study—compound **14** (HDAC isoforms—orientation and 2D docked poses); molecular dynamics (MD); assessment of the expression levels of p21, an HDAC downstream target, and p-PLCγ2 Y1217, a SYK downstream target, following treatment with compound **14**; pharmacokinetic characterization of compound **14**; *in vitro* assessment—antitumor activity of compound **14** against HL-60 cell lines; antileukemic activity in leukemia induced NOD SCID mice model; ^1^H NMR for compounds **ED1** to **ED-30**; ^1^H NMR for compounds **1–18**; ^13^C NMR for compounds **1–18**; HPLC for compounds **1–18**; HRMS for compounds **1–18** (PDF)
Molecular formula strings (CSV)

## Abbreviations Used

HDAC: histone deacetylase
GEPIA3: gene expression profiling interaction 3
BLNK: B cell linker
ERK: extracellular regulated kinase
FLT3-ITD: fms-like tyrosine kinase 3–internal tandem duplication
SLK: STE20-like kinase
LCK: lymphocyte specific protein tyrosine kinase
GSEA: gene set enrichment analysis
KEGG: Kyoto Encyclopedia of Genes and Genomes
DHCR24: 24-dehydrocholesterol reductase
FASN: fatty acid synthase
LDLR: low-density lipoprotein receptor
SCD: sickle cell disease
SREBF1: sterol regulatory element-binding transcription factor
FOXO1: forehead box protein O1
HPLC: high-performance liquid chromatography
NMR: nuclear magnetic resonance
DBU: 1,8-diazabicyclo­[5.4.0]­undec-7-ene
HATU: O-(7-azabenzotriazol-1-yl)-N,N,N’,N’-tetramethyluronium hexafluorophosphate
RPKM: reads per kilobase per million mapped reads
HUVECs: human umbilical vein endothelial cells
EDTA: ethylenediaminetetraacetic acid
ACN: acetonitrile
TFA: trifluoroacetic acid
IC_50_: half-maximum inhibitory concentration
SAR: structural activity relationship
PI3K: phosphoinositide 3-kinase
