# Supplementary material for: Dual SYK-HDAC Inhibitor Elicits Striking Efficacy against Acute Myeloid Leukemia: Rational Design, Synthesis, and Biological Evaluation
Source: J Med Chem. 2026 Jun 9;69(12):14275–308. doi: 10.1021/acs.jmedchem.6c00039 (PMC13312455; doi:10.1021/acs.jmedchem.6c00039)

SUPPORTING INFORMATION

A Dual SYK-HDAC Inhibitor Elicits Striking Efficacy Against Acute Myeloid Leukemia: Rational Design, Synthesis and Biological Evaluation

Anshul Mishra[a#], Wen-Bin Yang [b,c#], Tsu-Shao Liu[a], Amandeep Thakur[a], Ajmer Singh Grewal[d], Jacek Marczyk[e,f], Giovanni Stelitano[g], Ram Sharma[a], Mandeep Rana[a], Gurpreet Singh[h], Santosh Kumar Guru[i], Goutam Rath[j], Saurabh Chawla[k,l], Jing Ping Liou[a,m], Chun Hsu Pan[a*], Kunal Nepali[a,m*]

[a] School of Pharmacy, College of Pharmacy, Taipei Medical University, Taipei 110031, Taiwan.

[b] Research Center for Neuroscience, Taipei Medical University, Taipei 110031, Taiwan.

[c] Ph.D. Program in Medical Neuroscience, College of Medical Science and Technology, Taipei Medical University, Taipei 110031, Taiwan.

[d] Department of Pharmaceutical Sciences, Guru Gobind Singh College of Pharmacy, Near Guru Nanak Khalsa College, Yamuna Nagar 135001, Haryana, India.

[e] Ontonix S.r.l , 23100, Sondrio, Italy.

[f] BioDynLab Ltd., Toronto, Ontario M5B1Y4, Canada.

[g] Department of Biotechnology, University of Pavia, Via Ferrata 9, 27100, Pavia, Italy.

[h] Department of Pharmaceutical Chemistry, ISF College of Pharmacy, Moga 142001, Punjab, India.

[i] Department of Biological Sciences, National Institute of Pharmaceutical Education and Research, Hyderabad 500037, India.

[j] Department of Pharmaceutics, School of Pharmaceutical Science, Siksha 'O' Anusandhan (Deemed to Be University), Bhubaneswar, Odisha, 751003, India.

[k] School of Biological Sciences, National Institute of Science Education and Research (NISER), Bhubaneswar, P.O. Bhimpur-Padanpur, Jatni, Khurda, Odisha, 752050, India.

[l] Homi Bhabha National Institute (HBNI), Training School Complex, Anushaktinagar, Mumbai, 400094, India.

[m] Ph.D. Program in Drug Discovery and Development Industry, College of Pharmacy, Taipei Medical University, Taipei 110031, Taiwan.

# Anshul Mishra and Wen-Bin Yang are joint first authors

*** Corresponding authors:**

1) **Kunal Nepali, E-mail: nepali@tmu.edu.tw**
2) **Chun Hsu Pan, E-mail: panch@tmu.edu.tw**

Contents:

1. Structures of investigational SYK inhibitors and FDA-approved HDAC inhibitors …………. S3

2. Clinical transcriptomic analysis………………………………………………………………S4-S6

3. Structural Investigation of Entospletinib ………………………………………………………S7-S9

4. Validation of docking protocol…………………………………………………………………..S10

5. Docking Study – Compound 1 (2D Docked poses) ……………………………………………….S11-S12

6. Docking Study – Compound 14 (SYK - Orientation and 2D docked poses) ……………………S13

7. Docking Study – Compound 14 (HDAC isoforms - Orientation and 2D docked poses) ……….S14-S15

8. Molecular dynamics (MD)………………………………………………………………………..S16-S21

9. Assessment of the expression levels of p21, an HDAC downstream target, and p-PLCγ2 Y1217, a SYK downstream target, following treatment with compound 14……………………...S22-S23

10. Pharmacokinetic characterization of compound 14……………………………………………S24

11. In-vitro assessment - antitumor activity of compound 14 against HL-60 cell lines……………..S25-S30

12. Anti-leukemic Activity in Leukemia Induced NOD SCID Mice Model…………………………S31-S37

13. ^{1}H NMR for compounds ED-1 to ED-30………………………………………………...S38-S52

14. ^{1}H NMR for compounds 1-18………………………………………………………….…S53-S61

15. ^{13}C NMR for compounds 1-18……………………………………………………..........S62-S70

16. HPLC for compounds 1-18…………………………………………………………………….S71-S88

17. HRMS for compounds 1-18………………… ……………………………………….........S89-S97

1. Structures of investigational SYK inhibitors and FDA-approved HDAC inhibitors.

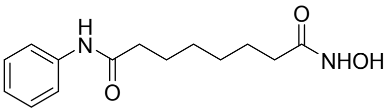

I. SAHA

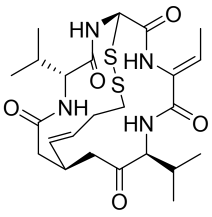

II. Romidepsin

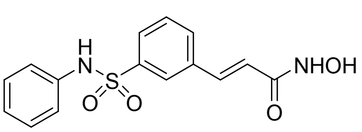

III. Belinostat (PXD-101)

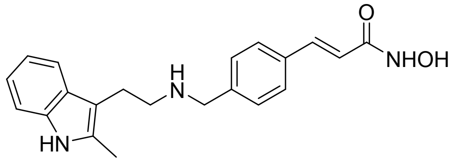

IV. Panabinostat (LBH-589)

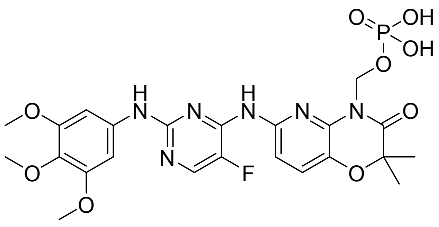

V. Fostamatinib

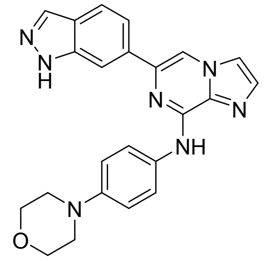

VI. Entospletinib

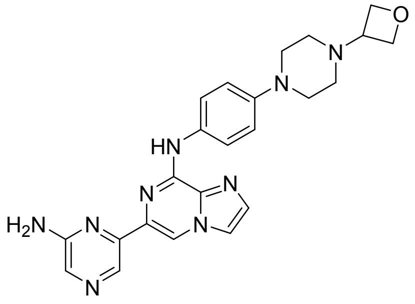

VII. Lanraplenib

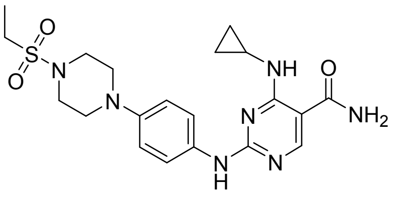

VIII. Cerdulatinib

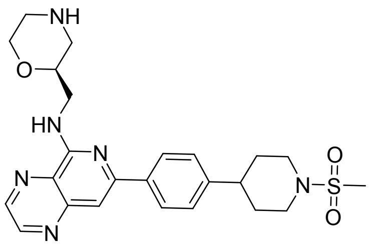

IX. Sovleplenib

Fig. 1S Structures of investigational SYK inhibitors and FDA-approved HDAC inhibitors.

2. Clinical transcriptomic analysis supports the therapeutic relevance of SYK and HDAC co-targeting in AML

To support the proposed co-targeting approach, we analyzed the publicly available AML transcriptomic datasets using the GEPIA3 platform. Gene expression profiles and associated clinical annotations, including patient age, FLT3 mutational status (FLT3-ITD vs FLT3-WT), overall survival, and ELN2027 risk classification, were retrieved from the corresponding datasets. It was observed that SYK expression was significantly elevated in AML samples relative to normal controls (**Fig. 2S A**). Increased SYK expression was associated with poorer overall survival (**Fig. 2S B**), suggesting clinical relevance of SYK signaling in AML progression. Stratified analysis further demonstrated variation across FLT3 status, age groups, and ELN risk categories, with higher SYK levels observed in adverse-risk FLT3-ITD AML (**Fig. 2S E**). A differential expression pattern was also displayed by Multiple HDAC isoforms. Among them, HDAC1, HDAC2, HDAC3, and HDAC6 showed the highest transcript abundance. Also, a correlation of HDAC1 expression with reduced survival probability was observed in the survival analysis (**Fig. 2S C–D**). Notably, HDAC1 levels demonstrated a tendency toward enrichment in higher-risk disease categories (**Fig. 2S F**). Collectively, these observations indicate that SYK and HDAC pathways are clinically associated with aggressive AML phenotypes, thereby providing a rationale for simultaneous targeting of these targets. Importantly, these analyses were intended to support target selection and do not establish a driver role of SYK or HDAC isoforms in AML pathogenesis.

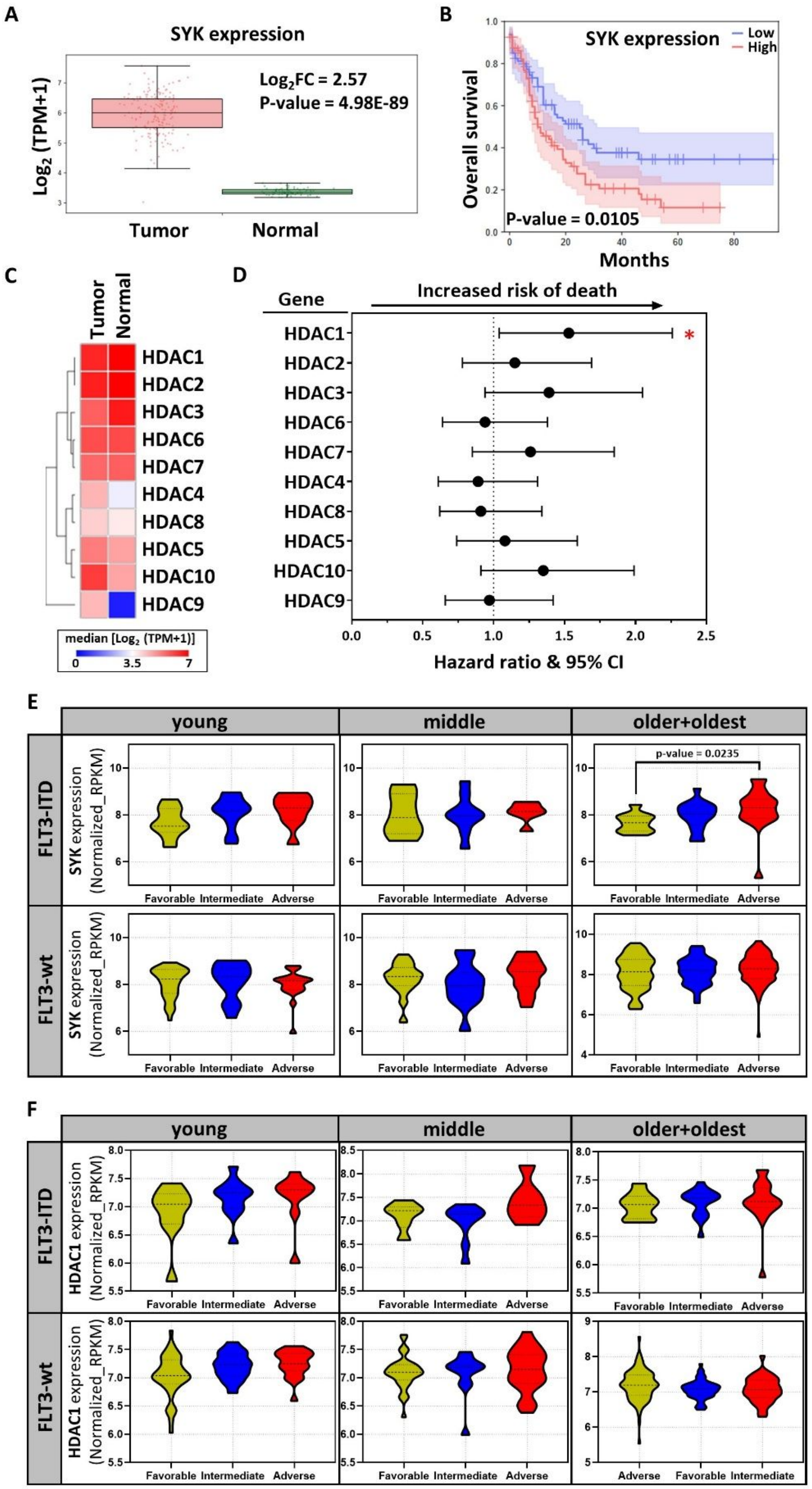
A
SYK expression
Log2 (TPM+1)
Log2FC = 2.57
P-value = 4.98E-89
Tumor
Normal
B
SYK expression
Low
High
Overall survival
P-value = 0.0105
Months
C
Tumor
Normal
HDAC1
HDAC2
HDAC3
HDAC6
HDAC7
HDAC4
HDAC8
HDAC5
HDAC10
HDAC9
median [Log2 (TPM+1)]
0
3.5
7
D
Gene
Increased risk of death
HDAC1
HDAC2
HDAC3
HDAC6
HDAC7
HDAC4
HDAC8
HDAC5
HDAC10
HDAC9
*
0.0
0.5
1.0
1.5
2.0
2.5
Hazard ratio & 95% CI
E
young
middle
older+oldest
FLT3-ITD
FLT3-wt
SYK expression (Normalized_RPKM)
p-value = 0.0235
Favorable
Intermediate
Adverse
F
young
middle
older+oldest
FLT3-ITD
FLT3-wt
HDAC1 expression (Normalized_RPKM)
Favorable
Intermediate
Adverse

Fig. 2S. Clinical association of SYK and HDAC isoform expression in AML. (A) Boxplots showing SYK mRNA expression levels in AML tumor samples compared with normal controls. (B) Kaplan–Meier overall survival curves stratified by high versus low SYK expression. (C) Violin plots showing SYK expression in patients with FLT3-ITD (top row) or FLT3-wild-type (bottom row) across age groups (young, middle, older/oldest) and AML risk categories defined by the European LeukemiaNet (ELN2027) classification (favorable, intermediate, adverse). (D) Heatmap showing expression patterns of HDAC family genes (HDAC1–HDAC10) in AML tumor samples compared with normal controls. (E) Forest plot displaying hazard ratios (HR) with 95% confidence intervals for overall survival associated with individual HDAC gene expression. Values to the right of the dashed line (HR = 1) indicate increased risk of death (*p < 0.05, ***p < 0.001). (F) Violin plots showing HDAC1 expression in patients with FLT3-ITD (top row) or FLT3-wild-type (bottom row) across age groups (young, middle, older/oldest) and AML risk categories defined by the ELN2027 classification (favorable, intermediate, adverse).

3. Structural Investigation of Entospletinib

A series of 30 Entospletinib derivatives was furnished and evaluated for their SYK inhibitory effects. The structural interrogation was conducted to select the most potent Entospletinib derivative that could be installed in the HDAC inhibitory structural template. The chemical route to the synthesis of Entospletinib derivatives is depicted in **Scheme 1,** and the SYK inhibitory activity of the synthesized adducts is presented in the supporting information. Correlation of the impact of C-6 substituents with the SYK inhibitory values (**ED-1 to ED-27**) revealed that none of the substituents could outshine indazole as a C-6 occupier. Indeed, switching from bicyclic heteroaryl fragments to monocyclic aromatic rings irrespective of the electronic effects (OCH_3, CN, F, OH, CF_3 and disubstitutions) culminated in a notable decline in the SYK inhibitory activity of the compounds. In addition to the assessment of C-6 substituents, replacement of morpholine with piperazine (**ED-28**), truncation of the morpholine ring (**ED-29**) and the removal of the phenyl ring bearing the piperazine ring were the other three structural alterations explored. The former two alterations did not elicit any significant variation in the activity profile, as compounds were found to be tantamount in potency to Entospletinib, while the latter structural change was not tolerated, as compound **(ED-30)** exhibited a decline in SYK inhibitory activity.

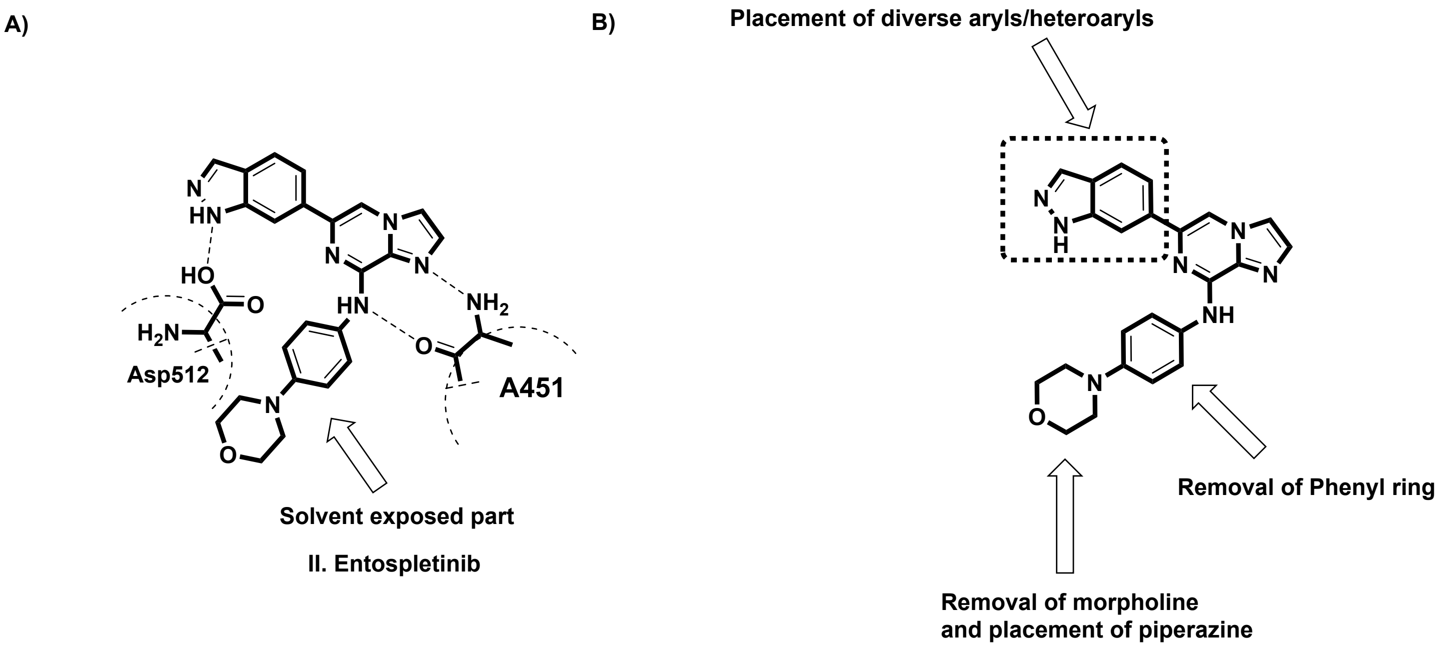

C)

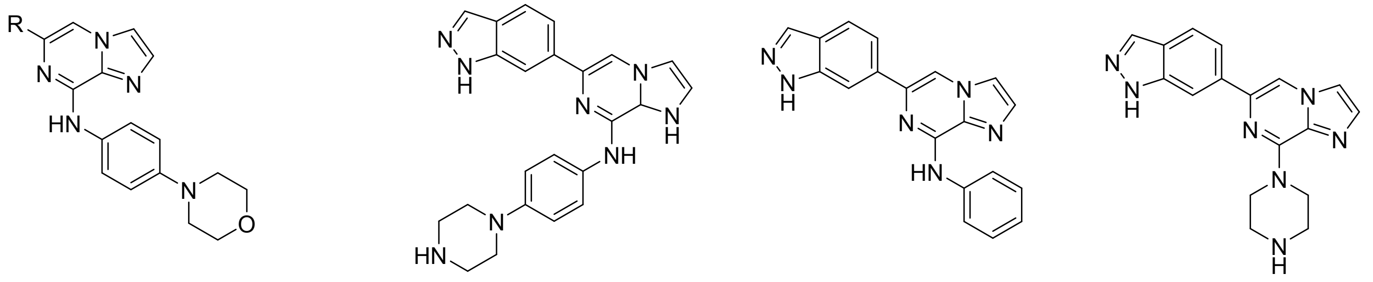

ED-1-27 **ED-28** **ED-29** **ED-30**

ED-1. R = phenyl
ED-2. R = pyridin-3-yl
ED-3. R = quinolin-6-yl
ED-4. R = 3,4,5-trimethoxyphenyl
ED-5. R = 1*H*-indol-5-yl
ED-6. R = 4-methoxyphenyl
ED-7. R = 4-nitrophenyl
ED-8. R = 4-(*tert*-butyl)phenyl
ED-9. R = 4-chlorophenyl
ED-10. R = pyrimidin-5-yl
ED-11. R = 3,5-dimethoxyphenyl
ED-12. R = phenol
ED-13. R = furan-3-yl
ED-14. R = thiophen-3-yl
ED-15. R = 2,3-dihydrobenzo[*b*][1,4] dioxin-6-yl
ED-16. R = 1,3-dimethyl-1*H*-pyrazol-5-yl)
ED-17. R = pyridin-4-yl
ED-18. R = 2-methylpyridin-4-yl
ED-19. R = 4-fluorophenyl
ED-20. R = 6-fluoropyridin-3-yl
ED-21. R = benzonitrile
ED-22. R = 1*H*-indol-4-yl
ED-23. R = benzaldehyde
ED-24. R = 4-(trifluoromethyl)phenyl
ED-25. R = 3,4-difluorophenyl
ED-26. R = 3-chloro-4-fluorophenyl)
ED-27. R = 4-(methylsulfonyl)phenyl)

Fig. 3S (A) Structure activity relationship of Entospletinib. (B) Structural Interrogation of Entospletinib. (C) Synthesized Entospletinib derivatives (**ED-1 to ED-30**)

Table 1S. SYK inhibitory activity of ED-1 to ED-30

**Compound code**	**% Inhibition SYK (IC_{50}, 100nM)**
ED-1	32.30
ED-2	50.84
ED-3	83.74
ED-4	53.99
ED-5	65.02
ED-6	55.13
ED-7	27.26
ED-8	36.56
ED-9	40.68
ED-10	59.35
ED-11	57.42
ED-12	58.65
ED-13	23.18
ED-14	20.97
ED-15	42.33
ED-16	39.37
ED-17	55.45
ED-18	51.71
ED-19	48.42
ED-20	42.33
ED-21	28.91
ED-22	87.82
ED-23	32.42
ED-24	36.62
ED-25	40.95
ED-26	44.52
ED-27	49.27
ED-28	99.31
ED-29	97.07
ED-30	47.69
Entospletinib	99.06

Procedure for assay

The test compounds were evaluated in vitro against SYK using the SYK assay kit from BPS Bioscience, USA. All samples and Entospletinib were tested in duplicate according to the kit's instructions. Test compounds were initially dissolved in DMSO to 10 mM and then diluted into kinase buffer to 100 nM. Luminescence was recorded, and the percentage inhibition was calculated in Microsoft Excel by normalizing to the DMSO-treated control and the blank solution (1).

4. Validation of docking protocol

The docking protocol used in this study was first validated by redocking the co-crystallized ligands of the target proteins (except HDAC-3). The re-docked ligands of these proteins (HDAC1, HDAC2, HDAC6 and SYK) produced poses similar to those of the co-crystallized inhibitors with HDAC1, HDAC2, HDAC6 and SYK **(Fig. 4S)** with docking score (kcal/mol) of -7.7, -9.1, -7.8 and -8.1, respectively for HDAC1, HDAC2, HDAC6 and SYK (PDB IDs: 5ICN, 6WBZ, 5EDU and 4PUZ, respectively), indicating that a rational docking protocol was used in this study.

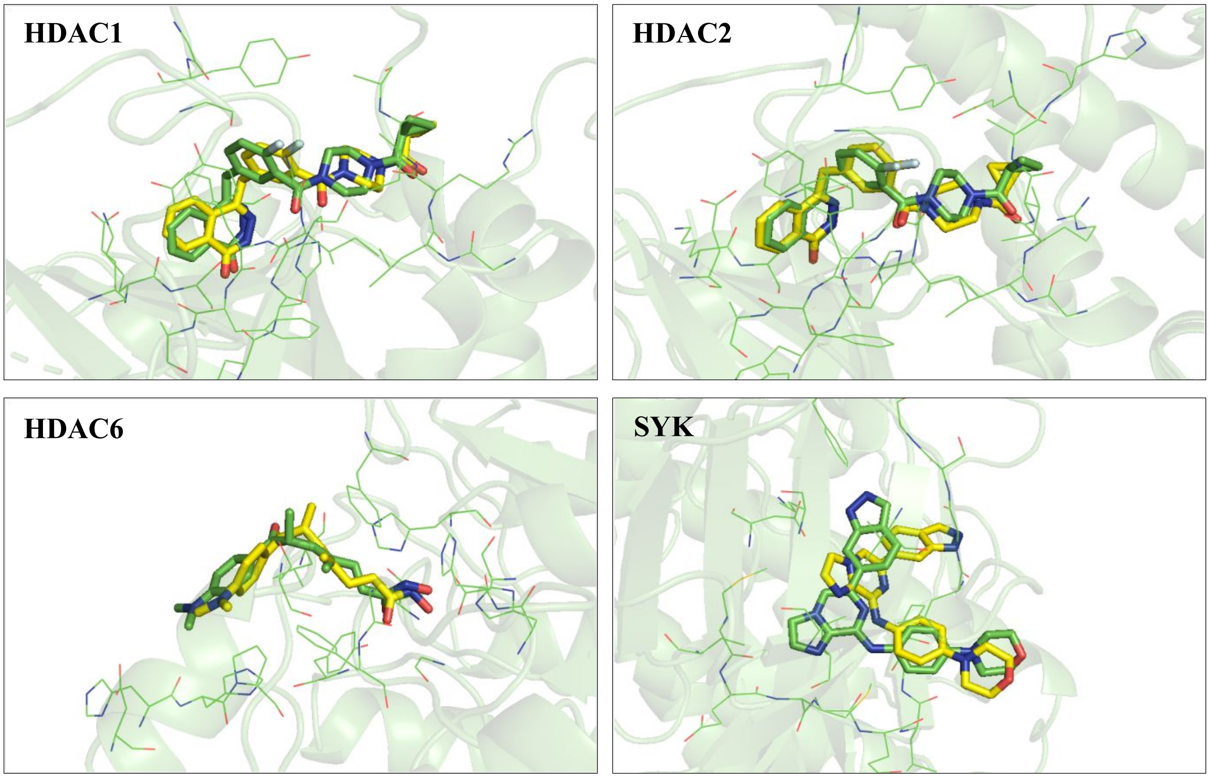

Fig. 4S Validation of the docking protocol. The docking protocol was validated via redocking the co-crystallized ligands. The re-docked ligands (yellow) produced a pose similar to those of the co-crystallized ligands (green).

5. Docking Study – Compound 1 (2D Docked poses)

SYK

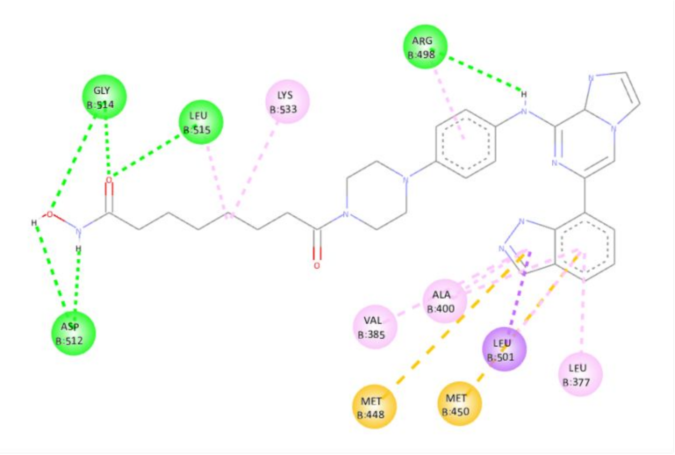

HDAC1

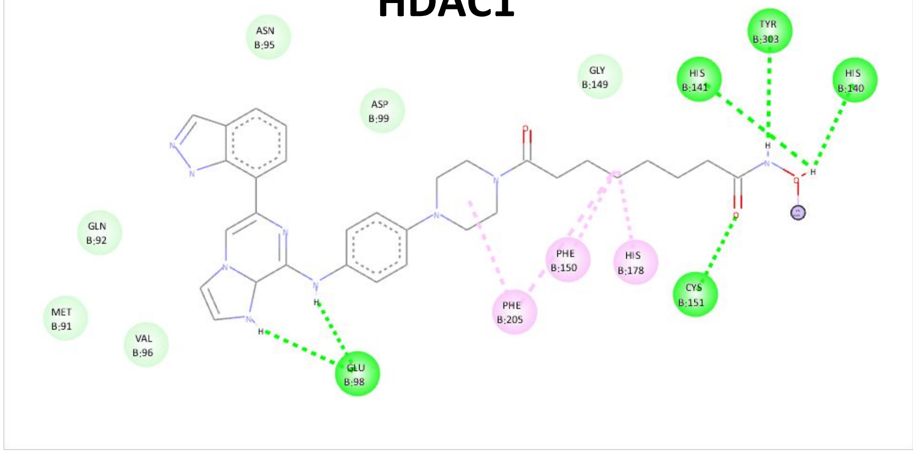

HDAC2

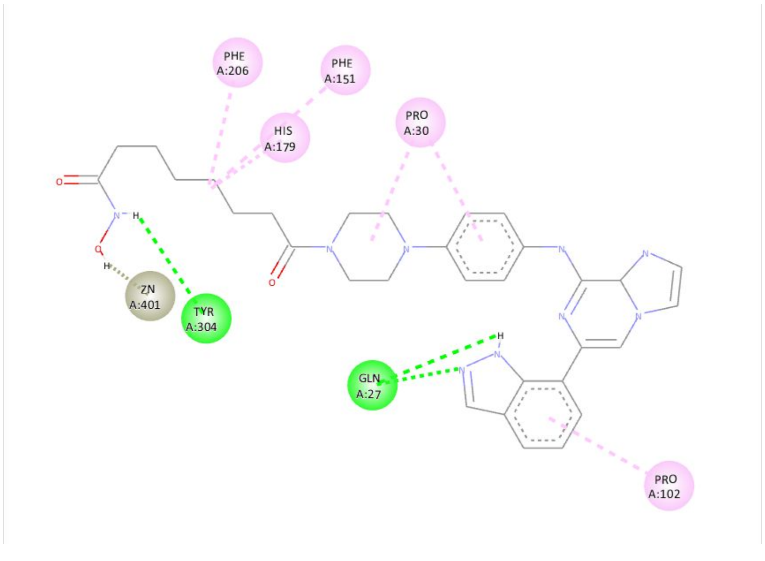

HDAC3

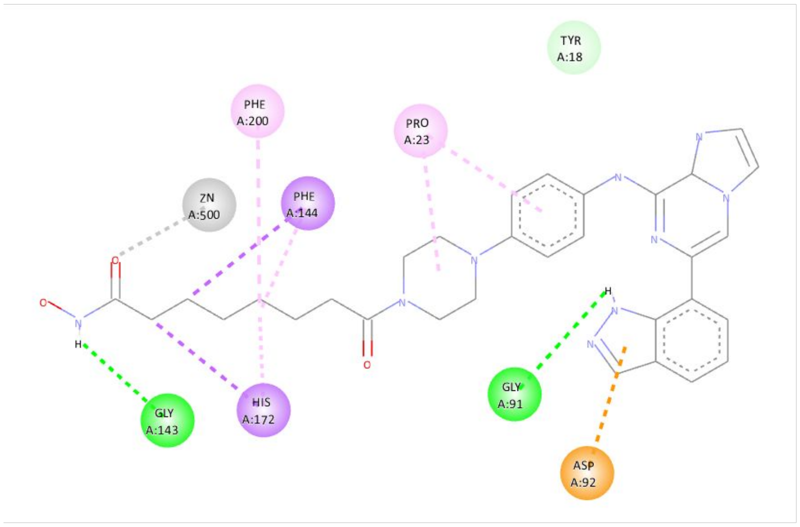

HDAC6

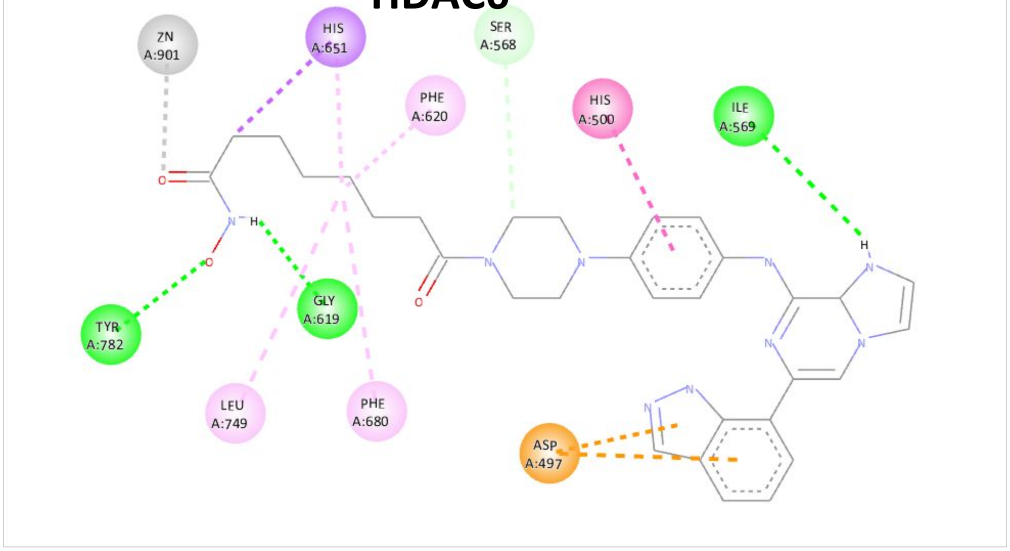

Fig. 5S Docking Analysis of **1** within the active site of **SYK, HDAC1, HDAC2, HDAC3 and HDAC6** (2D docked poses)

6. Docking Study – Compound 14 (SYK)

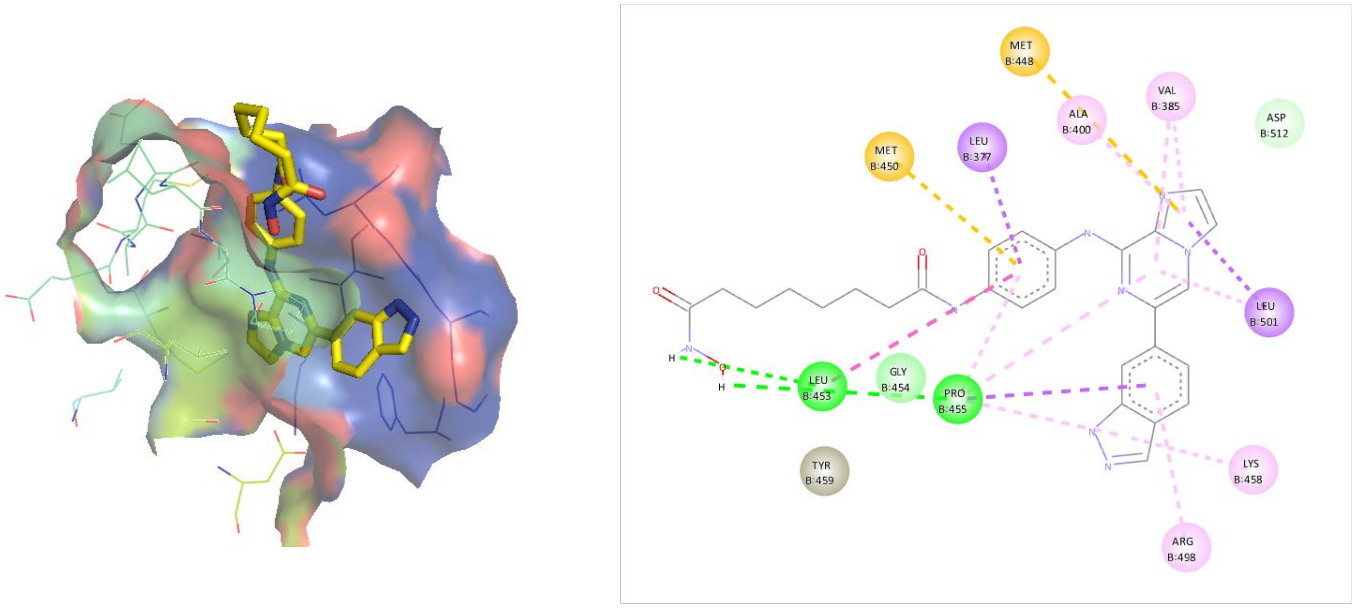

Fig. 6S Interaction analysis of **compound 14** with the amino acid residues of SYK - Orientation of **compound 14** in the active site of the HDAC1 protein and 2D docked pose of **compound 14** showing hydrogen bond and hydrophobic interactions (docking study)

Table 2S. Docking score and residues involved in binding interactions of the **compound 14** with the target proteins.

Protein	ΔG (Std.)	ΔG (Ligand)	Hydrogen bonding (bond distance)	Hydrophobic and other interactions (residues involved)
**SYK**	-8.1	-8.3	Leu453 (2.30 Å), and Pro455 (2.57 Å)	Amide-Pi-Stacked (Leu453), Pi-Sigma (Leu377, Pro455, & Leu501), Pi-Alkyl (Val385, Ala400, Pro455, Lys458, Arg498, & Leu501), and Pi-Sulfur (Met448, & Met450)

7. Docking Study – Compound 14 (HDAC isoforms)

HDAC1

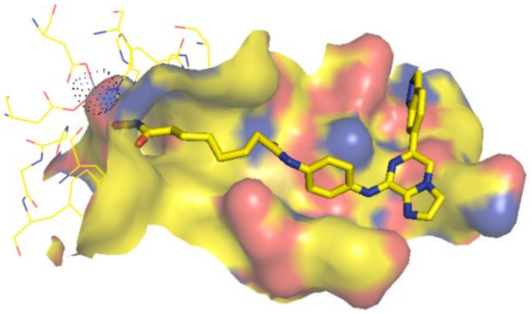

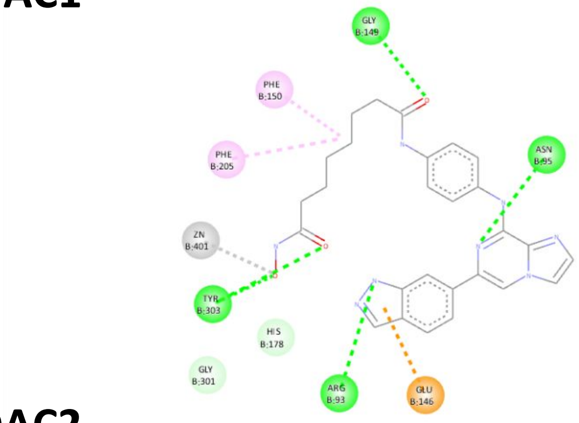

HDAC2

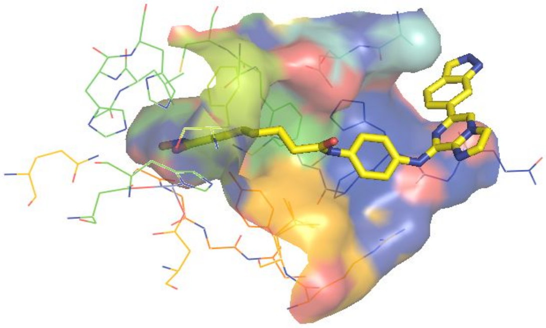

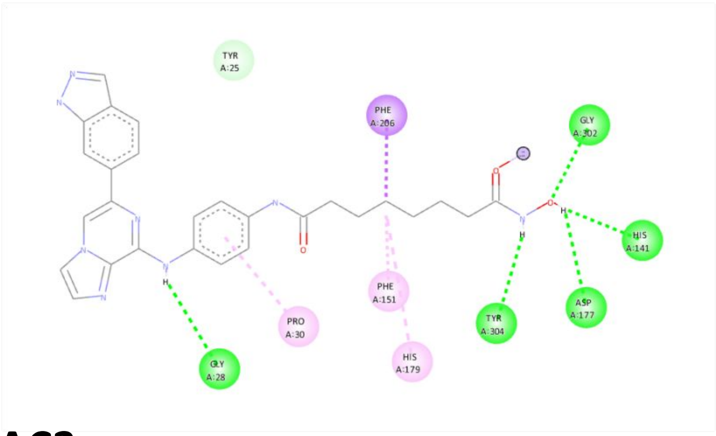

HDAC3

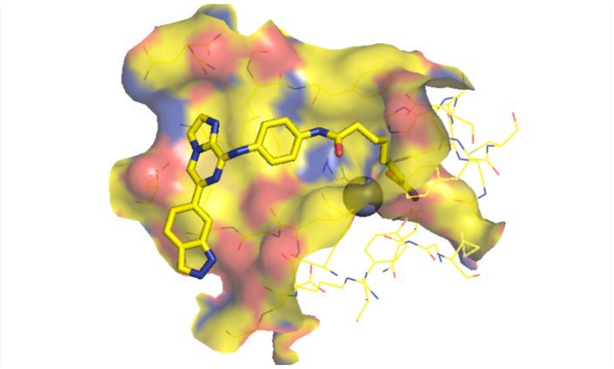

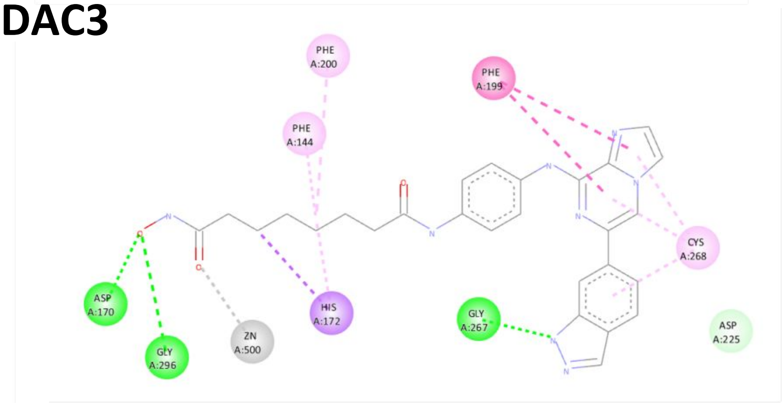

HDAC6

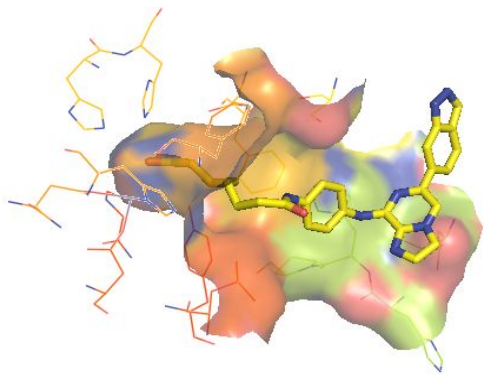

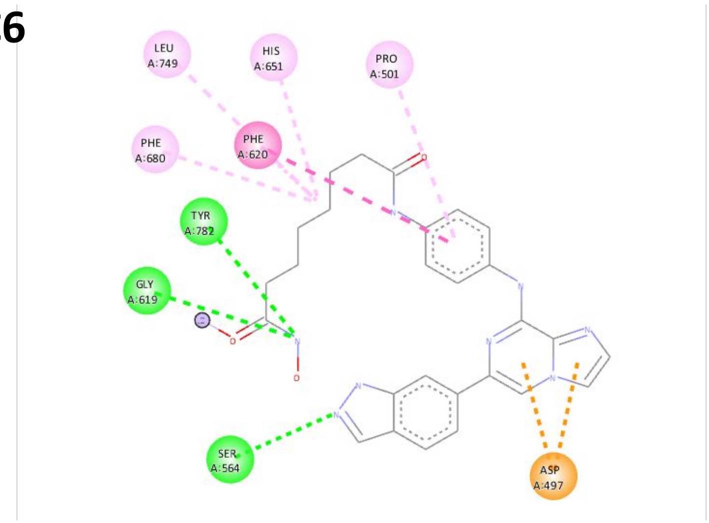

Fig. 7S Interaction analysis of **compound 14** with the amino acid residues of HDAC isoforms (docking study) - Orientation of **compound 14** in the active site of the HDAC isoforms and 2D docked pose of **compound 14**.

Table 3S. Docking score and residues involved in binding interactions of the **compound 14** with the target proteins.

Protein	ΔG (Std.)	ΔG (Ligand)	Hydrogen bonding (bond distance)	Hydrophobic and other interactions (residues involved)
**HDAC1**	-7.7	-8.9	Arg93 (3.16 Å), Asn95 (3.15 Å), Gly145 (2.84, Å), and Tyr303 (2.74, & 3.10 Å)	Pi-Alkyl (Phe150, His178, & Phe205), Pi-Anion (Glu186), and Metal Interaction (Zn401)
**HDAC2**	-9.1	-8.8	Gly28 (3.40 Å), His141 (3.17 Å), Asp177 (3.04), and Gly302 (3.04 Å)	Pi-Alkyl (Pro30, Phe151, & His179), Pi-Sigma (Phe206), and Metal Interaction (Zn401)
**HDAC3**	NA	-8.3	Asp170 (2.92Å), Gly267 (3.11 Å), and Gly296 (3.02 Å)	Pi-Alkyl (Phe144, Phe200, & Cys268), Pi-Sigma (His172), Pi-Pi T-Shaped (Phe199), and Metal Interaction (Zn500)
**HDAC6**	-7.8	-8.1	Ser564 (2.81 Å), Gly619 (3.28 Å), and Tyr782 (2.95 Å)	Pi-Anion (Asp497), Pi-Pi-T-shaped (Phe620), Alkyl (Leu749), Pi-Alkyl (Pro501, Phe620, His651, & Phe680), and Metal Interaction (Zn901)

8. Molecular dynamics (MD)

8.1 Results and discussion

The stability and dynamic behaviour of **compound 14** in complex with HDAC1, HDAC2, HDAC3, HDAC6 and SYK were evaluated by molecular dynamics (MD) simulations. **Table 4S** presents the key parameters observed in the study. All complexes reached equilibrium after an initial equilibration phase, as evident from the RMSD profiles (**Fig. 8S**). Overall structural stability was maintained throughout the simulation; however, varying ligand mobility was observed among the systems. The average RMSD values ranged from 0.20 to 0.24 nm, indicating minimal backbone deviations. Among the systems, SYK demonstrated higher structural rigidity, as evident from the lowest RMSD values, whereas slightly higher fluctuations without compromising overall stability were observed with HDAC6. The results of the RMSF analysis indicated that most residues exhibited (**Fig. 9S**) low to moderate fluctuations, with loop and terminal regions demonstrating higher flexibility. Residues within the binding pocket remained stable across all systems, thereby indicating the contribution of ligand binding to stabilization of the active site. The radius of gyration (Rg) profiles (**Fig. 10S**) aligned with the aforementioned observations and exhibited only minor variations (2.61–2.75 nm). Notably, all protein–ligand complexes maintained compact conformations and any significant unfolding was not observed. Persistent interactions between **compound 14** and the target proteins (2.85 to 3.67 hydrogen bonds) were observed (hydrogen bond analysis, **Fig. 11S**, **Table 4S**).

Quantification of binding affinities was done by MM-PBSA calculations (**Table 5S**). The energy component distributions are illustrated in **Fig. 12S.** Notably, negative binding free energy (ΔG_bind) values were observed for all the complexes, confirming favorable and spontaneous ligand binding. A careful analysis of the studied systems revealed the most favourable binding energy (−33.58 ± 1.88 kcal/mol) with the HDAC6 complex, followed by HDAC1, HDAC2, and HDAC3. Notably, comparable but slightly less favourable binding energetics (−28.94 ± 1.67 kcal/mol) were demonstrated by SYK. In light of these outcomes, it was deduced that ligand binding was primarily driven by van der Waals and electrostatic interactions, while polar solvation contributed unfavorably due to desolvation penalties. Also, a modest stabilizing effect was attributed to non-polar solvation. Collectively, these results are indicative of effective engagement of both SYK and HDAC targets by **compound 14** with complementary binding characteristics. Greater conformational rigidity and lower backbone deviations were observed with SYK, while the HDAC isoforms, particularly HDAC6, exhibited more favorable binding energetics. In a nutshell, molecular dynamics and MM-PBSA outcomes support the potential of **compound 14** as a dual-target ligand against SYK and HDACs.

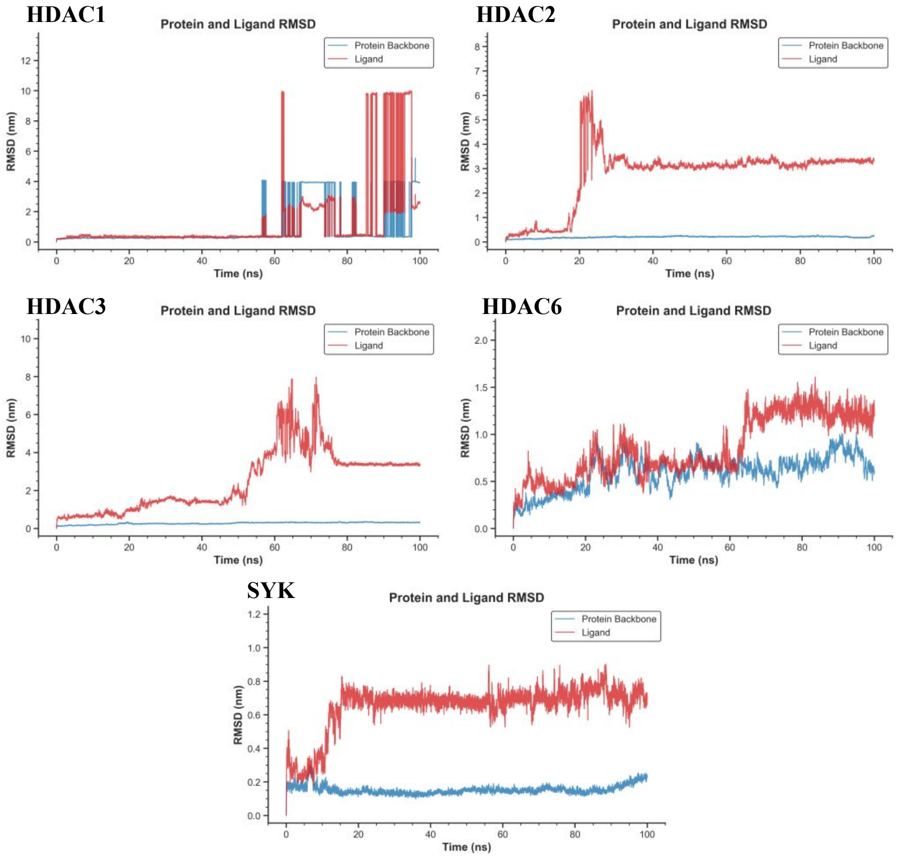

Fig. 8S RMSD profiles of protein-ligand complexes (HDAC1, HDAC2, HDAC3, HDAC6, and SYK)

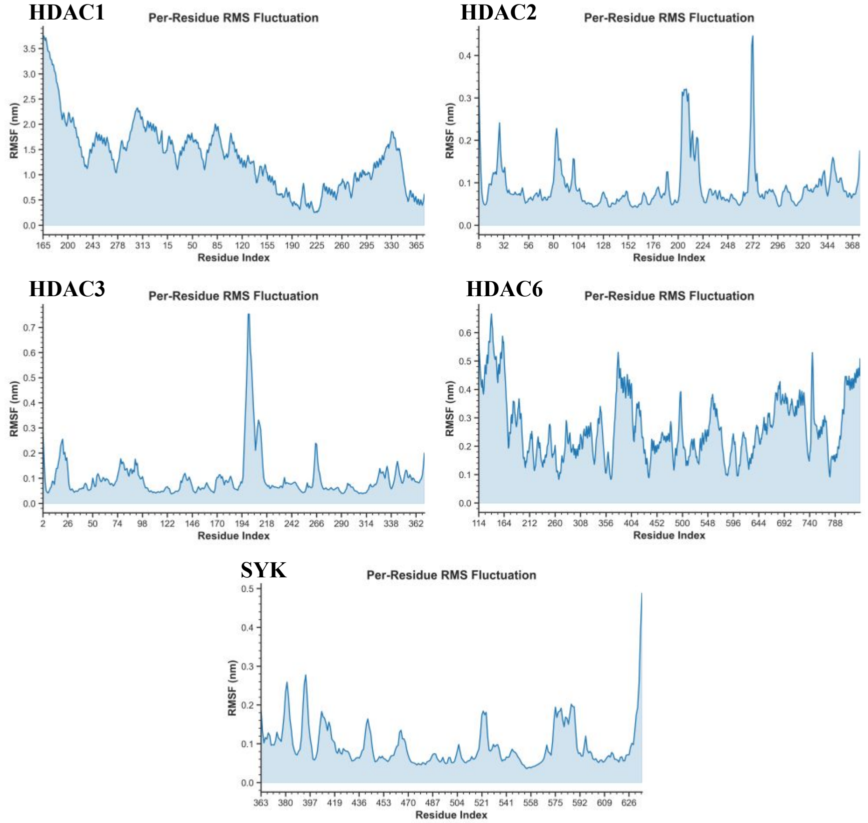

Fig. 9S RMSF of amino acid residues for all protein-ligand complexes (HDAC1, HDAC2, HDAC3, HDAC6, and SYK)

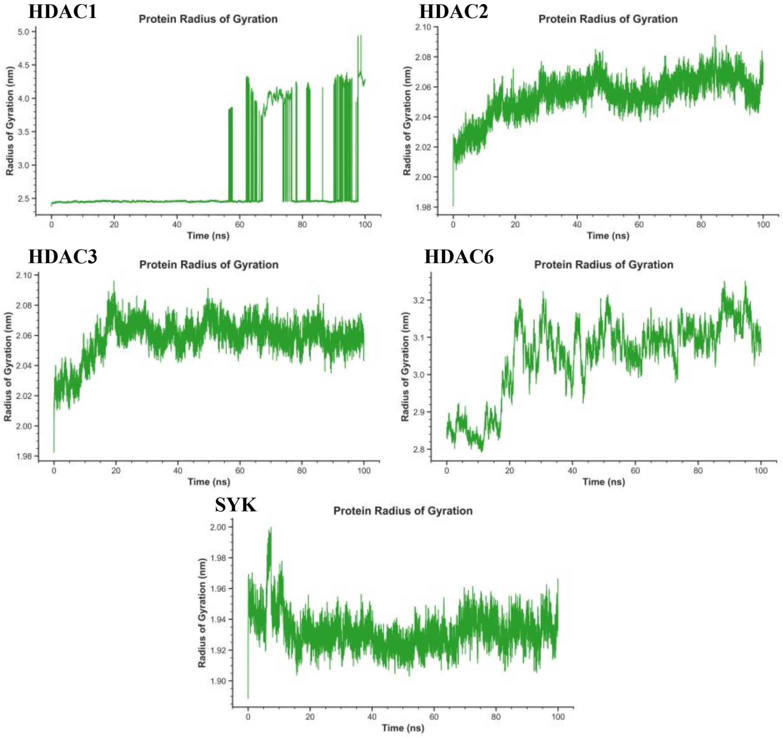

Fig. 10S Rg plots of protein-ligand complexes showing maintenance of compact structural conformations throughout the simulation period, with minor fluctuations corresponding to natural protein dynamics.

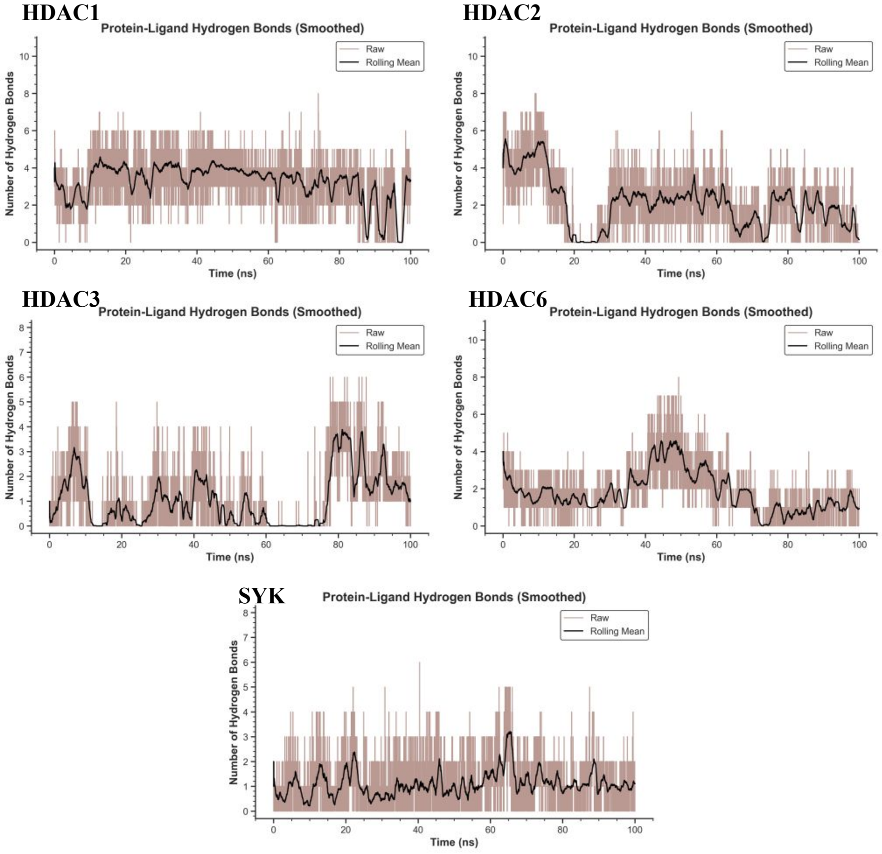

Fig. 11S Number of H-bonds formed between **compound 14** and protein (HDAC1, HDAC2, HDAC3, HDAC6, and SYK) over the simulation time.

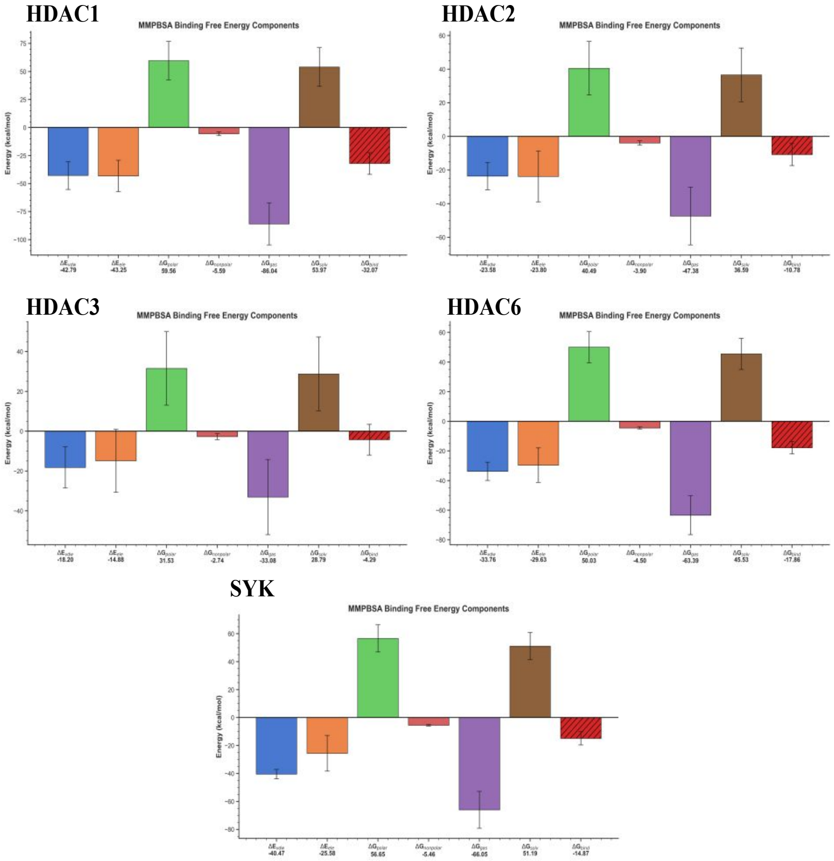

Fig. 12S MM-PBSA binding free energy components (van der Waals, electrostatic, polar solvation, and non-polar solvation) for all complexes, highlighting dominant contributions of van der Waals and electrostatic**.**

Table 4S. MD simulation parameters, including RMSD, RMSF, Rg, and average number of H-bonds for all protein-ligand complexes.

System	RMSD (nm)	RMSF (nm)	Rg (nm)	H-bonds (avg)
**HDAC1**	0.21 ± 0.02	1.34 ± 0.65	2.72 ± 0.08	3.21 ± 1.32
**HDAC2**	0.23 ± 0.03	1.28 ± 0.60	2.69 ± 0.07	3.45 ± 1.28
**HDAC3**	0.22 ± 0.02	1.22 ± 0.58	2.64 ± 0.06	2.98 ± 1.21
**HDAC6**	0.24 ± 0.03	1.30 ± 0.62	2.75 ± 0.09	3.67 ± 1.40
**SYK**	0.20 ± 0.02	1.18 ± 0.55	2.61 ± 0.06	2.85 ± 1.15

Table 5S. MM-PBSA binding free energy (ΔG_bind) and its individual energy components for **compound 14** in complex with target proteins.

System	ΔG_bind (kcal/mol)	van der Waals	Electrostatic	Polar Solvation	Non-polar
**HDAC1**	−32.07 ± 1.80	−42.79	−43.25	+49.12	−5.15
**HDAC2**	−30.84 ± 1.95	−40.65	−41.18	+46.87	−5.88
**HDAC3**	−29.76 ± 1.72	−38.92	−39.74	+44.95	−6.05
**HDAC6**	−33.58 ± 1.88	−44.36	−45.02	+51.20	−5.40
**SYK**	−28.94 ± 1.67	−37.85	−36.91	+42.73	−6.91

8.2 Experimental

8.2.1 Molecular Dynamics (MD) Simulation

The stability and dynamic behaviour of **compound 14** in complex with target proteins (HDAC1, HDAC2, HDAC3, HDAC6, and SYK) was evaluated using MD simulations.[1] Structural and dynamic parameters, including root mean square deviation (RMSD), root mean square fluctuation (RMSF), radius of gyration (Rg), and hydrogen bonding, were calculated using built-in tools of GROMACS.[1-6] Statistical analyses and graphical representations were performed using NumPy,[7] Pandas,[8] and Seaborn libraries. [9]

8.2.2 Binding Free Energy Calculations (MM-PBSA)

MMPBSA tool was used for the binding free energy calculations to estimate the interaction strength between **compound 14** and target proteins.[10-12]

8.3 References

(1) Abraham, M. J.; Murtola, T.; Schulz, R.; Páll, S.; Smith, J. C.; Hess, B.; Lindahl, E., GROMACS: High performance molecular simulations through multi-level parallelism from laptops to supercomputers. *SoftwareX* **2015,** *1* (2), 19-25.

(2) Huang, J.; MacKerell Jr, A. D., CHARMM36 all-atom additive protein force field: Validation based on comparison to NMR data. *J. Comput. Chem.* **2013,** *34* (25), 2135-2145.

(3) Vanommeslaeghe, K.; Hatcher, E.; Acharya, C.; Kundu, S.; Zhong, S.; Shim, J.; Darian, E.; Guvench, O.; Lopes, P.; Vorobyov, I., CHARMM general force field: A force field for drug-like molecules compatible with the CHARMM all-atom additive biological force fields. *J. Comput. Chem.* **2010,** *31* (4), 671-690.

(4) Jorgensen, W. L.; Chandrasekhar, J.; Madura, J. D.; Impey, R. W.; Klein, M. L., Comparison of simple potential functions for simulating liquid water. *J. Chem. Phys.* **1983,** *79* (2), 926-935.

(5) Darden, T.; York, D.; Pedersen, L., Particle mesh Ewald: An N log (N) method for Ewald sums in large systems. *J. Chem. Phys.* **1993,** *98* (12), 10089-10092.

(6) Hess, B.; Bekker, H.; Berendsen, H. J.; Fraaije, J. G., LINCS: A linear constraint solver for molecular simulations. *J. Comput. Chem.* **1997,** *18* (12), 1463-1472.

(7) Easton, J. M.; Harris, L. M.; Viant, M. R.; Peet, A. C.; Arvanitis, T. N., Linked Metabolites: A tool for the construction of directed metabolic graphs. *Comput. Biol. Med.* **2010,** *40* (3), 340-349.

(8) McKinney, W., Data structures for statistical computing in Python. *scipy* **2010,** 445 (1), 51-56.

(9) Waskom, M. L., Seaborn: statistical data visualization. *J. Open Source Softw.* **2021,** *6* (60), 3021.

(10) Valdés-Tresanco, M. S.; Valdés-Tresanco, M. E.; Valiente, P. A.; Moreno, E., gmx_MMPBSA: a new tool to perform end-state free energy calculations with GROMACS. *J. Chem. Theory Comput.* **2021,** *17* (10), 6281-6291.

(11) Maier, J. A.; Martinez, C.; Kasavajhala, K.; Wickstrom, L.; Hauser, K. E.; Simmerling, C., ff14SB: improving the accuracy of protein side chain and backbone parameters from ff99SB. *J. Chem. Theory Comput.* **2015,** *11* (8), 3696-3713.

(12) Wang, J.; Wolf, R. M.; Caldwell, J. W.; Kollman, P. A.; Case, D. A., Development and testing of a general amber force field. *J. Comput. Chem.* **2004,** *25* (9), 1157-1174.

9. Assessment of the expression levels of p21, an HDAC downstream target, and p-PLCγ2, a SYK downstream target, following treatment with compound 14.

The impact of **compound 14** treatment on the expression levels of p21, acetylated α-tubulin and acetylated histone H3 was assessed in MV4-11 cells by western blot after 24 h treatment. It was observed that **compound 14** treatment led to upregulation in the expression levels of acetylated α-tubulin and acetylated histone H3 in a concentration-dependent manner, thereby supporting its HDAC inhibitory potential. Concomitantly, **compound 14** treatment exerted an increase in p21 expression (**HDAC downstream target**), with maximal upregulation observed at the concentrations of 0.5 and 1 μM. Notably, a decrease in p21 levels was observed with 2.5 μM of **compound 14**, reflecting enhanced apoptosis at higher concentrations. It is important to mention that the FDA-approved HDAC inhibitor, SAHA, elicited a similar expression modulatory pattern (acetyl tubulin, acetyl histone and p21) (**Fig. 13S**). Also, the effect of **compound 14** on the phosphorylation levels of SYK and its downstream effector PLCγ was evaluated in MV4-11 cells by western blot **(Fig. 14S)**. The experiment was performed under oxidative stress conditions and hydrogen peroxide (20 mM) was used to increase the phosphorylation levels of SYK and its downstream effector PLCγ2. Under the aforementioned conditions, the total protein levels remained unchanged. Notably, **compound 14** treatment led to a reduction in p-SYK and p-PLCγ2 levels in a dose-dependent manner, thereby indicating inhibition of SYK signaling.

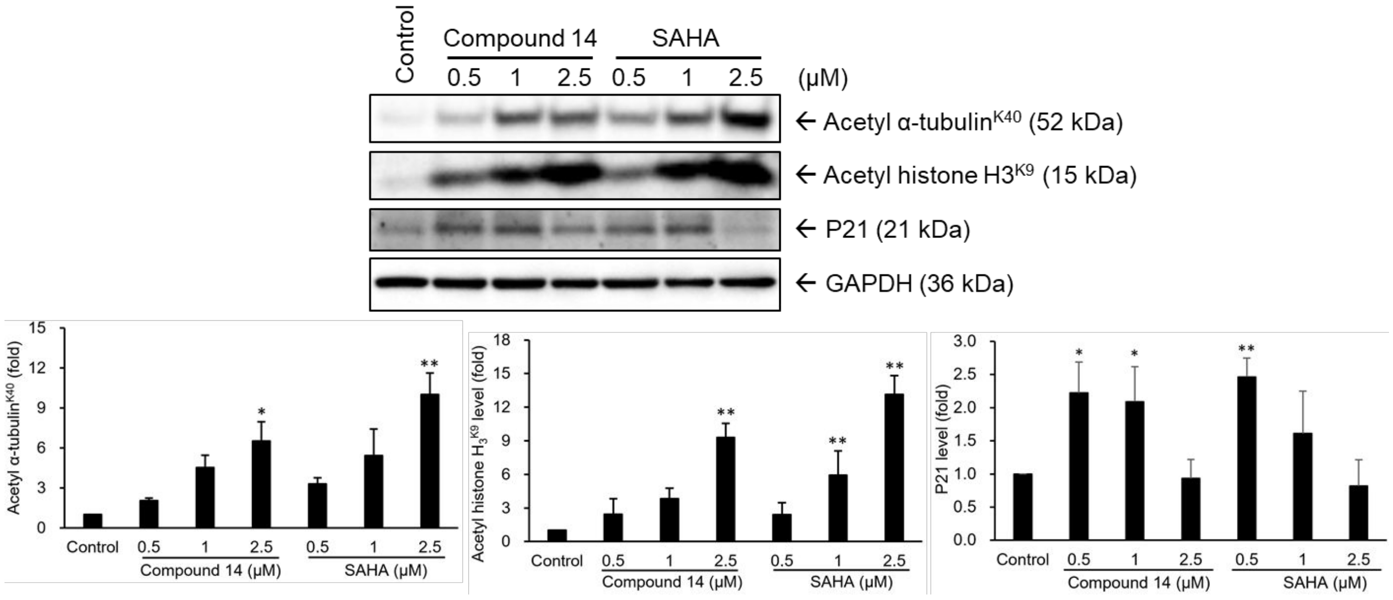

Fig. 13S The effect of **compound 14** on the level of P21 and the acetylation of α-tubulin and histone H3 in MV4-11 cells. Cells were treated with **compound 14** or SAHA for 24 hr and then harvested for Western blot analysis g. * $p < 0.05$ and ** $p < 0.01$ compared to the control group.

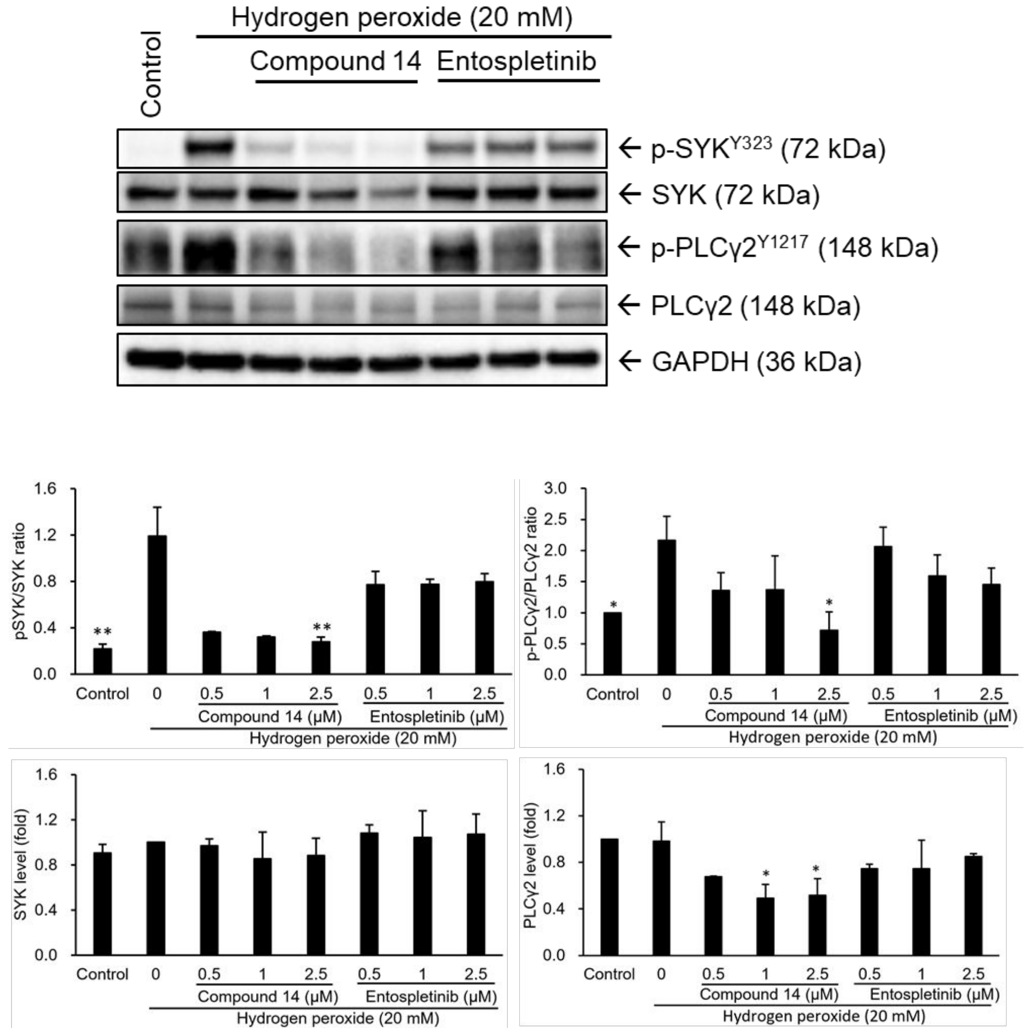

Fig. 14S. The effect of **compound 14** on total and phosphorylated levels of SYK and PLCγ2 in MV4-11 cells. Cells were treated with **compound 14** or Entospletinib for 24 hr and then co-incubated with hydrogen peroxide for 30 min. Afterward, the cells were harvested for Western blot analysis. ** *p* <0.01 compared to the group treated with 20 mM hydrogen peroxide alone.

10. Pharmacokinetic characterization of compound 14

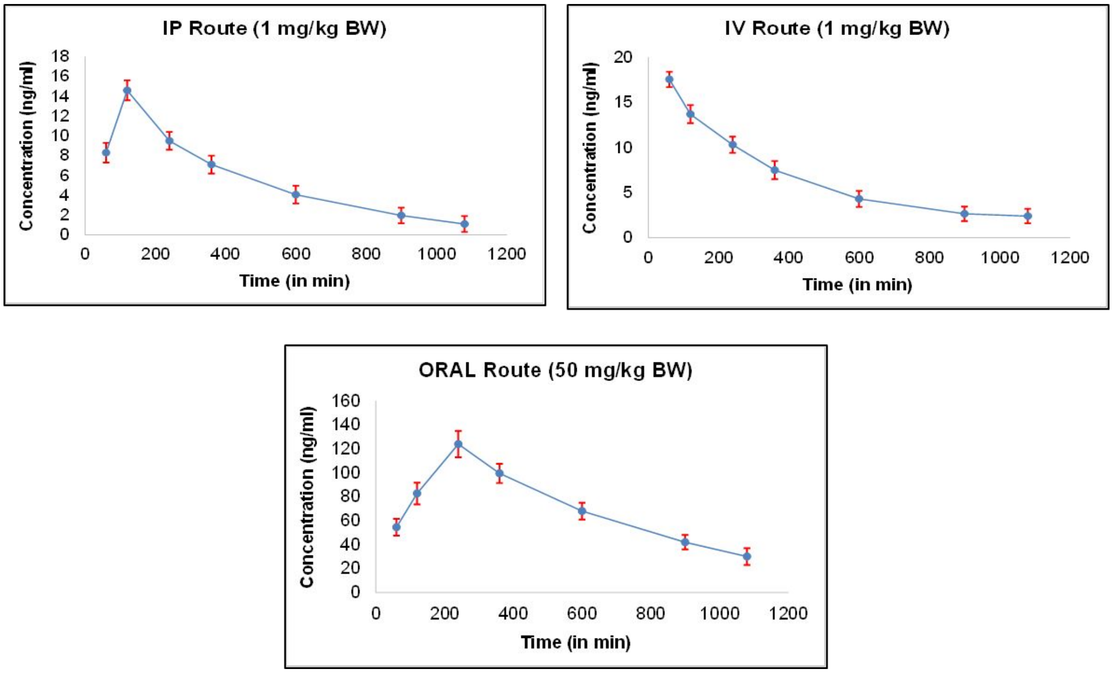

Fig. 15S Time vs concentration graphs after the administration of **compound 14**.

11. *In-vitro* assessment-antitumor activity of compound 14 against HL-60 cell lines

11.1 *In-vitro* cytotoxicity

Given the strategy of piperazine truncation justified and found to be fruitful through the experimental results, the piperazine truncated compounds were evaluated for the *in-vitro* cytotoxicity effects against HL-60 cell lines **(Table 6S)**. Notably, HL-60 cell lines were employed to capture the biological heterogeneity of AML and assess the impact of the compounds on the cell growth of AML cell lines that do not harbor FLT3-ITD mutation. Resultantly, it was observed that **compound 13-17** exhibited superior antiproliferative effects against HL60 cell lines than the standards (SAHA and Entospletinib), thereby justifying our scaffold construction strategy. In particular, **compound 14,** endowed with the most potent cell growth inhibitory effects against MV4-11 cell lines, also exerted significant cytotoxicity against HL-60 cell lines. Important to mention that linker variations were found to be impactful in the context of cytotoxicity of compounds, as **compound 10-12** bearing a methylene chain (n =2-4) exerted moderate cell growth inhibitory effects against HL-60 cell lines. Installation of the benzyl acrylamide unit as a linker, culminating in **compound 18,** also could not outperform the standards in terms of cytotoxicity.

Table 6S. *In-vitro* cytotoxicity studies of the synthesized compounds against MV4-11 cells

**Compound**	**HL-60 cells ($IC_{50,}$ µM)[a]**
10	4.908 ± 0.484
11	3.383 ± 0.554
**12**	3.143 ± 0.722
**13**	1.464 ± 0.190
**14**	**0.909 ± 0.494**
**15**	**1.359 ± 0.681**
**16**	**1.281 ± 0.281**
**17**	**1.831 ± 0.293**
18	2.48 ± 0.938
SAHA	2.343 ± 0.171
Entospletinib	5.304 ± 0.326

[a]SD: standard deviation. All experiments were independently performed at least three times (*n* = 3) to determine the mean and SD.

11.2 Western blot analysis to assess the impact of compound 14 on expression levels of acetyl histone, acetyl tubulin and p-SYK

Western blot analysis was performed to assess the impact of **compound 14** on the expression levels of acetyl and acetyl tubulin in HL60 cells. Resultantly, it was observed that **compound 14** exerted upregulation of acetylated α-tubulin and acetylated histone H3 in a concentration-dependent manner (**Fig. 16S**). In addition, **compound 14** treatment also led to a reduction in p-SYK levels **(Fig. 17S).** These outcomes are indicative of the dual modulatory ability (SYK-HDAC) of **compound 14**.

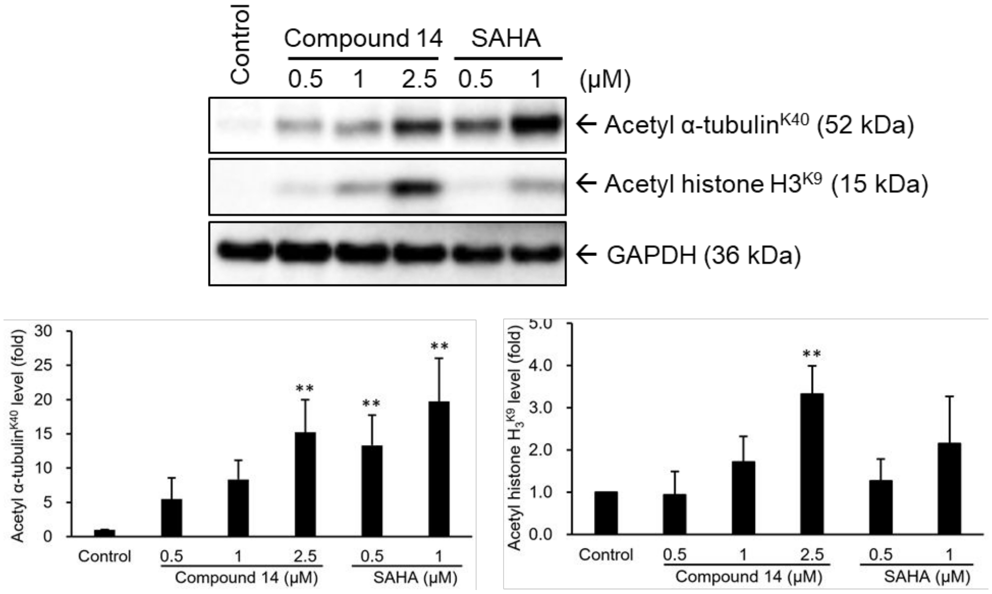

Fig. 16S The effect of **compound 14** on the acetylation of α-tubulin and histone H3 in HL60 cells.

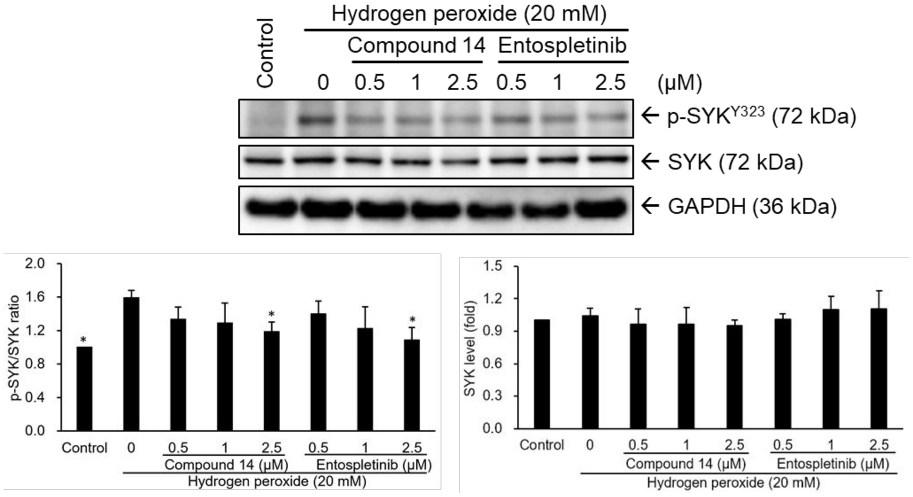

Fig. 17S The effect of **compound 14** on the total and phosphorylated levels of SYK in HL60 cells.

11.3 Cell Cycle Analysis, Annexin V-PI assay, western blot analysis to assess apoptosis and autophagy-inducing ability of compound 14

Flow cytometry was performed to evaluate the effect of **compound 14** on cell cycle progression (HL60 cells). The results indicated that **compound 14** exerted a concentration-dependent redistribution of cells. Specifically, a notable decrease in the G0/G1 population with marked S phase arrest was observed. Also, **compound 14** at the concentrations of 2.5 and 5 μM led to an increase in the sub-G1 fraction, thereby indicating the apoptosis-inducing ability of **compound 14** (**Fig. 18S**). Further, Annexin V–FITC/PI double staining was performed to assess the pro-apoptotic effect of **compound 14** in HL60 cells. It was observed that **compound 14** treatment exerted a concentration-dependent increase in apoptotic cells, as evidenced by a progressive rise in both early (Annexin V^+/PI^-) and late (Annexin V^+/PI^+) populations. Noteworthy to mention that, **compound 14** at a concentration of 5 μM increased late apoptotic and non-viable cell populations (**Fig. 19S**). Further, the western blot analysis results revealed a concentration-dependent upregulation of cleaved caspase-3 (17 kDa), indicative of the apoptosis-inducing ability of **compound 14.** Also, an increase in LC3-II levels and a decrease in p62 expression were observed with **compound 14** treatment, indicative of the potential of **compound 14** as an autophagy inducer (**Fig. 20S**). DAPI staining was also performed to validate the induction of apoptosis with **compound 14** treatment. Control cells exhibited uniformly distributed, round nuclei with diffuse staining, indicative of intact chromatin and normal cellular morphology. On the contrary, notable changes in nuclear architecture, *viz.* chromatin condensation and nuclear fragmentation, were observed with **compound 14** treatment. Important to mention that the aforementioned effects were demonstrated by **compound 14** in a concentration-dependent manner. Overall, these findings confirm the ability of **compound 14** as an apoptosis inducer (**Fig. 21S A).** Rhodamine staining revealed a dose-dependent reduction in mitochondrial membrane potential ($\Delta\Psi m$) with **compound 14** treatment. Notably, intact mitochondrial function, evidenced by strong green fluorescence was observed with control cells, whereas **compound 14**-treated cells exhibited a progressive decrease in fluorescence intensity. Overall, these findings suggest the involvement of mitochondrial depolarization in **compound 14**-induced apoptosis (**Fig. 21S B).**

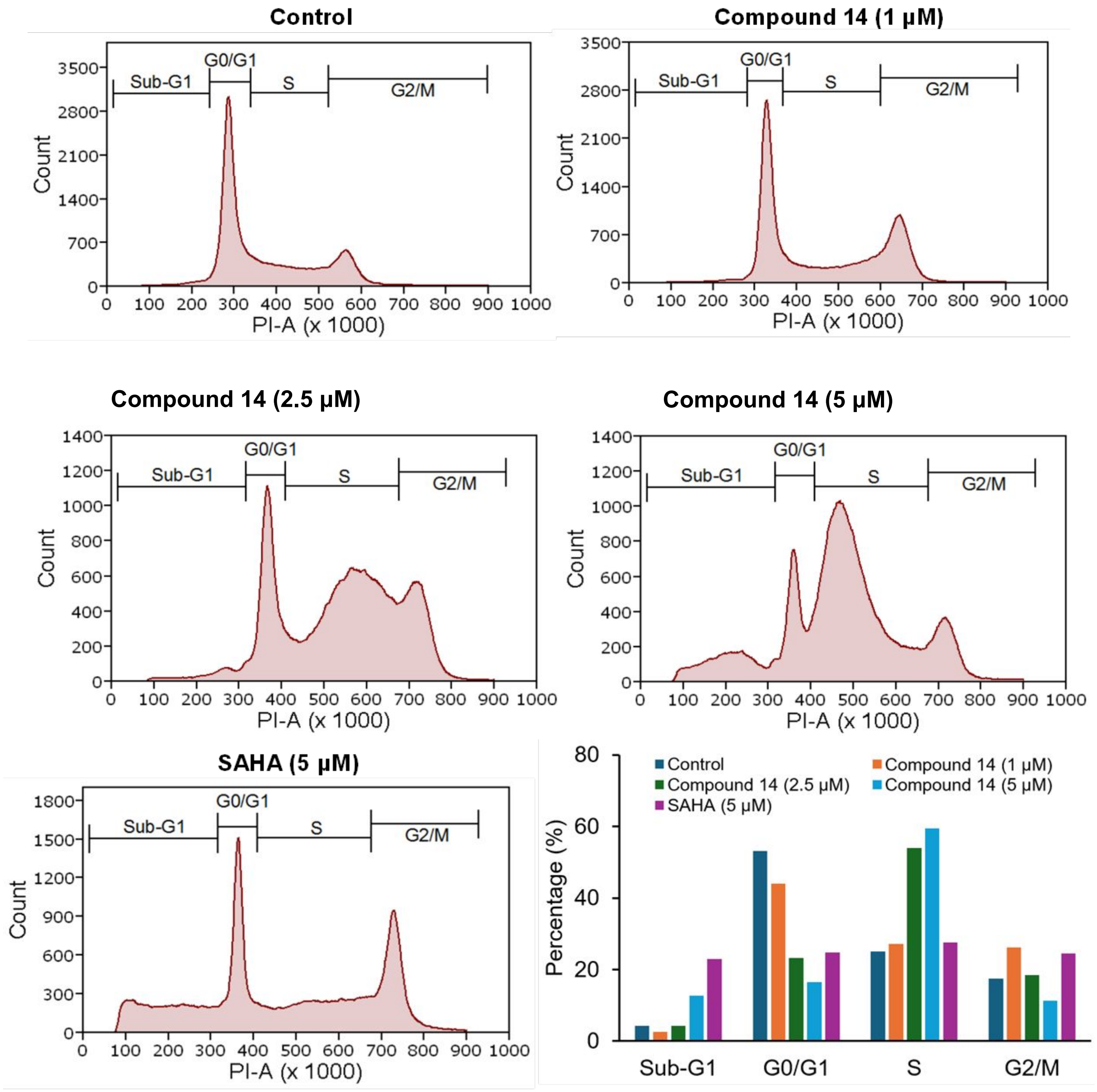

Fig. 18S The impact of compound 14 on the distribution of cell cycle phases in HL60 cells. The cells were incubated with **compound 14** or SAHA for 24 hr. After treatment, cells were harvested, and the distribution of cell-cycle phases was analyzed by flow cytometry.

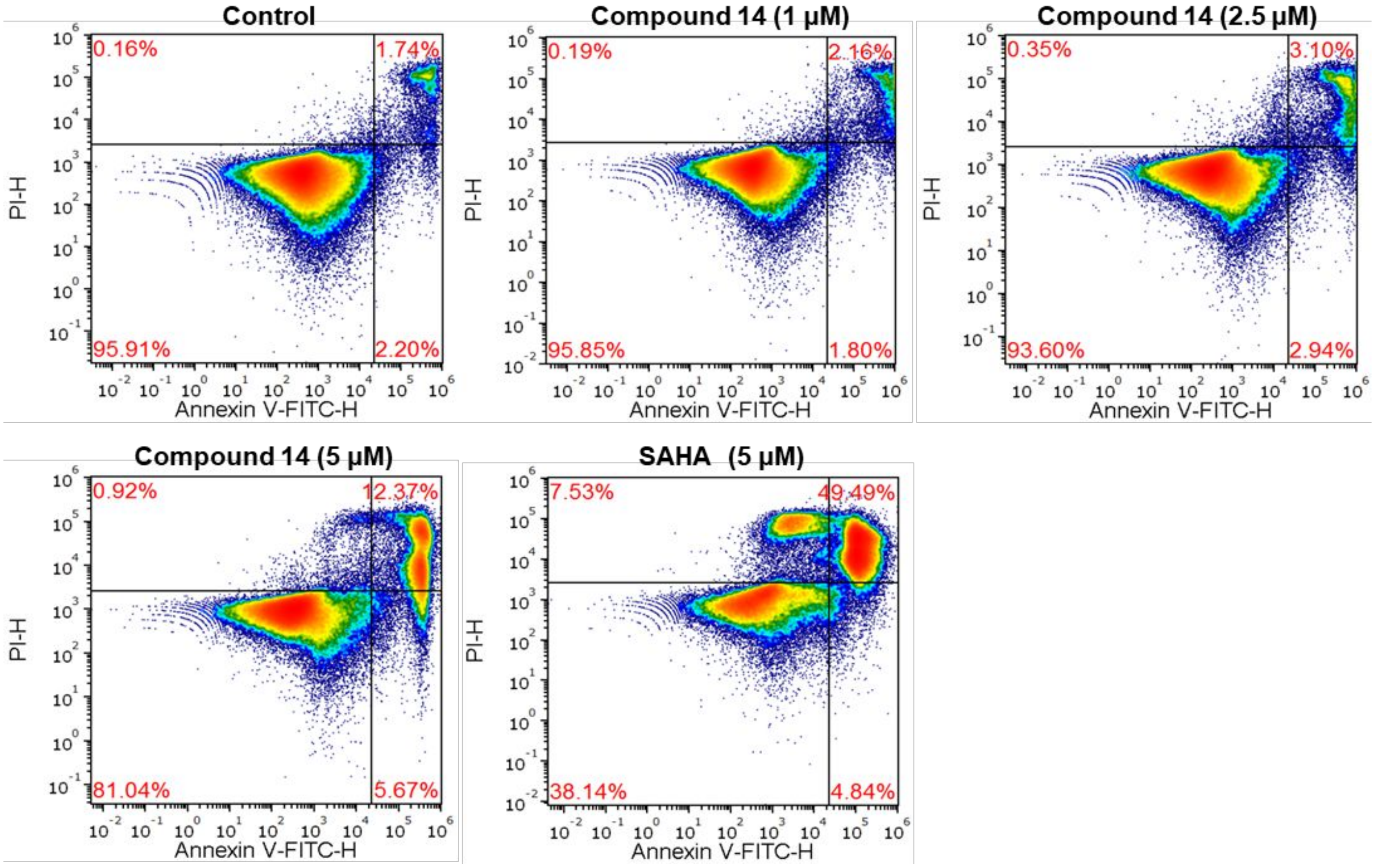

Fig. 19S The effect of compound 14 on inducing apoptosis in HL60 cells. Cells were incubated with **compound 14** or SAHA for 24 hr. After the treatment, cells were harvested and stained using PI and annexin V-FITC for 30 min. The apoptotic cells were subsequently identified using flow cytometry.

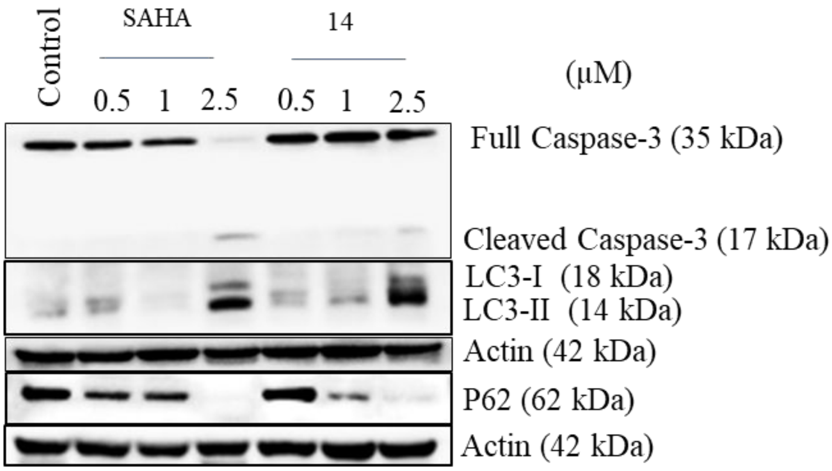

Fig. 20S Western blot analysis to determine the expression level of cleaved caspase 3, p62 and LC3-II with **compound 14** treatment in HL60 cells

A)

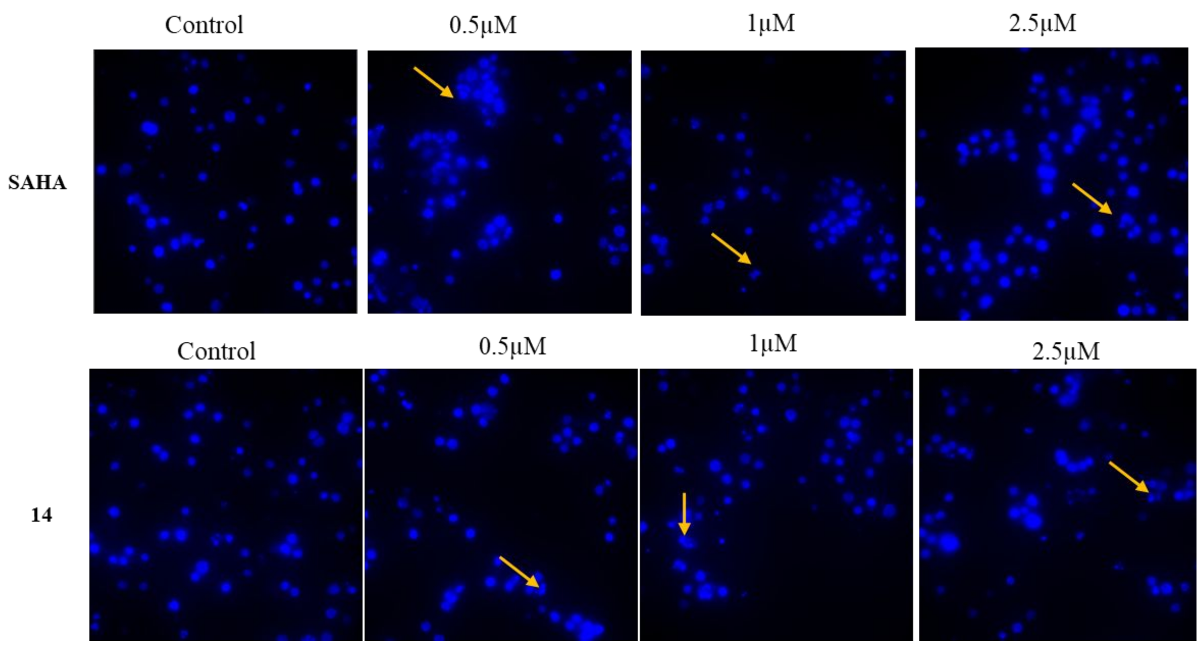

B)

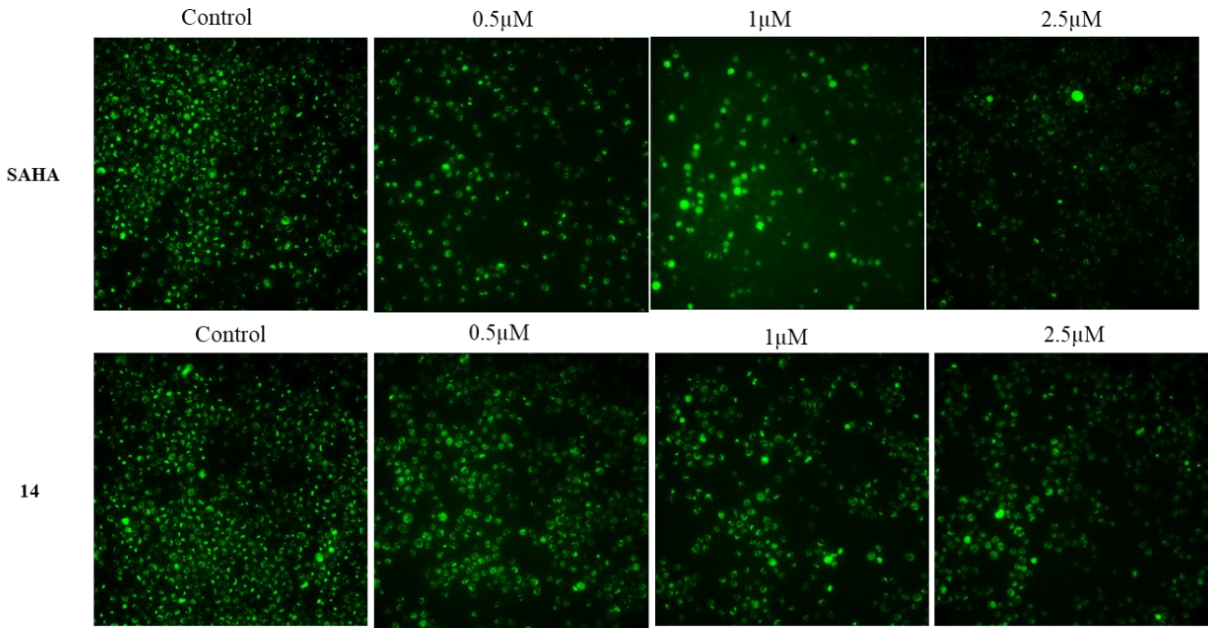

Fig. 21S (A) Analysis of nuclear morphology by DAPI staining in HL-60 cells. (B) Evaluation of mitochondrial membrane potential by rhodamine 123 staining in HL-60 cells.

12. Anti-leukemic activity in leukemia-induced NOD SCID mice model

12.1 Results

12.1.1 Anti-leukemic effect assessment of compound 14

The anti-leukemic efficacy of **compound 14** was evaluated in a systemic leukemia model. The model was developed in NOD/SCID mice via intravenous inoculation of human MV4-11 leukemia cells. The establishment of leukaemia was confirmed by microscopic examination of peripheral blood smears (days 14 and 20 post-inoculation), which confirmed a progressive increase in circulating leukemic blast cells. Clear differences among the treatment groups were observed on subsequent cytological examination of Giemsa-stained blood smears collected on days 28 and 36 (**Fig. 22S A**). Notably, an extensive leukemic infiltration with a high density of blast cells was observed in the disease control group, which indicated aggressive disease progression. On the contrary, significant reductions in circulating leukemic cells were observed in both SAHA and compound 14-treated groups, with **compound 14** exhibiting a greater anti-leukemic efficacy in comparison to SAHA. These results are corroborated by the outcomes of quantitative blast cell analysis based on morphological assessment of Giemsa-stained smears combined with Trypan Blue exclusion to determine viable leukemic cells (**Fig. 22S B**). Quantification analysis revealed a marked reduction in the leukemic cell burden with **compound 14** treatment, compared to both disease control and SAHA-treated animals, thereby confirming the *in-vivo* anti-leukemic activity of **compound 14**.

12.1.2 Hematological, hepatic, and renal biochemical analysis

Subsequently, hematological, hepatic, and renal biochemical analysis were conducted in the experimental animals to assess the systemic therapeutic efficacy of **compound 14.** Pronounced hematological abnormalities consistent with leukemia-associated marrow dysfunction (**Table 7S**) were observed in the disease control, with a significant reduction in hemoglobin concentration, RBC count, hematocrit, and platelet count and elevation in total leukocyte count, indicating anemia, thrombocytopenia, and leukocytosis. The results of the hematological analysis revealed that **compound 14** exerted a notable corrective effect, as evidenced by a significant increase in hemoglobin, RBC count, hematocrit, and platelet levels. Also, **compound 14** led to reductions in total leukocyte count toward normal values. The aforementioned improvements were also observed with SAHA; however, **compound 14** was found to be superior in terms of restoration of hematopoietic function as well as suppression of leukemic burden. Serum biochemical analysis revealed elevated bilirubin, AST (SGOT), ALT (SGPT) and reduced albumin levels (hepatic impairment) in the control group (**Table 8S**). The outcome of this analysis demonstrated moderate improvement in these parameters with SAHA, whereas significant reductions in bilirubin and transaminase levels, as well as restoration of serum albumin were observed in the **compound 14 treatment** group, leading to greater normalization indicative of improved hepatic status and reduced disease-associated liver injury. Kidney function test showed renal dysfunction characterized by elevated creatinine, blood urea nitrogen (BUN), urea, and uric acid levels was observed in the disease control group. Aligning with the trends of the liver function

analysis, the outcome of this study also indicated better efficacy of **compound 14** in comparison to SAHA treatment, in the context of significant reductions in all measured renal biomarkers. In light of these results, it is deduced that **compound 14** improves systemic physiological status and preserves vital organ function in addition to exerting strong anti-leukemic activity.

12.1.3 Body weight and survival Analysis

Body weight analysis showed a progressive decline in the control group, indicating severe disease progression and worsening leukemia burden (**Fig. 23S A**). Notably, stabilization of body weight following therapy initiation was observed with both SAHA and **compound 14**; however, SAHA-treated animals showed moderate recovery, whereas substantial improvement was observed with **compound 14,** leading to body weights approaching baseline values by the end of the study. Kaplan–Meier survival analysis demonstrated reduced survival in the disease control group compared with both treated groups (**Fig. 23S B**). Both **treatment groups** showed improved survival relative to disease control during the observation period. On the whole, **compound 14** was well tolerated based on body weight maintenance and biochemical parameters.

12.1.4 Histopathological Evaluation

Histopathological examination of kidney, liver, and spleen tissues was conducted, with the disease control group exhibiting severe leukemic infiltration with marked disruption of splenic architecture, lymphoid depletion, hepatic cord disorganization, sinusoidal infiltration, and mild renal damage (**Fig. 23S C**). A partial restoration of tissue architecture and reduced leukemic burden was observed with the SAHA treatment group, whereas **compound 14** led to more significant reduction in cellular infiltration. Also, **compound 14** markedly preserved tissue architecture in all examined organs, thereby demonstrating a better safety profile.

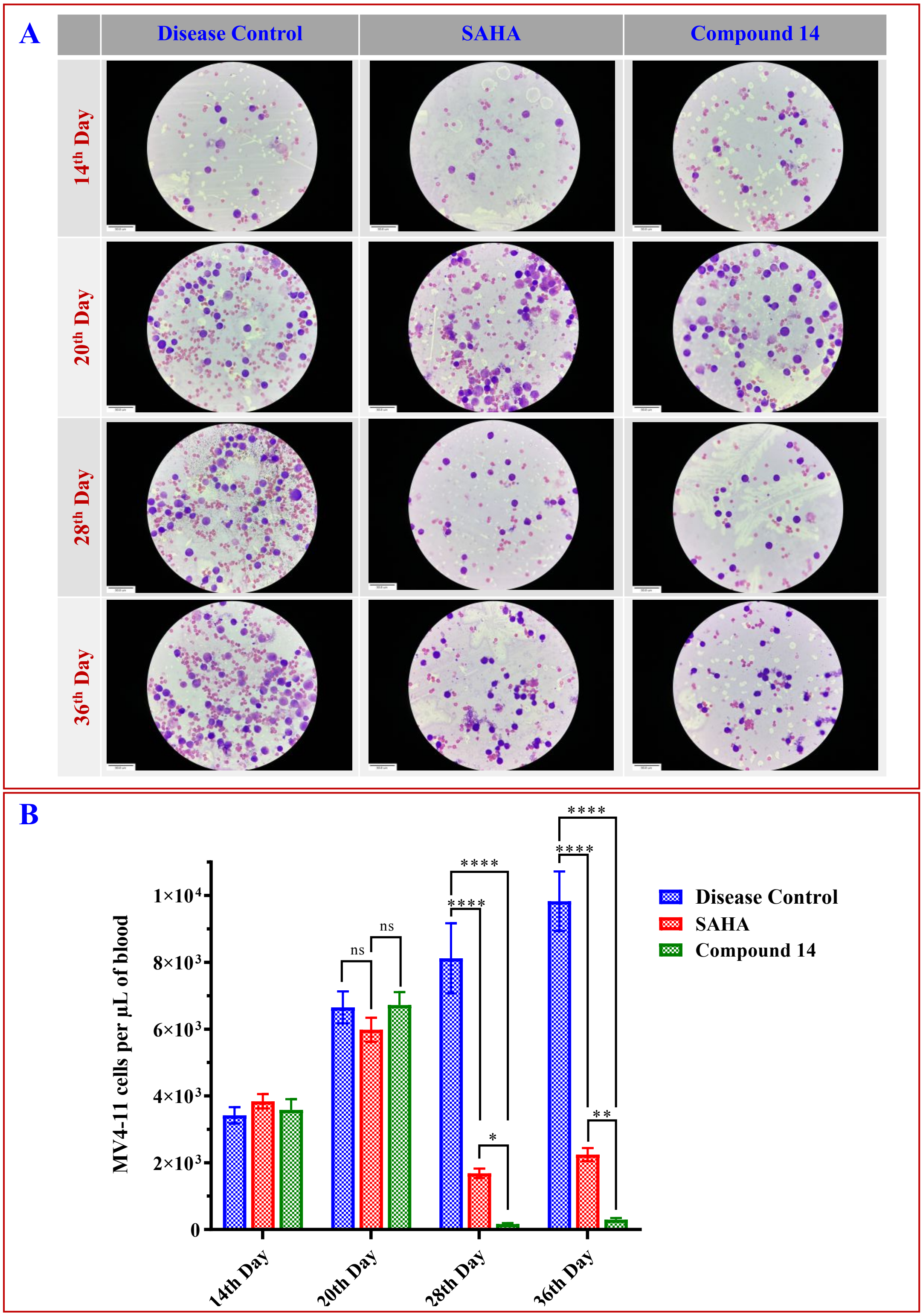

Fig. 22S (A) Cytology images of blood samples collected after 14, 20, 28, and 36 days of leukaemia induction. (B) Blast cell count in blood samples collected after leukemia induction. Data are presented as mean ± SD (*$p < 0.05$, **$p < 0.01$, ****$p < 0.0001$; ns, not significant). **Compound 14** exerted notable reduction in circulating blast cell counts in comparison to the disease control and SAHA-treated groups at later stages of leukemia progression.

Table 7S. Comparative haematological analysis in leukaemia-induced NOD SCID mice. Values are expressed as mean ± SD. *, **, ***, and ns signify $p < 0.05$, $p < 0.01$, $p < 0.001$, and not significant, respectively. Statistical significance symbols represent comparisons between **compound 14** and the SAHA-treated group.

Parameter (Units)	Disease Control	SAHA	Compound 14
Hemoglobin (g/dL)	8.5 ± 0.4	10.2 ± 0.5	11.3 ± 0.6^{ns}
RBC Count (10^6/µL)	5.2 ± 0.3	6.4 ± 0.4	7.5 ± 0.3*
Hematocrit (%)	28.0 ± 2.0	34.5 ± 2.2	36.2 ± 2.5^{ns}
Total Leukocyte Count (10^3/µL)	12.5 ± 1.8	8.8 ± 1.2	7.2 ± 1.0*
Neutrophils (%)	10.0 ± 1.5	14.0 ± 2.0	21.0 ± 2.8***
Lymphocytes (%)	68.0 ± 3.5	75.0 ± 3.0	77.0 ± 2.5^{ns}
Monocytes (%)	2.0 ± 0.5	3.5 ± 0.6	4.0 ± 0.8^{ns}
Mean Corpuscular Volume (MCV) (fL)	48.0 ± 2.0	54.0 ± 2.2	56.5 ± 2.5^{ns}
Red Cell Distribution Width (RDW-SD) (%)	19.5 ± 1.2	16.8 ± 1.0	13.5 ± 0.9**
Platelet Count (10^3/µL)	320 ± 30	480 ± 35	650 ± 40***

Table 8S. Effect of treatment on liver function parameters in leukaemia-induced NOD SCID mice. Serum levels of total bilirubin, AST (SGOT), ALT (SGPT), and albumin were evaluated on day 28 post-induction to assess hepatic function following treatment with SAHA and **compound 14**. Values are expressed as mean ± SD. *, ***, and ns signify $p < 0.05$, $p < 0.001$, and not significant, respectively. Statistical significance symbols represent comparisons between **compound 14** and the SAHA-treated group.

Parameter (Units)	Disease Control	SAHA	Compound 14
Total Bilirubin (mg/dL)	0.80 ± 0.06	0.62 ± 0.05	0.45 ± 0.04***
SGOT / AST (U/L)	350 ± 40	240 ± 35	180 ± 18*
SGPT / ALT (U/L)	140 ± 12	95 ± 10	72 ± 7*
Albumin (g/dL)	2.8 ± 0.2	3.4 ± 0.3	3.8 ± 0.3^{ns}

Table 9S. Effect of treatment on kidney function parameters in leukaemia-induced NOD SCID mice. Serum levels of creatinine, urea, blood urea nitrogen (BUN), and uric acid were evaluated on day 28 post-induction to assess renal function following treatment with SAHA and **compound 14**. Values are expressed as mean ± SD. *, **, and *** signify $p < 0.05$, $p < 0.01$, and $p < 0.001$, respectively. Statistical significance symbols represent comparisons between **compound 14** and the SAHA-treated group.

Parameter (Units)	Disease Control	SAHA	Compound 14
Creatinine (mg/dL)	0.85 ± 0.06	0.62 ± 0.05	0.48 ± 0.04***
Blood Urea Nitrogen (BUN) (mg/dL)	32.5 ± 2.5	24.0 ± 2.0	18.5 ± 1.5**
Urea (mg/dL)	65.0 ± 4.0	48.0 ± 3.5	33.0 ± 2.8***
Uric Acid (mg/dL)	4.8 ± 0.5	4.2 ± 0.4	3.6 ± 0.3*

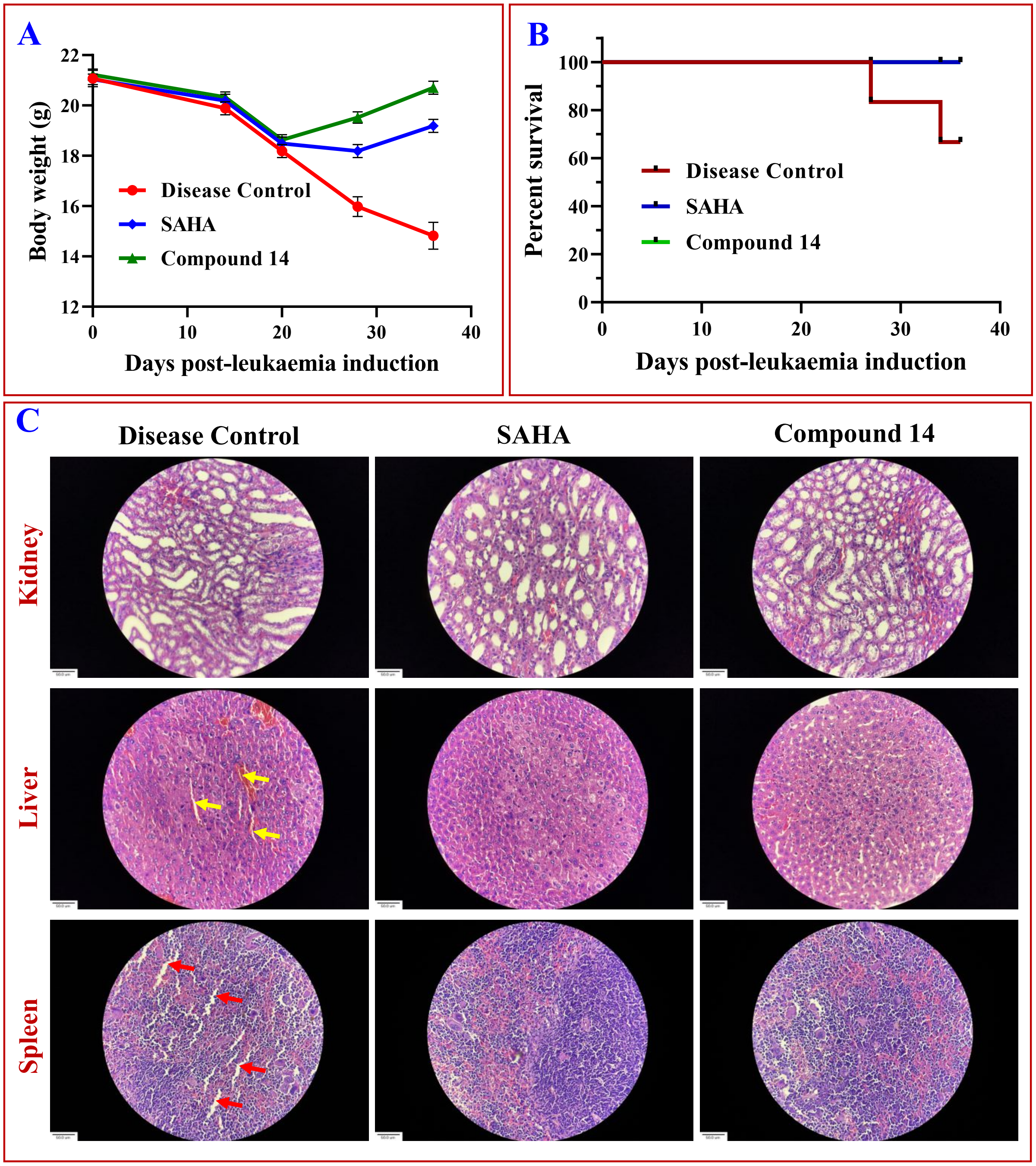

Fig 23S Effect of treatment on body weight (A) and survival **(B)** in leukaemia induced animals. (C) Histopathological microscopic images of kidney, liver, and spleen of animals from each group.

12.2 Experimental

12.2.1 Evaluation of Anti-tumor activity in a systemic leukemia model in NOD/SCID mice

Anti-leukemic activity was evaluated using a systemic leukemia model in NOD/SCID mice (aged 6-8 weeks, weighing 18-25 g). This animal study with approval number NISER/SBS/AH-378 was conducted following the guidelines of the Institutional Animal Care and Use Committee of the National Institute of Science Education and Research (NISER). Animals were housed under standard laboratory conditions with free access to food and water.

Human MV4-11 leukemia cells (2×10^6 cells per animal) were administered intravenously via tail vein injection to induce disseminated leukemia. Animals were randomly divided into three groups (n = 6 per group): 1) Disease control 2) SAHA-treated 3) Compound 14-treated. Leukemia induction was confirmed on days 14 and 20 post-inoculation by microscopic examination of peripheral blood smears. Following confirmation of disease establishment, treatment was initiated using SAHA (50 mg/kg, i.p) and **Compound 14** (50 mg/kg, i.p). Blood samples were collected on days 28 and 36 post-induction for evaluation of anti-leukaemic activity.

12.2.2 Cytological Evaluation and Leukemic Cell Quantification

On days 14 and 20 after inoculation, peripheral blood smears were prepared, stained with Giemsa, and examined by medical technologists. Following staining, leukemia cells generally appeared as enlarged, immature "blast" cells, characterized by a high nucleus-to-cytoplasmic ratio, loosely condensed chromatin, and prominent nucleoli. A minimum of 200 cells per field was manually counted to distinguish red blood cells (RBCs), white blood cells (WBCs), and leukemic cells (MV4-11) based on their morphological features, and the percentage of MV4-11 cells was determined.

For viability assessment, blood samples were incubated with ammonium chloride buffer to remove RBCs and then stained with Trypan Blue. Total viable cells were quantified using an automated cell counter (Bio-Rad TC-20). The absolute leukemic cell burden was calculated as follows:

Total leukemic cells = Viable cells × % MV4-11 cells

12.2.3 Hematological and Biochemical Analysis

Peripheral blood samples were collected on day 28 post-inoculation for complete blood count (CBC) analysis using an automated hematology analyzer. Serum was obtained by centrifugation and used for biochemical analysis of liver and kidney function with standard diagnostic kits, following the manufacturer's instructions.

12.2.4 Body Weight and Survival Analysis

The body weight of all experimental animals was recorded at predetermined time points (days 0, 14, 20, 28, and 36) using a calibrated digital weighing balance. Body weight changes were monitored throughout the study to assess disease progression and treatment-related effects.

Animals were observed daily for survival throughout the experimental period (up to day 36). The time of death for each animal was recorded, while animals surviving until the study endpoint were considered censored. Survival data were analyzed using the Kaplan–Meier method, and survival curves were generated for each group.

12.2.5 Histopathology

Tissues including kidney, spleen, and liver were collected from experimental animals at the study endpoint (day 36) and immediately fixed in 10% neutral buffered formalin. Fixed tissues were processed, embedded in paraffin,

sectioned at ~4–5 µm thickness, and mounted on glass slides. Sections were stained with haematoxylin and eosin (H&E) and examined under a light microscope for histopathological changes.

12.2.6 Statistical Analysis

Data are represented as mean ± SD. Statistical analysis of the obtained data was performed using one-way ANOVA followed by Tukey's post hoc test. Survival curves were analyzed using the log-rank test.

^{1}H NMR of compound ED-1

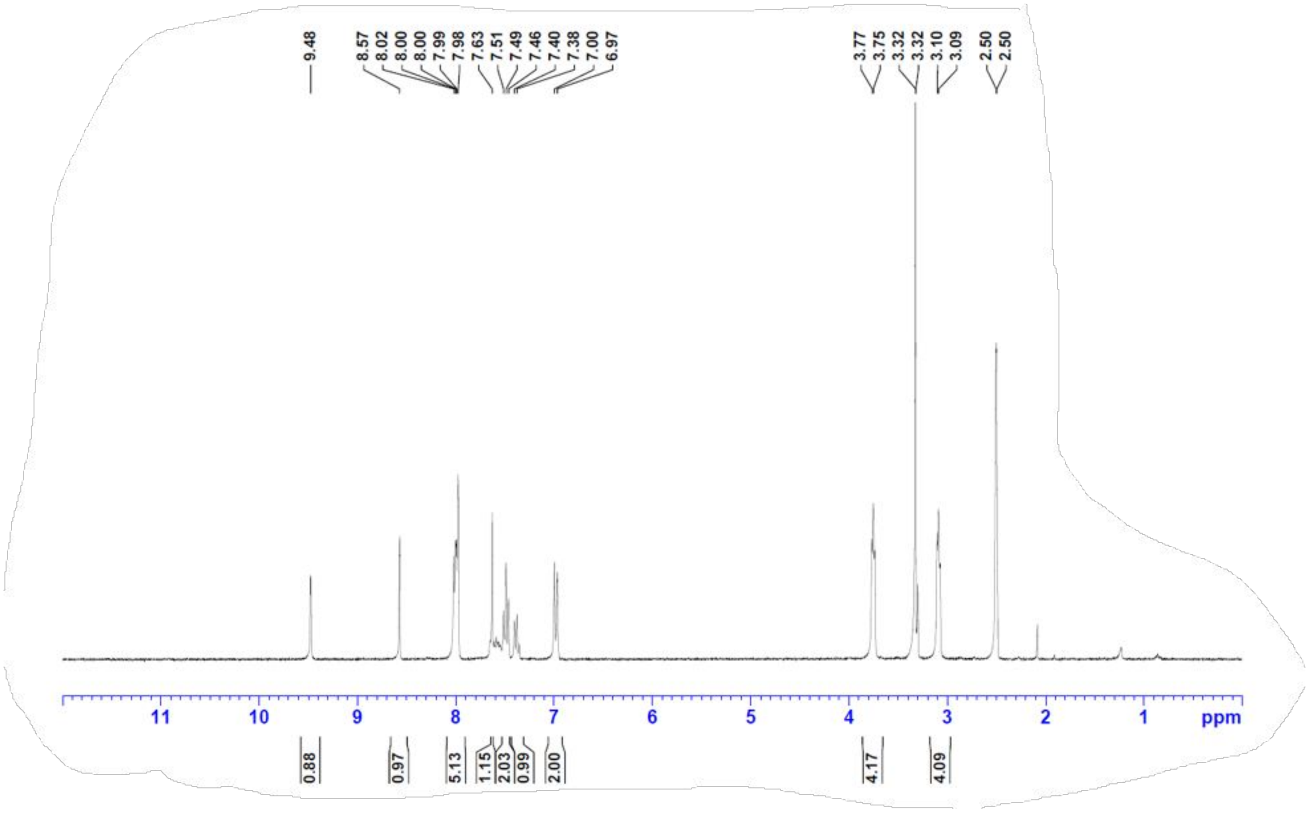

^{1}H NMR of compound ED-2

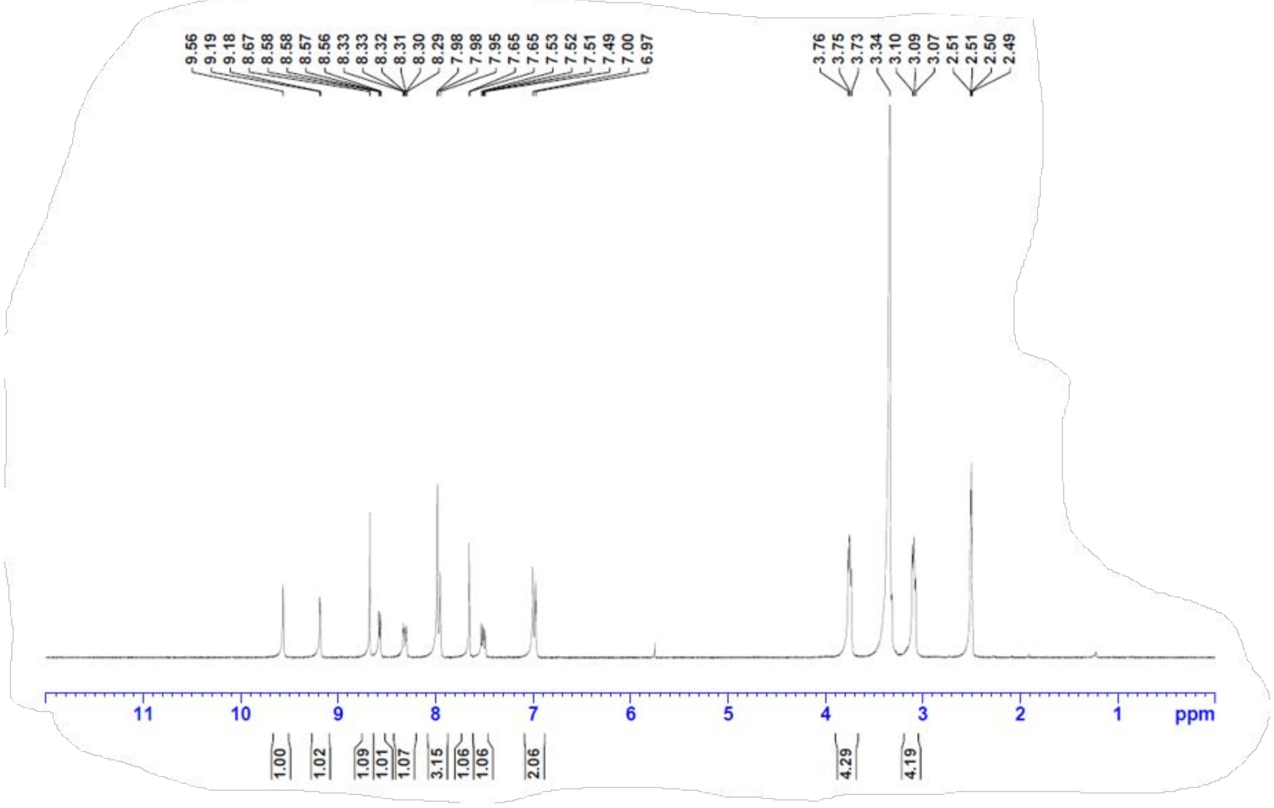

^{1}H NMR of compound ED-3

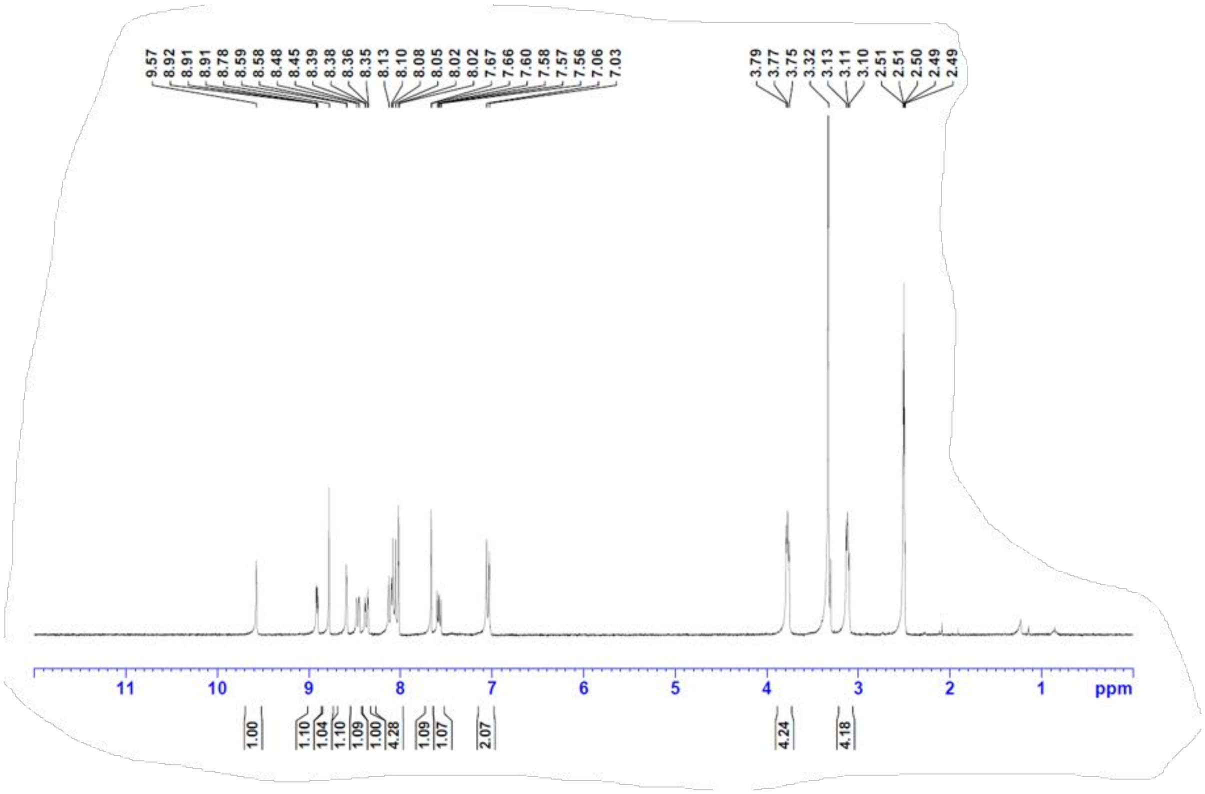

^{1}H NMR of compound ED-4

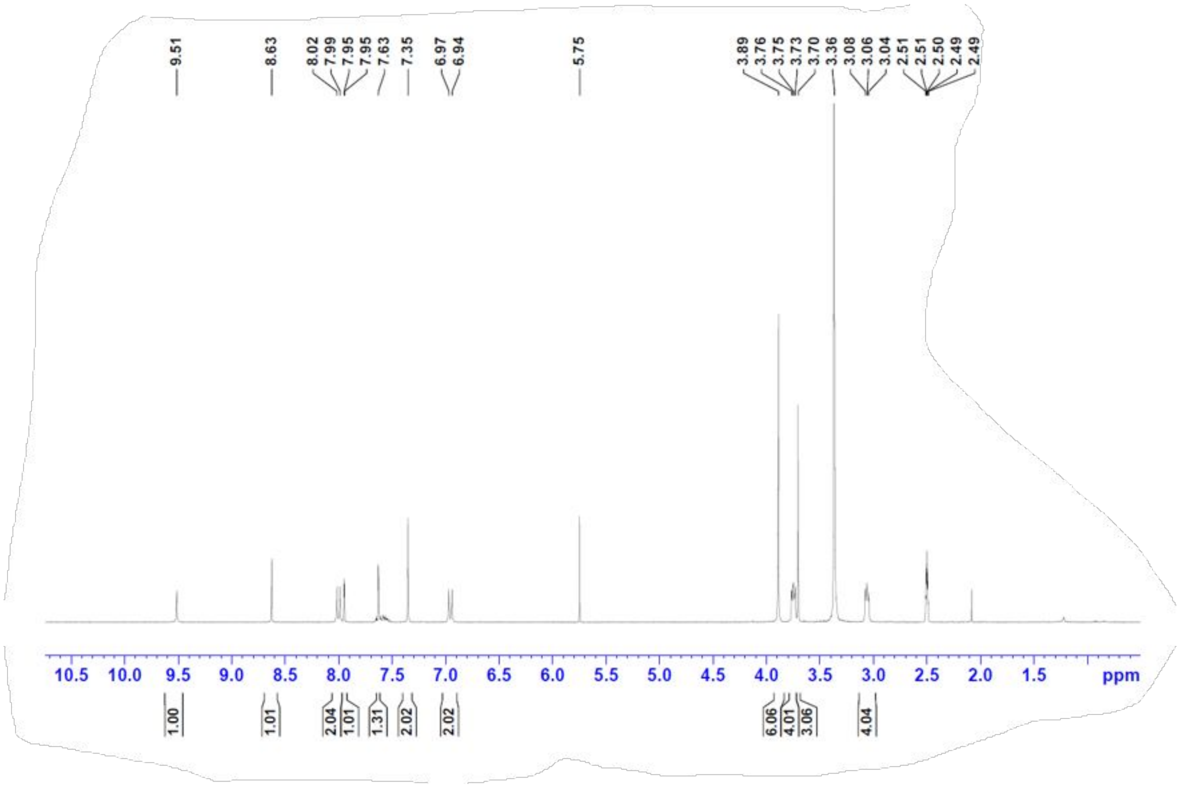

^{1}H NMR of compound ED-5

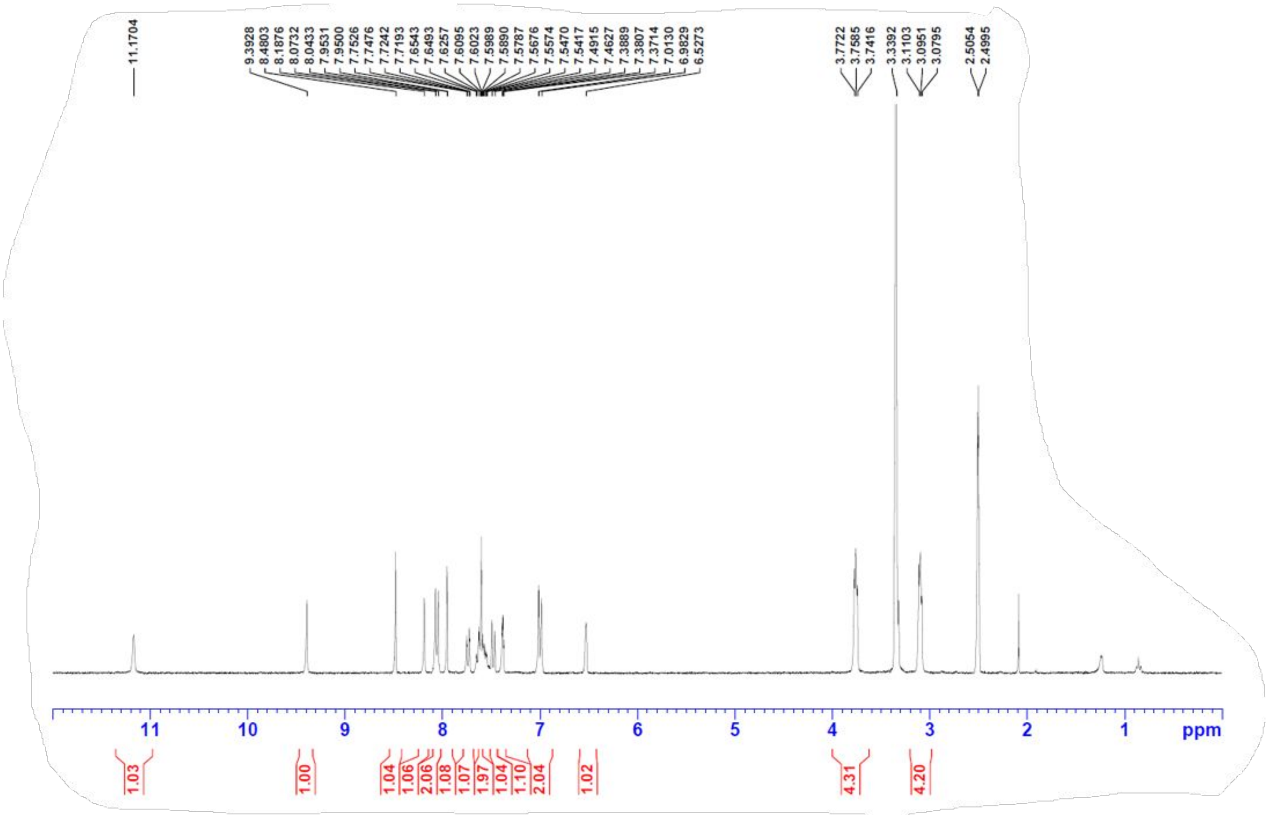

^{1}H NMR of compound ED-6

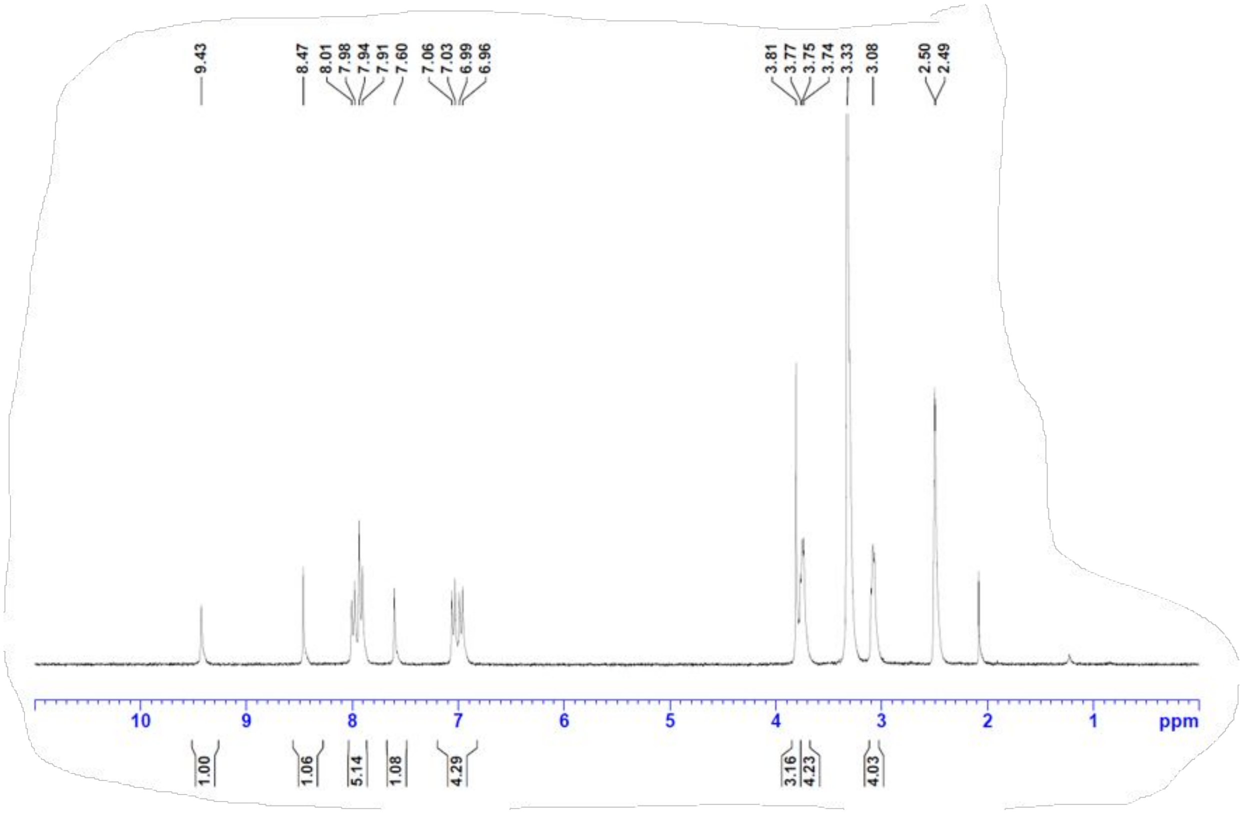

^{1}H NMR of compound ED-7

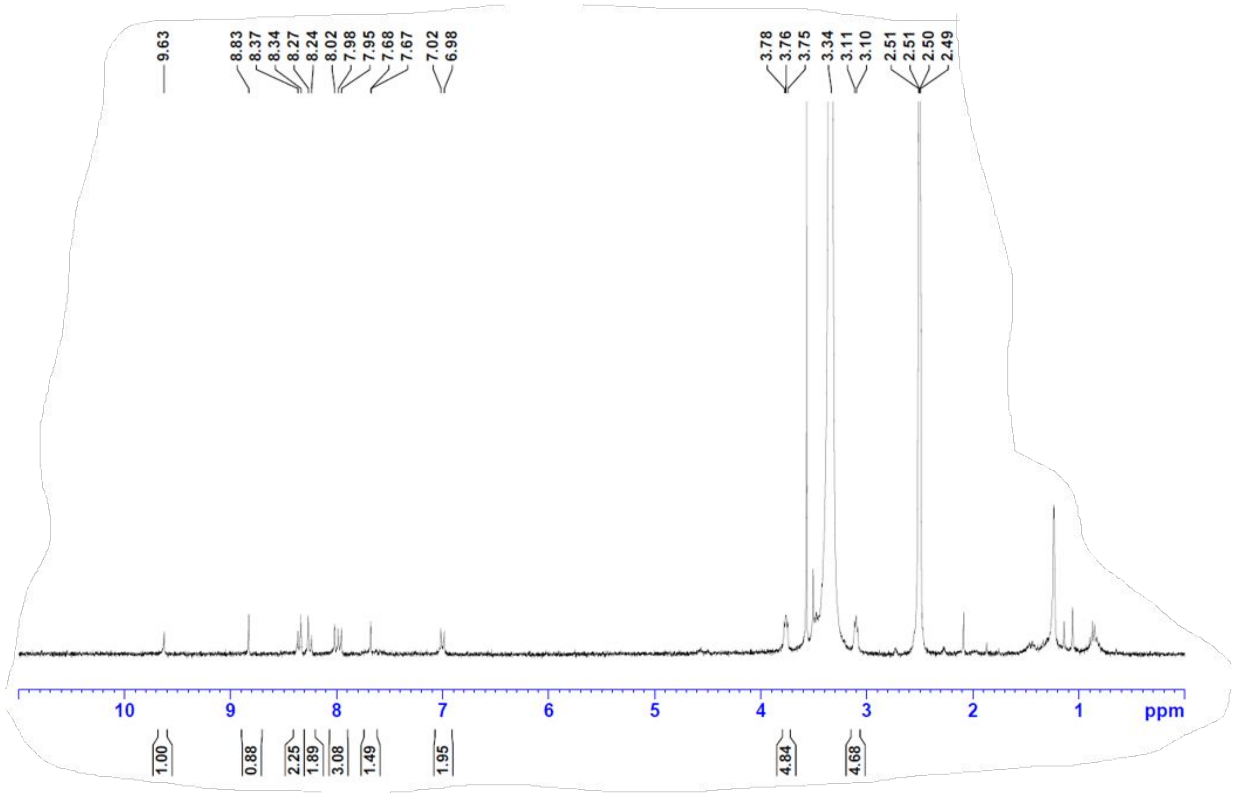

^{1}H NMR of compound ED-8

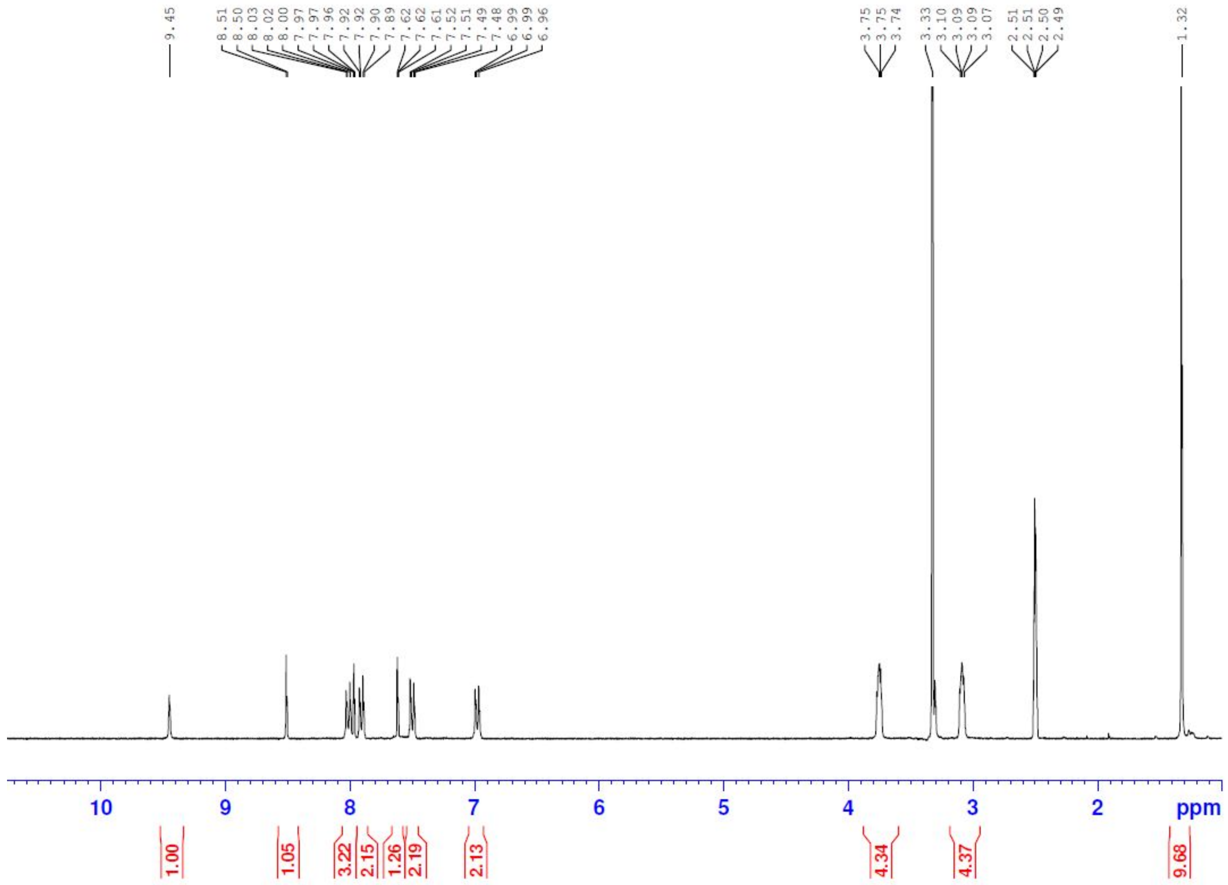

^{1}H NMR of compound ED-9

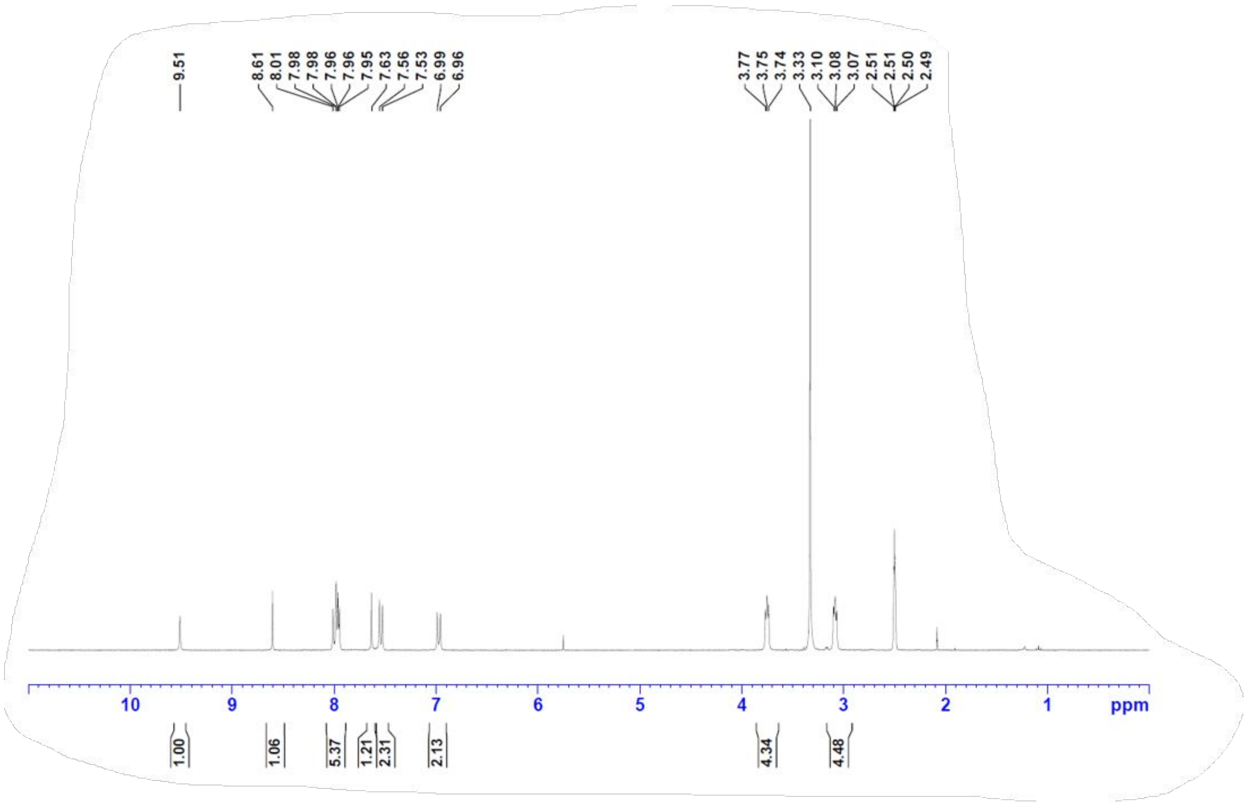

^{1}H NMR of compound ED-10

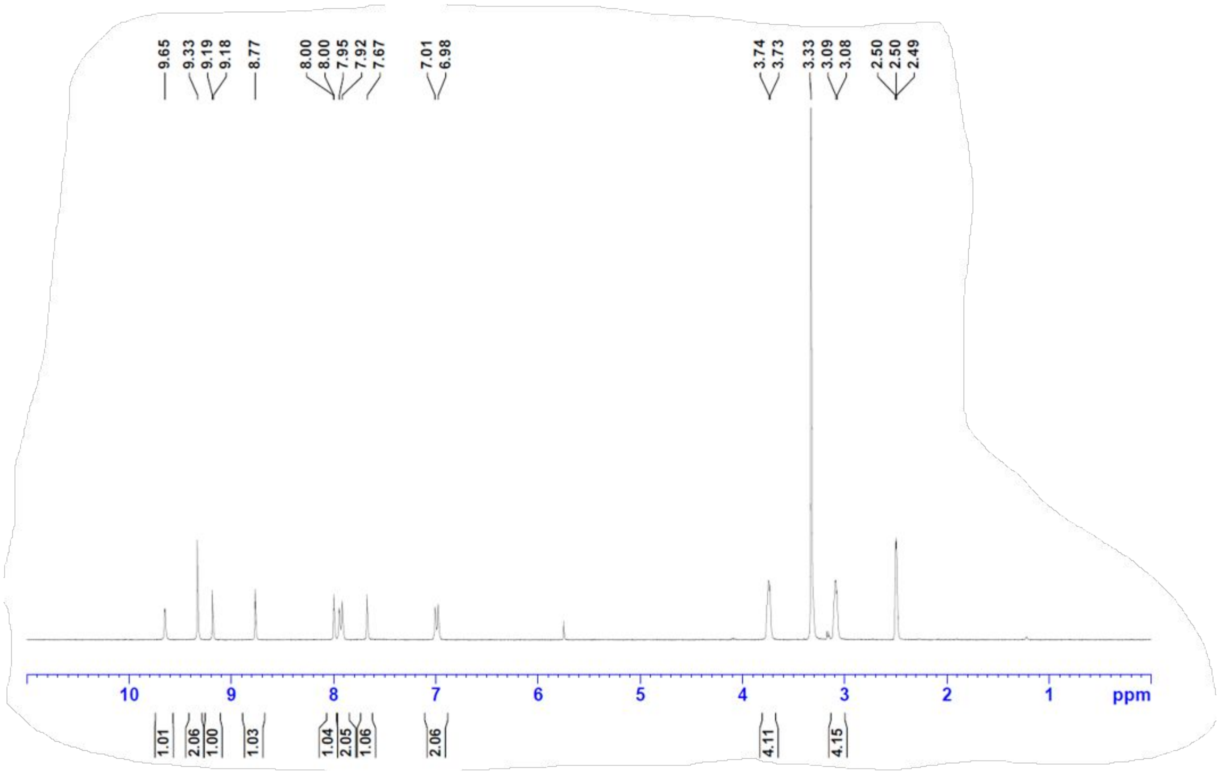

^{1}H NMR of compound ED-11

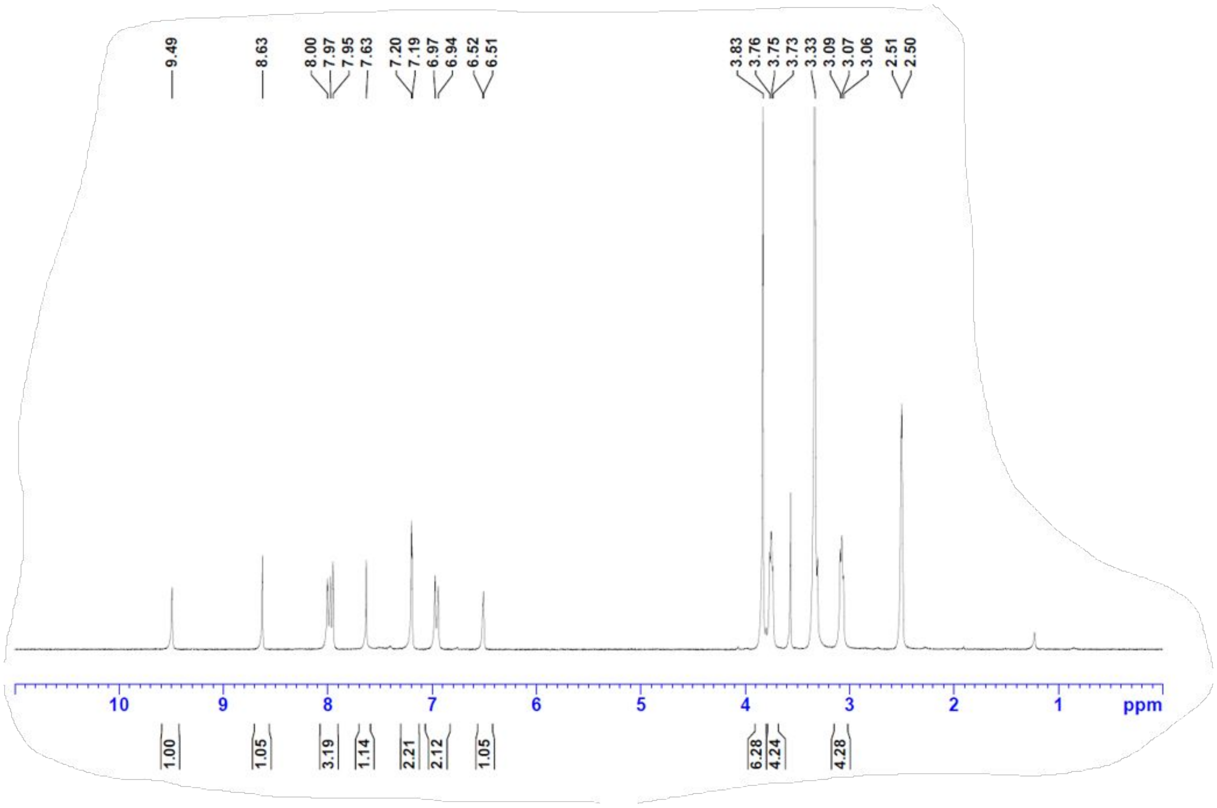

^{1}H NMR of compound ED-12

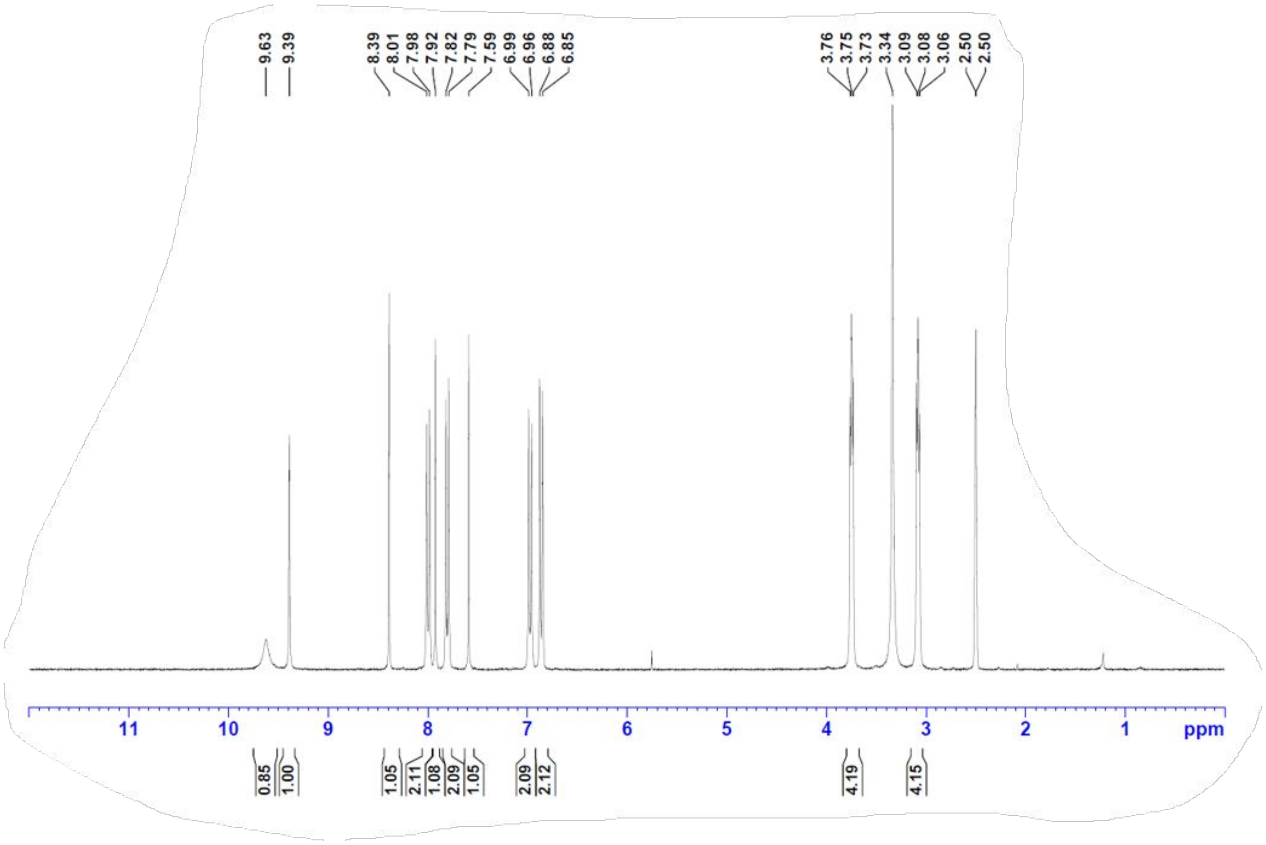

^{1}H NMR of compound ED-13

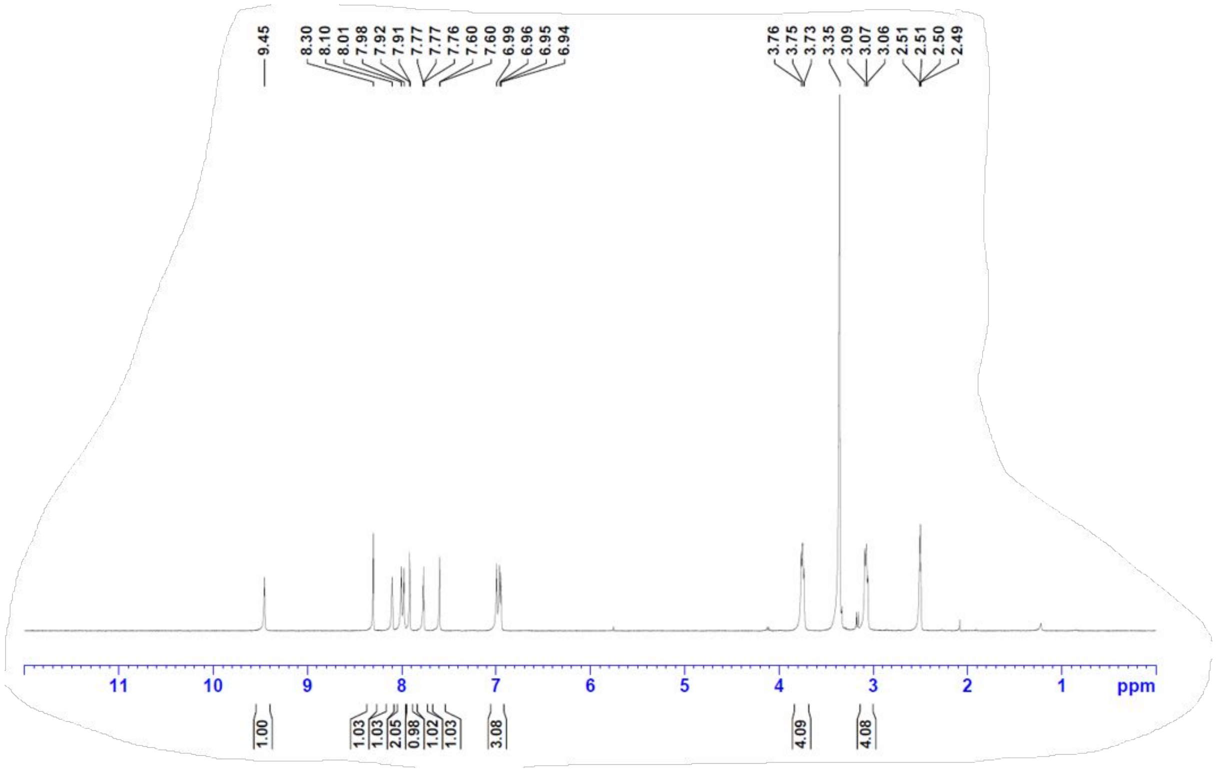

^{1}H NMR of compound ED-14

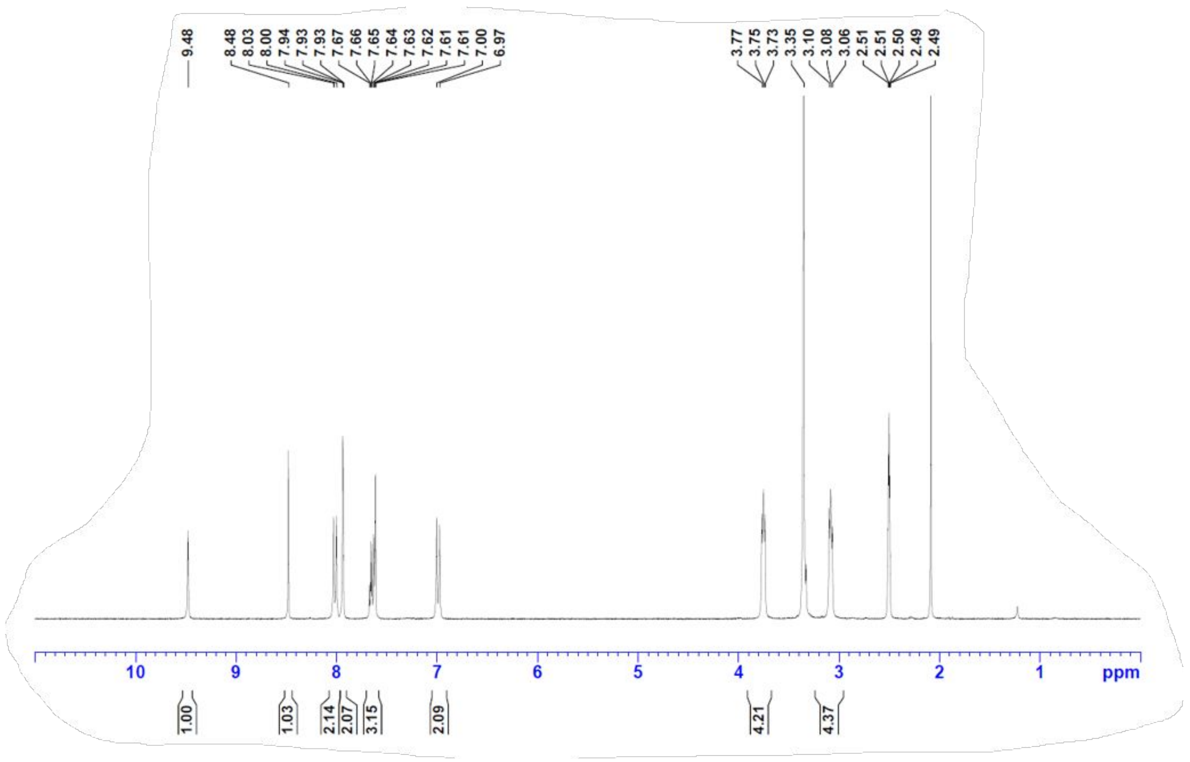

^{1}H NMR of compound ED-15

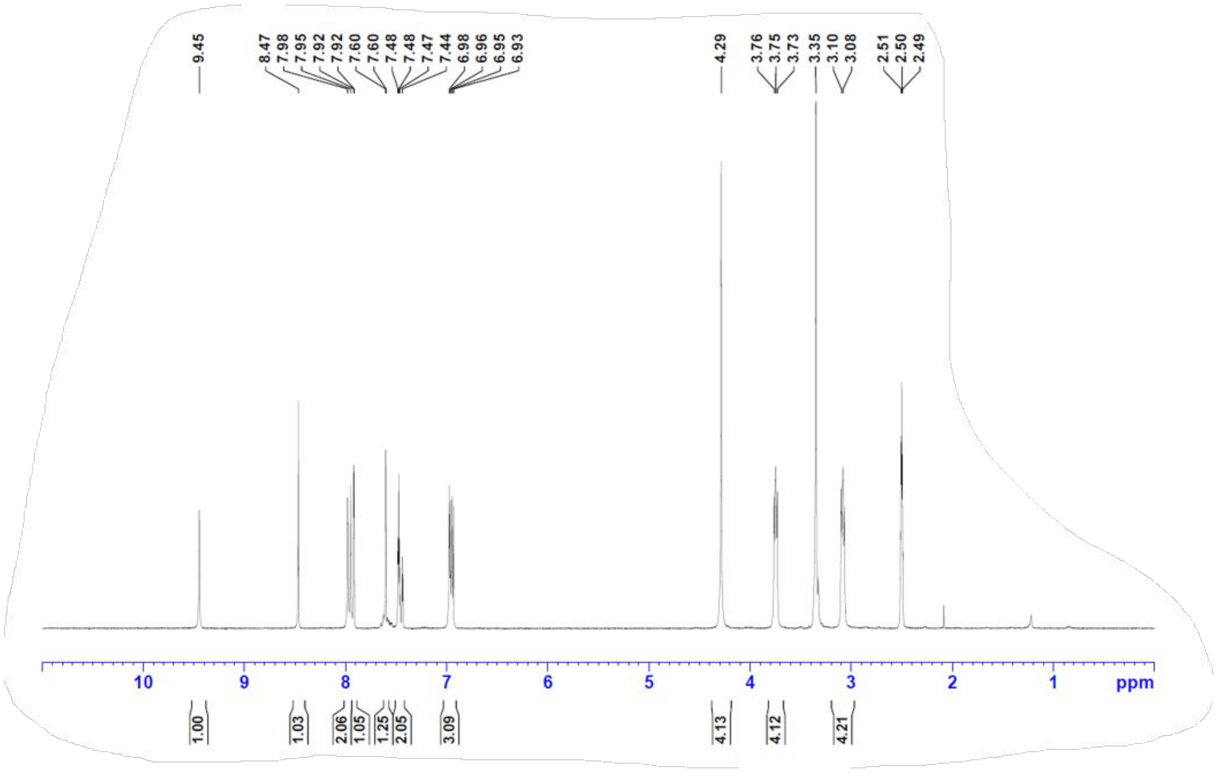

^{1}H NMR of compound ED-16

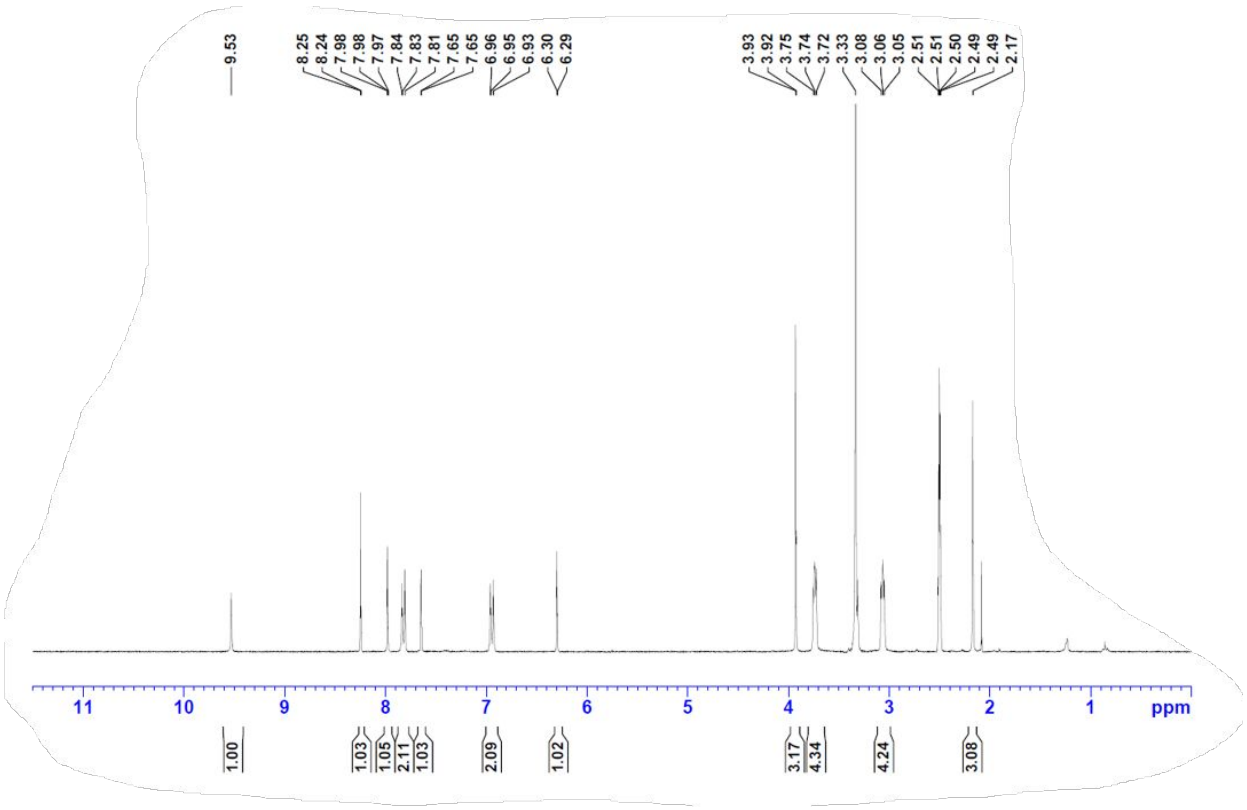

^{1}H NMR of compound ED-17

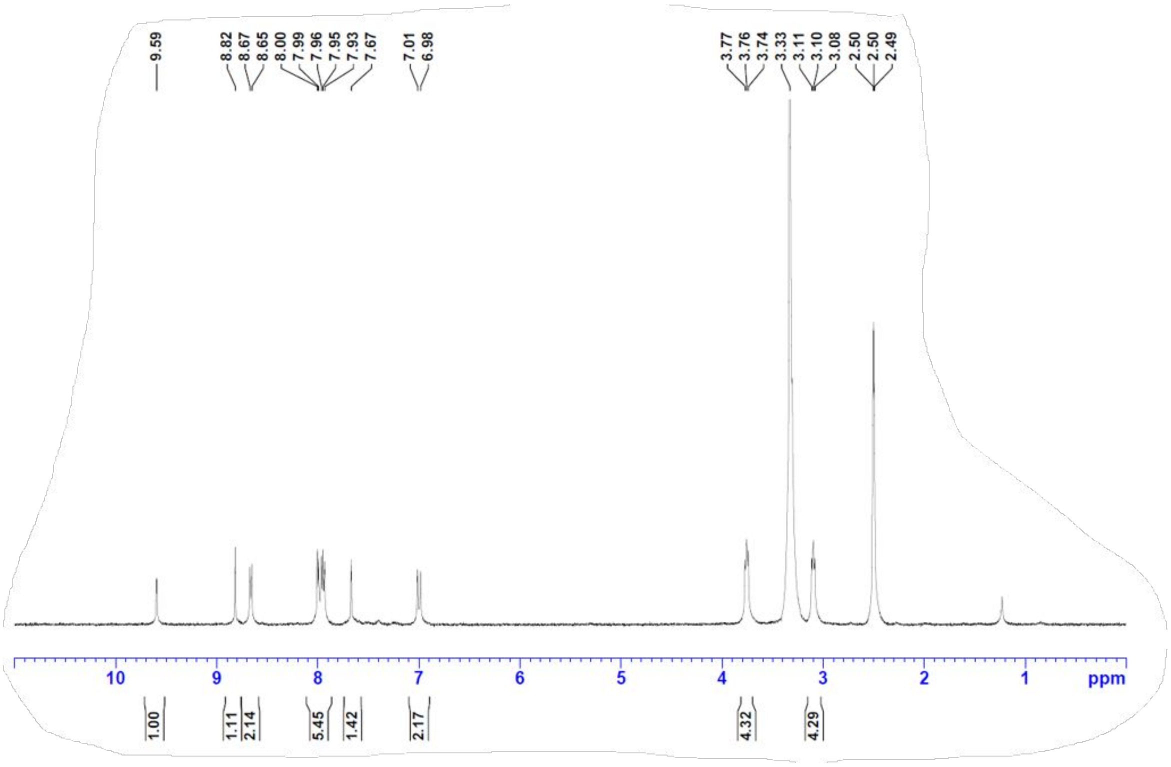

^{1}H NMR of compound ED-18

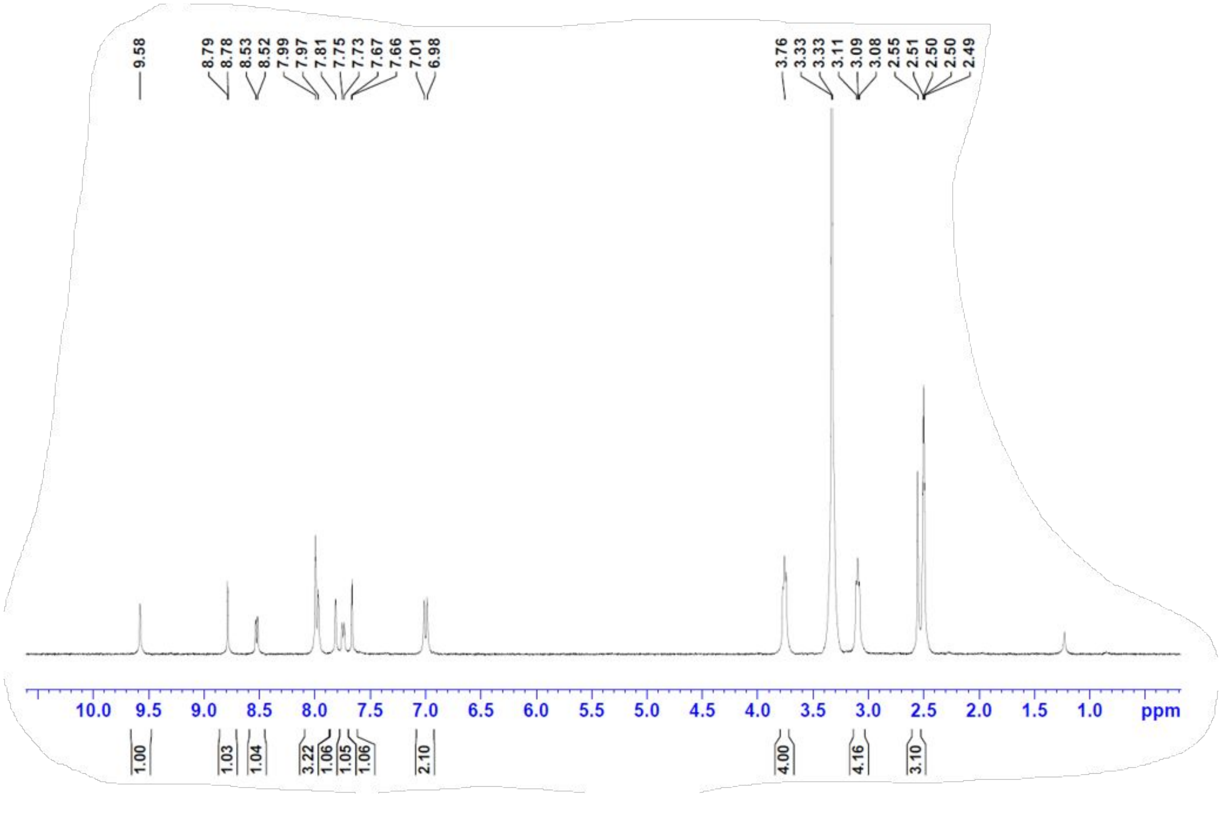

^{1}H NMR of compound ED-19

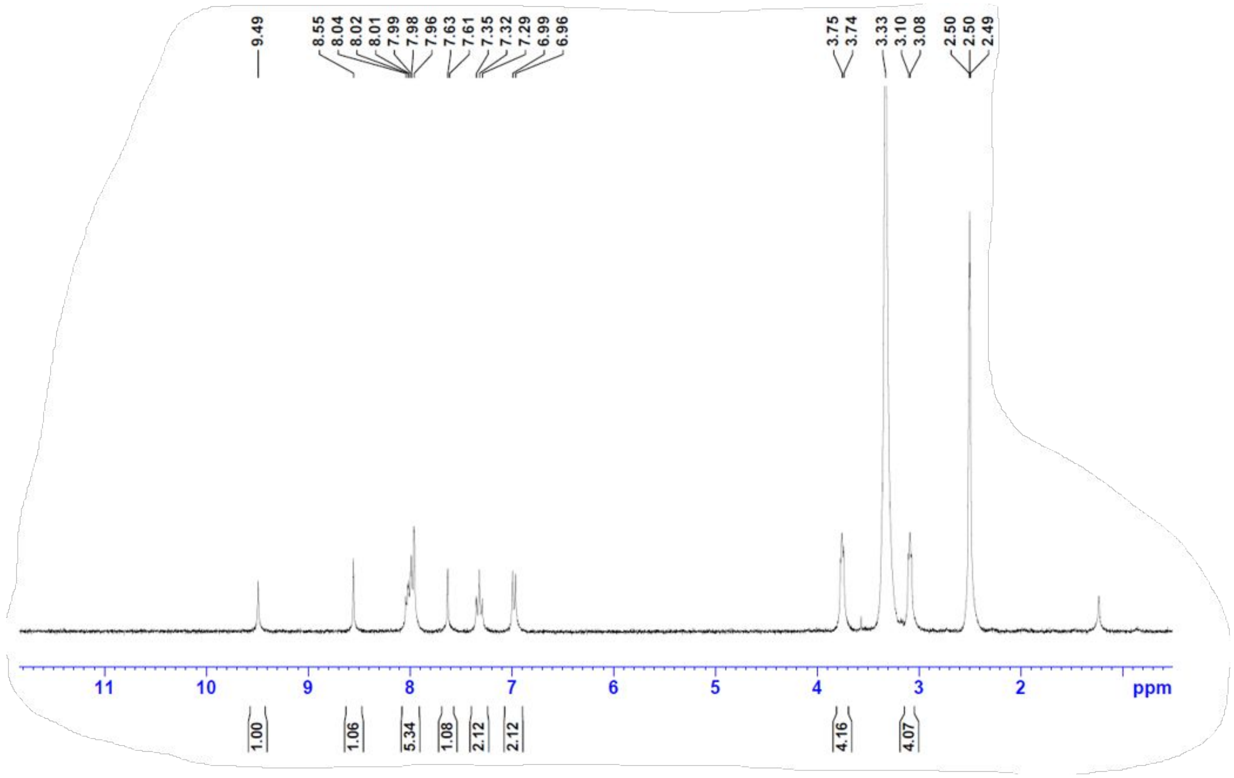

^{1}H NMR of compound ED-20

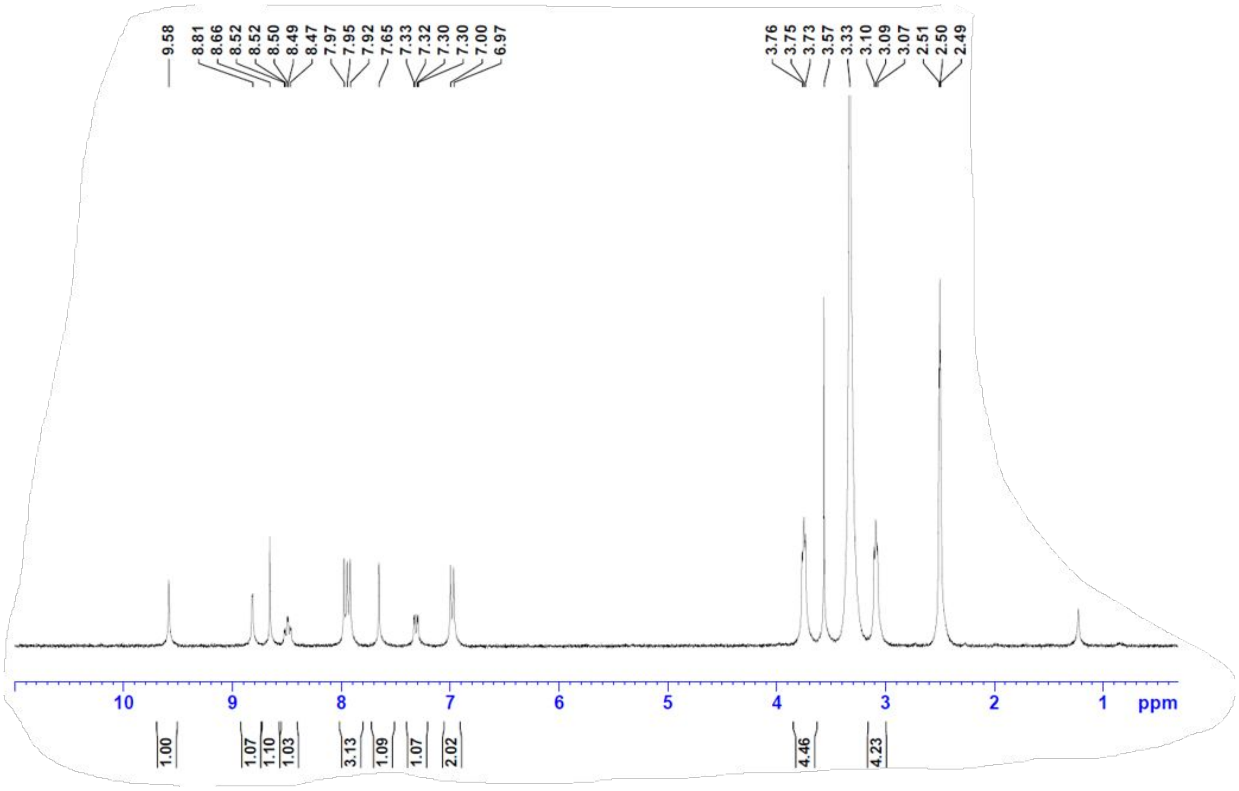

^{1}H NMR of compound ED-21

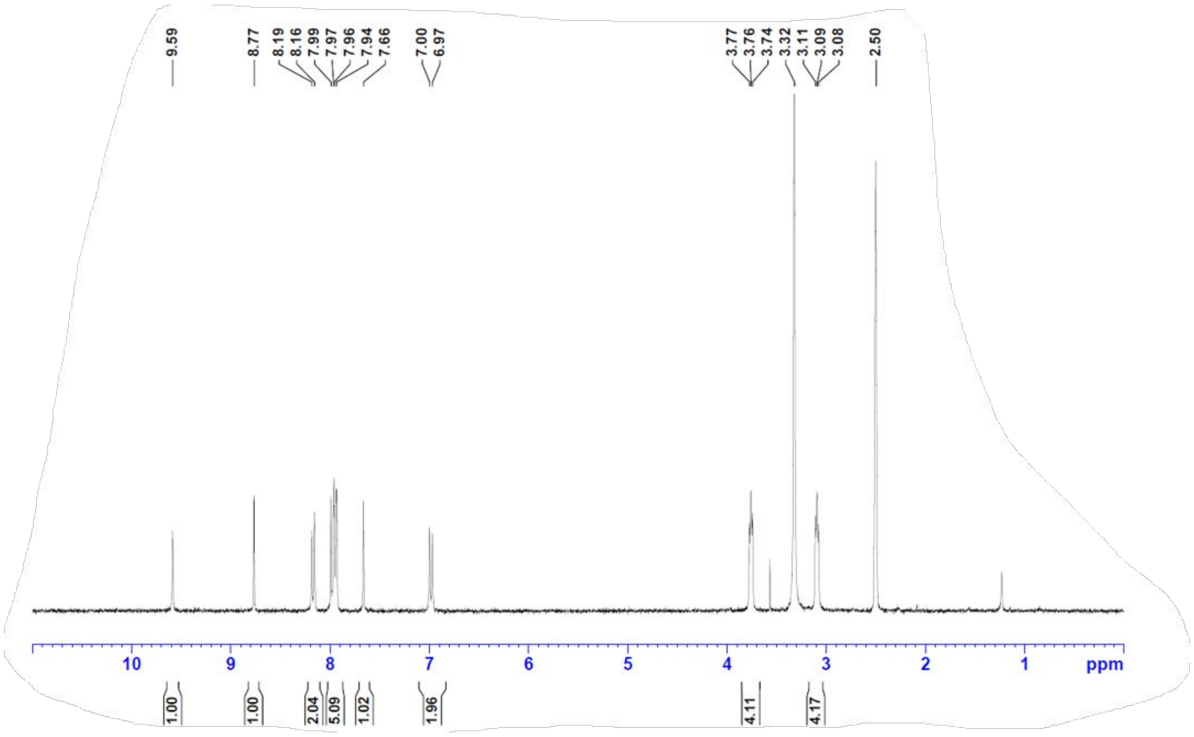

^{1}H NMR of compound ED-22

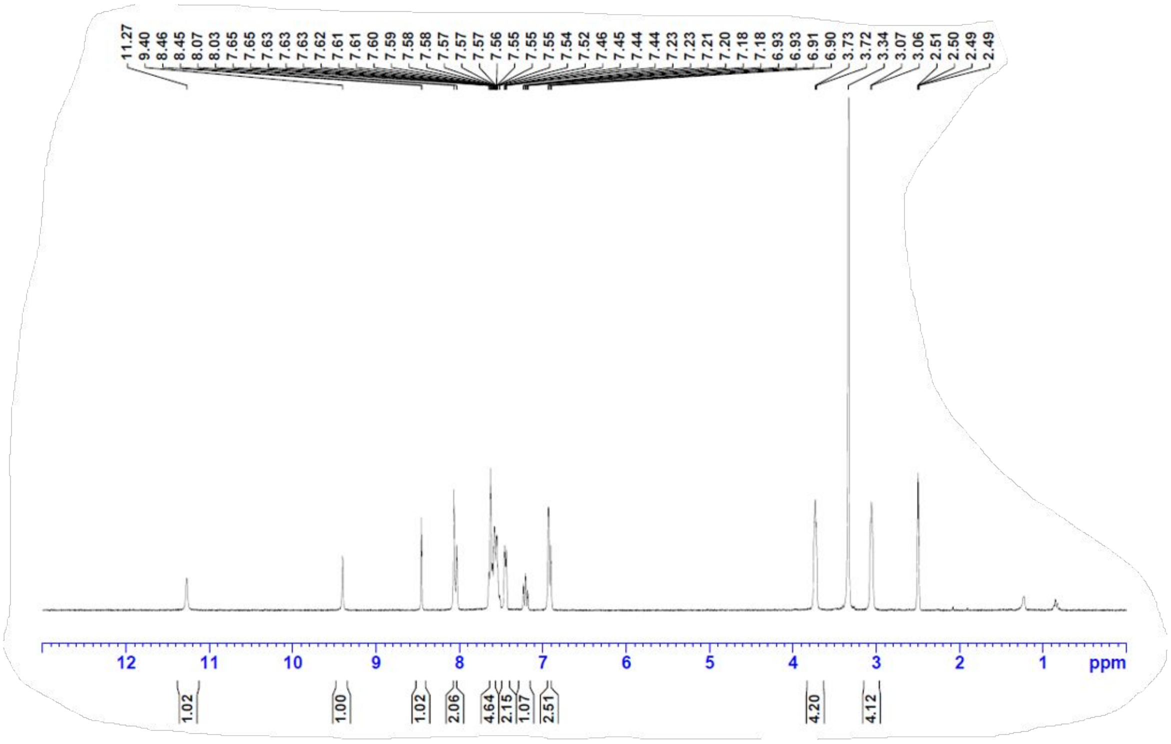

^{1}H NMR of compound ED-23

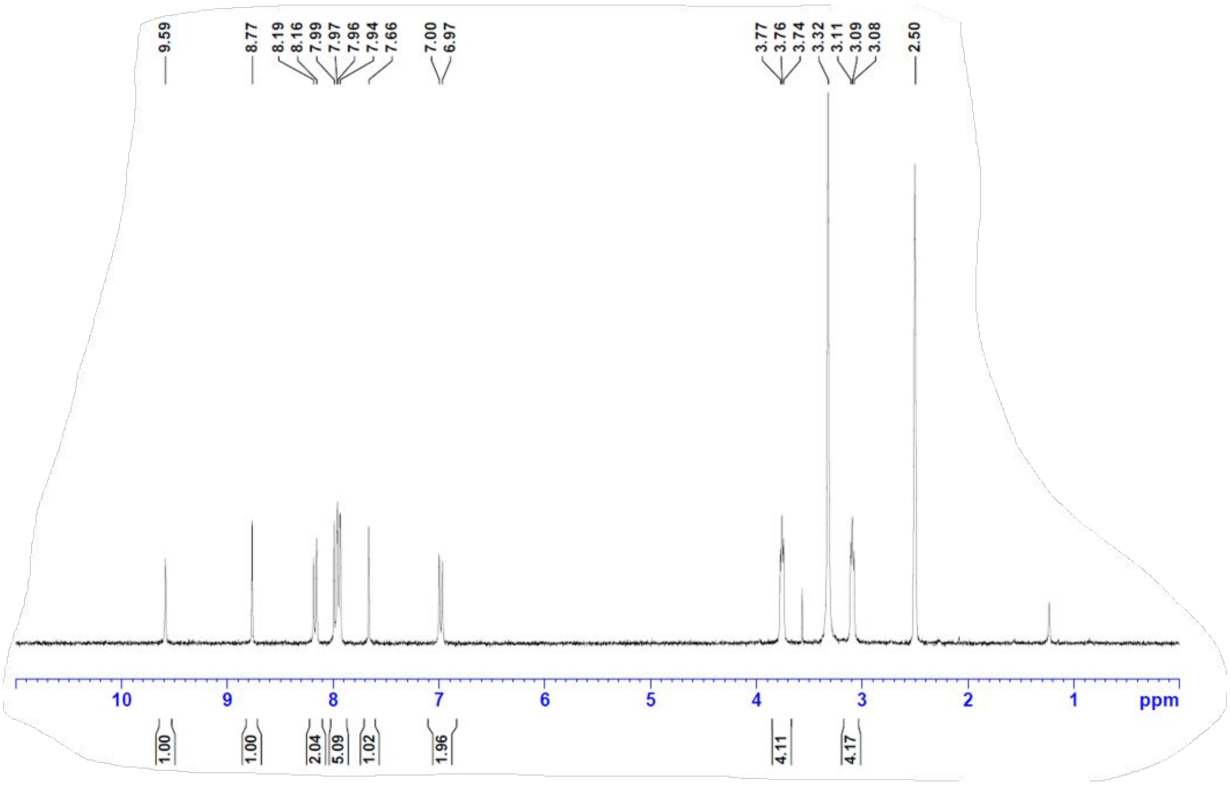

^{1}H NMR of compound ED-24

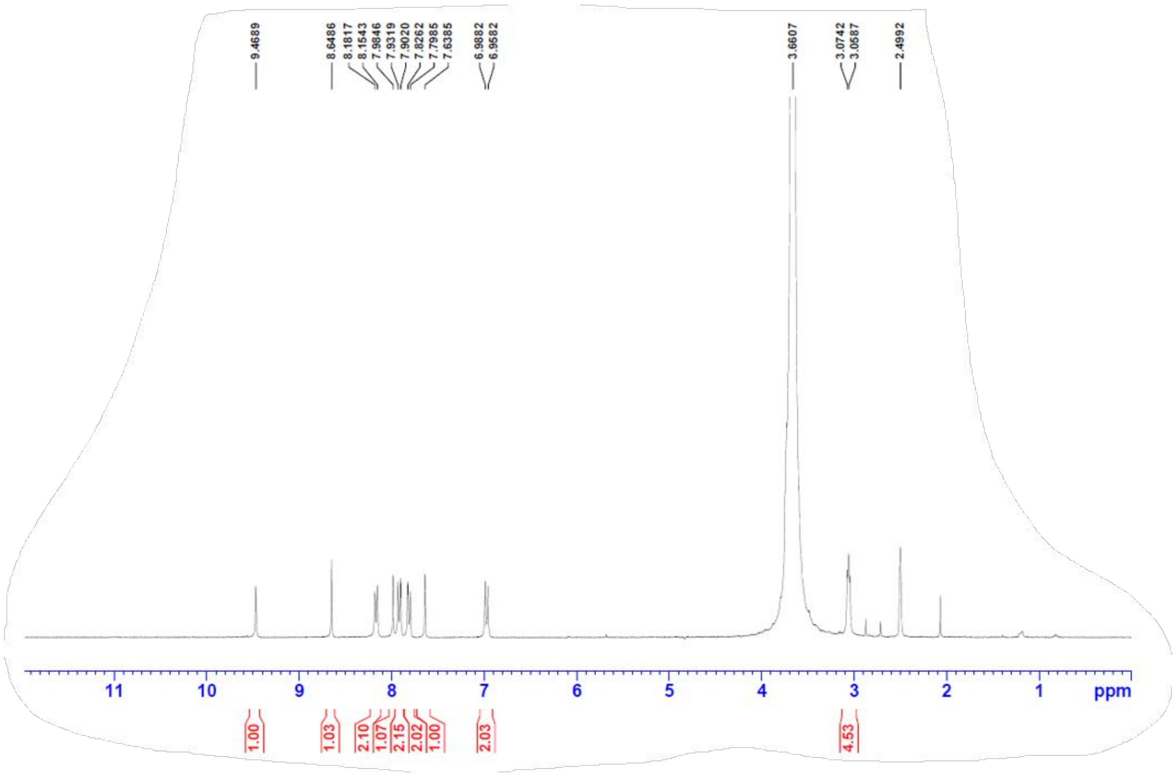

^{1}H NMR of compound ED-25

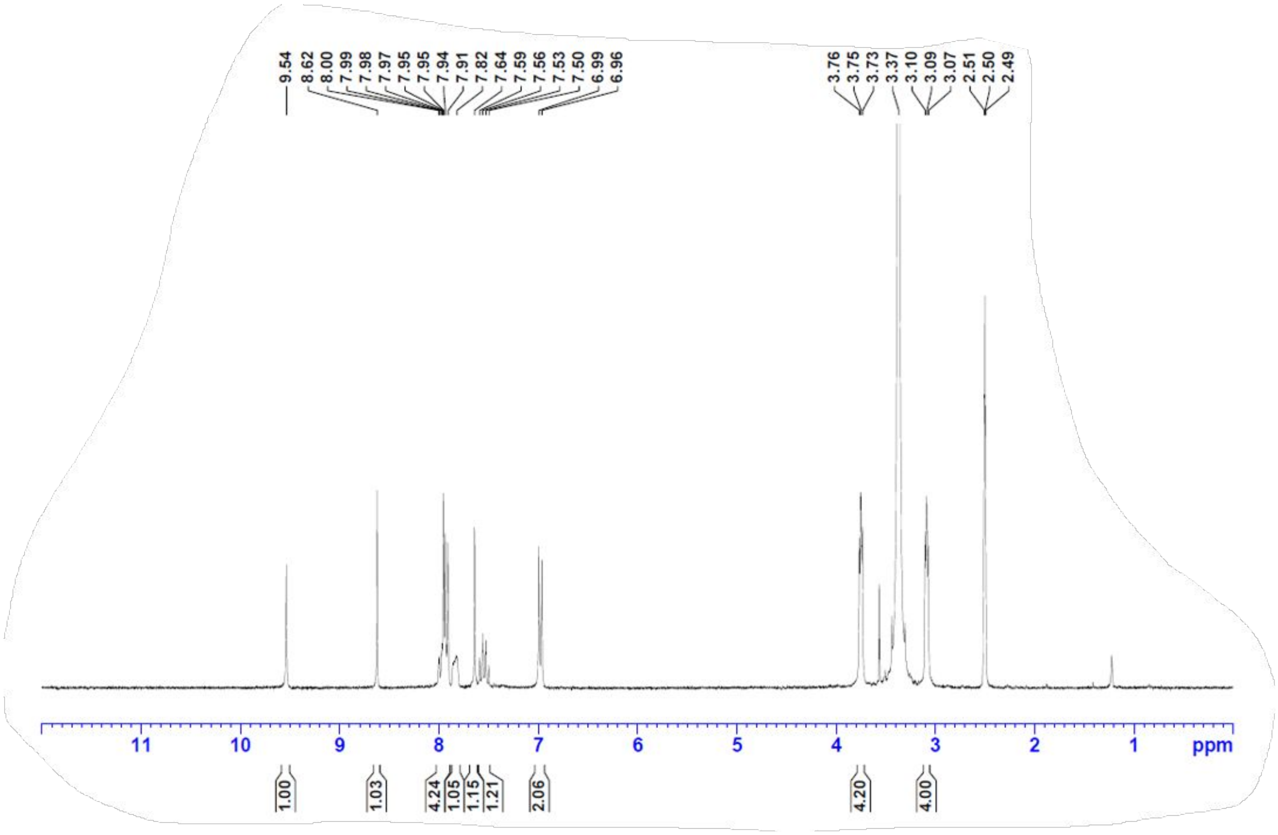

^{1}H NMR of compound ED-26

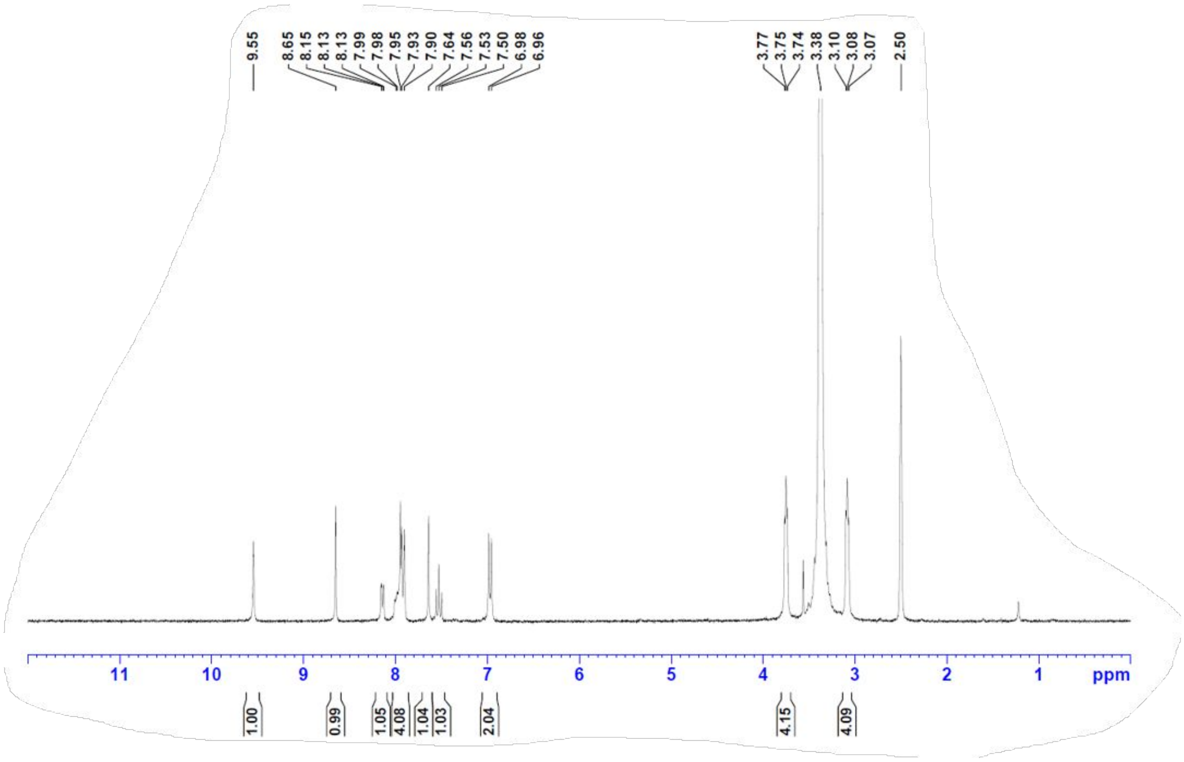

^{1}H NMR of compound ED-27

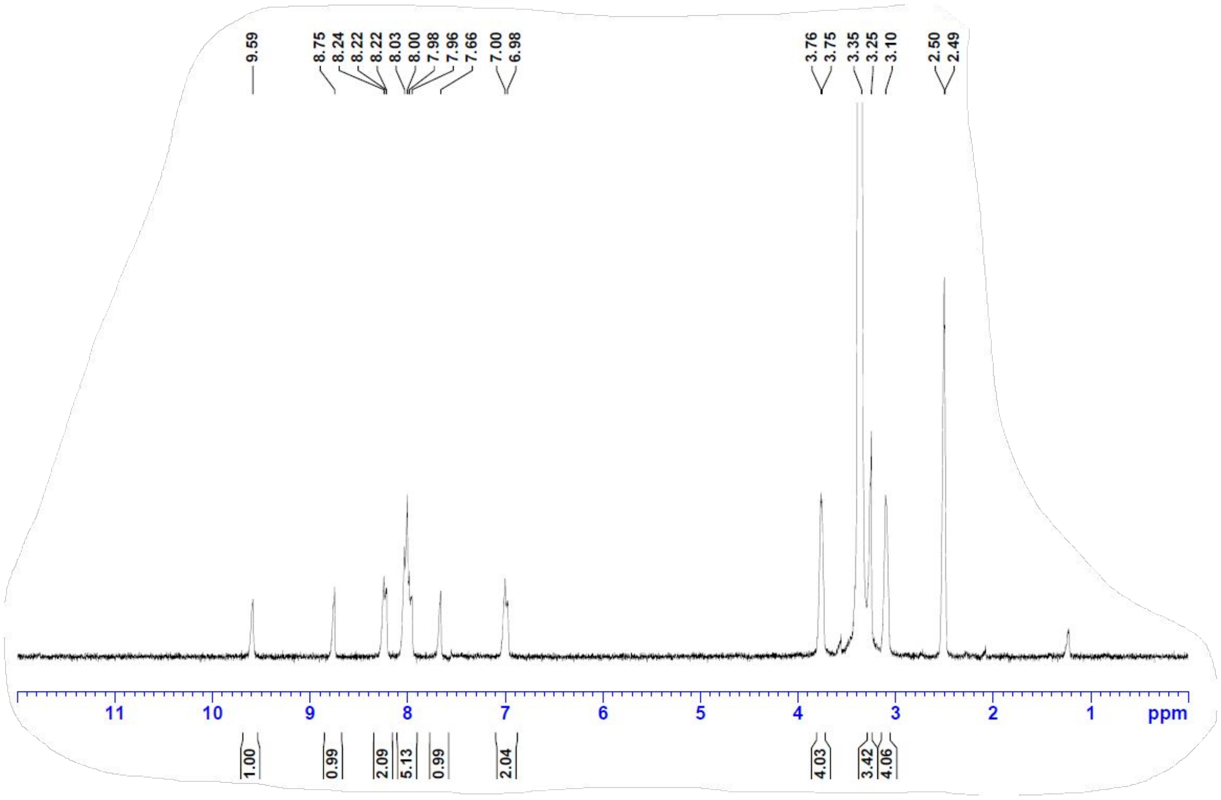

^{1}H NMR of compound ED-28

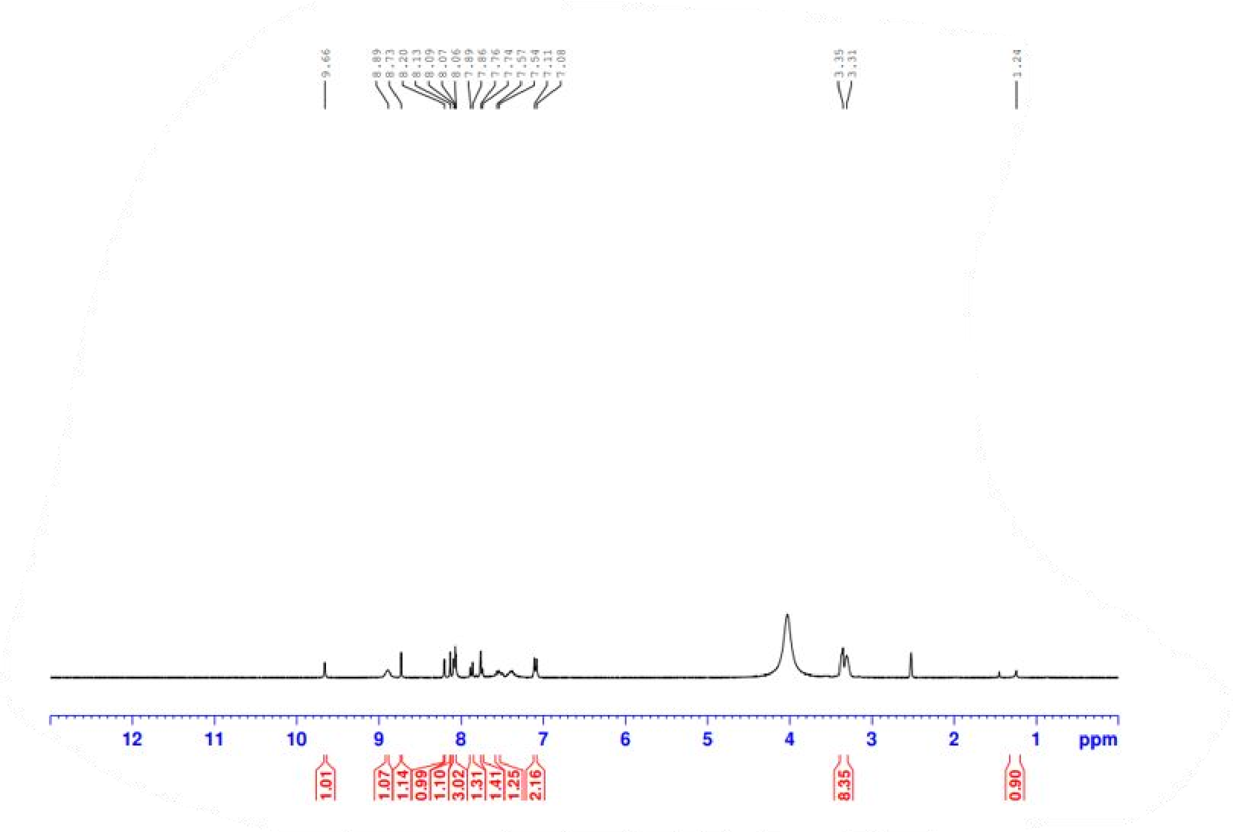

^{1}H NMR of compound ED-29

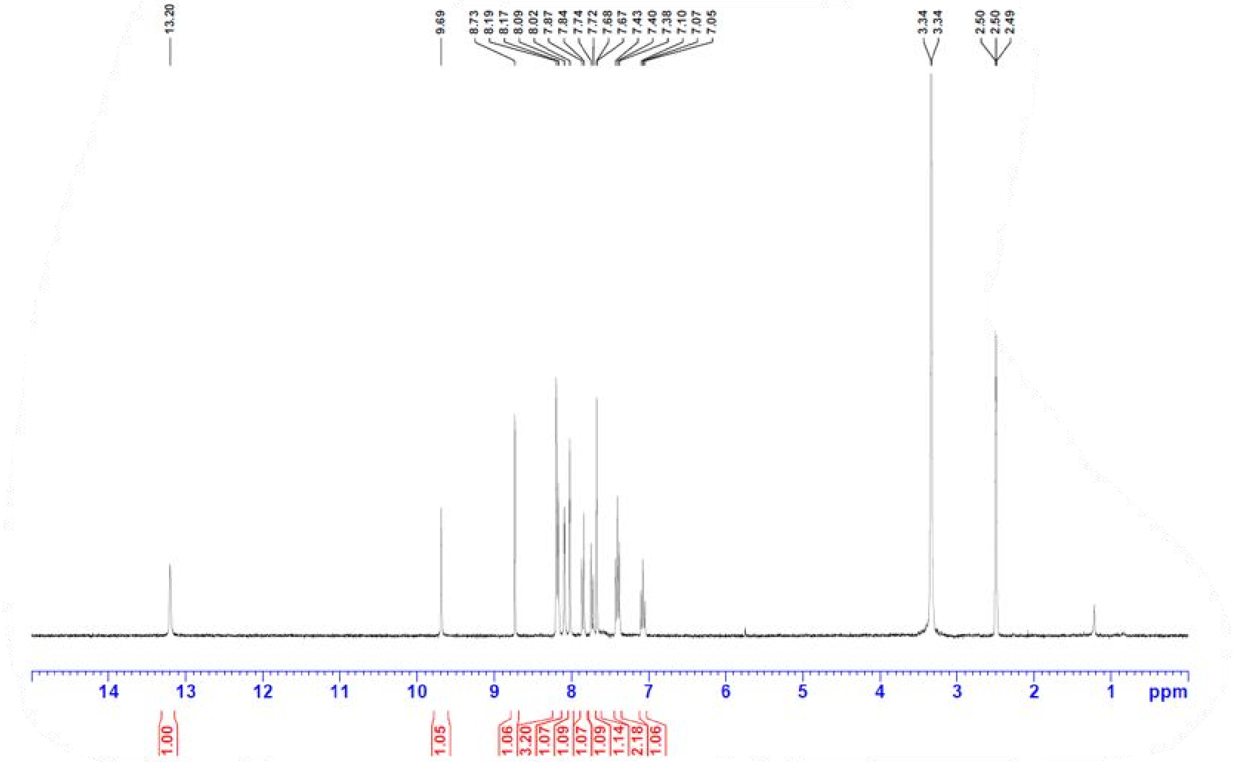

^{1}H NMR of compound ED-30

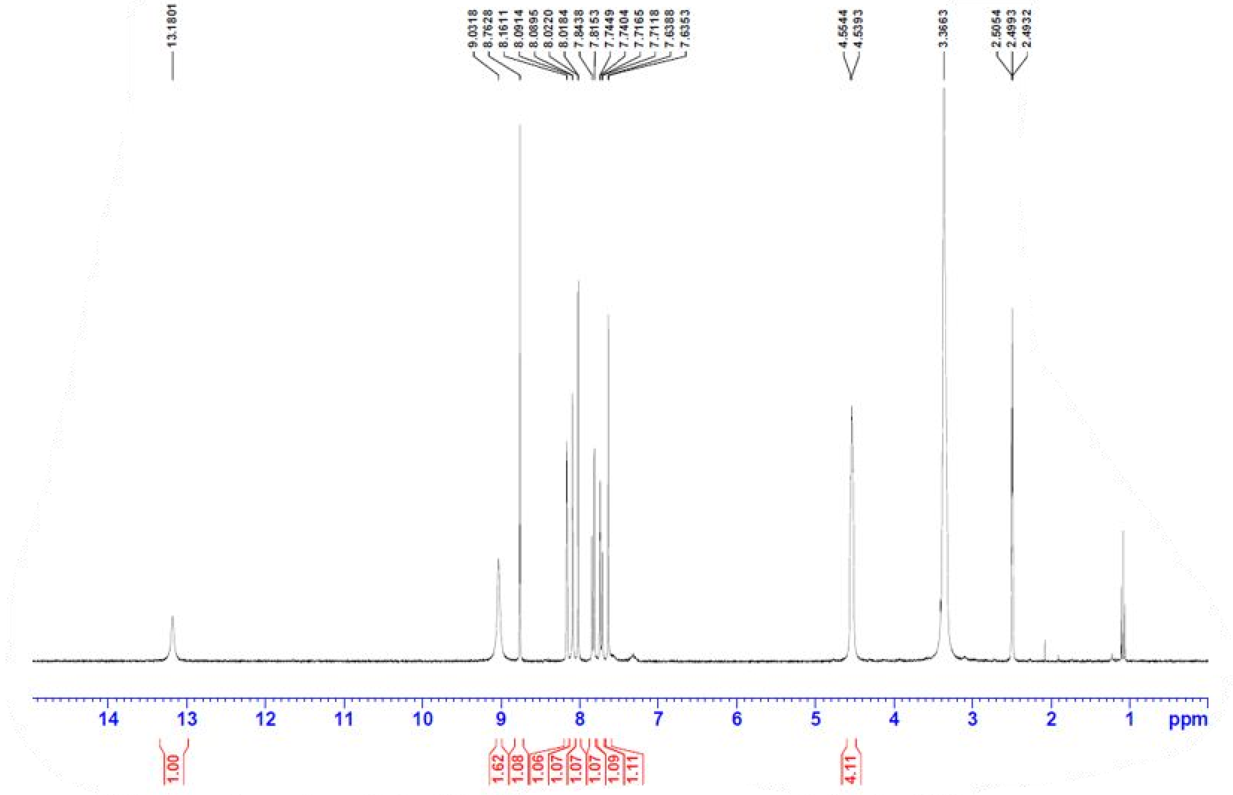

^1H NMR of compound 1

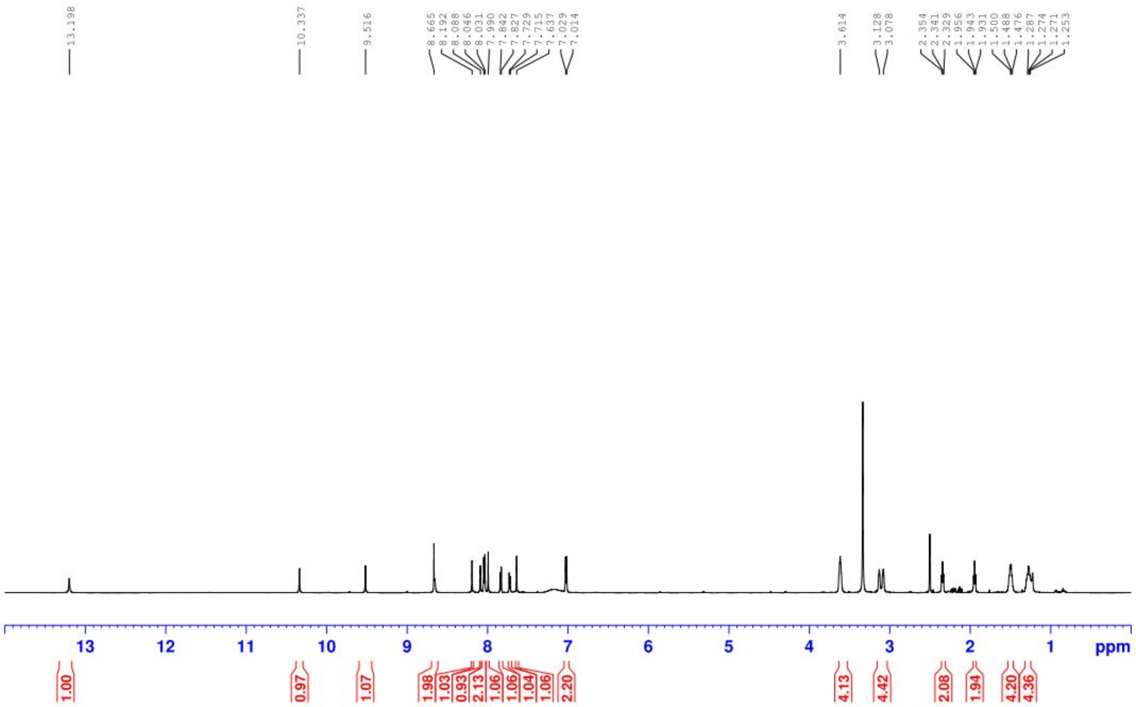

^1H NMR of compound 2

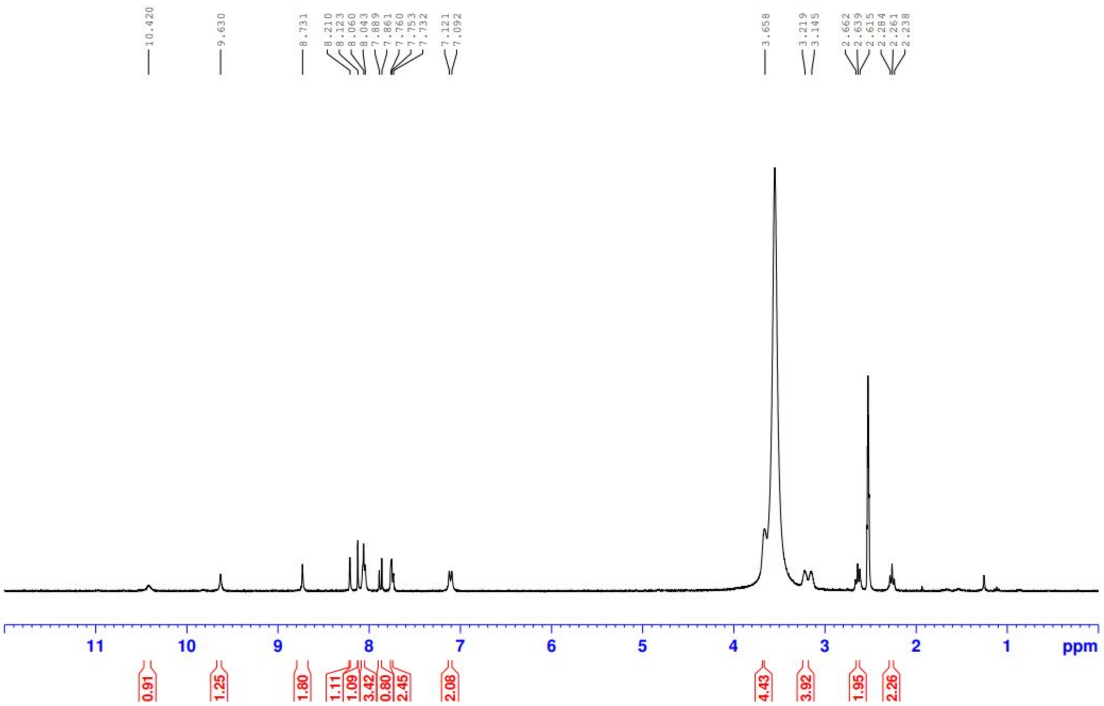

^{1}H NMR of compound 3

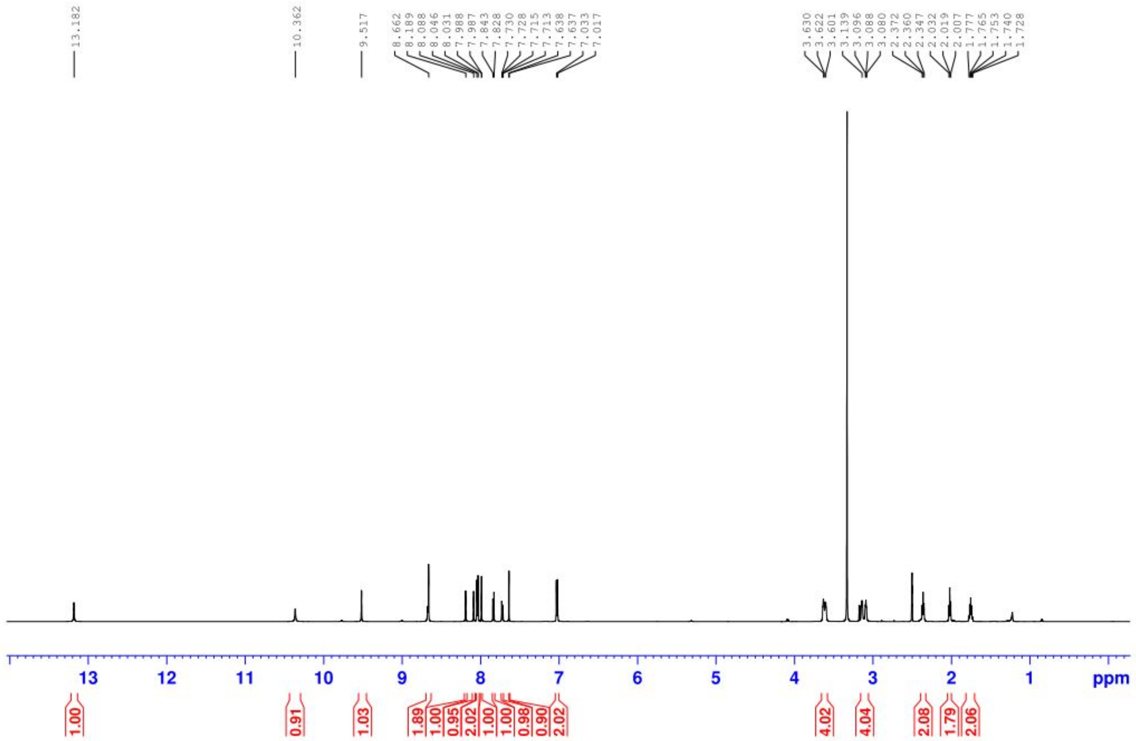

^{1}H NMR of compound 4

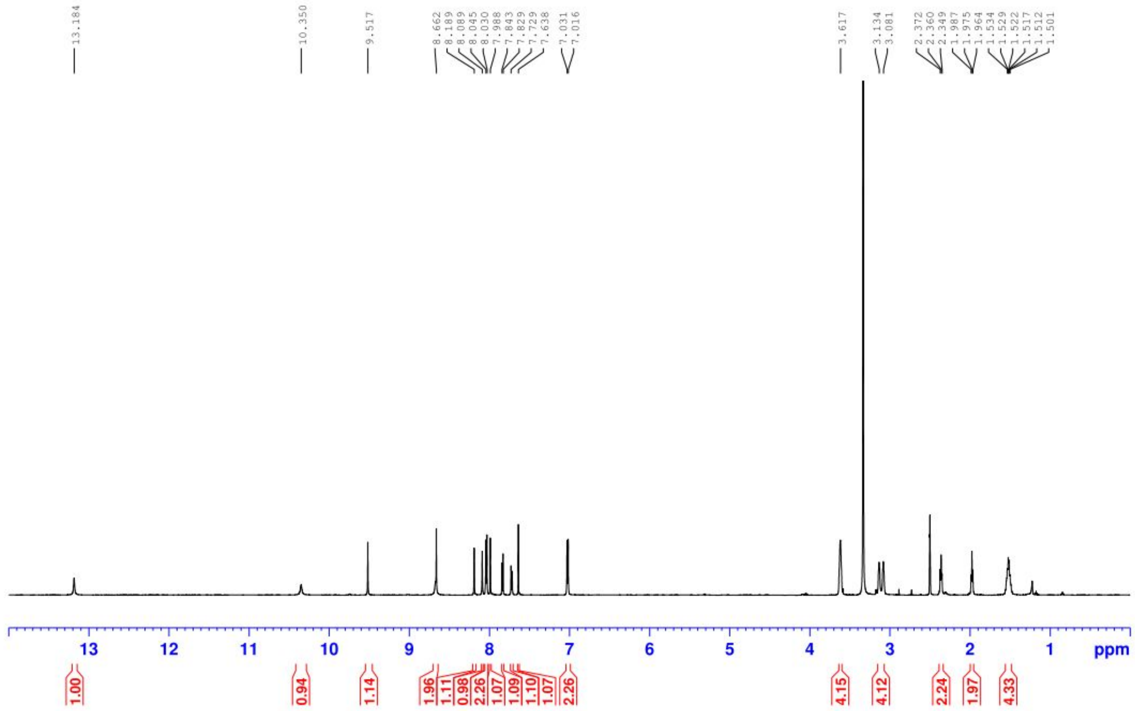

^{1}H NMR of compound 5

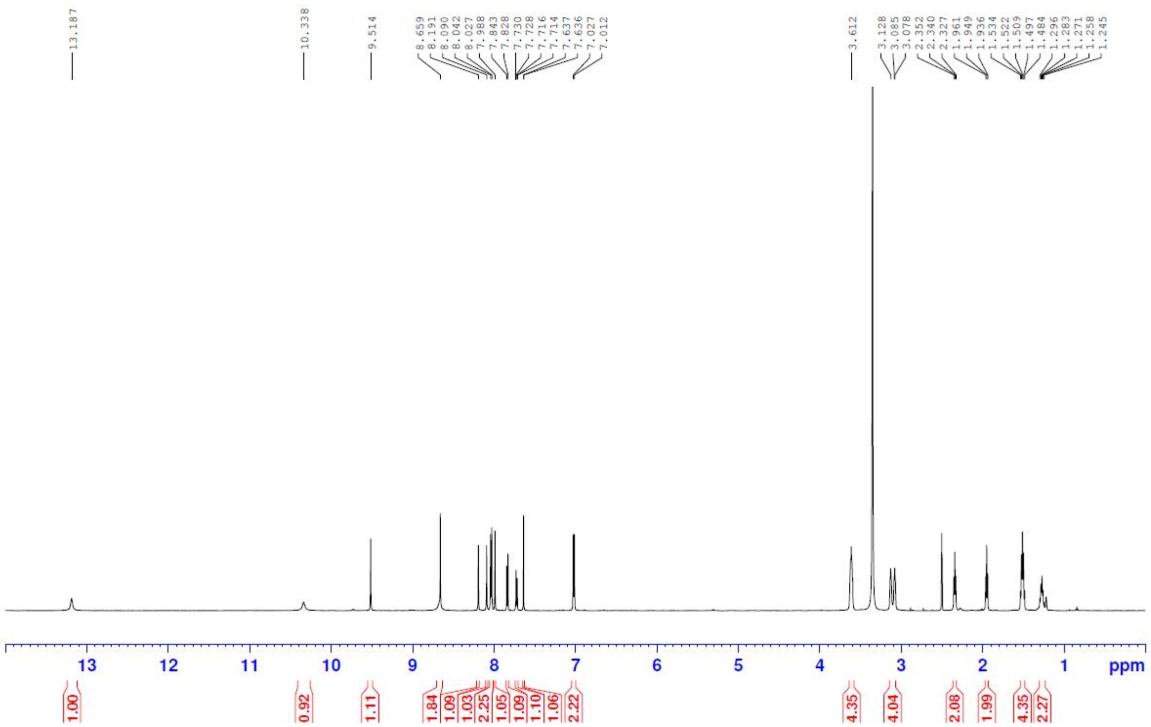

^{1}H NMR of compound 6

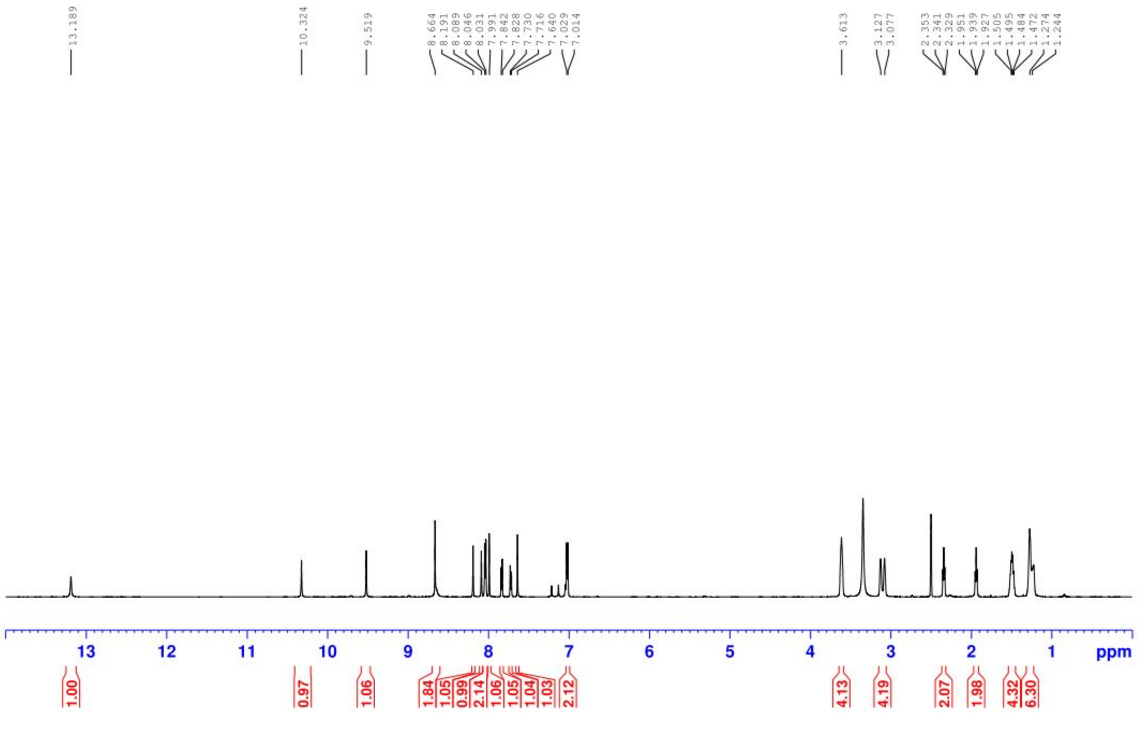

^{1}H NMR of compound 7

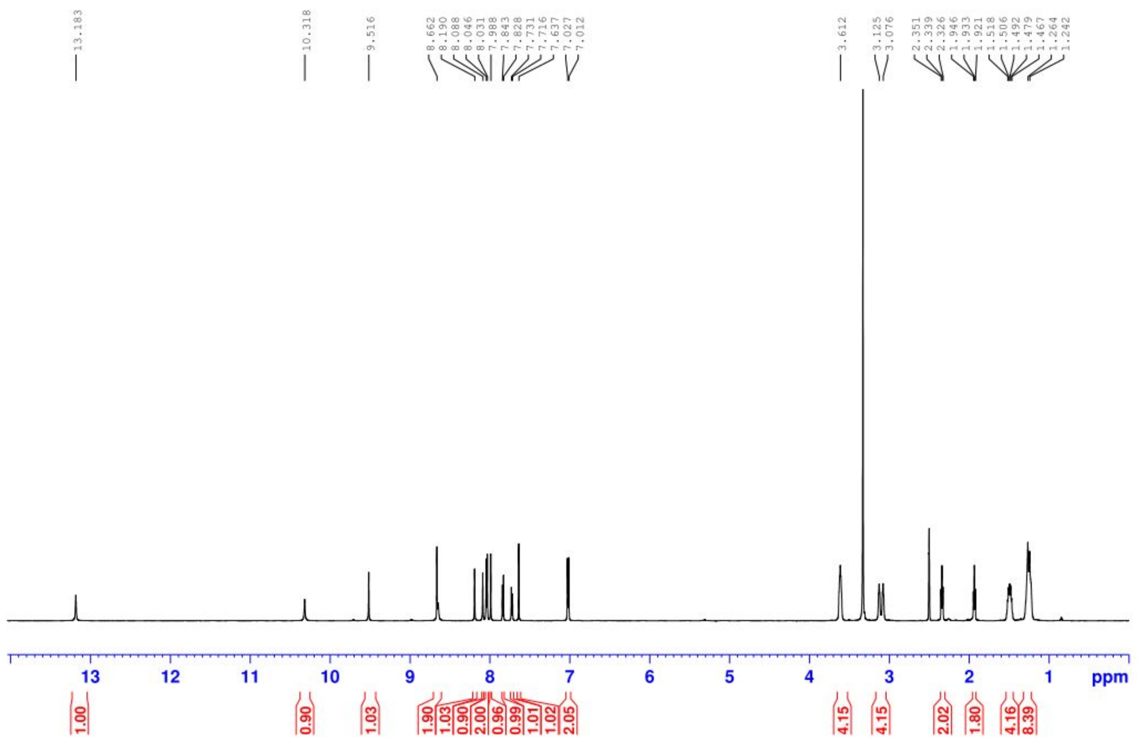

^{1}H NMR of compound 8

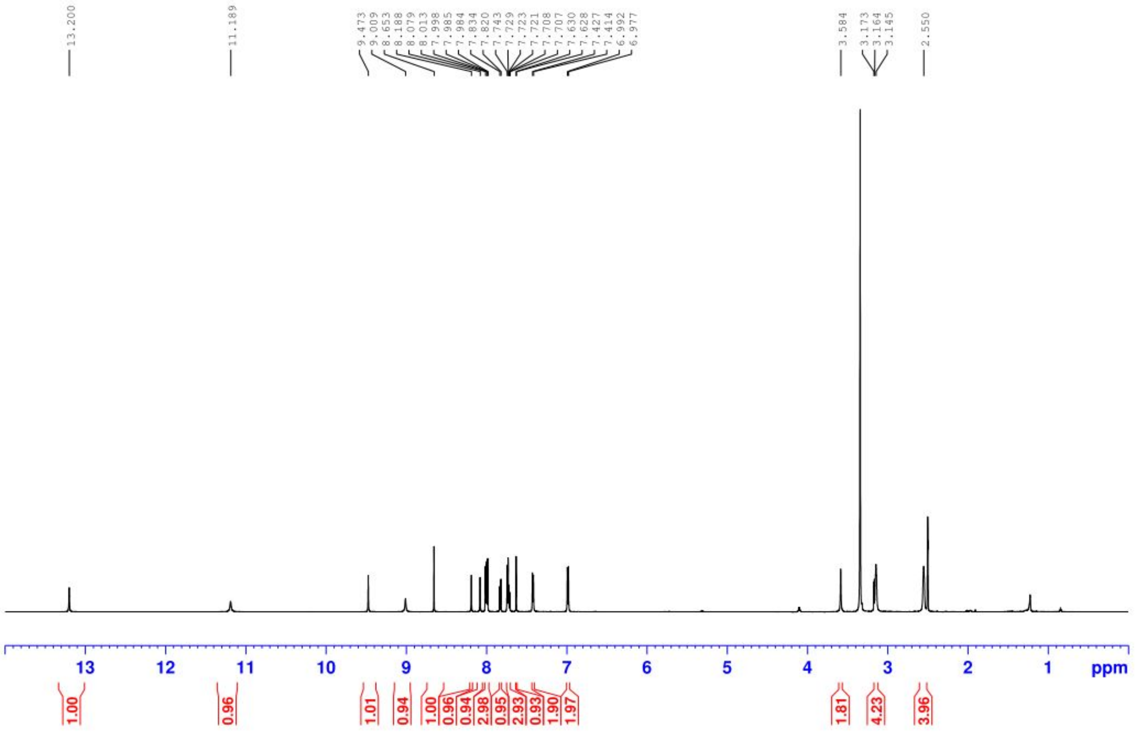

¹H NMR of compound 9

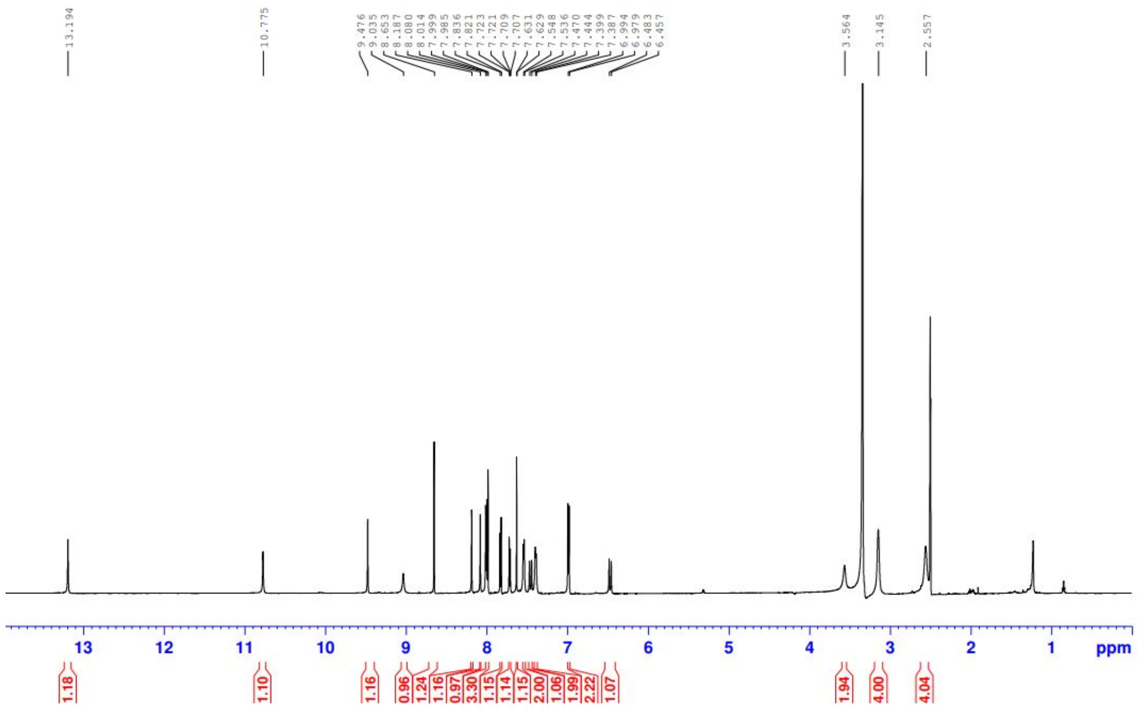

¹H NMR of compound 10

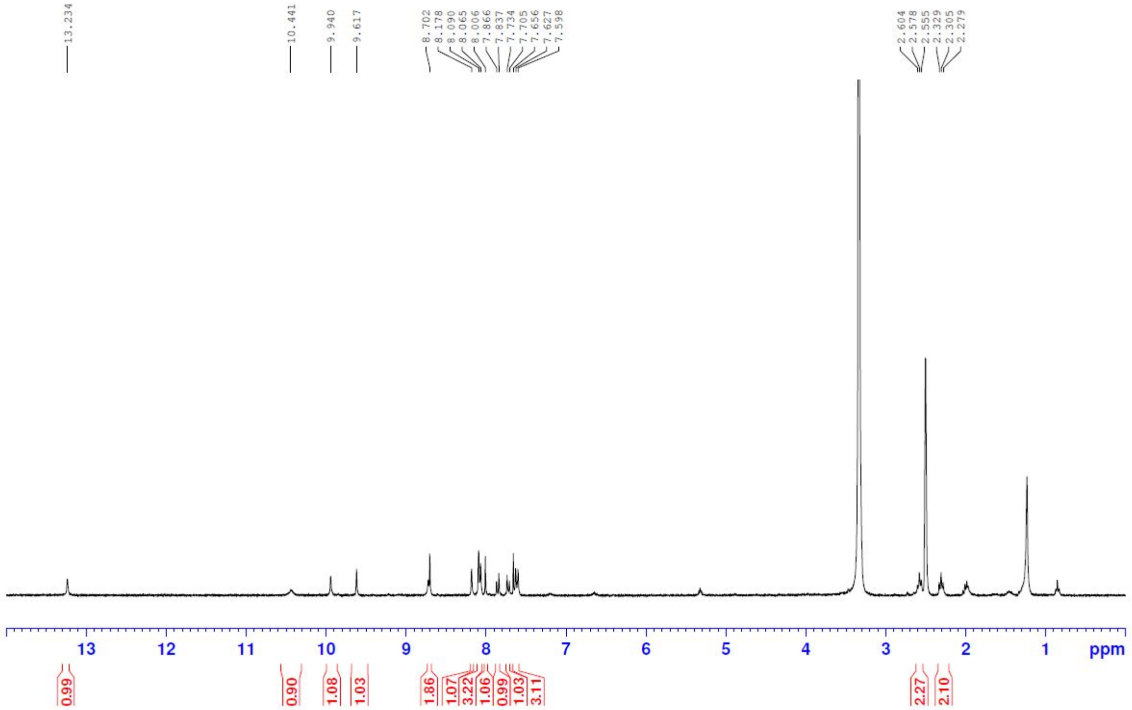

^{1}H NMR of compound 11

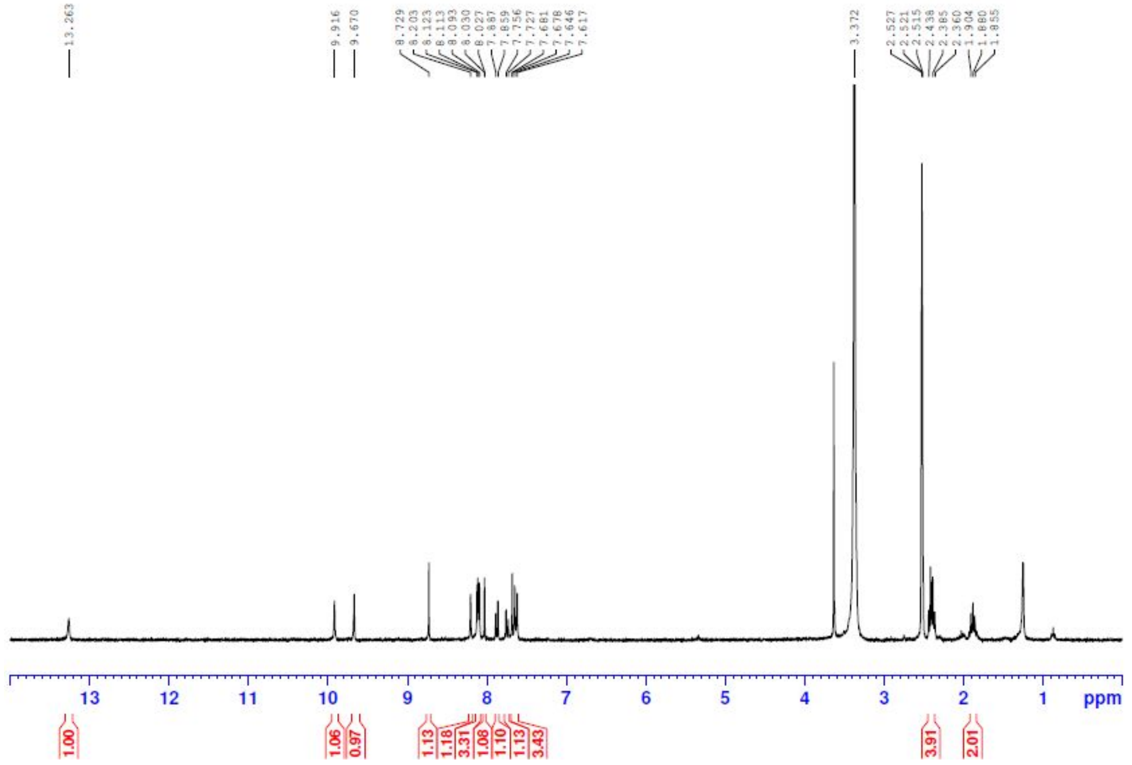

^{1}H NMR of compound 12

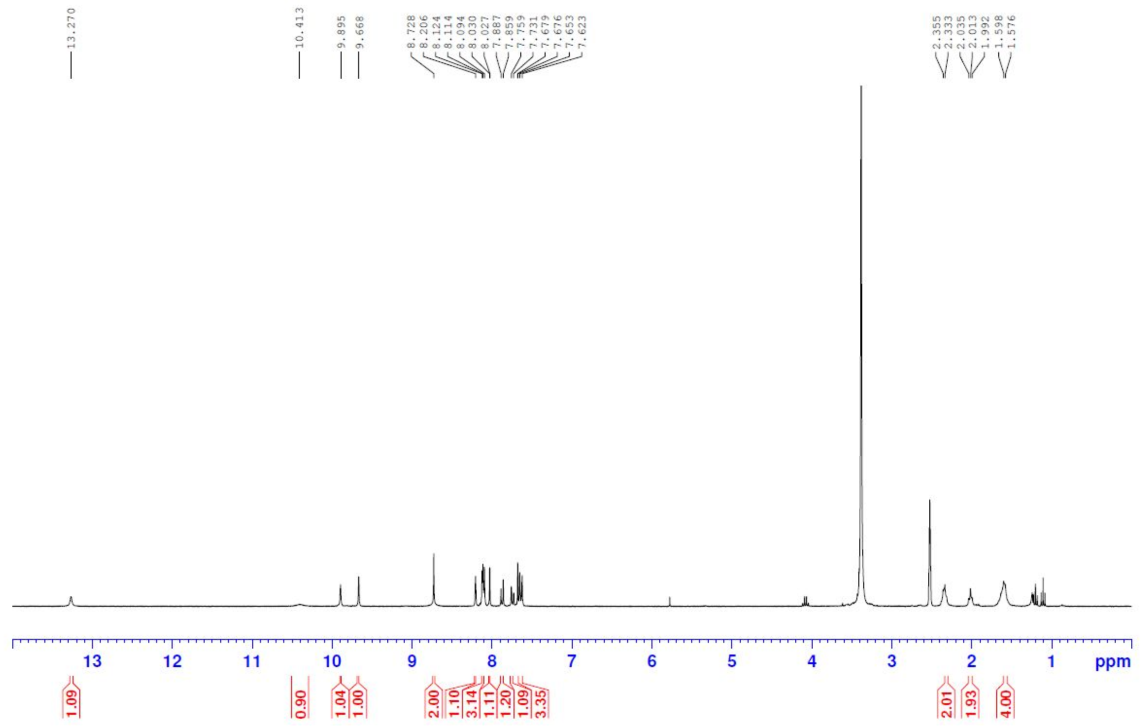

^{1}H NMR of compound 13

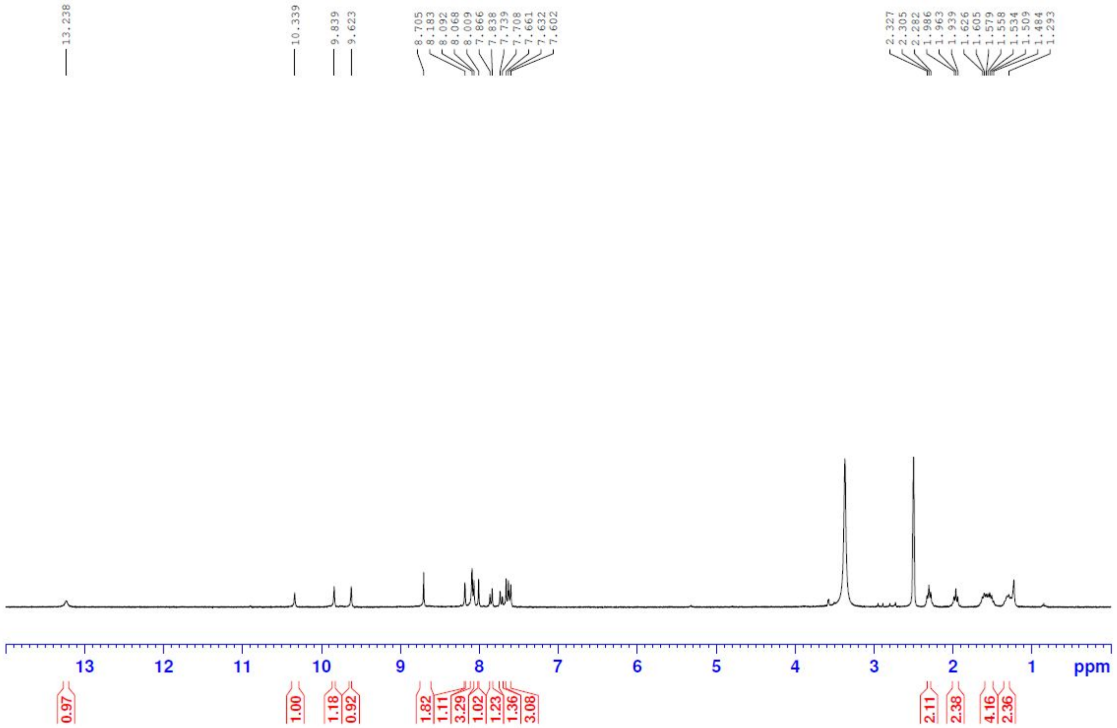

^{1}H NMR of compound 14

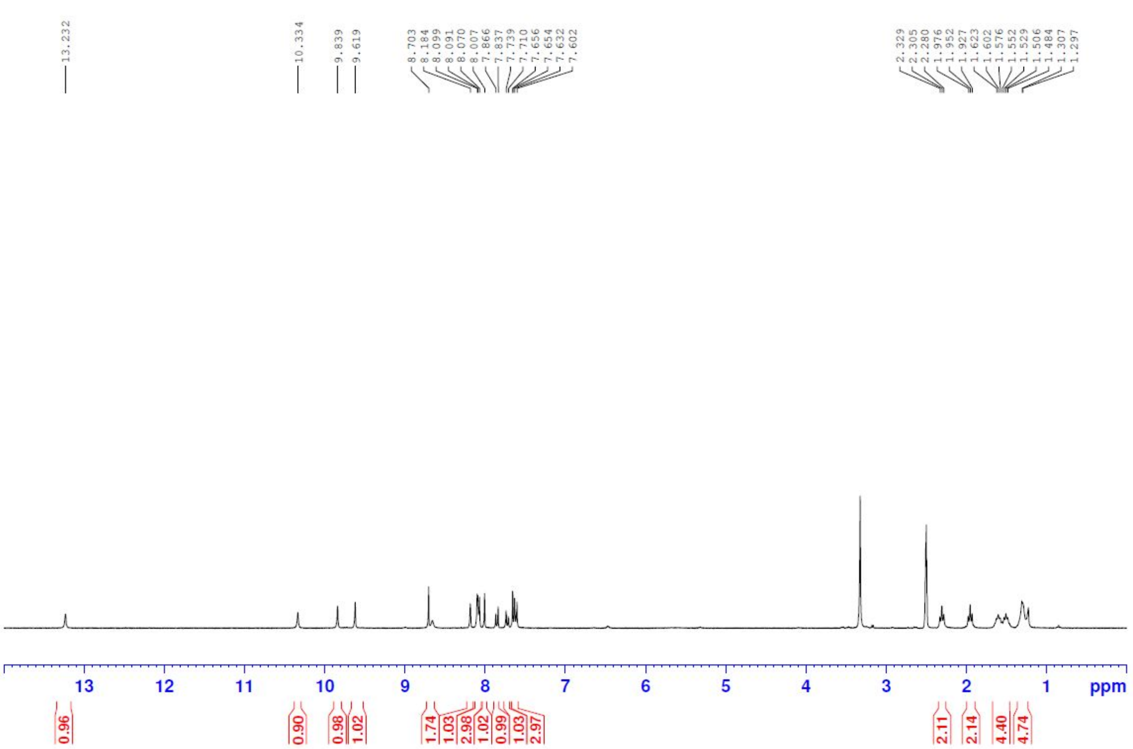

^{1}H NMR of compound 15

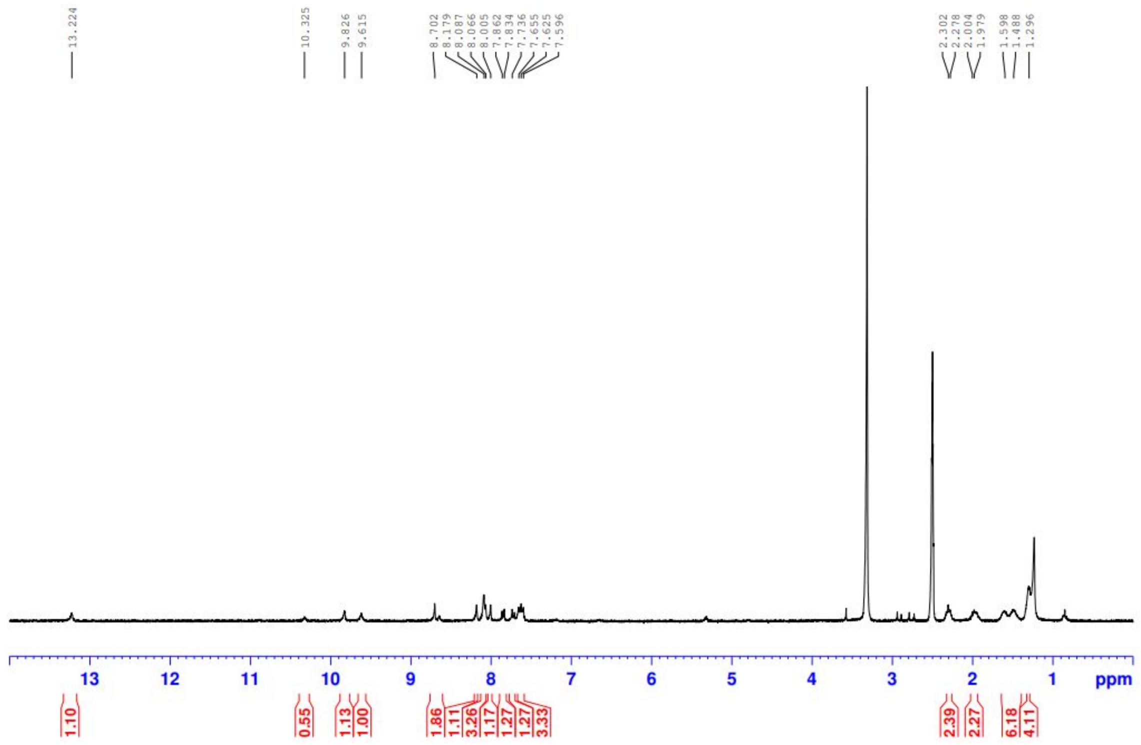

^{1}H NMR of compound 16

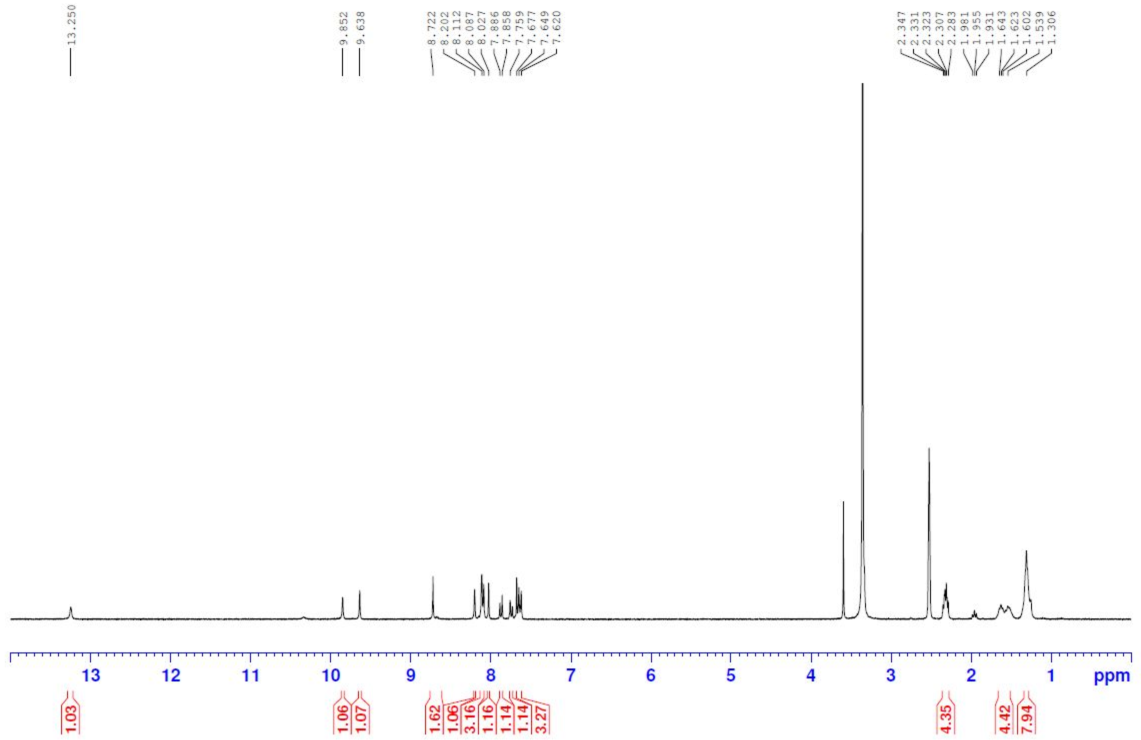

^{1}H NMR of compound 17

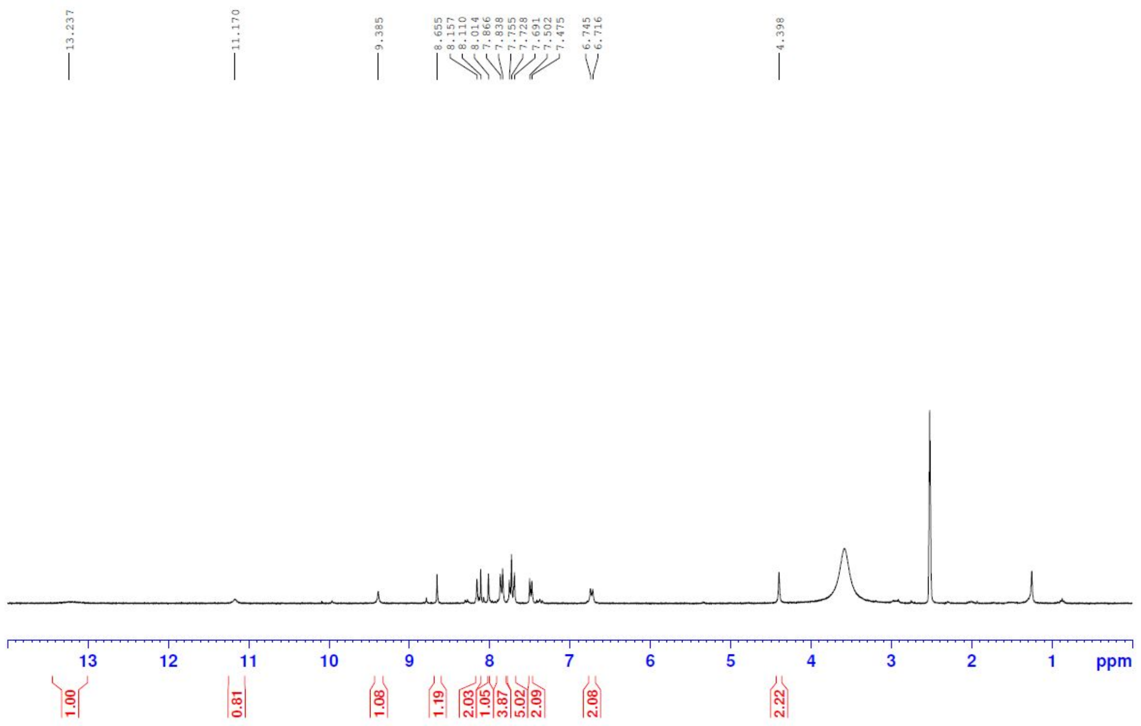

^{1}H NMR of compound 18

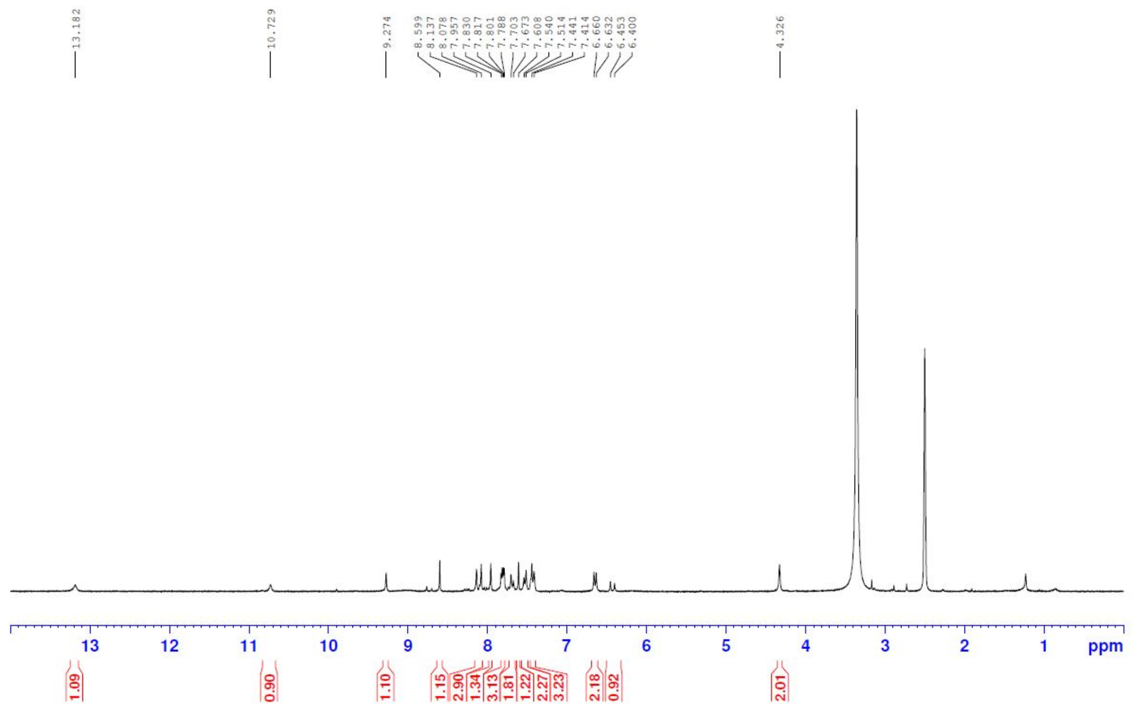

^{13}C NMR of compound 1

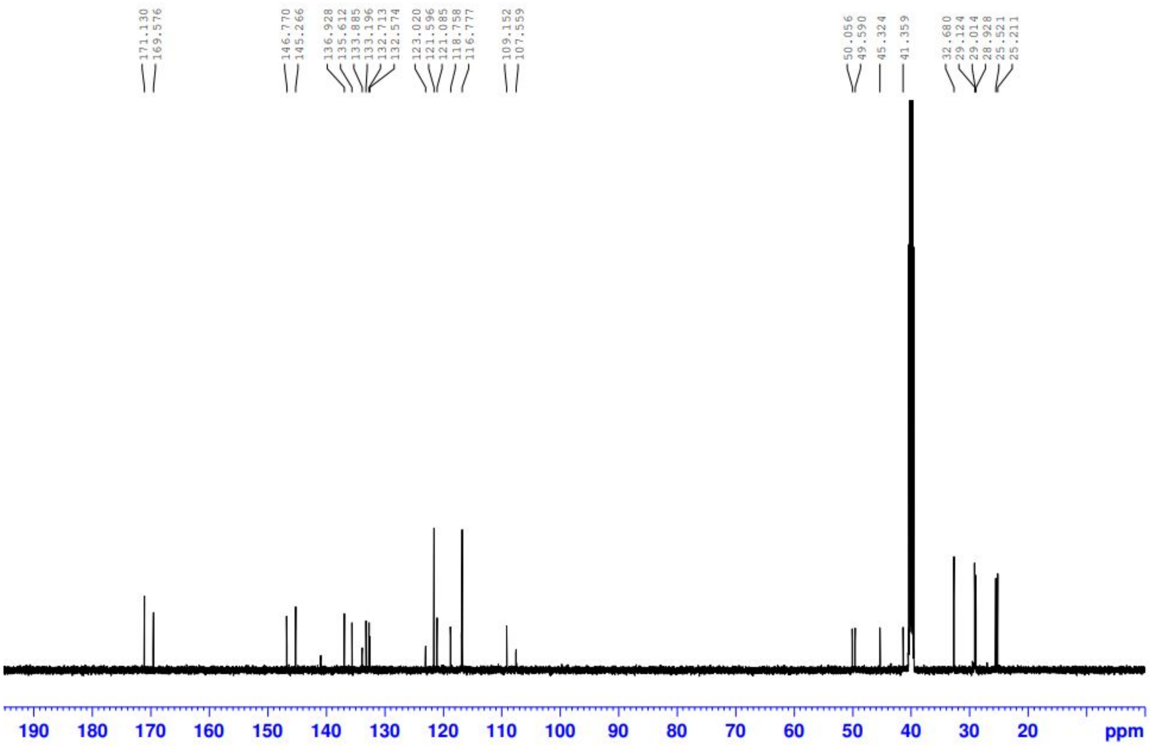

^{13}C NMR of compound 2

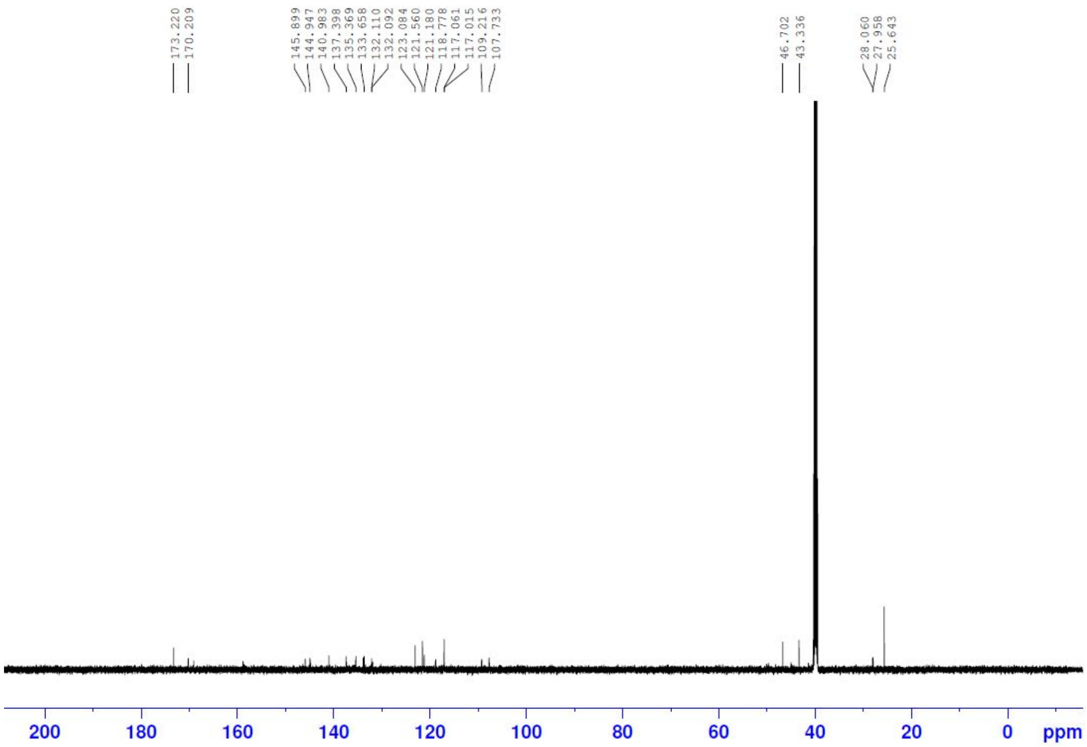

^{13}C NMR of compound 3

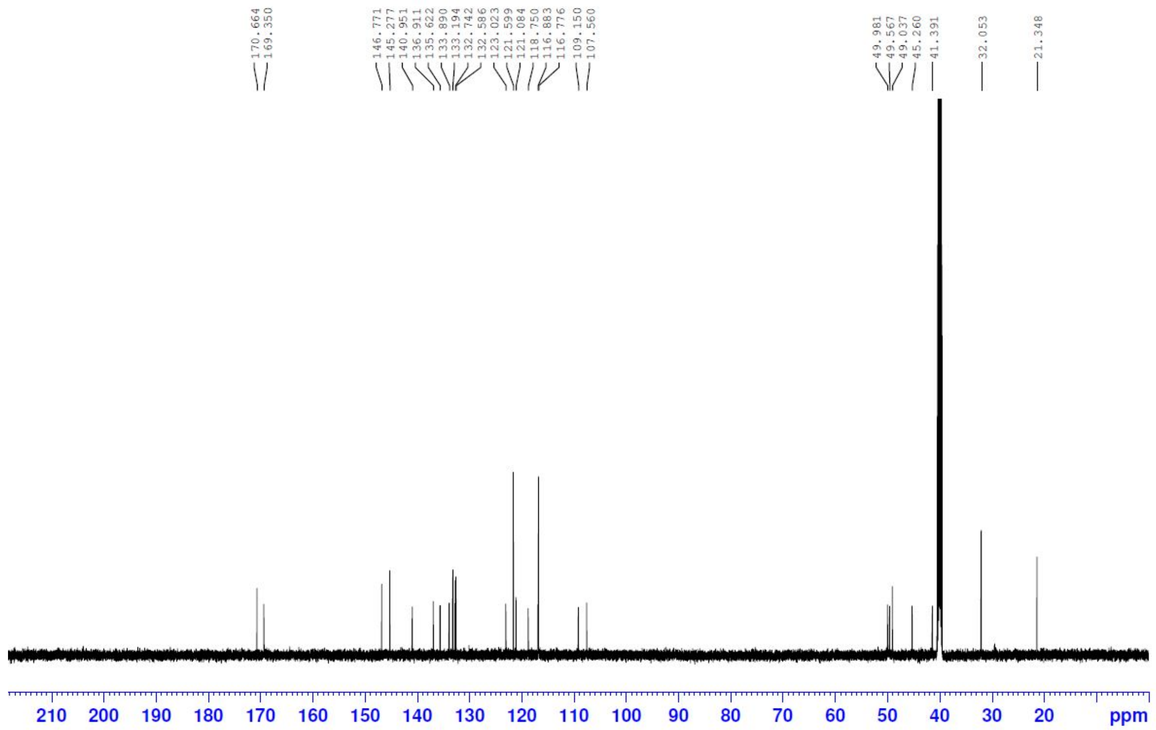

^{13}C NMR of compound 4

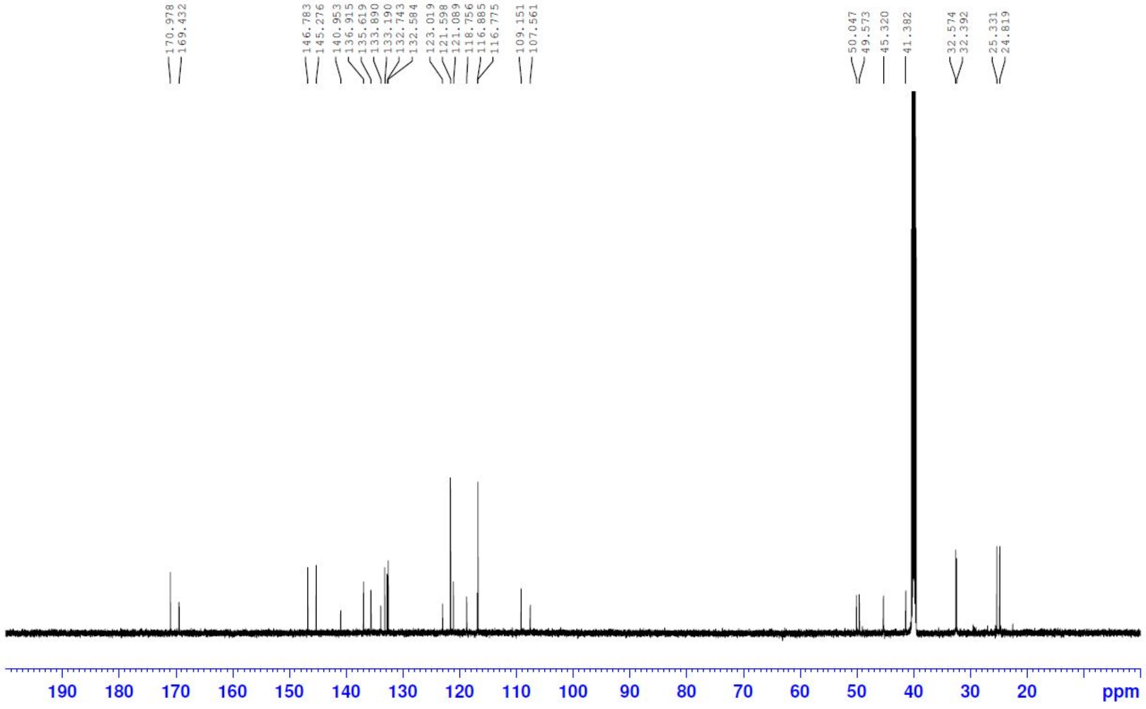

^{13}C NMR of compound 5

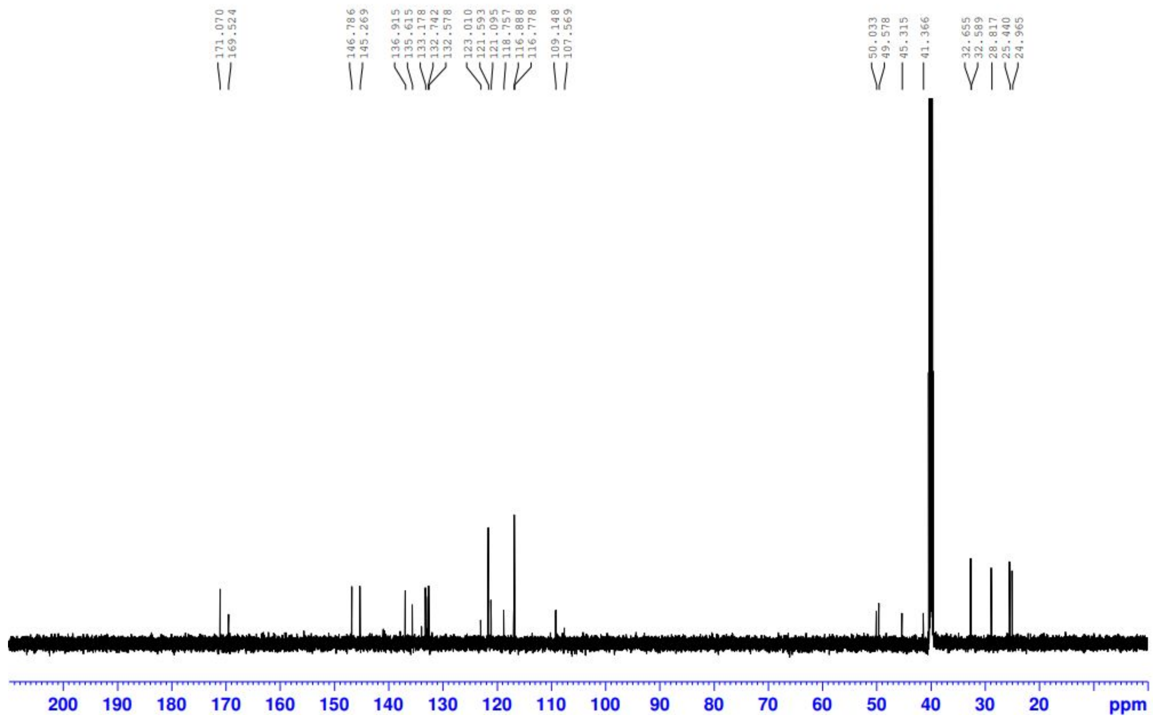

^{13}C NMR of compound 6

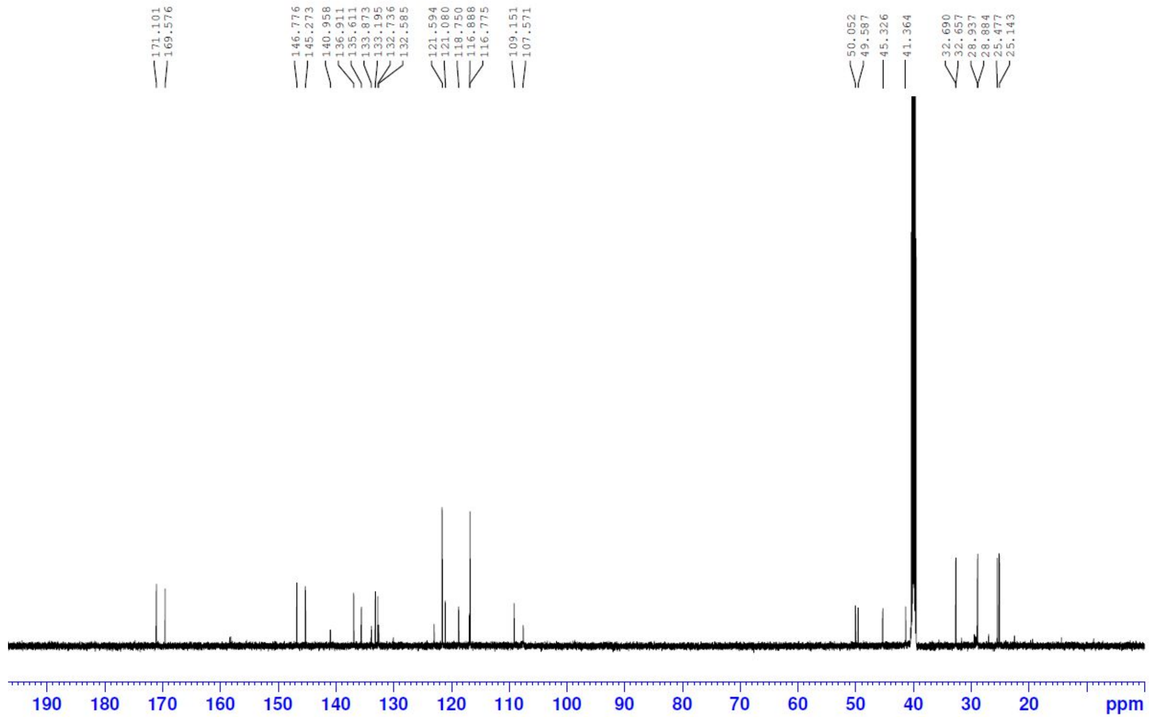

^{13}C NMR of compound 7

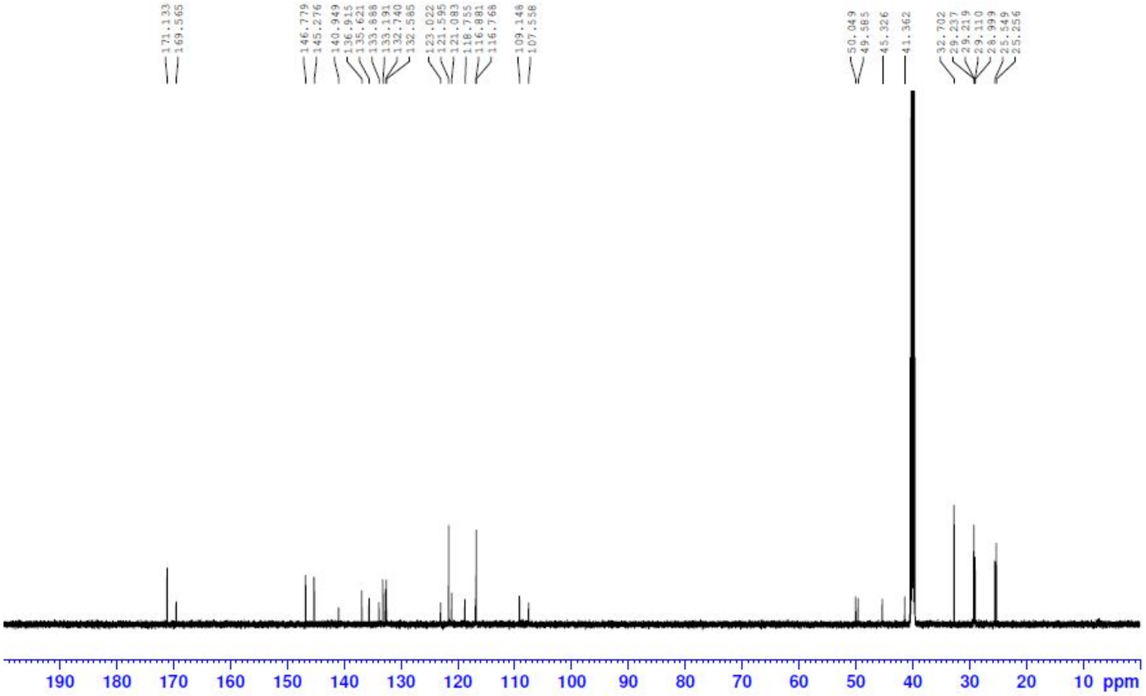

^{13}C NMR of compound 8

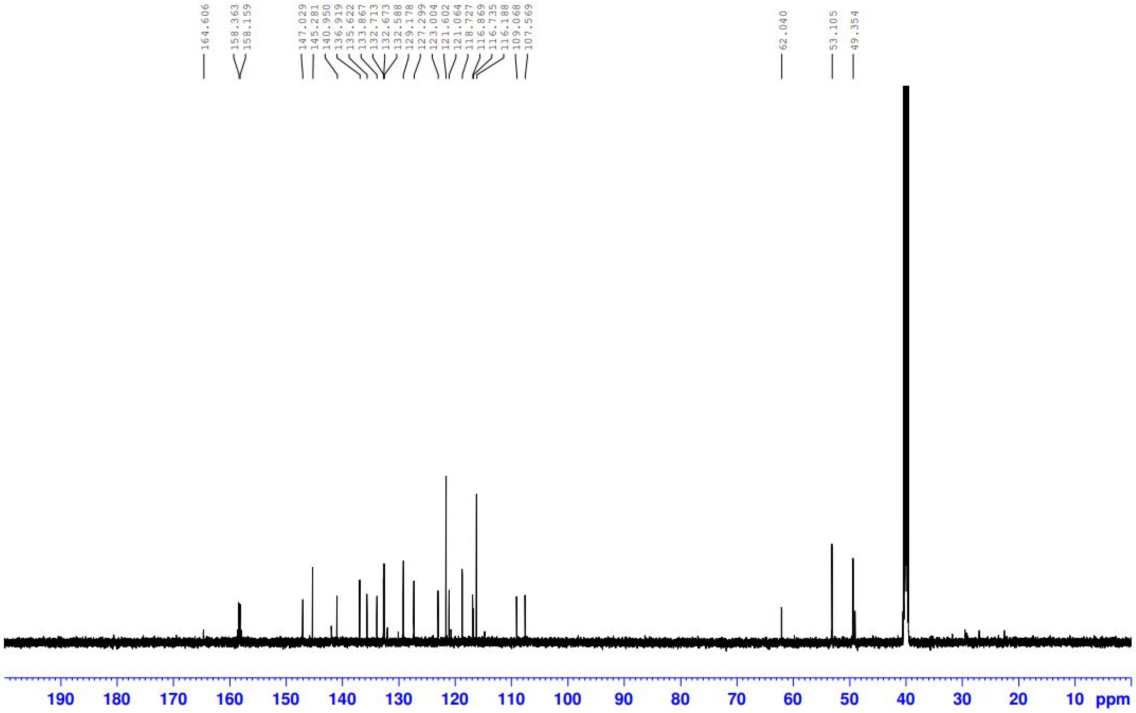

^{13}C NMR of compound 9

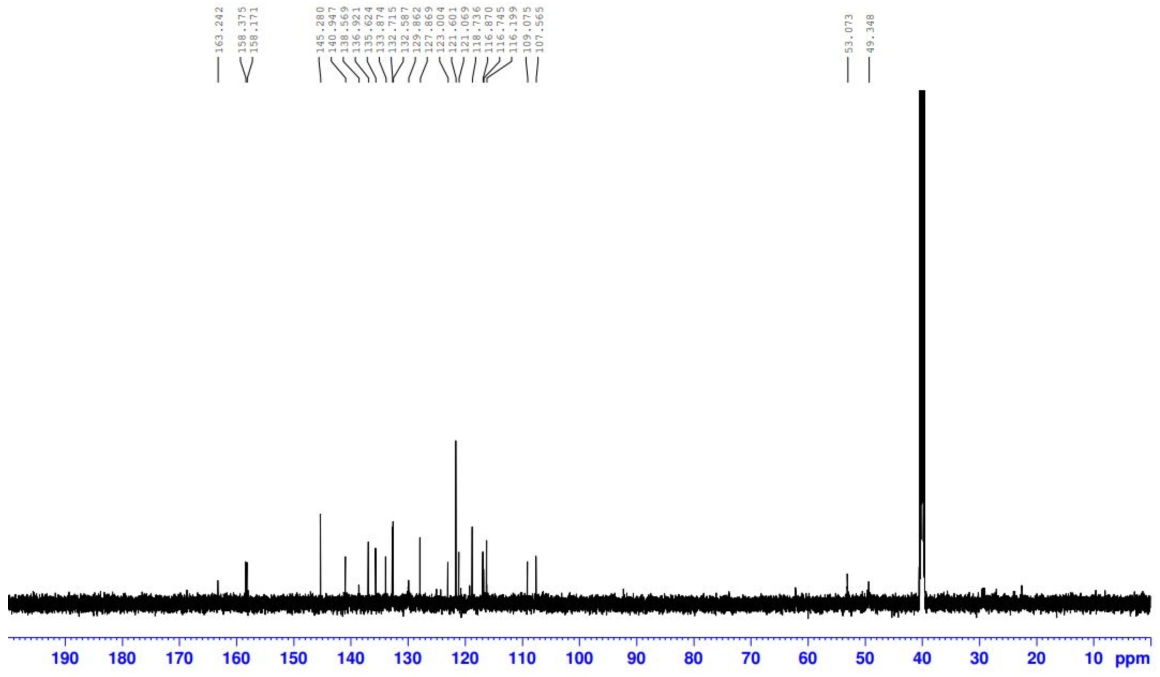

^{13}C NMR of compound 10

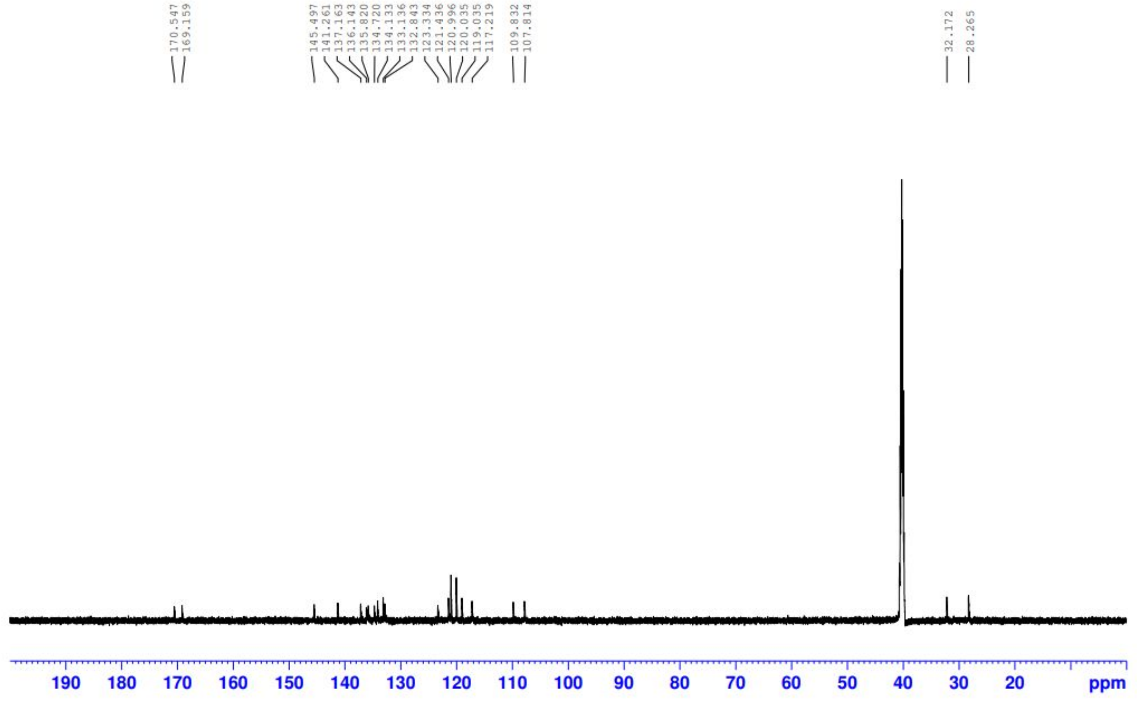

^{13}C NMR of compound 11

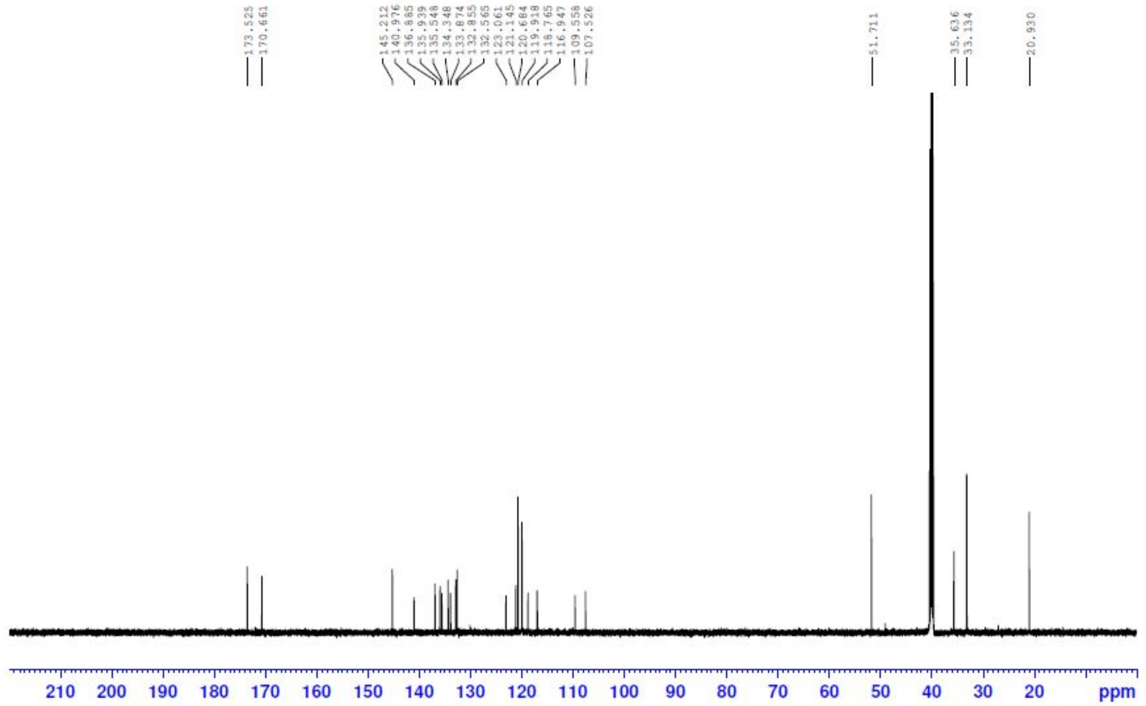

^{13}C NMR of compound 12

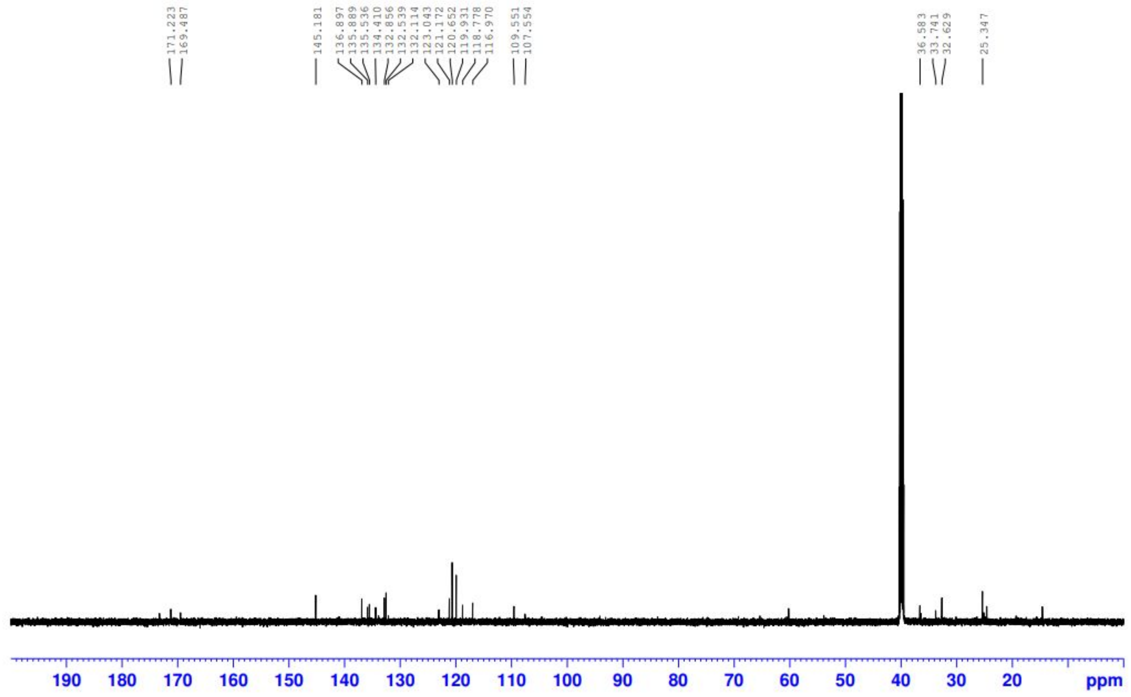

^{13}C NMR of compound 13

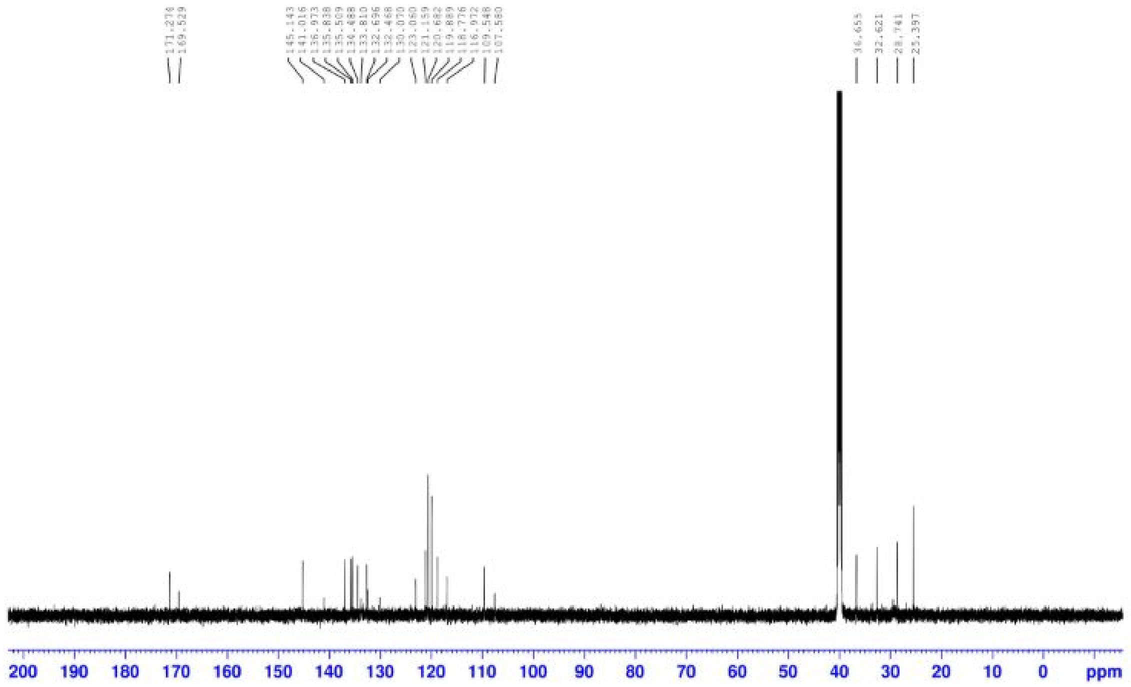

^{13}C NMR of compound 14

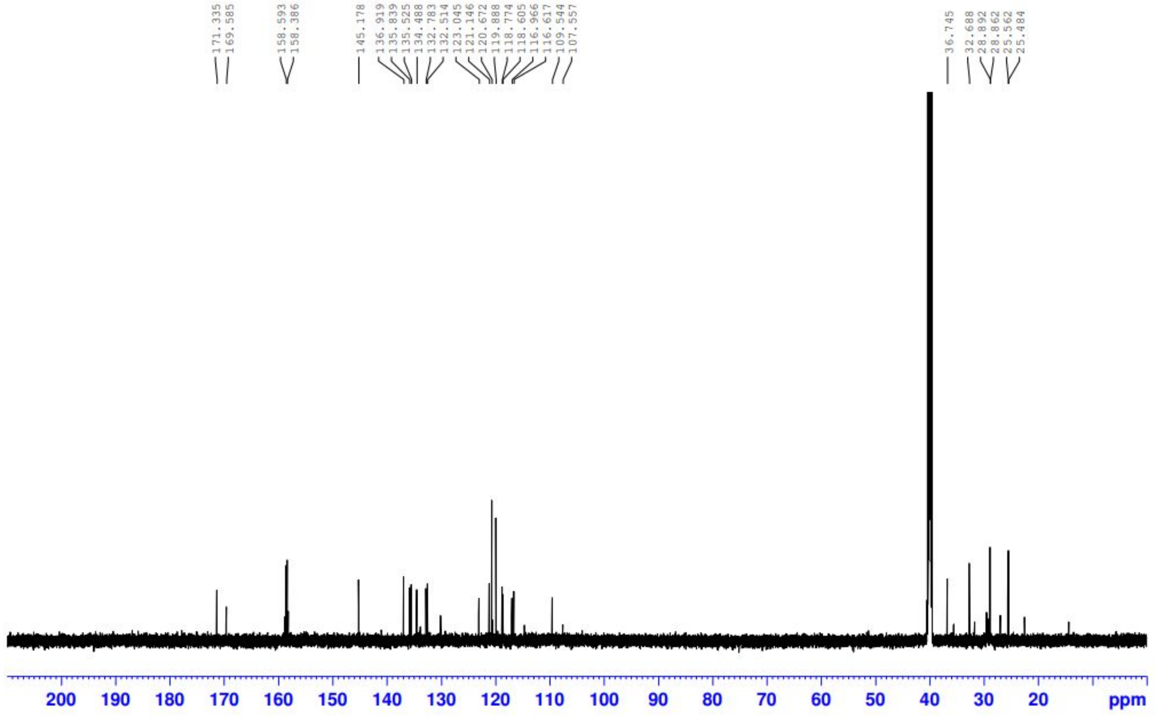

^{13}C NMR of compound 15

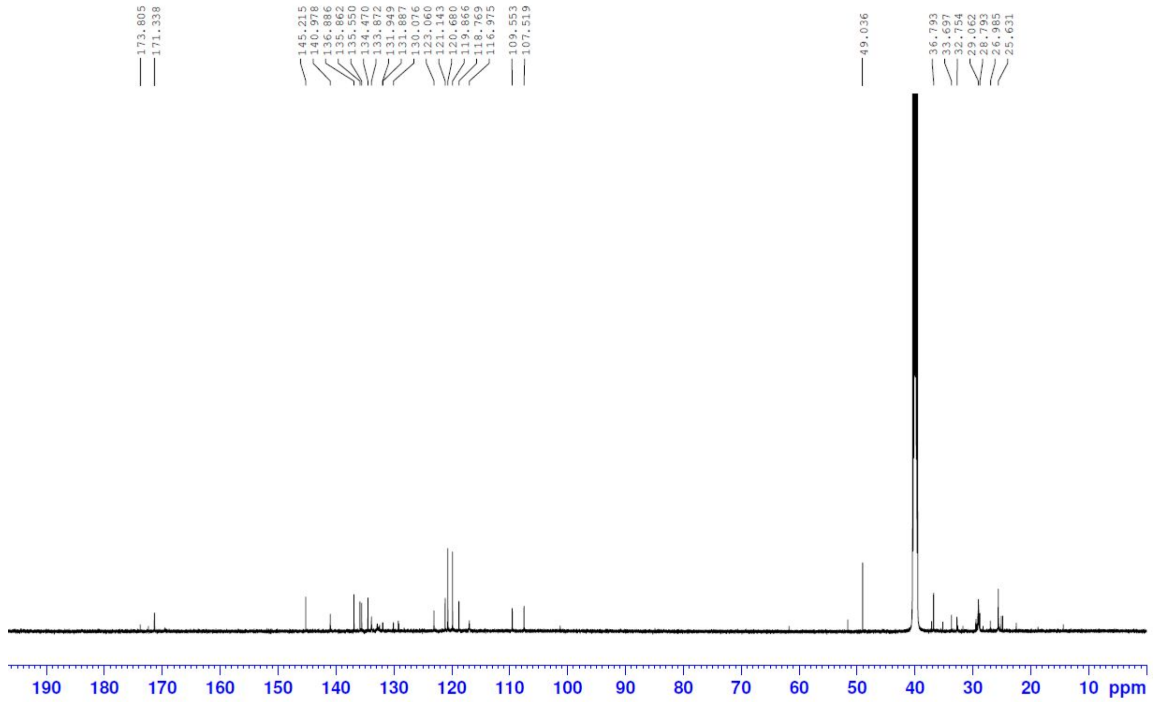

^{13}C NMR of compound 16

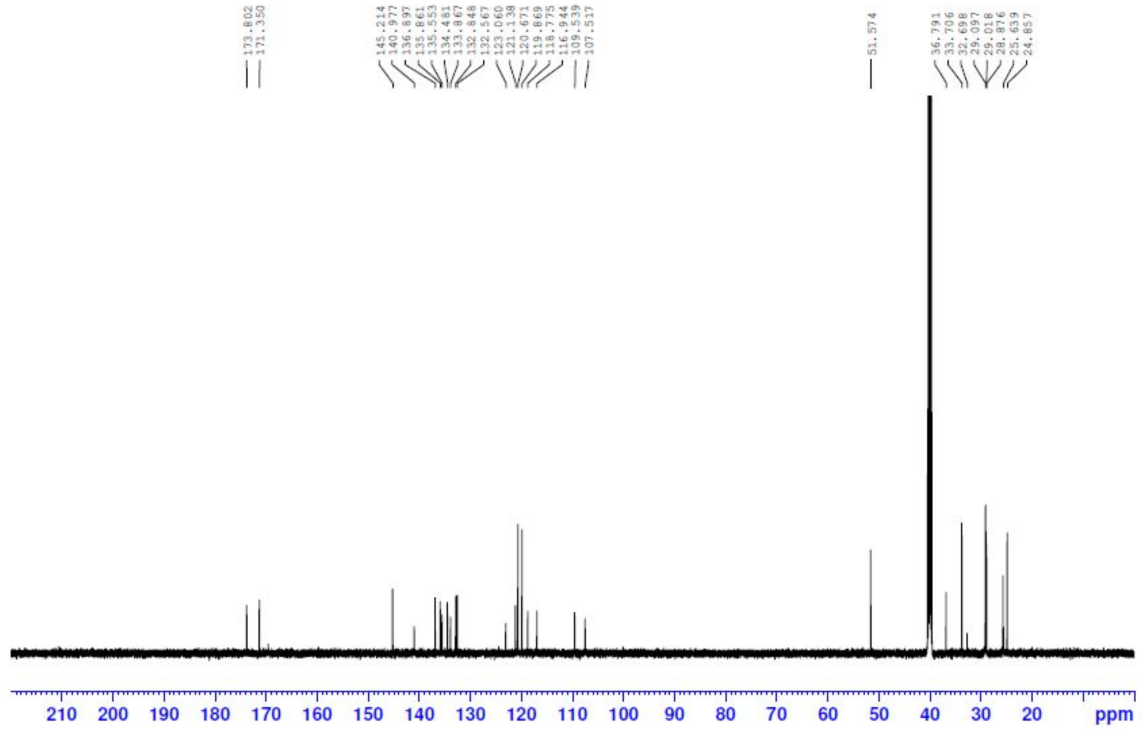

^{13}C NMR of compound 17

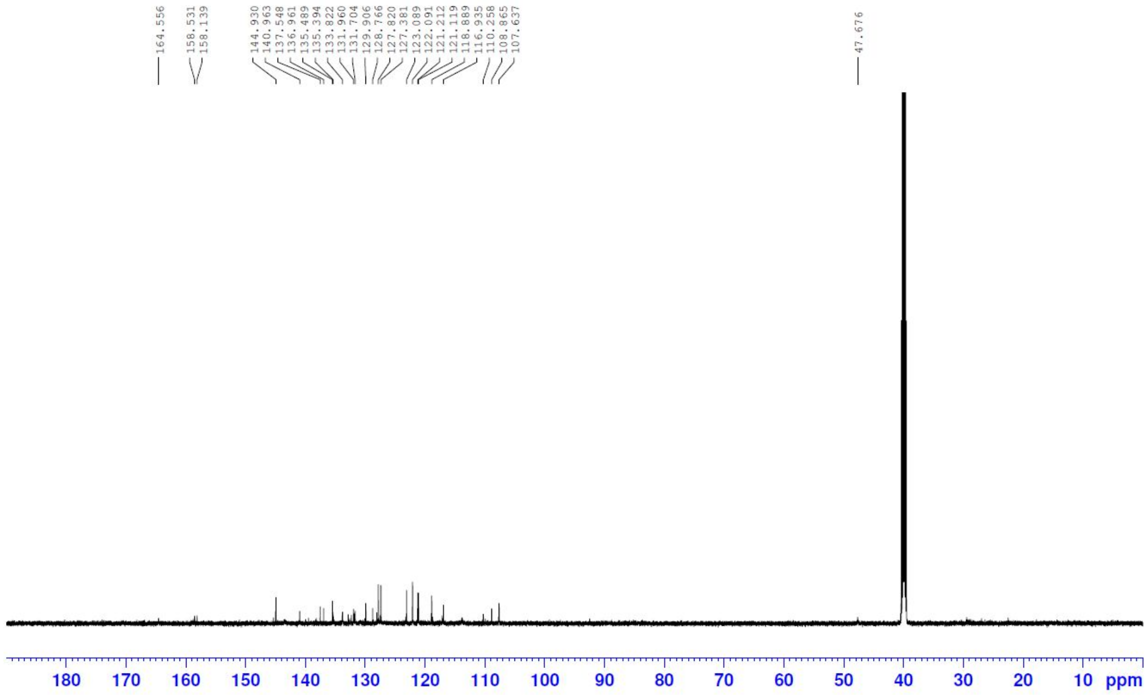

^{13}C NMR of compound 18

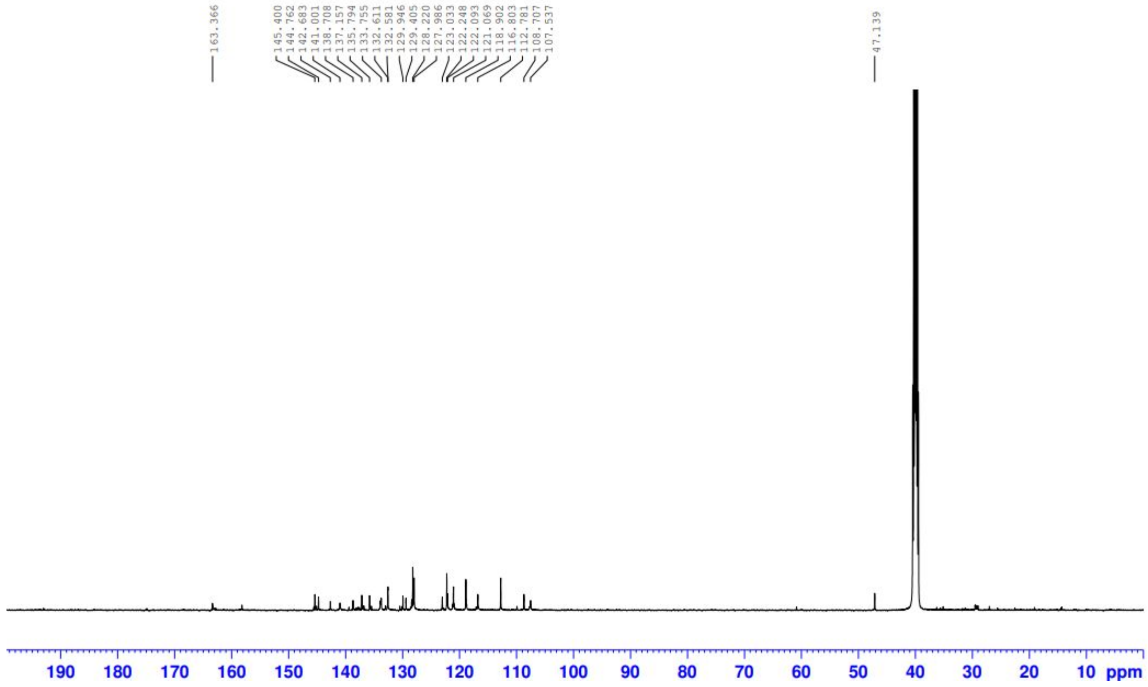

HPLC data of compound 1

```
=====================================================================
Acq. Operator   : JPLpublic                       Seq. Line :   8
Acq. Instrument : FRD-LC-01                        Location :   P2-C-07
Injection Date  : 7/24/2025 10:19:50 PM                 Inj :   1
                                                Inj Volume : 20.000 µl
Method          : C:\Chem32\1\Data\purity_20250724_1 2025-07-24 15-10-18\Purity_original.M (
                  Sequence Method)
Last changed    : 10/21/2024 5:03:57 PM by JPLpublic
Additional Info : Peak(s) manually integrated
```

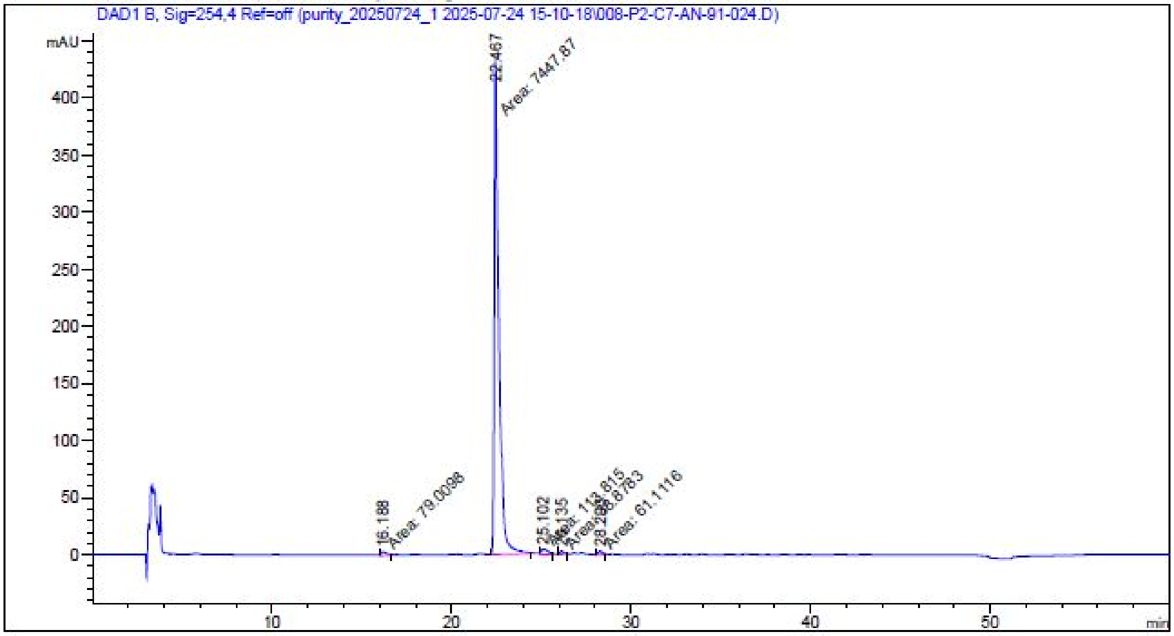


```
=====================================================================
                         Area Percent Report
=====================================================================

Sorted By             :      Signal
Multiplier            :      1.0000
Dilution              :      1.0000
Use Multiplier & Dilution Factor with ISTDs


Signal 1: DAD1 B, Sig=254,4 Ref=off
```

Peak #	RetTime [min]	Type	Width [min]	Area [mAU*s]	Height [mAU]	Area %
1	16.188	MM	0.3701	79.00976	3.55833	1.0207
2	22.467	MM	0.2859	7447.87354	434.15167	96.2172
3	25.102	MM	0.4446	113.81532	4.26701	1.4704
4	26.135	MM	0.2448	38.87826	2.64669	0.5023
5	28.293	MM	0.3394	61.11157	3.00098	0.7895

HPLC data of compound 2

```
=====================================================================
Acq. Operator   : JPLpublic                        Seq. Line :  11
Acq. Instrument : FRD-LC-01                         Location :   P2-C-10
Injection Date  : 7/25/2025 1:23:28 AM                   Inj :   1
                                                 Inj Volume : 20.000 µl
Method          : C:\Chem32\1\Data\purity_20250724_1 2025-07-24 15-10-18\Purity_original.M (
                  Sequence Method)
Last changed    : 10/21/2024 5:03:57 PM by JPLpublic
Additional Info : Peak(s) manually integrated
```

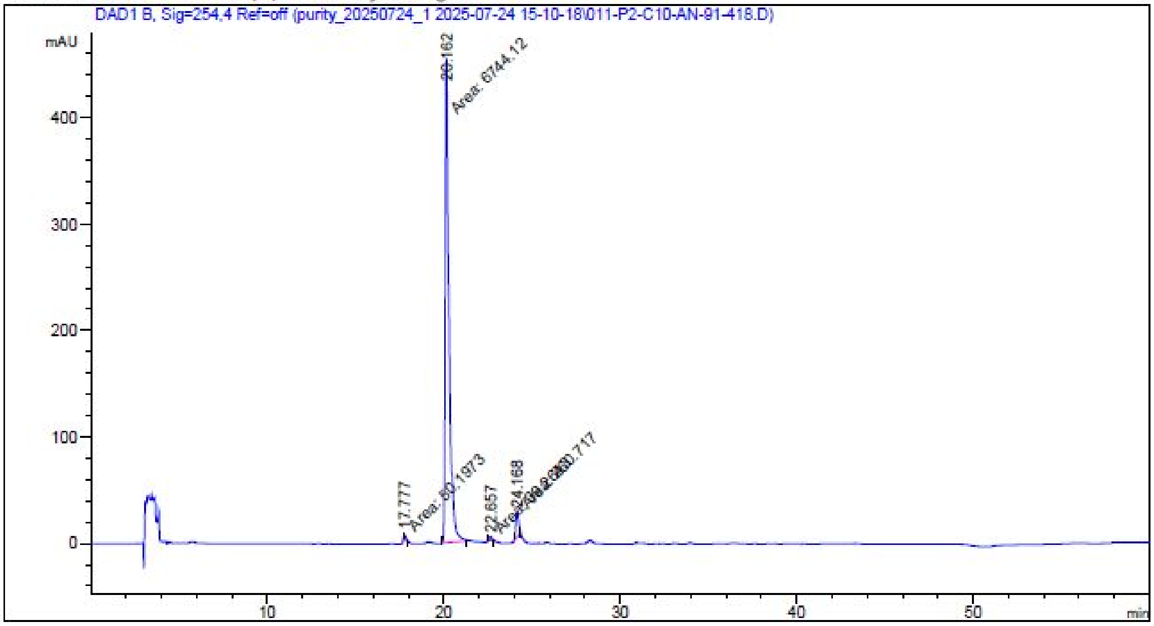


```
=====================================================================
                         Area Percent Report
=====================================================================

Sorted By             :      Signal
Multiplier            :      1.0000
Dilution              :      1.0000
Use Multiplier & Dilution Factor with ISTDs


Signal 1: DAD1 B, Sig=254,4 Ref=off
```

Peak #	RetTime [min]	Type	Width [min]	Area [mAU*s]	Height [mAU]	Area %
1	17.777	MM	0.1294	50.19734	6.46564	0.7076
2	20.162	MM	0.2468	6744.12012	455.44757	95.0639
3	22.657	MM	0.1753	39.26425	3.73234	0.5535
4	24.168	MM	0.1820	260.71738	23.87709	3.6750

HPLC data of compound 3

```
=====================================================================
Acq. Operator   : JPLpublic                     Seq. Line :  10
Acq. Instrument : FRD-LC-01                      Location :   P2-C-09
Injection Date  : 7/25/2025 12:22:15 AM               Inj :   1
                                               Inj Volume : 20.000 µl
Method          : C:\Chem32\1\Data\purity_20250724_1 2025-07-24 15-10-18\Purity_original.M (
                  Sequence Method)
Last changed    : 10/21/2024 5:03:57 PM by JPLpublic
Additional Info : Peak(s) manually integrated
```

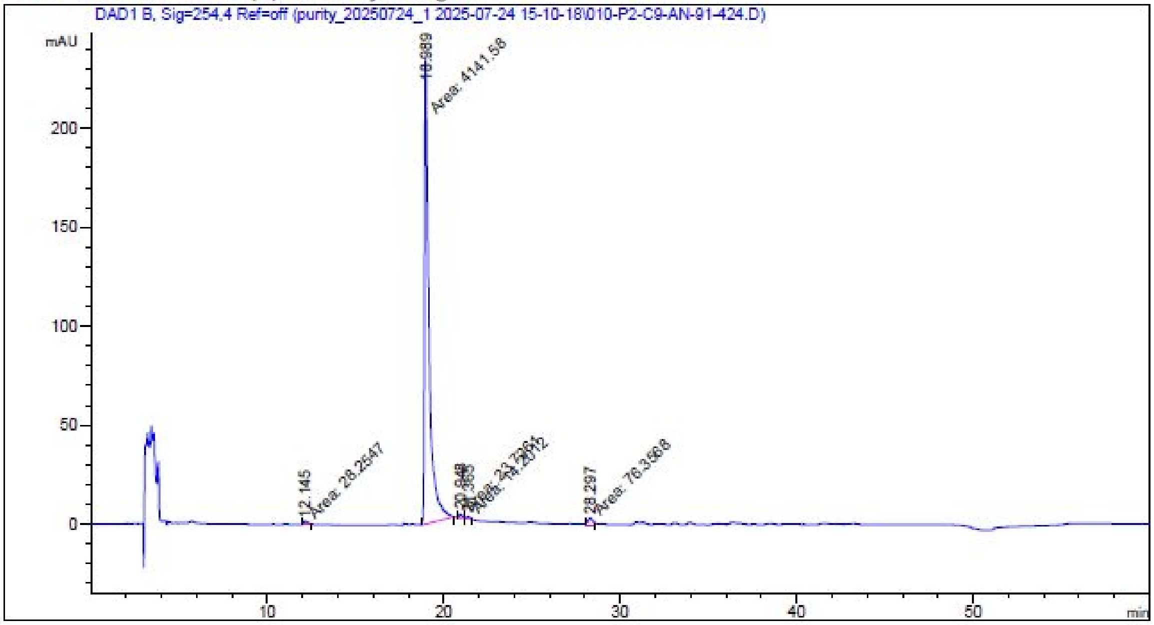


```
=====================================================================
                         Area Percent Report
=====================================================================

Sorted By             :      Signal
Multiplier            :      1.0000
Dilution              :      1.0000
Use Multiplier & Dilution Factor with ISTDs


Signal 1: DAD1 B, Sig=254,4 Ref=off

Peak RetTime Type  Width     Area      Height     Area
  #   [min]        [min]   [mAU*s]     [mAU]        %
----|-------|----|-------|----------|----------|--------|
   1  12.145 MM    0.2696   28.25472    1.74642   0.6595
   2  18.989 MM    0.2926 4141.57959  235.90146  96.6729
   3  20.948 MM    0.1946   23.72614    2.03239   0.5538
   4  21.365 MM    0.2054   14.20125    1.15240   0.3315
   5  28.297 MM    0.3646   76.35682    3.49079   1.7823
```

HPLC data of compound 4

```
=====================================================================
Acq. Operator   : JPLpublic                       Seq. Line :    7
Acq. Instrument : FRD-LC-01                        Location :   P1-D-07
Injection Date  : 7/23/2025 8:09:30 PM                  Inj :    1
                                               Inj Volume : 20.000 µl
Method          : C:\Chem32\1\Data\purity_20250723-098-1 2025-07-23 14-00-47\Purity_original.
                  M (Sequence Method)
Last changed    : 10/21/2024 5:03:57 PM by JPLpublic
Additional Info : Peak(s) manually integrated
```

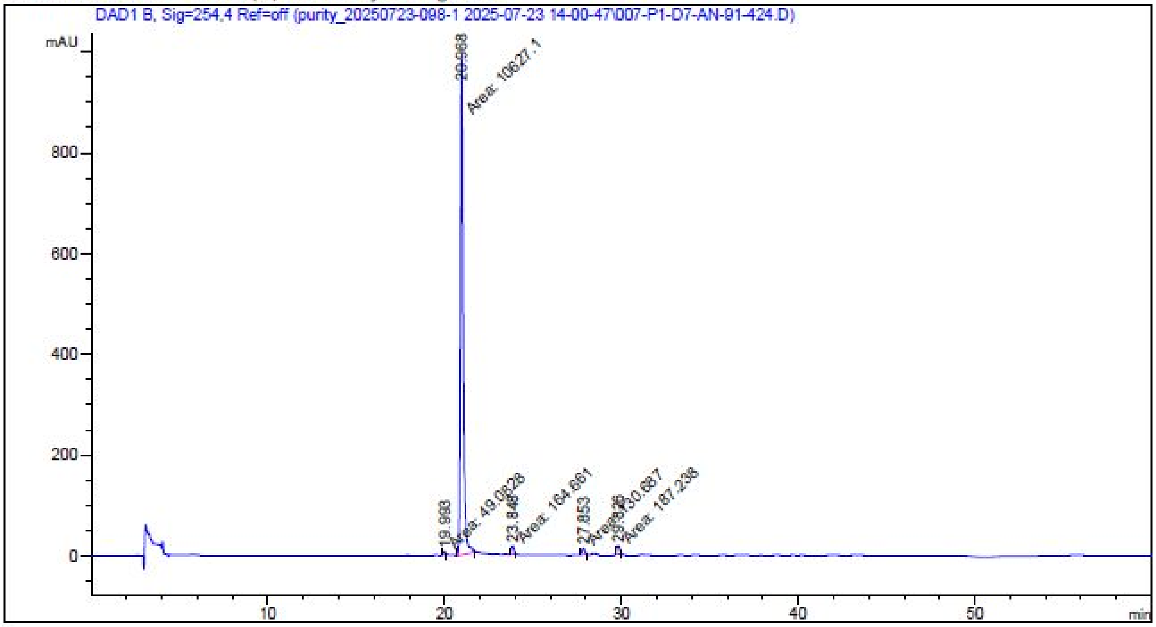


```
=====================================================================
                         Area Percent Report
=====================================================================

Sorted By             :      Signal
Multiplier            :      1.0000
Dilution              :      1.0000
Use Multiplier & Dilution Factor with ISTDs


Signal 1: DAD1 B, Sig=254,4 Ref=off
```

Peak #	RetTime [min]	Type	Width [min]	Area [mAU*s]	Height [mAU]	Area %
1	19.993	MM	0.1312	49.08284	6.23482	0.4399
2	20.968	MM	0.1795	1.06271e4	986.88605	95.2354
3	23.848	MM	0.1690	164.66135	16.24269	1.4756
4	27.853	MM	0.1878	130.68732	11.59792	1.1712
5	29.826	MM	0.1934	187.23787	16.13766	1.6779

HPLC data of compound 5

```
=====================================================================
Acq. Operator   : JPLpublic                        Seq. Line :   7
Acq. Instrument : FRD-LC-01                         Location :   P2-C-06
Injection Date  : 7/24/2025 9:18:38 PM                   Inj :   1
                                                  Inj Volume : 20.000 µl
Method          : C:\Chem32\1\Data\purity_20250724_1 2025-07-24 15-10-18\Purity_original.M (
                  Sequence Method)
Last changed    : 10/21/2024 5:03:57 PM by JPLpublic
Additional Info : Peak(s) manually integrated
```

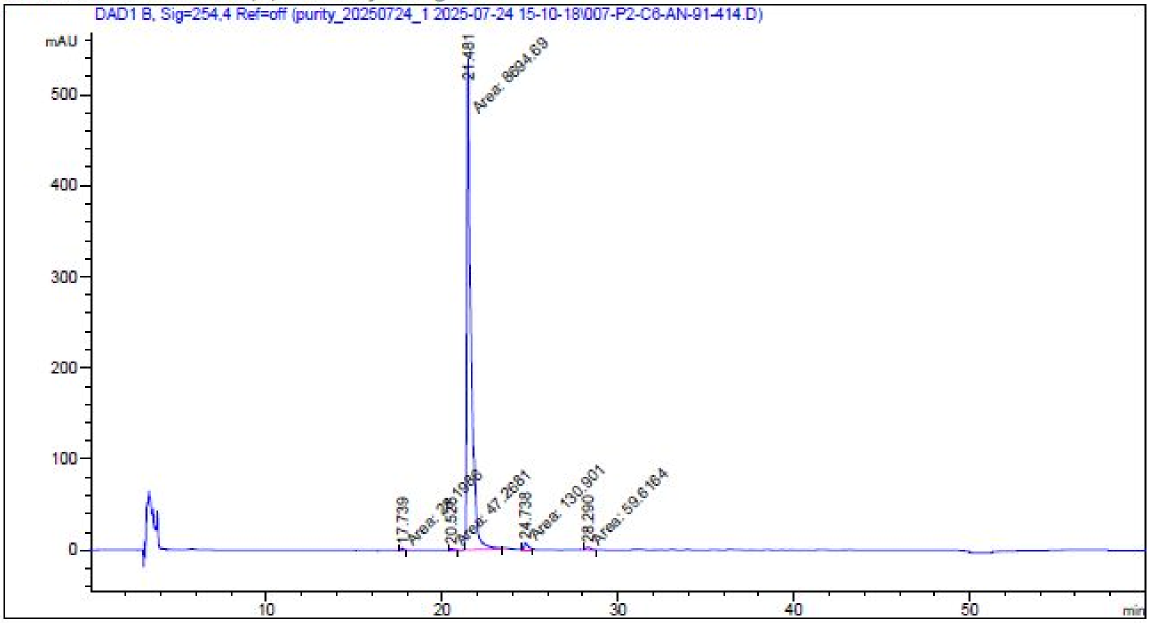


```
=====================================================================
                         Area Percent Report
=====================================================================

Sorted By             :      Signal
Multiplier            :      1.0000
Dilution              :      1.0000
Use Multiplier & Dilution Factor with ISTDs


Signal 1: DAD1 B, Sig=254,4 Ref=off
```

Peak #	RetTime [min]	Type	Width [min]	Area [mAU*s]	Height [mAU]	Area %
1	17.739	MM	0.2047	28.19858	2.29586	0.3147
2	20.525	MM	0.3243	47.26805	2.42890	0.5275
3	21.481	MM	0.2680	8694.68945	540.79144	97.0316
4	24.738	MM	0.2828	130.90135	7.71370	1.4608
5	28.290	MM	0.3554	59.61641	2.79551	0.6653

HPLC data of compound 6

```
======================================================================
Acq. Operator   : JPLpublic                    Seq. Line :  12
Acq. Instrument : FRD-LC-01                     Location :   P2-C-11
Injection Date  : 7/25/2025 2:24:43 AM               Inj :   1
                                              Inj Volume : 20.000 µl
Method          : C:\Chem32\1\Data\purity_20250724_1 2025-07-24 15-10-18\Purity_original.M (
                  Sequence Method)
Last changed    : 10/21/2024 5:03:57 PM by JPLpublic
Additional Info : Peak(s) manually integrated
```

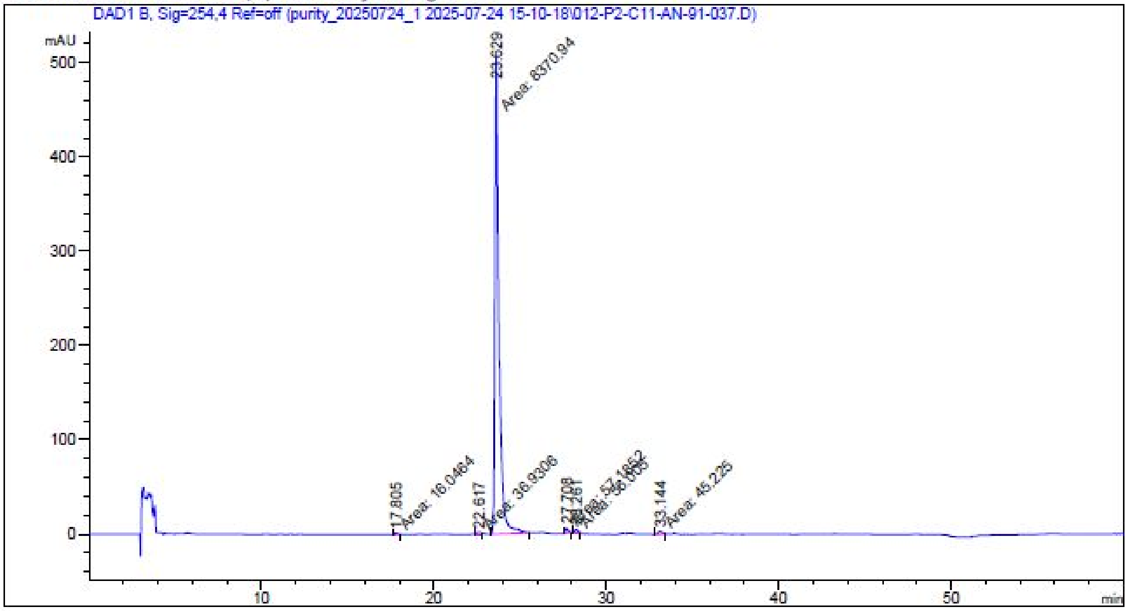


```
======================================================================
                         Area Percent Report
======================================================================

Sorted By             :      Signal
Multiplier            :      1.0000
Dilution              :      1.0000
Use Multiplier & Dilution Factor with ISTDs


Signal 1: DAD1 B, Sig=254,4 Ref=off
```

Peak #	RetTime [min]	Type	Width [min]	Area [mAU*s]	Height [mAU]	Area %
1	17.805	MM	0.1673	16.04637	1.59841	0.1870
2	22.617	MM	0.3080	36.93057	1.99838	0.4303
3	23.629	MM	0.2757	8370.94336	506.03143	97.5369
4	27.708	MM	0.1939	57.18519	4.91514	0.6663
5	28.261	MM	0.2799	56.00499	3.33532	0.6526
6	33.144	MM	0.2557	45.22496	2.94782	0.5270

HPLC data of compound 7

```
=====================================================================
Acq. Operator   : JPLpublic                        Seq. Line :   9
Acq. Instrument : FRD-LC-01                         Location :   P2-C-08
Injection Date  : 7/24/2025 11:21:03 PM                  Inj :   1
                                                  Inj Volume : 20.000 µl
Method          : C:\Chem32\1\Data\purity_20250724_1 2025-07-24 15-10-18\Purity_original.M (
                  Sequence Method)
Last changed    : 10/21/2024 5:03:57 PM by JPLpublic
Additional Info : Peak(s) manually integrated
```

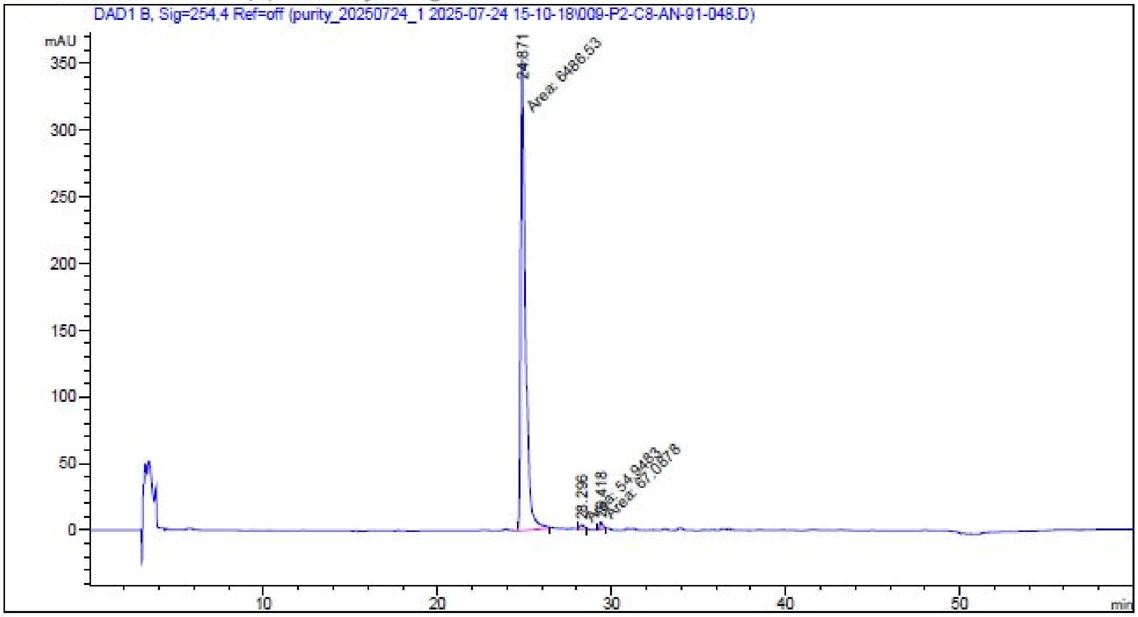


```
=====================================================================
                         Area Percent Report
=====================================================================

Sorted By             :      Signal
Multiplier            :      1.0000
Dilution              :      1.0000
Use Multiplier & Dilution Factor with ISTDs


Signal 1: DAD1 B, Sig=254,4 Ref=off

Peak RetTime Type  Width     Area      Height     Area
  #   [min]        [min]   [mAU*s]     [mAU]        %
----|-------|----|-------|----------|----------|--------|
   1  24.871 MM    0.3044 6486.52832  355.13440  98.1534
   2  28.296 MM    0.2830   54.94831    3.23552   0.8315
   3  29.418 MM    0.2155   67.08780    5.18756   1.0152
```

HPLC data of compound 8

```
=====================================================================
Acq. Operator   : JPLpublic                        Seq. Line :   5
Acq. Instrument : FRD-LC-01                         Location :   P2-C-04
Injection Date  : 7/24/2025 7:16:14 PM                   Inj :   1
                                                  Inj Volume : 20.000 µl
Method          : C:\Chem32\1\Data\purity_20250724_1 2025-07-24 15-10-18\Purity_original.M (
                  Sequence Method)
Last changed    : 10/21/2024 5:03:57 PM by JPLpublic
Additional Info : Peak(s) manually integrated
```

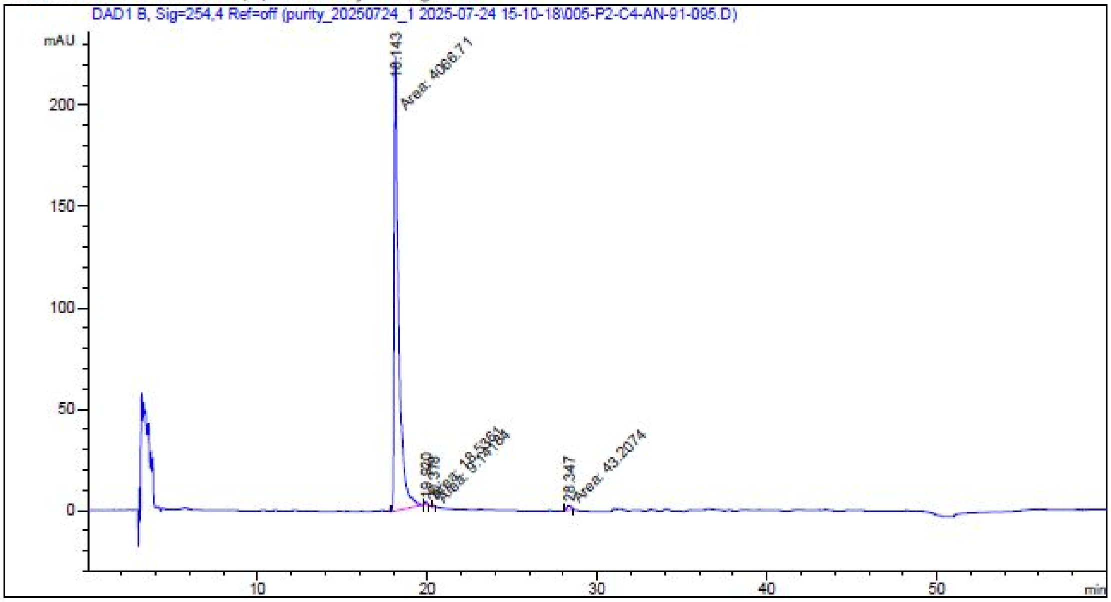


```
=====================================================================
                         Area Percent Report
=====================================================================

Sorted By             :      Signal
Multiplier            :      1.0000
Dilution              :      1.0000
Use Multiplier & Dilution Factor with ISTDs


Signal 1: DAD1 B, Sig=254,4 Ref=off
```

Peak #	RetTime [min]	Type	Width [min]	Area [mAU*s]	Height [mAU]	Area %
1	18.143	MM	0.3014	4066.70630	224.88983	98.2868
2	19.920	MM	0.1857	18.53606	1.66383	0.4480
3	20.378	MM	0.1491	9.14184	1.02199	0.2209
4	28.347	MM	0.2944	43.20744	2.44570	1.0443

HPLC data of compound 9

```
=====================================================================
Acq. Operator   : JPLpublic                        Seq. Line :    6
Acq. Instrument : FRD-LC-01                         Location :   P2-C-05
Injection Date  : 7/24/2025 8:17:26 PM                   Inj :    1
                                                  Inj Volume : 20.000 µl
Method          : C:\Chem32\1\Data\purity_20250724_1 2025-07-24 15-10-18\Purity_original.M (
                  Sequence Method)
Last changed    : 10/21/2024 5:03:57 PM by JPLpublic
Additional Info : Peak(s) manually integrated
```

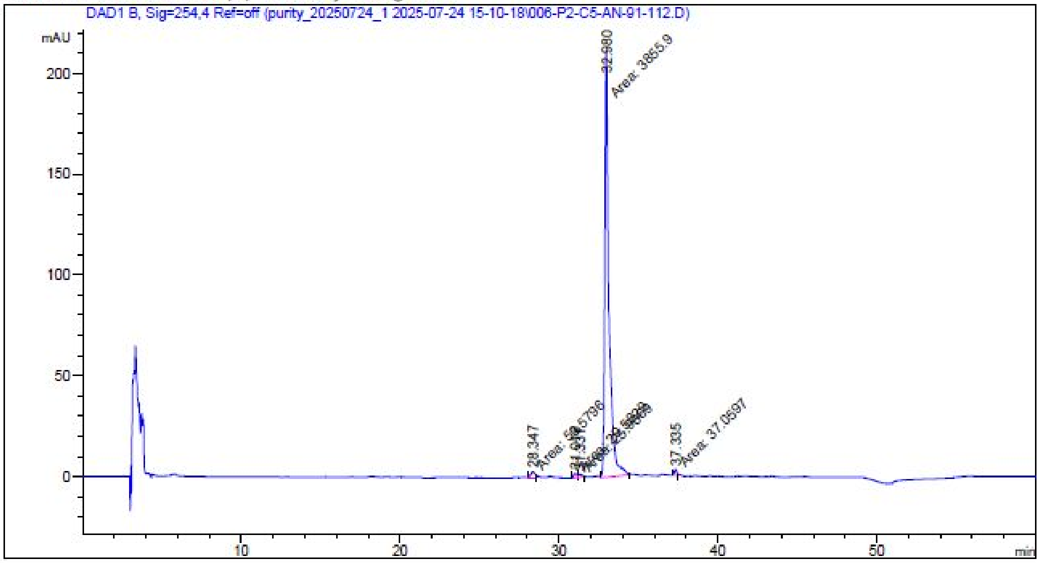


```
=====================================================================
                         Area Percent Report
=====================================================================

Sorted By             :      Signal
Multiplier            :      1.0000
Dilution              :      1.0000
Use Multiplier & Dilution Factor with ISTDs


Signal 1: DAD1 B, Sig=254,4 Ref=off
```

Peak #	RetTime [min]	Type	Width [min]	Area [mAU*s]	Height [mAU]	Area %
1	28.347	MM	0.3136	53.57955	2.84758	1.3388
2	31.019	MF	0.2309	29.58289	2.13531	0.7392
3	31.331	FM	0.2845	25.96693	1.32572	0.6488
4	32.980	MM	0.3051	3855.89771	210.64737	96.3472
5	37.335	MM	0.1927	37.05967	3.20511	0.9260

HPLC data of compound 10

```
=====================================================================
Acq. Operator   : JPLpublic                     Seq. Line :    6
Acq. Instrument : FRD-LC-01                      Location :   P1-D-06
Injection Date  : 7/23/2025 7:08:15 PM                Inj :    1
                                               Inj Volume : 20.000 µl
Method          : C:\Chem32\1\Data\purity_20250723-098-1 2025-07-23 14-00-47\Purity_original.
                  M (Sequence Method)
Last changed    : 10/21/2024 5:03:57 PM by JPLpublic
Additional Info : Peak(s) manually integrated
```

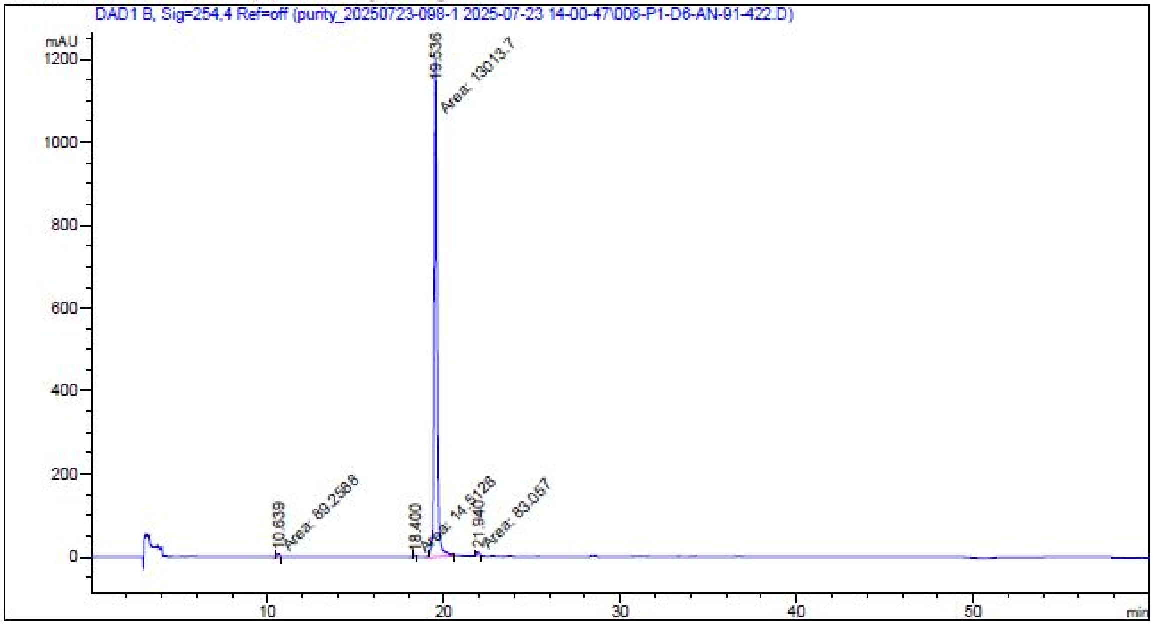


```
=====================================================================
                         Area Percent Report
=====================================================================

Sorted By             :      Signal
Multiplier            :      1.0000
Dilution              :      1.0000
Use Multiplier & Dilution Factor with ISTDs


Signal 1: DAD1 B, Sig=254,4 Ref=off
```

Peak #	RetTime [min]	Type	Width [min]	Area [mAU*s]	Height [mAU]	Area %
1	10.639	MM	0.1860	89.25884	7.99957	0.6762
2	18.400	MM	0.1023	14.51280	2.36395	0.1099
3	19.536	MM	0.1801	1.30137e4	1204.13660	98.5847
4	21.940	MM	0.1531	83.05701	9.04369	0.6292

HPLC data of compound 11

```
=====================================================================
Acq. Operator   : JPLpublic                        Seq. Line :  21
Acq. Instrument : FRD-LC-01                         Location :   P1-E-10
Injection Date  : 7/28/2025 10:35:52 AM                  Inj :   1
                                                  Inj Volume : 20.000 µl
Method          : C:\Chem32\1\Data\purity_20250727-098_1 2025-07-27 14-09-26\Purity_original.
                  M (Sequence Method)
Last changed    : 10/21/2024 5:03:57 PM by JPLpublic
Additional Info : Peak(s) manually integrated
```

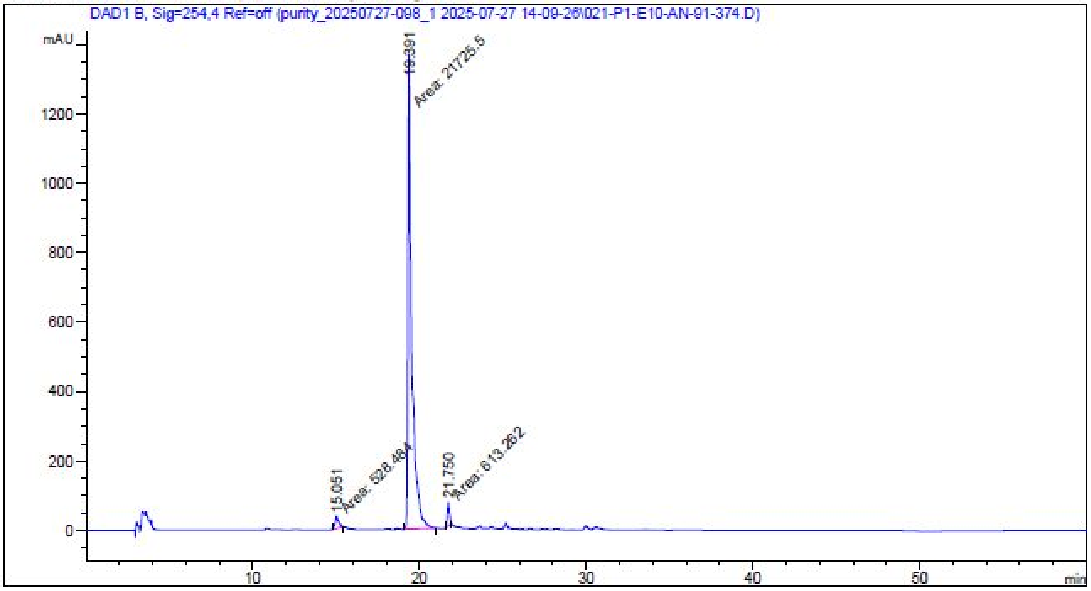


```
=====================================================================
                         Area Percent Report
=====================================================================

Sorted By             :      Signal
Multiplier            :      1.0000
Dilution              :      1.0000
Use Multiplier & Dilution Factor with ISTDs


Signal 1: DAD1 B, Sig=254,4 Ref=off
```

Peak #	RetTime [min]	Type	Width [min]	Area [mAU*s]	Height [mAU]	Area %
1	15.051	MM	0.2590	528.48358	34.01020	2.3111
2	19.391	MM	0.2647	2.17255e4	1367.87976	95.0071
3	21.750	MM	0.1523	613.26160	67.12917	2.6818

HPLC data of compound 12

```
=====================================================================
Acq. Operator   : JPLpublic                        Seq. Line :  13
Acq. Instrument : FRD-LC-01                          Location :   P1-F-03
Injection Date  : 8/22/2025 6:59:54 AM                    Inj :   1
                                                   Inj Volume : 20.000 µl
Method          : C:\Chem32\1\Data\Purity_20250821_SD6 2025-08-21 18-43-18\Purity_original.M
                  (Sequence Method)
Last changed    : 10/21/2024 5:03:57 PM by JPLpublic
Sample Info     : Buff_10mM_NH4OAC in H2O

Additional Info : Peak(s) manually integrated
```

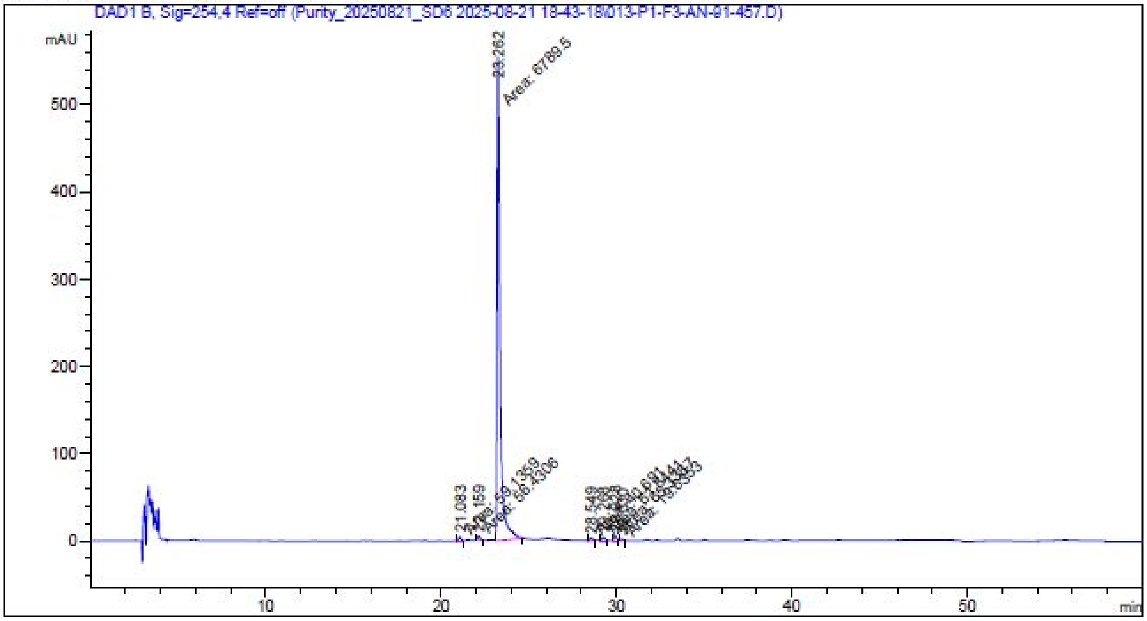


```
=====================================================================
                         Area Percent Report
=====================================================================

Sorted By             :      Signal
Multiplier            :      1.0000
Dilution              :      1.0000
Use Multiplier & Dilution Factor with ISTDs


Signal 1: DAD1 B, Sig=254,4 Ref=off
```

Peak #	RetTime [min]	Type	Width [min]	Area [mAU*s]	Height [mAU]	Area %
1	21.083	MM	0.1734	59.13590	5.68312	0.8338
2	22.159	MM	0.1886	56.43057	4.98615	0.7956
3	23.262	MM	0.2037	6789.50439	555.64111	95.7269
4	28.549	MM	0.2023	40.69101	3.35217	0.5737
5	29.268	MM	0.2463	61.84413	4.18555	0.8720
6	29.928	MM	0.1941	65.33865	5.60928	0.9212
7	30.330	MM	0.1737	19.63531	1.88375	0.2768

HPLC data of compound 13

```
=====================================================================
Acq. Operator   : JPLpublic                     Seq. Line :  15
Acq. Instrument : FRD-LC-01                      Location :   P1-F-05
Injection Date  : 8/22/2025 9:02:28 AM                Inj :    1
                                               Inj Volume : 20.000 µl
Method          : C:\Chem32\1\Data\Purity_20250821_SD6 2025-08-21 18-43-18\Purity_original.M
                  (Sequence Method)
Last changed    : 10/21/2024 5:03:57 PM by JPLpublic
Sample Info     : Buff_10mM_NH4OAC in H2O

Additional Info : Peak(s) manually integrated
```

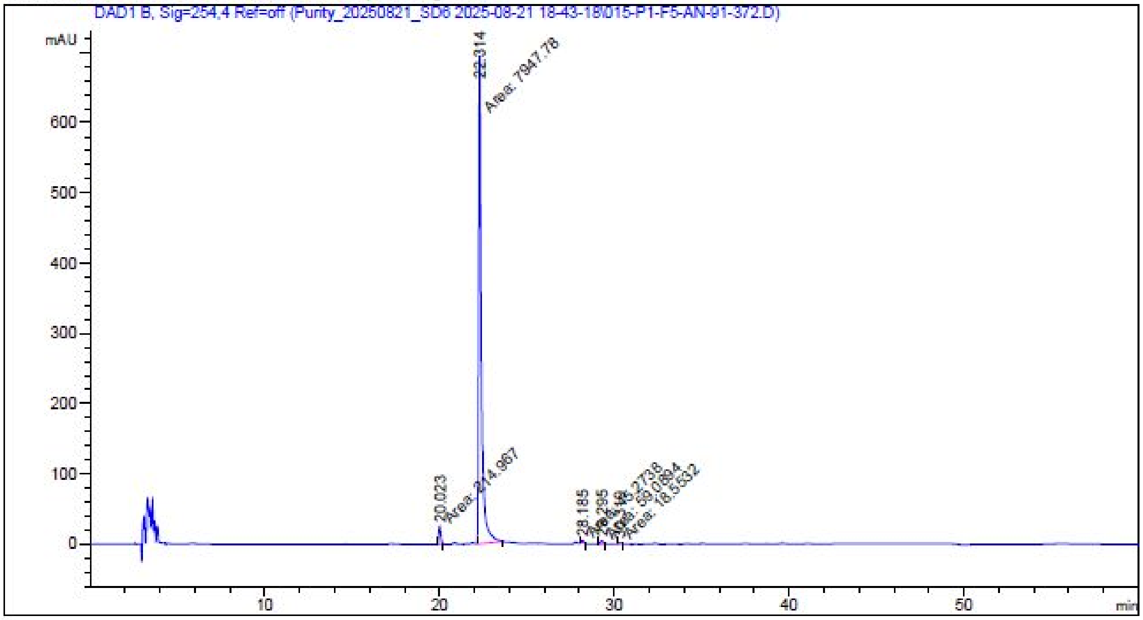


```
=====================================================================
                         Area Percent Report
=====================================================================

Sorted By             :      Signal
Multiplier            :      1.0000
Dilution              :      1.0000
Use Multiplier & Dilution Factor with ISTDs


Signal 1: DAD1 B, Sig=254,4 Ref=off
```

Peak #	RetTime [min]	Type	Width [min]	Area [mAU*s]	Height [mAU]	Area %
1	20.023	MM	0.1425	214.96730	25.14657	2.5944
2	22.314	MM	0.1907	7947.77881	694.78076	95.9221
3	28.185	MM	0.1830	45.27376	4.12261	0.5464
4	29.295	MM	0.2446	59.08940	4.02648	0.7132
5	30.319	MM	0.1602	18.55323	1.92967	0.2239

HPLC data of compound 14

```
=====================================================================
Acq. Operator   : JPLpublic                     Seq. Line :   5
Acq. Instrument : FRD-LC-01                      Location :   P1-D-05
Injection Date  : 7/23/2025 6:06:58 PM                Inj :   1
                                               Inj Volume : 20.000 µl
Method          : C:\Chem32\1\Data\purity_20250723-098-1 2025-07-23 14-00-47\Purity_original.
                  M (Sequence Method)
Last changed    : 10/21/2024 5:03:57 PM by JPLpublic
Additional Info : Peak(s) manually integrated
```

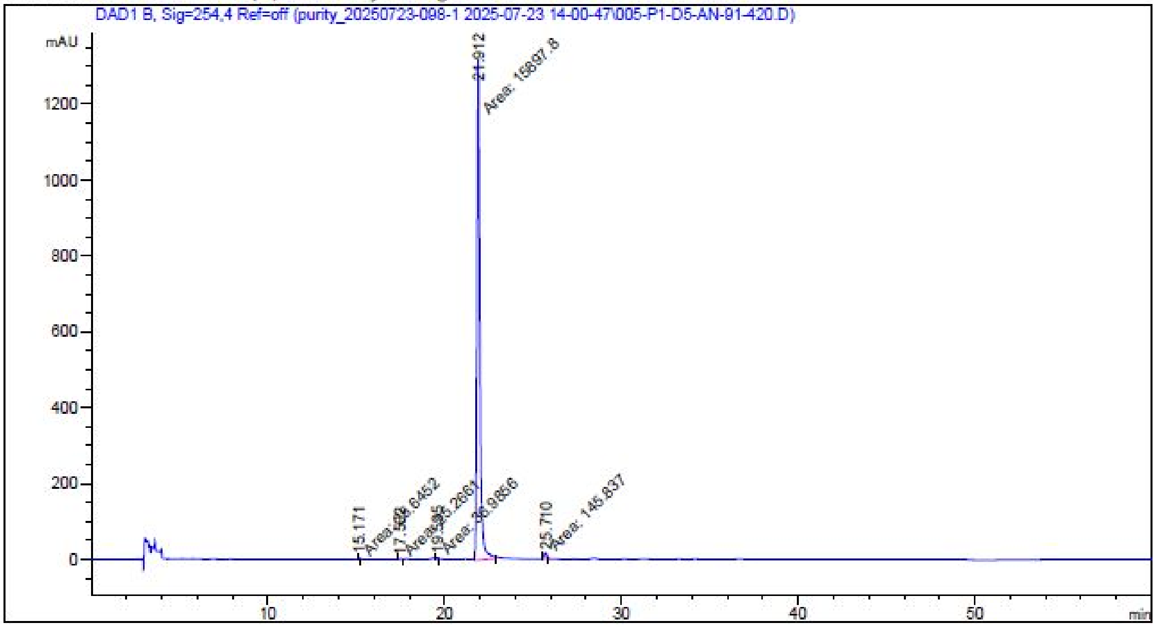


```
=====================================================================
                         Area Percent Report
=====================================================================

Sorted By             :      Signal
Multiplier            :      1.0000
Dilution              :      1.0000
Use Multiplier & Dilution Factor with ISTDs


Signal 1: DAD1 B, Sig=254,4 Ref=off
```

Peak #	RetTime [min]	Type	Width [min]	Area [mAU*s]	Height [mAU]	Area %
1	15.171	MM	0.1255	25.64520	3.40441	0.1590
2	17.502	MM	0.1744	25.26609	2.41511	0.1566
3	19.595	MM	0.1384	36.98560	4.45472	0.2293
4	21.912	MM	0.1999	1.58978e4	1325.46887	98.5511
5	25.710	MM	0.1814	145.83727	13.40154	0.9041

HPLC data of compound 15

```
=====================================================================
Acq. Operator   : JPLpublic                      Seq. Line :  14
Acq. Instrument : FRD-LC-01                       Location :   P1-F-04
Injection Date  : 8/22/2025 8:01:11 AM                 Inj :   1
                                                Inj Volume : 20.000 µl
Method          : C:\Chem32\1\Data\Purity_20250821_SD6 2025-08-21 18-43-18\Purity_original.M
                  (Sequence Method)
Last changed    : 10/21/2024 5:03:57 PM by JPLpublic
Sample Info     : Buff_10mM_NH4OAC in H2O

Additional Info : Peak(s) manually integrated
```

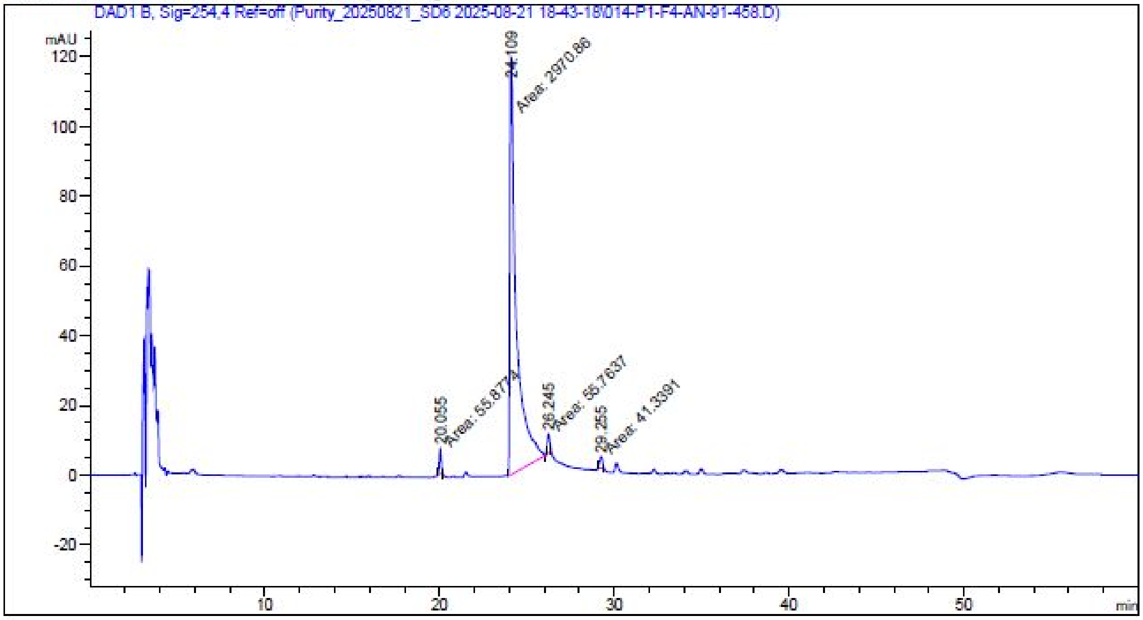


```
=====================================================================
                         Area Percent Report
=====================================================================

Sorted By             :      Signal
Multiplier            :      1.0000
Dilution              :      1.0000
Use Multiplier & Dilution Factor with ISTDs


Signal 1: DAD1 B, Sig=254,4 Ref=off
```

Peak #	RetTime [min]	Type	Width [min]	Area [mAU*s]	Height [mAU]	Area %
1	20.055	MM	0.1295	55.87740	7.19382	1.7887
2	24.109	MM	0.4125	2970.86084	120.03093	95.1028
3	26.245	MM	0.1724	55.76366	5.39094	1.7851
4	29.255	MM	0.2030	41.33911	3.39359	1.3233

HPLC data of compound 16

===

```
Acq. Operator   : JPLpublic                    Seq. Line :  22
Acq. Instrument : FRD-LC-01                     Location :   P1-E-11
Injection Date  : 7/28/2025 11:37:05 AM              Inj :   1
                                              Inj Volume : 20.000 µl
Method          : C:\Chem32\1\Data\purity_20250727-098_1 2025-07-27 14-09-26\Purity_original.
                  M (Sequence Method)
Last changed    : 10/21/2024 5:03:57 PM by JPLpublic
Additional Info : Peak(s) manually integrated
```

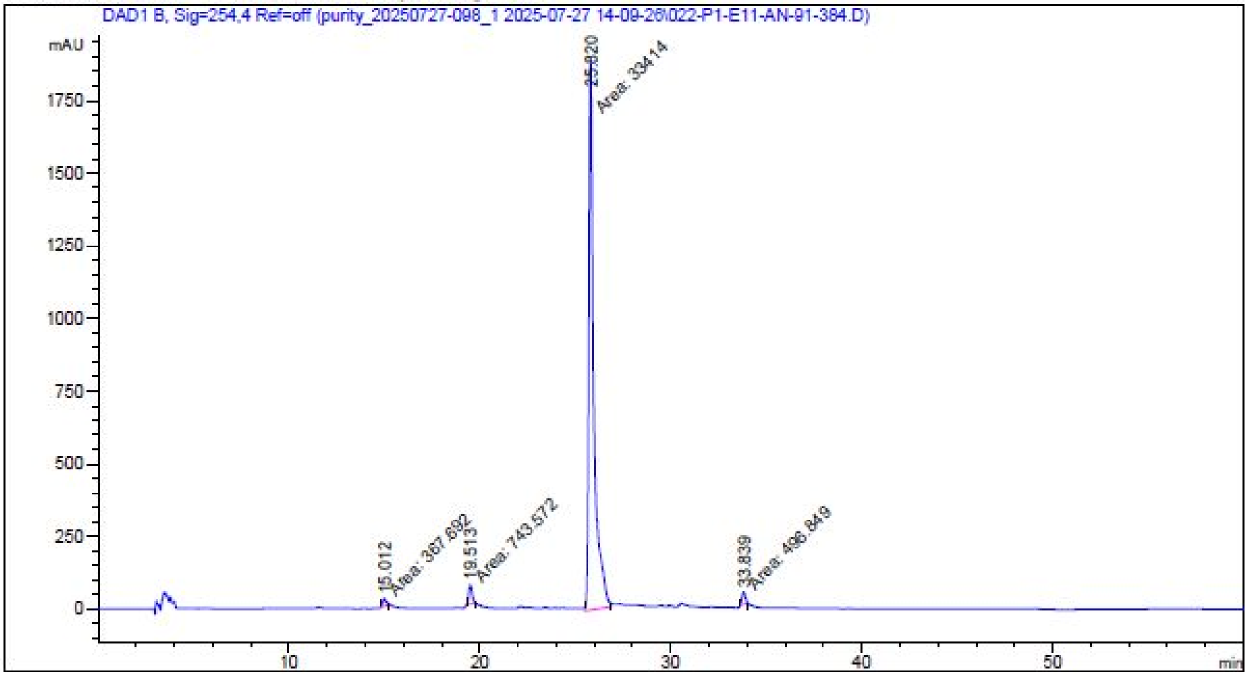

===

Area Percent Report

===

Sorted By : Signal
Multiplier : 1.0000
Dilution : 1.0000
Use Multiplier & Dilution Factor with ISTDs

Signal 1: DAD1 B, Sig=254,4 Ref=off

Peak #	RetTime [min]	Type	Width [min]	Area [mAU*s]	Height [mAU]	Area %
1	15.012	MM	0.2245	367.69217	27.29918	1.0499
2	19.513	MM	0.1875	743.57220	66.10537	2.1231
3	25.820	MM	0.2955	3.34140e4	1884.87195	95.4083
4	33.839	MM	0.2013	496.84900	41.13032	1.4187

HPLC data of compound 17

```
=====================================================================
Acq. Operator   : JPLpublic                       Seq. Line :  12
Acq. Instrument : FRD-LC-01                        Location :   P1-F-02
Injection Date  : 8/22/2025 5:58:37 AM                  Inj :   1
                                               Inj Volume : 20.000 µl
Method          : C:\Chem32\1\Data\Purity_20250821_SD6 2025-08-21 18-43-18\Purity_original.M
                  (Sequence Method)
Last changed    : 10/21/2024 5:03:57 PM by JPLpublic
Sample Info     : Buff_10mM_NH4OAC in H2O

Additional Info : Peak(s) manually integrated
```

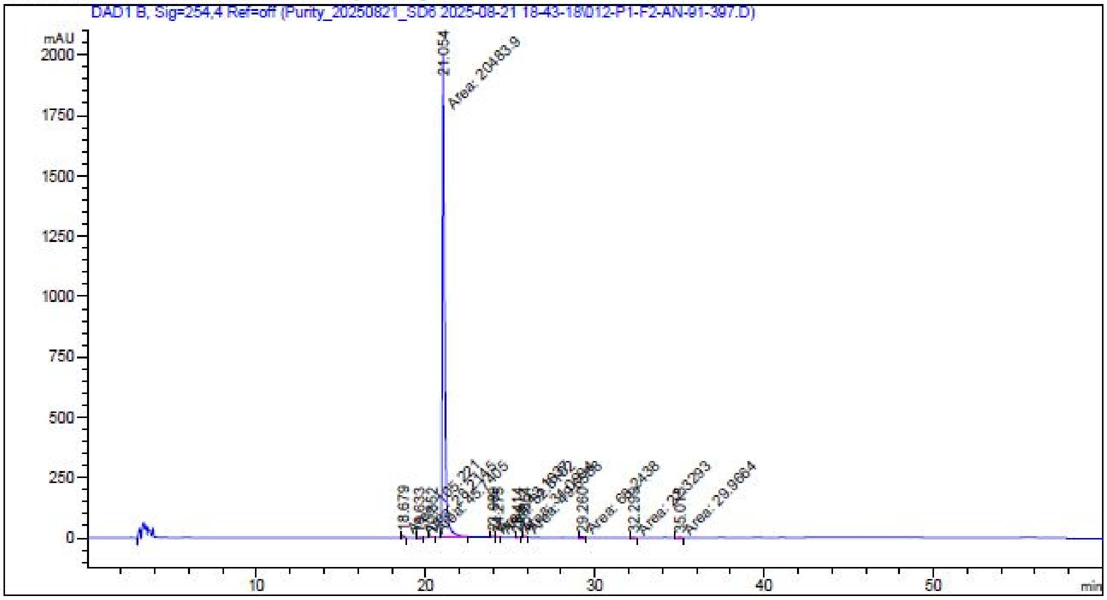


```
=====================================================================
                          Area Percent Report
=====================================================================

Sorted By             :      Signal
Multiplier            :      1.0000
Dilution              :      1.0000
Use Multiplier & Dilution Factor with ISTDs


Signal 1: DAD1 B, Sig=254,4 Ref=off
```

Peak #	RetTime [min]	Type	Width [min]	Area [mAU*s]	Height [mAU]	Area %
1	18.679	MM	0.1528	105.22089	11.47557	0.5025
2	19.633	MM	0.2401	28.21454	1.95865	0.1347
3	20.352	MM	0.2701	45.74054	2.82245	0.2184
4	21.054	MM	0.1705	2.04839e4	2002.10315	97.8216
5	23.989	MM	0.1631	43.16374	4.41014	0.2061
6	24.275	MM	0.1712	32.51023	3.16449	0.1553
7	25.414	MM	0.1920	31.09944	2.69990	0.1485

HPLC data of compound 18

```
=====================================================================
Acq. Operator   : JPLpublic                        Seq. Line :  20
Acq. Instrument : FRD-LC-01                         Location :   P1-E-08
Injection Date  : 9/11/2025 7:01:01 AM                   Inj :   1
                                                  Inj Volume : 20.000 µl
Method          : C:\Chem32\1\Data\Purity_20250910_98_Lara 2025-09-10 11-35-25\Purity_
                  original.M (Sequence Method)
Last changed    : 10/21/2024 5:03:57 PM by JPLpublic
Additional Info : Peak(s) manually integrated
```

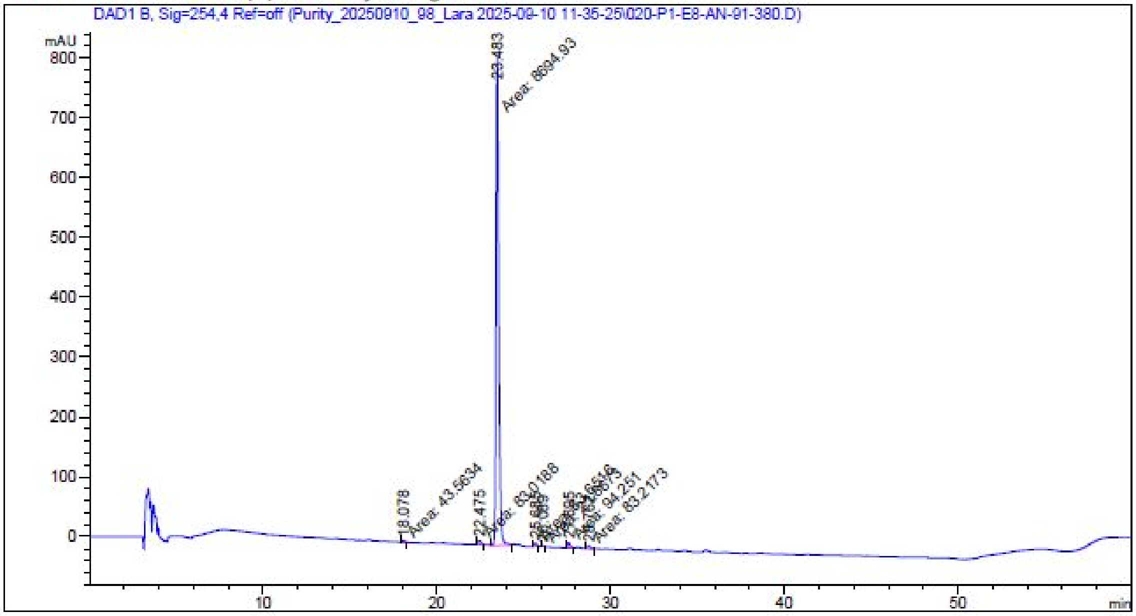


```
=====================================================================
                         Area Percent Report
=====================================================================

Sorted By             :      Signal
Multiplier            :      1.0000
Dilution              :      1.0000
Use Multiplier & Dilution Factor with ISTDs


Signal 1: DAD1 B, Sig=254,4 Ref=off
```

Peak #	RetTime [min]	Type	Width [min]	Area [mAU*s]	Height [mAU]	Area %
1	18.078	MM	0.1774	43.56336	4.09207	0.4795
2	22.475	MM	0.2296	83.01884	6.02565	0.9139
3	23.483	MM	0.1775	8694.92969	816.60504	95.7117
4	25.685	MM	0.1813	63.65160	5.85217	0.7007
5	26.089	MM	0.1618	21.86728	2.25217	0.2407
6	27.595	MM	0.1778	94.25098	8.83663	1.0375
7	28.762	MM	0.2944	83.21733	4.71169	0.9160

HRMS data of compound 1

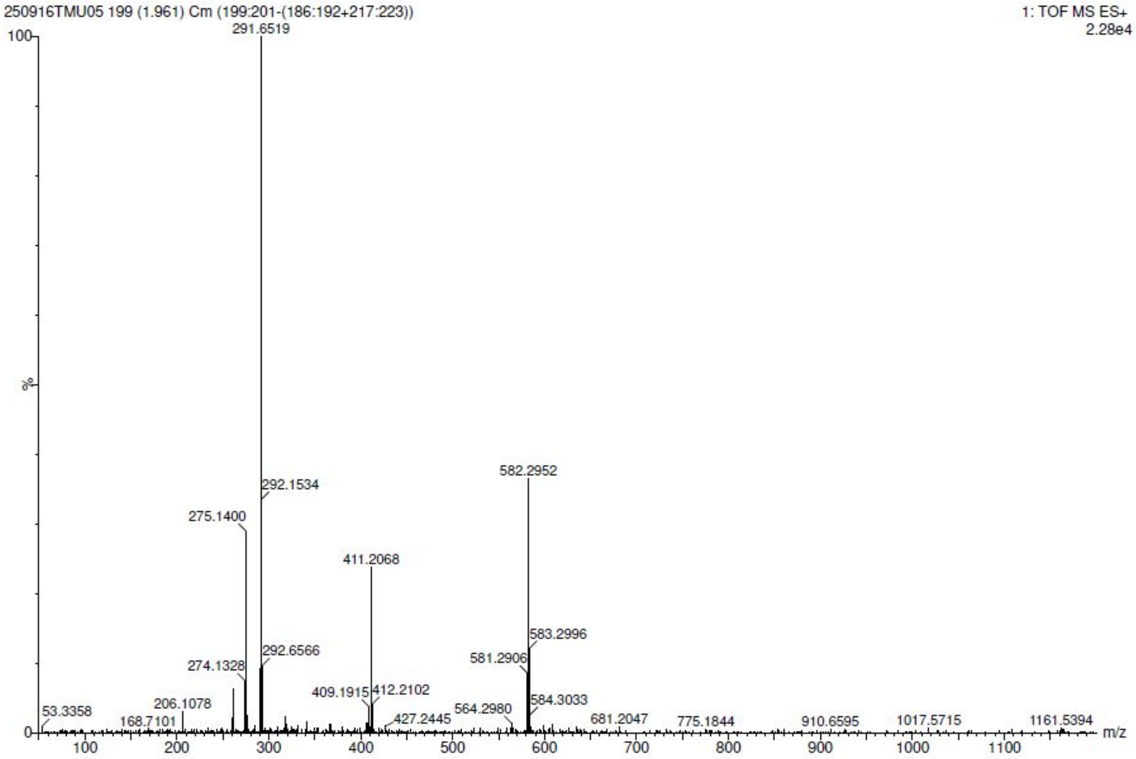

HRMS data of compound 2

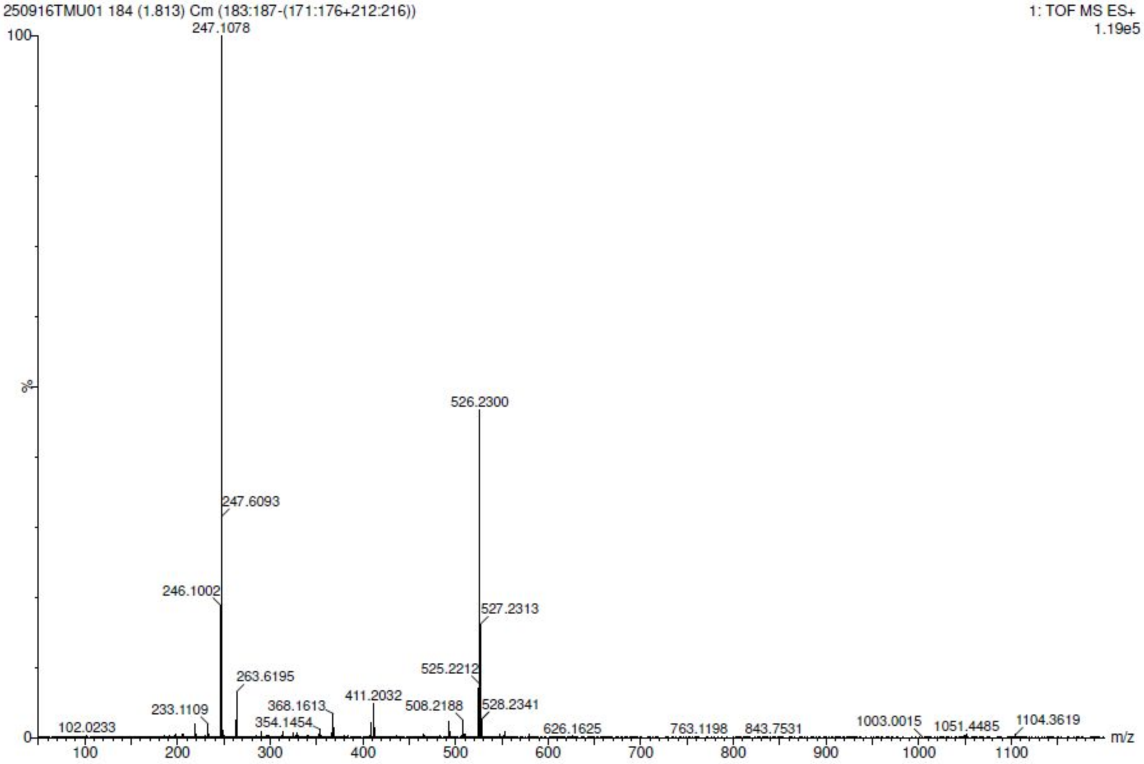

HRMS data of compound 3

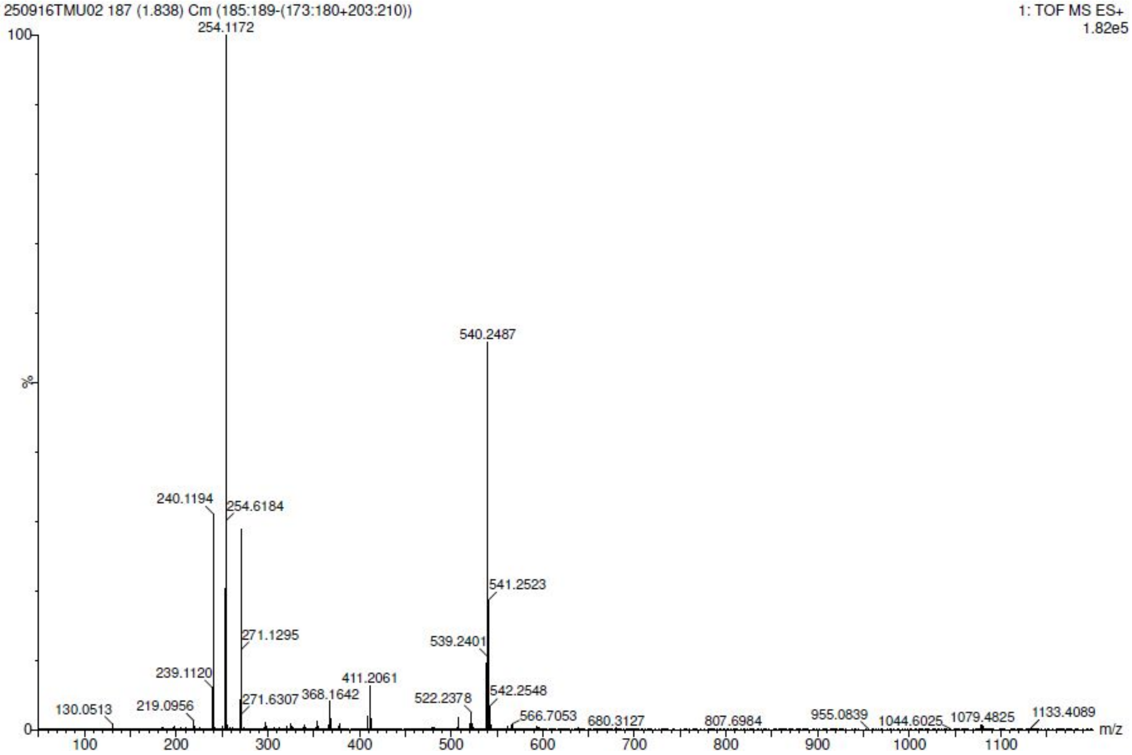

HRMS data of compound 4

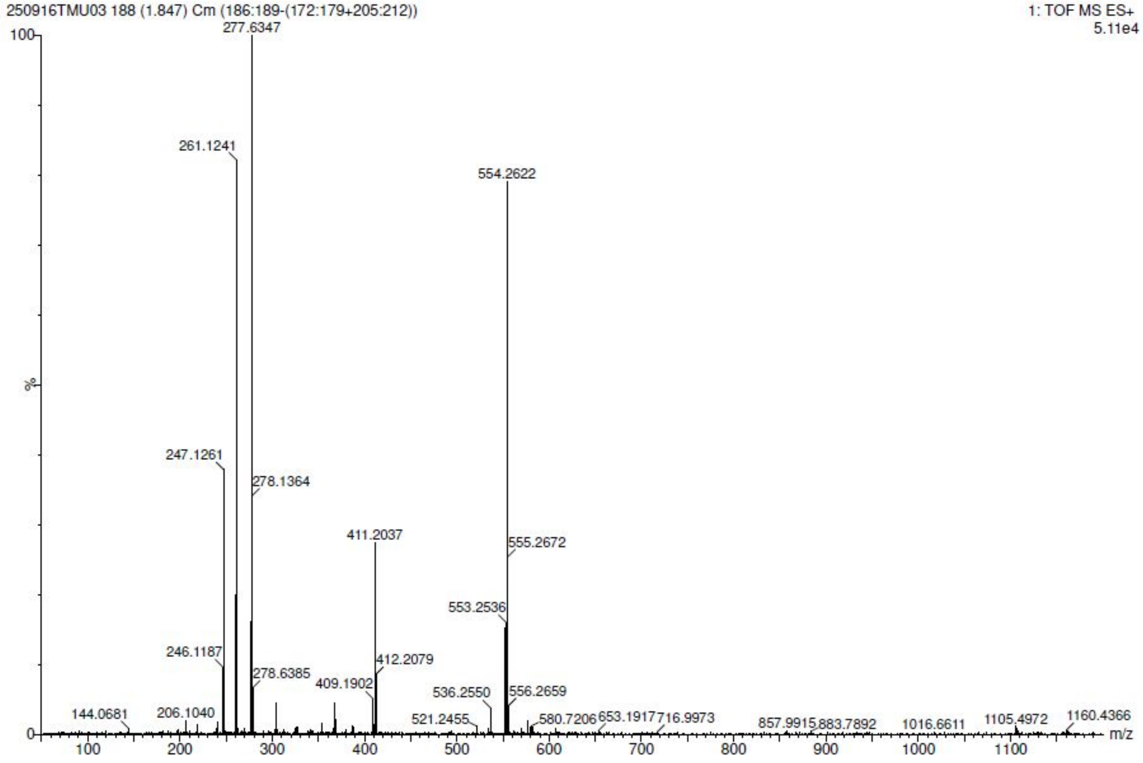

HRMS data of compound 5

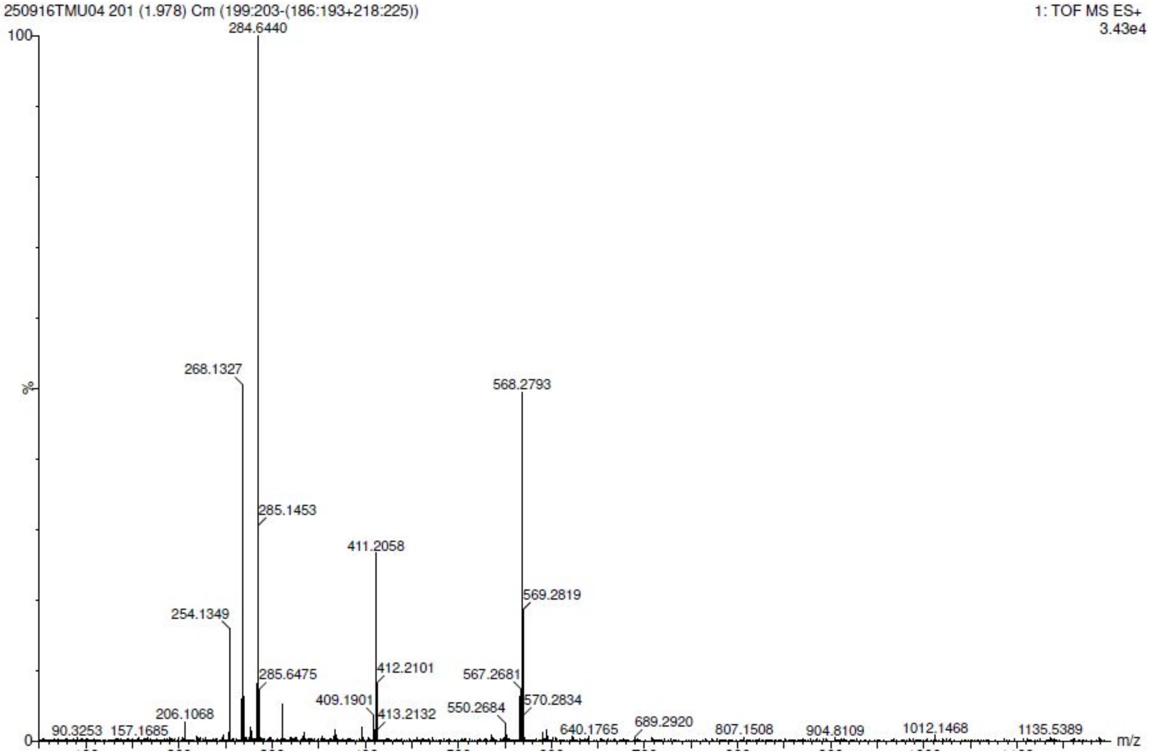

HRMS data of compound 6

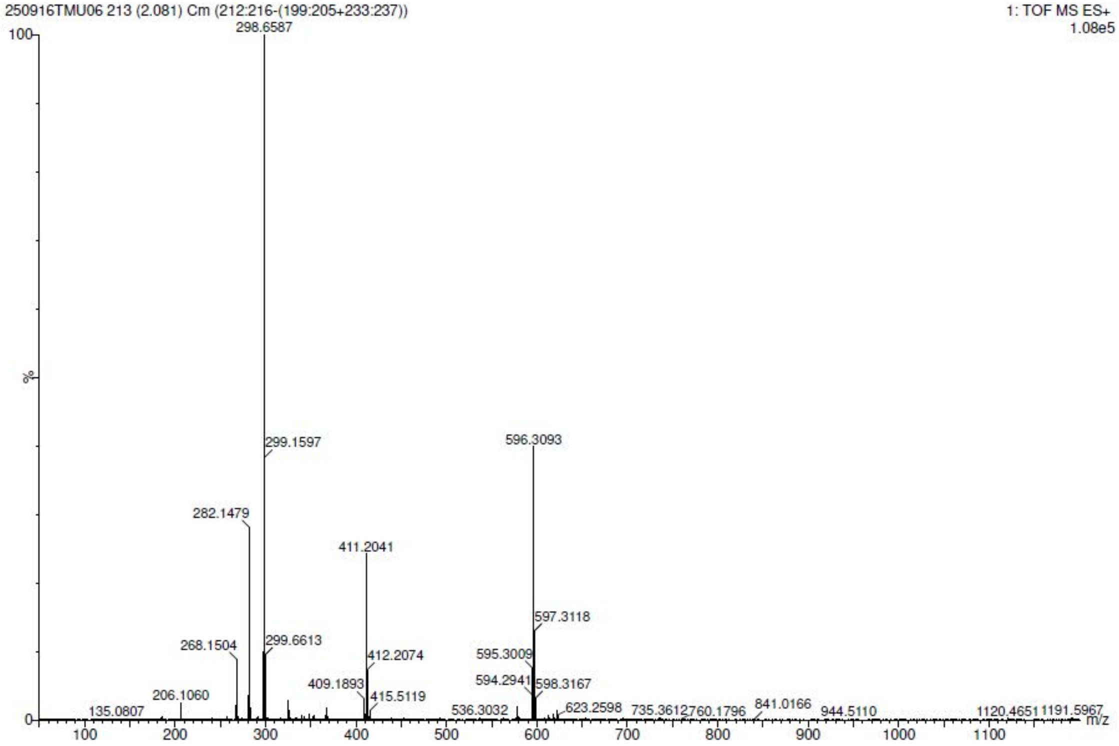

HRMS data of compound 7

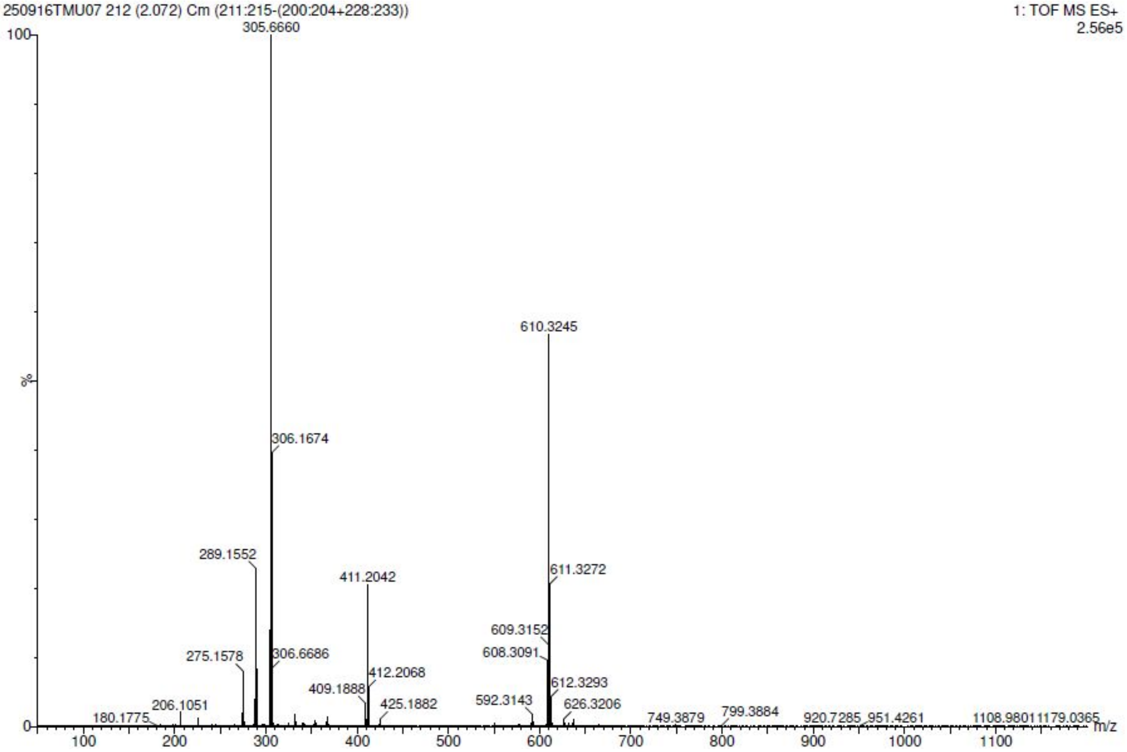

HRMS data of compound 8

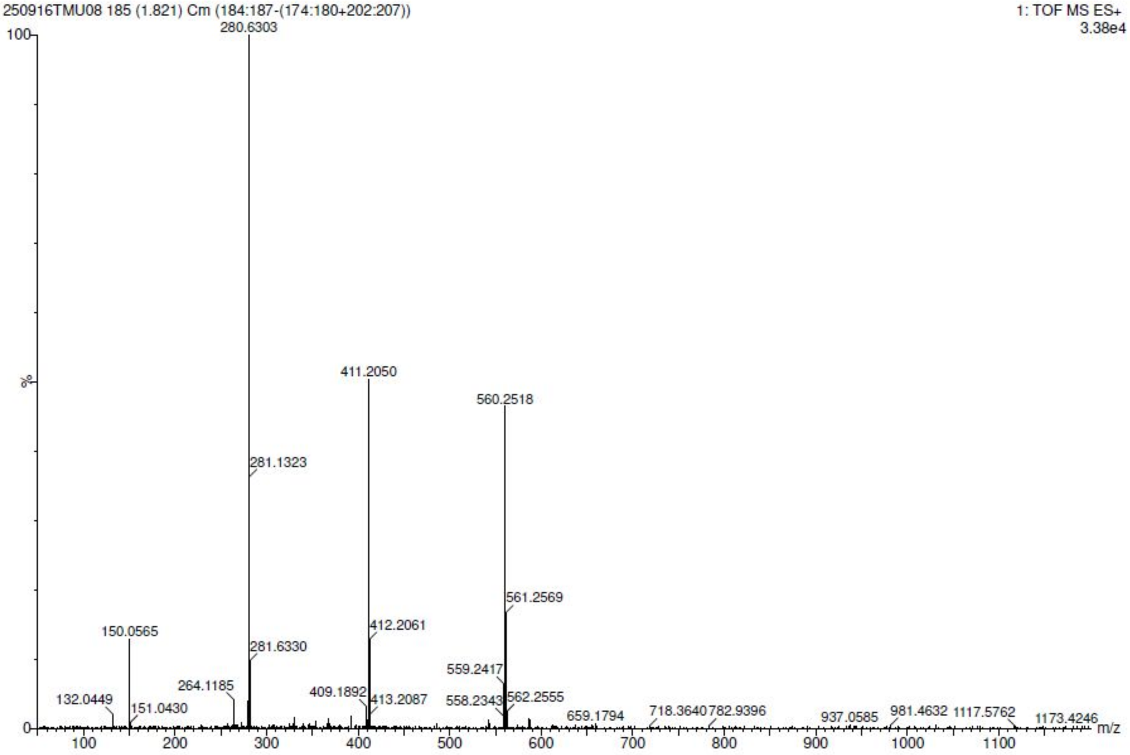

HRMS data of compound 9

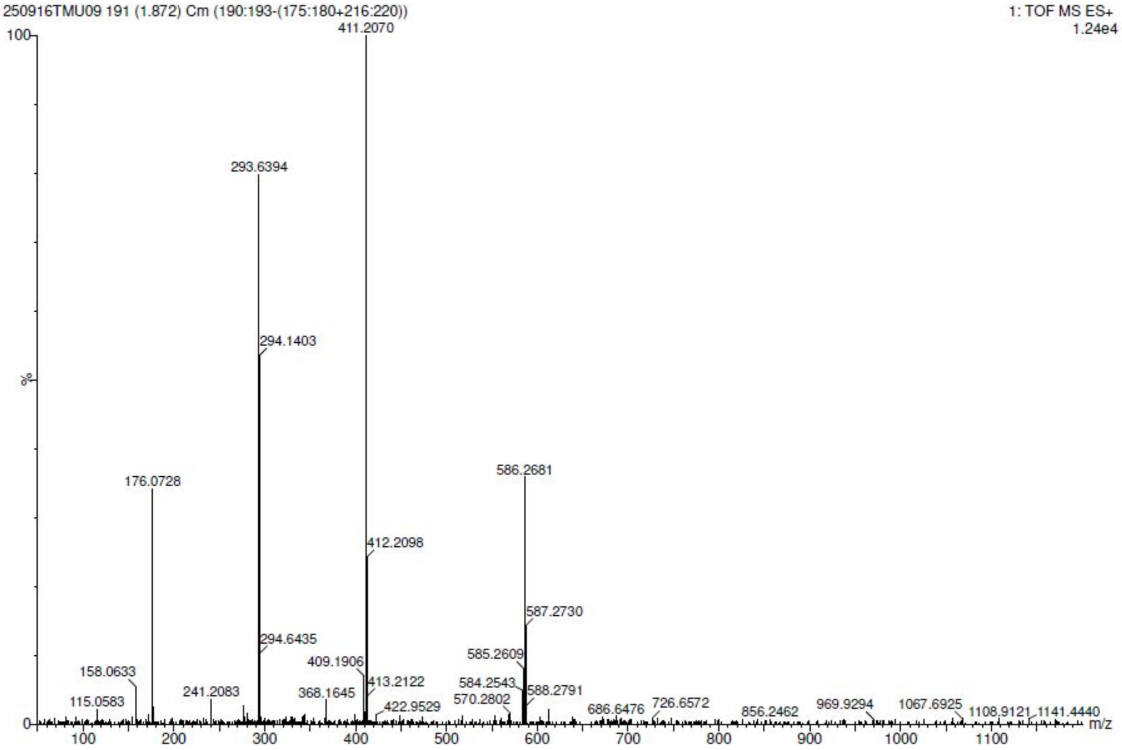

HRMS data of compound 10

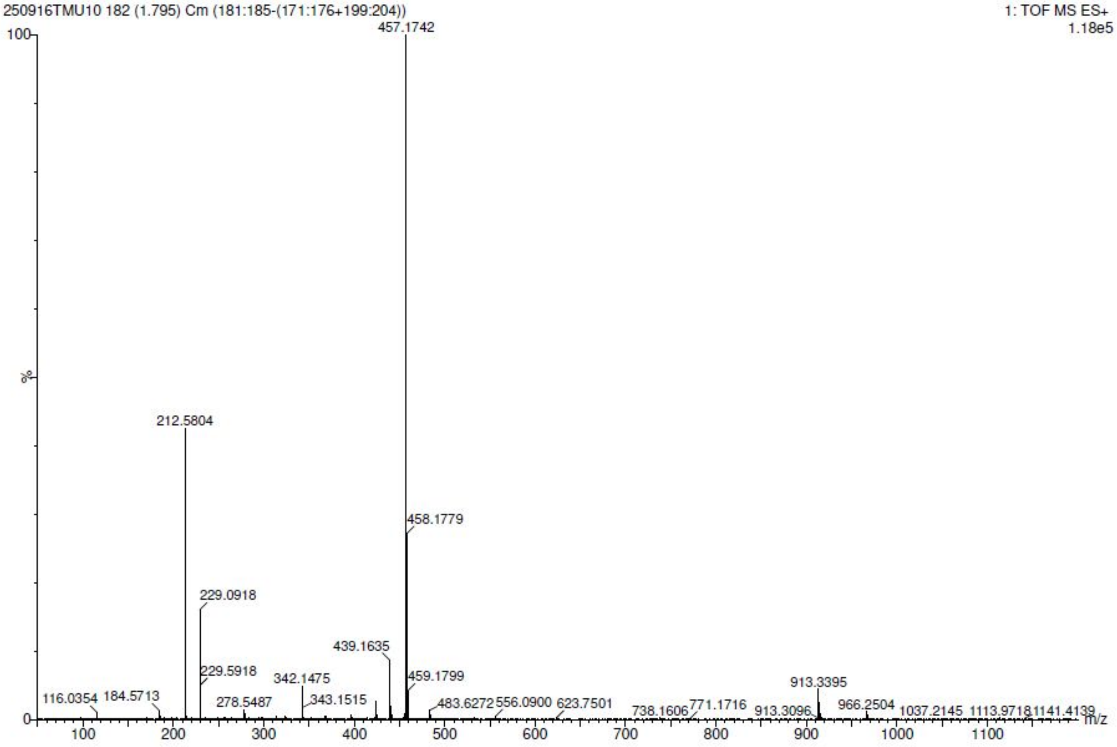

HRMS data of compound 11

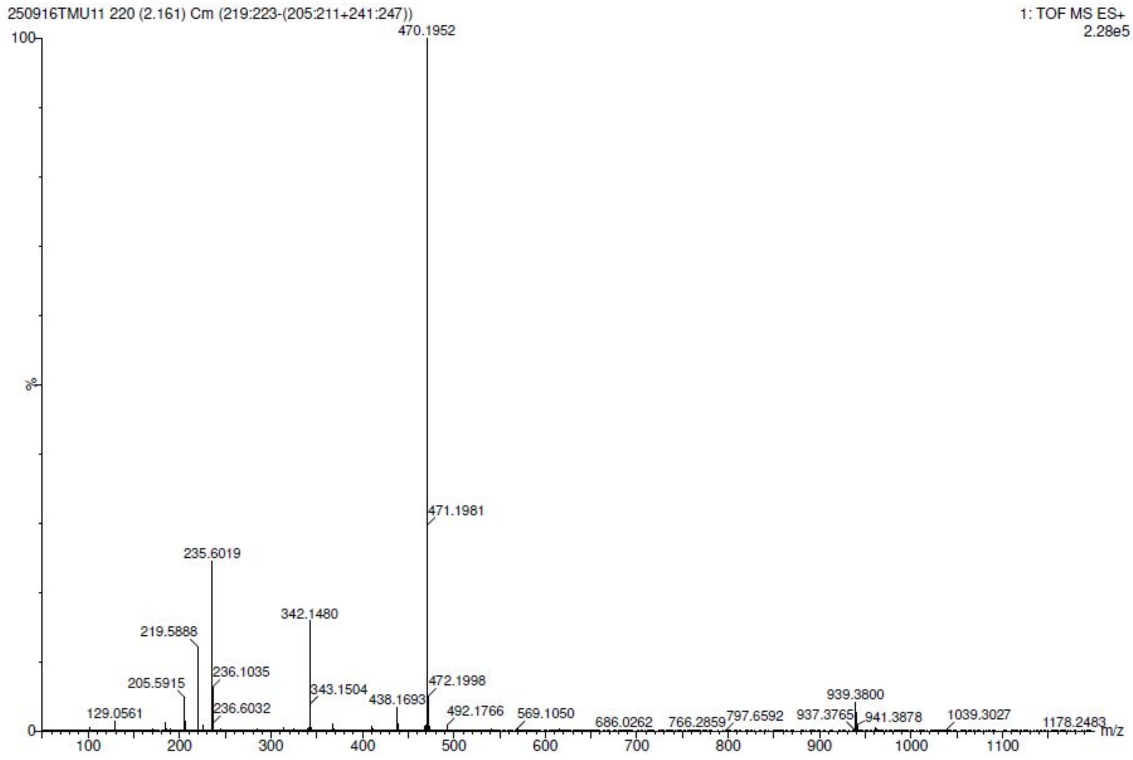

HRMS data of compound 12

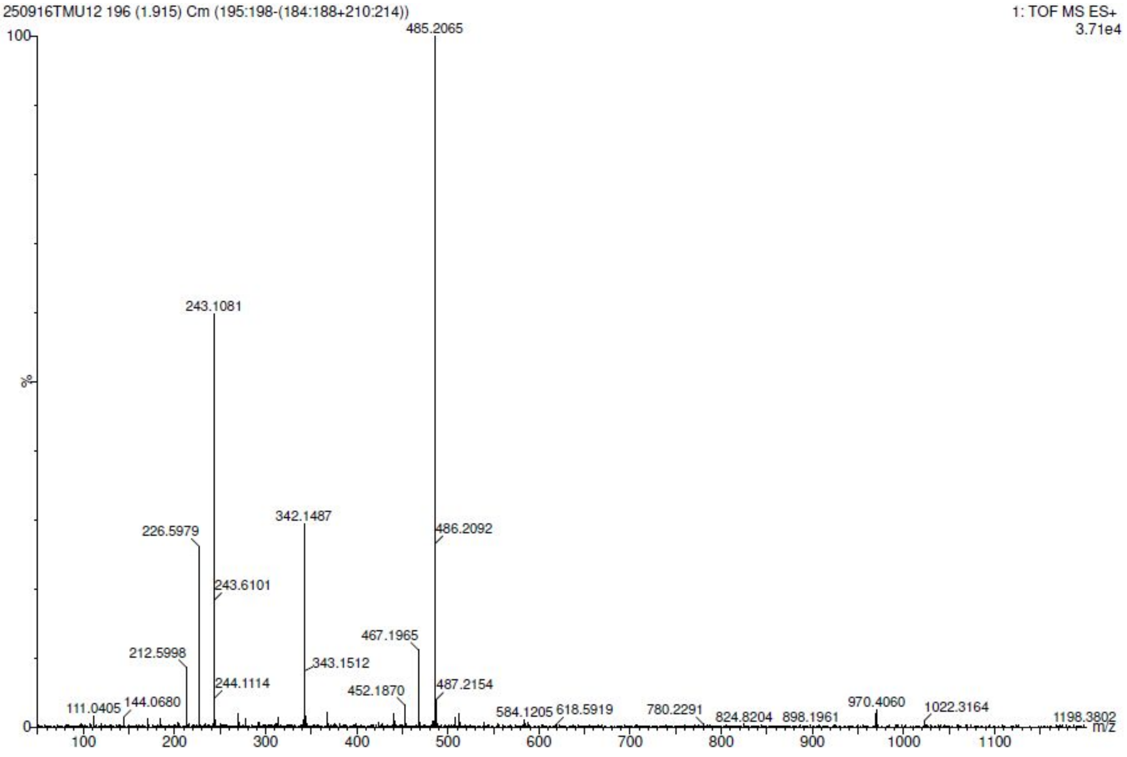

HRMS data of compound 13

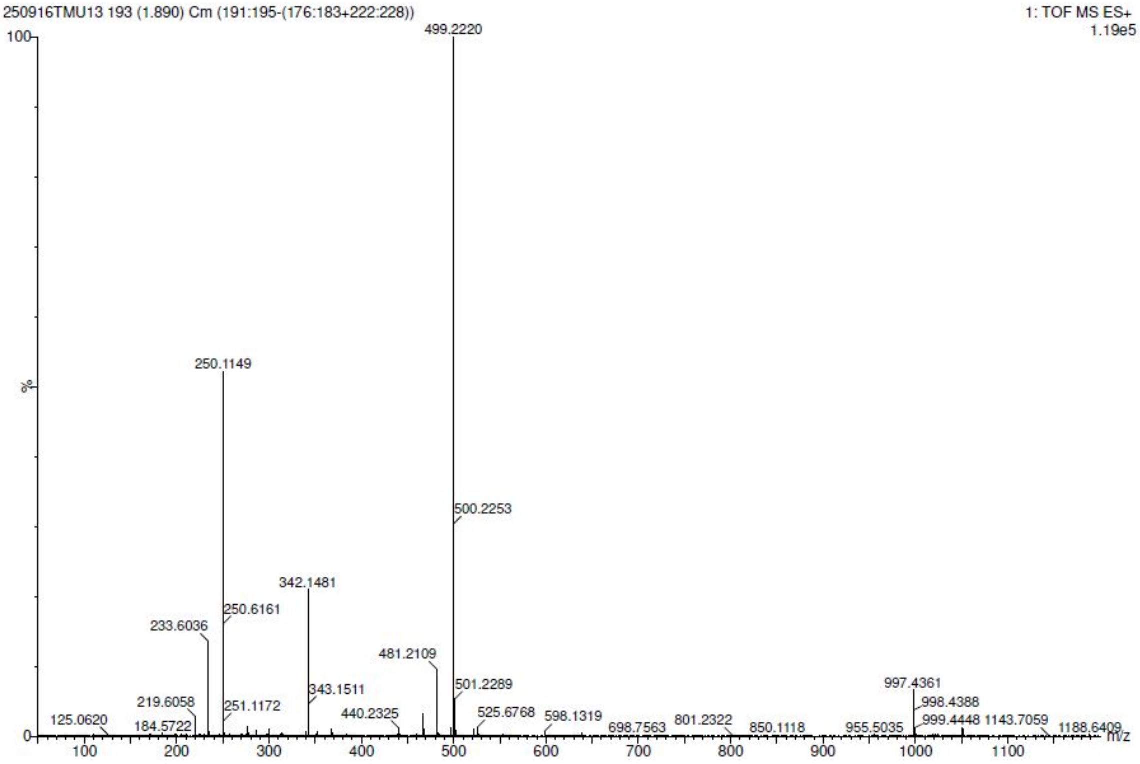

HRMS data of compound 14

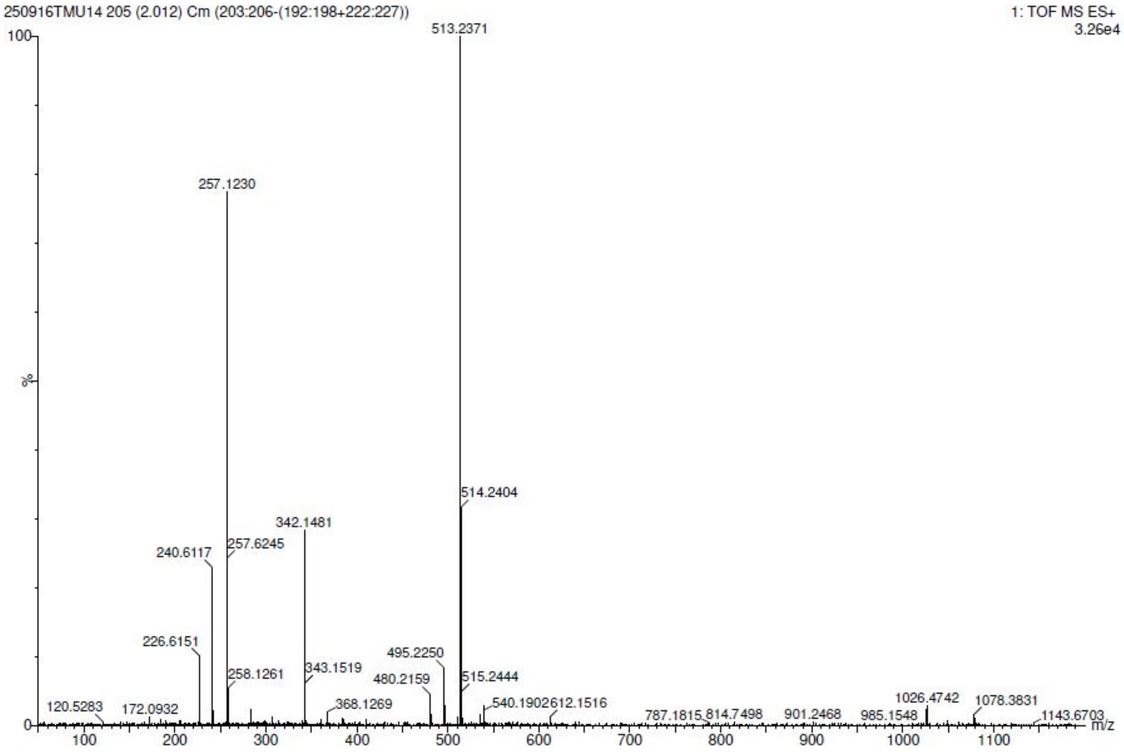

HRMS data of compound 15

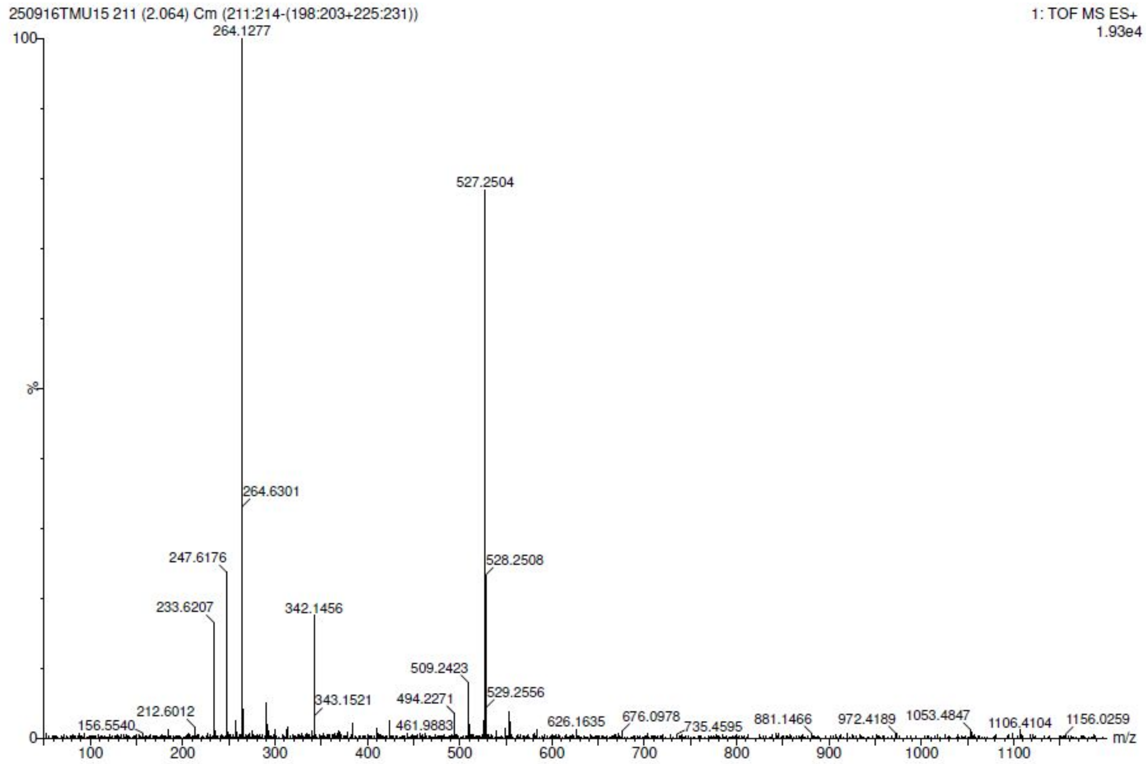

HRMS data of compound 16

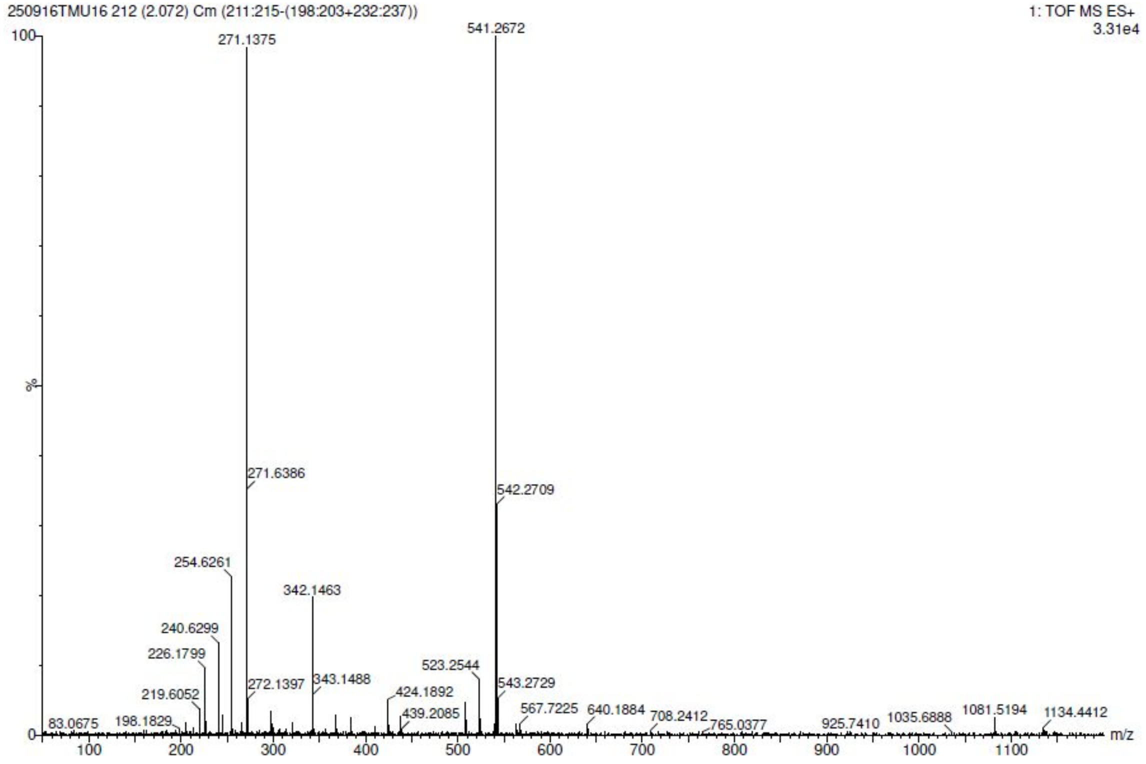

HRMS data of compound 17

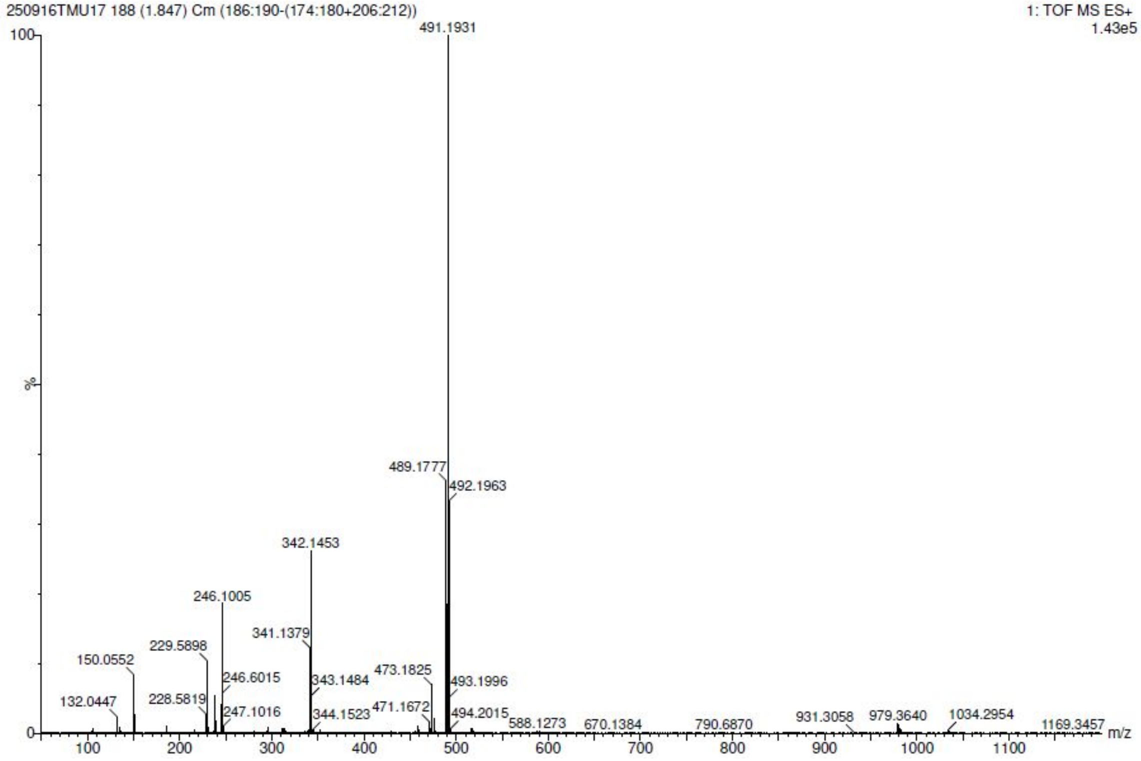

HRMS data of compound 18

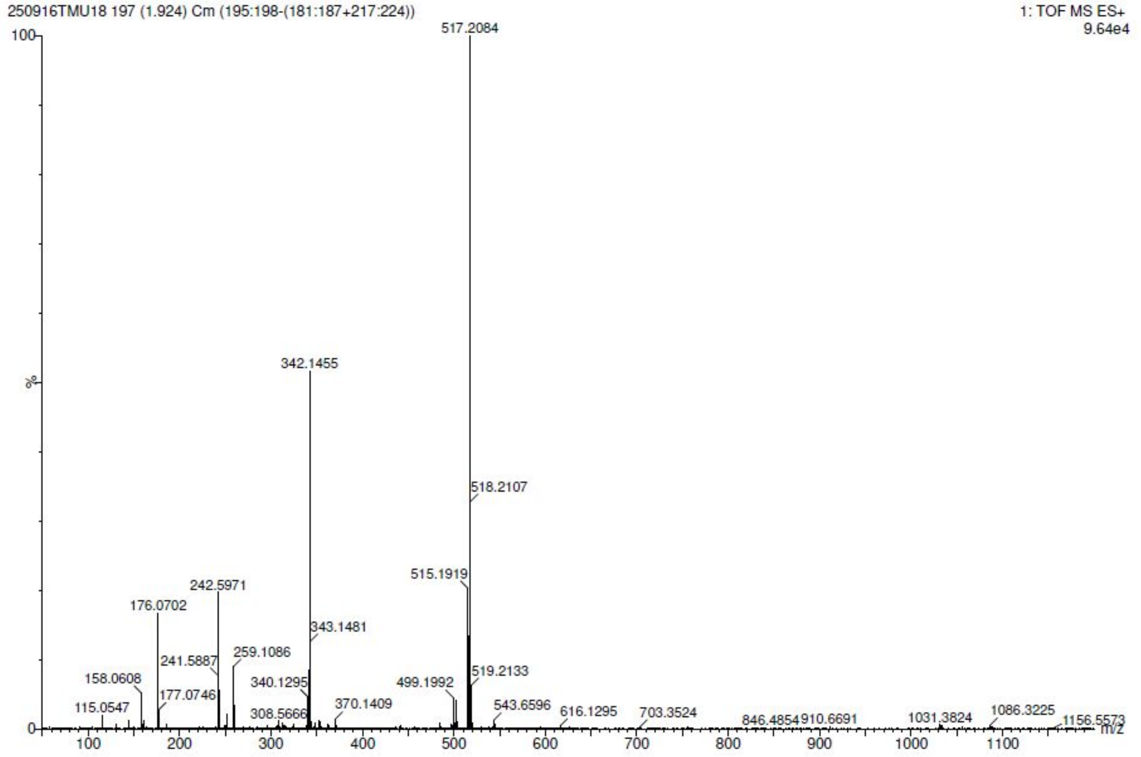

Supplement: Supplementary file 1 [file jm6c00039_si_001.pdf]
